# Systematic review and meta-analysis of COVID-19 maternal and neonatal clinical features and pregnancy outcomes up to June 3, 2021

**DOI:** 10.1016/j.xagr.2021.100049

**Published:** 2022-01-03

**Authors:** Greg Marchand, Avinash S. Patil, Ahmed T. Masoud, Kelly Ware, Alexa King, Stacy Ruther, Giovanna Brazil, Nicolas Calteux, Hollie Ulibarri, Julia Parise, Amanda Arroyo, Catherine Coriell, Chelsea Cook, Alexandra Ruuska, Anas Zakarya Nourelden, Katelyn Sainz

**Affiliations:** a Marchand Institute for Minimally Invasive Surgery, Mesa, AZ (Dr Marchand, Mses Ware, King, Ruther, and Brazil, Mr Calteux, Mses Ulibarri, Parise, Arroyo, and Coriell); b Department of Obstetrics and Gynecology, The University of Arizona College of Medicine-Phoenix, Phoenix, AZ (Dr Patil); c Valley Perinatal Services, Phoenix, AZ (Dr Patil); d Faculty of Medicine, Fayoum University, Fayoum, Egypt (Dr Masoud); e Midwestern School of Osteopathic Medicine, Glendale, AZ (Mses Cook and Ruuska); f Faculty of Medicine, Al-Azhar University, Cairo, Egypt (Dr Nourelden); g Department of Pediatrics, Tucson Medical Center, Tucson, AZ (Dr Sainz)

**Keywords:** coronavirus, COVID-19 in pregnancy, COVID-19 pregnancy outcomes, pregnancy outcomes, SARS-CoV-2

## Abstract

**OBJECTIVE:**

COVID-19 is a rapidly changing and developing emergency that requires constant re-evaluation of available data. We report a systematic review and meta-analysis based on all published high-quality data up to and including June 3, 2021 on the maternal and neonatal outcomes in pregnant women infected with COVID-19.

**DATA SOURCES:**

PubMed, SCOPUS, MEDLINE, ClinicalTrials.gov, and Web of Science databases were queried from inception up to June 3, 2021.

**STUDY ELIGIBILITY CRITERIA:**

We included all clinical studies (prospective and retrospective cohort studies, case-control studies, case series, and rapid communications) that reported data on any maternal and neonatal outcomes of pregnant women with COVID-19.

**METHODS:**

The data were analyzed as pooled proportions or odds ratios and 95% confidence intervals in meta-analysis models.

**RESULTS:**

We included 111 studies enrolling 42,754 COVID-19-positive pregnant women. From COVID-19-positive pregnant women, the incidence rates were 53.2% (95% confidence interval, 48–58.4) for cesarean delivery, 41.5% (95% confidence interval, 36.3–46.8) for spontaneous vaginal delivery, and 6.4% (95% confidence interval, 4.5–9.2) for operative delivery. The rates of some adverse neonatal events, including premature delivery (16.7%; 95% confidence interval, 12.8–21.5) and low birthweight (16.7%; 95% confidence interval, 12.8–21.5) were relatively high in mothers infected with COVID-19. Vertical transmission (3.5%; 95% confidence interval, 2.7–4.7), neonatal death (3%; 95% confidence interval, 2–4), stillbirth (1.9%; 95% confidence interval, 1.5–2.4), and maternal mortality (0.012%; 95% confidence interval, 0.010–0.014) were rare adverse events. The mean birthweight was 3069.7 g (95% confidence interval, 3009.7–3129.8 g). In the comparative analysis, COVID-19 significantly increased the risk of premature delivery (odds ratio, 1. 48 [95% confidence interval, 1.22–1.8]), preeclampsia (odds ratio, 1.6 [95% confidence interval, 1.2–2.1]), stillbirth (odds ratio, 2.36 [95% confidence interval, 1.24–4.462]), neonatal mortality (odds ratio, 3.35 [95% confidence interval, 1.07–10.5]), and maternal mortality (odds ratio, 3.08 [95% confidence interval, 1.5–6.3]). The pooled analyses were homogenous, with mild heterogeneity in premature delivery and preeclampsia outcomes.

**CONCLUSION:**

The data must be interpreted with caution as limited data are available, and no complete assessment of bias is possible at this time. Our data suggest that pregnant women who test positive for COVID-19 seem to be at a higher risk of lower birth weights and premature delivery. There is no evidence at this time of the sharply increased maternal mortality that was seen previously with both the 2003 SARS and 2012 MERS pandemics.

**AJOG Global Reports at a Glance**

**Why was this study conducted?**

With the constant evolution of the COVID-19 pandemic, a periodic assessment of the available high-quality evidence is important in making informed decisions regarding the care of pregnant women infected with COVID-19.

**Key findings**

Like in previous systematic reviews, we found an increased risk of premature delivery and cesarean delivery rates in mothers infected with COVID-19. We did not find any evidence of the significant spike in maternal mortality that was seen with both the 2003 SARS and 2012 MERS coronavirus strains.

**What does this add to what is known?**

The large number of studies analyzed add strength to the notion that obstetricians may expect a higher incidence of preterm deliveries in mothers infected with COVID-19. It also adds strength to the consideration of respective changes in treatment plans such as antenatal steroid administration.

## Introduction

The COVID-19 pandemic, which was caused by the 2019 novel coronavirus (2019-nCoV) (first isolated in China in December 2019), has grown to unprecedented proportions in modern times.^1^ Even now, the consequences of infection with COVID-19 in pregnant women are not fully understood. This is largely because of the shortage of sufficient evidence in this regard. Previous published articles, which scrutinized the effects of infection with earlier beta coronaviruses, showed that infected pregnant women were more susceptible to developing sepsis and acute respiratory distress syndrome. This warranted critical admission to the intensive care unit.^2^ Medical literature reveals that all-cause pneumonia has been linked to preterm labor, premature rupture of membranes, fetal growth restriction, and fetal death in addition to neonatal demise.^3^^,^^4^

The most recent large systematic review and meta-analysis on this topic, performed by Matar et al,^5^ concluded that the clinical manifestations of pregnant women who were infected with COVID-19 were similar to nonpregnant individuals who had this disease. Nonetheless, the authors of this study found that pregnant women who had confirmed COVID-19 had higher rates of cesarean deliveries and preterm births than the average reported statistics globally. One of the limitations of this review by Matar et al^5^ and another recent review by Kasraeian et al^6^ was the small sample size of the reported patients with 137 and 86 patients, respectively. A large cohort of studies regarding the impact of COVID-19 infection on pregnant women along with the effects of the virus on the fetus continues to be published. Consequently, we aimed to implement this comprehensive systematic review and meta-analysis to appraise the contemporary literature and dissect the effects of COVID-19 on pregnant women and their babies. We build on the previous literature and have included all the published quality data up to and including a publication date of June 3, 2021, with a total of 111 included studies totaling 42,754 infected pregnant patients.

## Methods

We followed the MOOSE (Meta-analysis of Observational Studies in Epidemiology) statement guidelines during the preparation of this systematic review and meta-analysis.^7^ In addition, the reporting of this study was according to the Preferred Reporting Items for Systematic Reviews and Meta-Analysis (PRISMA) checklist.^8^

### Search strategy and eligibility criteria

The relevant articles were retrieved from 5 major databases (PubMed, SCOPUS, MEDLINE, ClinicalTrials.gov, and Web of Science databases) from December 1, 2019 to June 3, 2021. A comprehensive search was done using the following search strategy: (“COVID-19” OR “SARS-CoV-2”) AND (“maternal outcomes” OR “neonatal outcomes” OR pregnan*). In addition, we performed a manual search of the references of the included articles. Two reviewers independently screened the titles and abstracts of the search results to define the initially eligible studies. Further full-text screening of the initially eligible studies was performed to determine the articles that would be finally included in this meta-analysis. Disagreements were settled by discussion, and the final decision was made by a third reviewer.

### Inclusion and exclusion criteria

We included all prospective and retrospective cohort studies, case series, short communications, and case-control studies that reported data on the clinical characteristics and the maternal and neonatal outcomes of pregnant women with COVID-19. There were no restrictions on time or country of origin. Reviews, single case reports, non-English studies, expert opinions, letters to the editor, and studies without analyzable data were excluded from this study. The authors noted that some studies relative to our analyzed outcomes were excluded because they were published as a single case report or letter to the editor, when in fact, their subject matter could have qualified as a cohort study.

### Data extraction

The extracted data included the first author, year of publication, study design, country, income, sample size, age of pregnant women, and COVID-19 infection confirmation method. Furthermore, we extracted the following outcomes of interest: (1) Maternal coexisting comorbidities including gestational diabetes and preeclampsia (2) Maternal delivery outcomes including either emergency or elective cesarean delivery, spontaneous vaginal delivery, preterm delivery (defined as before 37 weeks’ gestation,) and operative delivery, intensive care unit (ICU) admission, and the maternal mortality rate (3) Neonatal outcomes including low birthweight babies, premature delivery, neonatal birthweight, neonatal intensive care unit (NICU) admission, neonatal death, fetal death or stillbirth, and vertical transmission of SARS-CoV-2 infection. Two different investigators performed the data extraction in parallel to prevent errors. Discrepancies were then resolved by consensus. A third investigator was assigned to decide in the event that any discrepancies could not be resolved by the 2 extracting investigators.

### Risk of bias assessment and strength of evidence

We assessed the quality of the included observational studies according to the quality assessment tools of the National Heart, Lung, and Blood Institute.^9^ We used both the tools of the observational cohort and case-control studies, which are composed of questions assessing the risk of bias and confounders. Each question was answered by “yes,” “no,” “not applicable,” “not reported,” or “cannot determine.” Then each study was given a score to guide the overall quality as either “poor,” “fair,” or “good.” In addition, the strength of evidence was evaluated by the Grading of Recommendations Assessment Development and Evaluation (GRADE) tool.^10^ A summary of the results of our risk of bias assessment can be found in supplemental Tables S1 and S2.

### Statistical analysis

Comprehensive Meta-Analysis software version 3 was used for quantitative synthesis. Dichotomous events and no events were pooled as weighted proportions and odds ratios (OR) with 95% confidence intervals (CI), whereas the pooled rates of proportions were calculated through the Freeman–Tukey transformation meta-analysis of proportions using MedCalc (Version 15.0; MedCalc Software, Ostend, Belgium). For continuous outcomes, we used mean difference with 95% CIs and a random effects meta-analysis model. A *P* value <.05 was considered statistically significant. Heterogeneity among studies was assessed by visual inspection and using the *I*-square (*I*^2^) and chi-squared tests. Chi-square *P* values of <.1 or *I*^2^ >50% were considered as indicators of a significant heterogeneity. When heterogeneity was encountered, we changed from a fixed effect to a random effects model (when possible) to attempt to solve the heterogeneity. We also attempted to solve it by omitting 1 study from the analysis, also referred to as the “leave-on-out” method.

## Results

### Study selections

**Figure 1:**
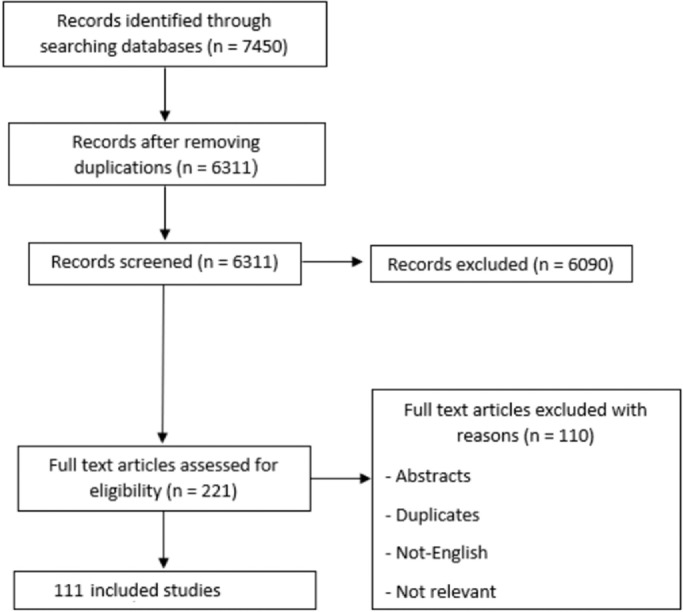
PRISMA flow diagram *PRISMA*, Preferred Reporting Items for Systematic Reviews and Meta-Analysis.

Database searching resulted in 7450 references. After duplicate removal by Endnote X8.0.1 (Build 1044) (Clarivate Analytics, London, United Kingdom), 6311 records were eligible for title and abstract screening. Thus, 221 reports were initially marked as eligible for inclusion. The full-text articles of these reports were examined, and 111 articles were included in the final systematic review and meta-analysis. A complete list of articles is included in (Appendix 1). The flow of data collection and screening process are shown in (Figure 1).

Marchand. COVID-19 outcomes in pregnancy. Am J Obstet Gynecol Glob Rep 2022.

### Baseline characteristics and strength of evidence

The summary and baseline characteristics of the included studies are shown in the (Table). Our systematic review included 111 studies that comprised a total of 42,754 infected pregnant women. The included studies varied in their design as prospective and retrospective cohort studies, case series, and case-control studies.

**Table:** Summary and baseline characteristics of the included studies

Study ID	Study design	Data source	Country	Setting	Income	Sample size	Mean age	COVID-19 confirmed by the following:	Main maternal and neonatal outcomes reported	Control group (n)
Abedzadeh-Kalahroudi 2021	Cohort	Facility-based	Iran	The exposed group: Referral Hospital of Kashan University of Medical Sciences (Shahid Beheshti Hospital). The nonexposed group: Midwifery clinics to receive prenatal care.	Middle-income	150 (56 positive)	31.6 (Exposed Group)	qRT-PCR, or based on clinical manifestations, laboratory findings, and positive findings on CT scan.	C-delivery, preeclampsia, preterm labor, and fetal distress	Not applicable
Ahlberg 2020		Registry-based	Sweden	Karolinska University Hospital, Stockholm	High-income	759 (155 positive)	32.1 (Positive)/ 32.0 (Negative)	RT-PCR	Preeclampsia, breastfeeding at discharge, gestational diabetes, preterm birth, induction of labor, epidural analgesia, mode of delivery, postpartum hemorrhage, 5-min Apgar score, large for gestational age, small for gestational age, major birth defect, and stillbirth	Not applicable
Ajith 2021	Retrospective	Registry-based	India	Tertiary care center (Referral center for 2 northern districts of Kerala)	Low-income	350 COVID-19–positive pregnancies / 223 delivered	NA	Antigen test or RT-PCR	Stillbirth, mode of delivery, breastfeeding and rooming-in, and infected neonates	Not applicable
Anand 2020	Cohort	Facility-based	India	Vardhman Mahavir Medical College & Safdarjung Hospital, New Delhi	Low-income	69	26.7	RT-PCR OR (SARS-CoV 2 specific RdRp (RNA-dependent RNA polymerase) gene or Sarbeco subgenus ORF-1b-nsp14b gene)	Intrauterine death, neonatal infectivity, and viral load	Not applicable
Antoun 2020	Prospective cohort	Facility-based	United Kingdom	University Hospitals of Birmingham	High-income	23	29.3	RT-PCR	Cesarean delivery, vaginal delivery, maternal mortality, preeclampsia, postpartum hemorrhage, preterm birth, ICU, birthweight, 5-min Apgar score <7 and vertical transmission.	Not applicable
Anuk 2021	Prospective case-control	Population-based	Turkey	Ankara City Hospital	Middle-income	70	30 (cases)/ 29 (controls)	RT-PCR	Maternal-fetal Doppler parameters	Not applicable
Bachani 2020	Retrospective	Registry-based	India	Medical college affiliated tertiary care hospital	Low-income	57	26.71	qRT-PCR	Maternal mortality, neonatal infectivity, and disease's severity	Not applicable
Badr 2020	Retrospective case-control	Registry-based	France and Belgium	(1) Antoine Béclère, Clamart, Paris, France; (2) Bicêtre Hospital, Le Kremlin-Bicêtre, France; (3) Centre Hospitalier Sud Francilien, Corbeil-Essonnes, France; and (4) Brugmann University Hospital, Brussels, Belgium.	High-income	83	31.97	RT-PCR	ICU	Not applicable
Barbero 2020	Retrospective Cohort	Registry-based	Spain	Tertiary care center, Hospital Universitario “12 de Octubre,” Madrid	High-income	91	33.15	NP swab or suggestive radiological findings	Pneumonia, hospitalization rate, ICU admission, COVID-19 severe forms, demographic characteristics, pregnancy-related conditions and presenting symptoms, rate of cesarean delivery, preterm birth, and mortality rates.	Not applicable
Blitz 2020	Retrospective (Research Letters)	Registry-based	United States	large hospital system in New York State	High-income	82		RT-PCR	ICU	Not applicable
BRANDT 2020	Case-control	Population-based	United States	Robert Wood Johnson University Hospital, a 139 regional perinatal center in New Brunswick, New Jersey	High-income	183	30.3 for the COVID-19 group, 30.9 for the control group	Quantitative PCR	Adverse maternal outcomes: Preeclampsia, venous thromboembolism, antepartum admission, maternal ICU admission, need for mechanical ventilation, supplemental oxygen, or maternal death. Adverse neonatal outcomes: respiratory distress syndrome, intraventricular hemorrhage, necrotizing enterocolitis, 5-min Apgar score <5, persistent category 2 fetal heart rate tracing despite intrauterine resuscitation, or neonatal death.	Not applicable
Campbell 2020	Retrospective	Registry-based	United States	3 Yale New Haven Health hospitals in southern Connecticut	High-income	30		RT-PCR	Cesarean delivery, preterm birth, vertical transmission, 5-min Apgar score <7, and birthweight	Not applicable
Cheng 2020	Retrospective	Registry-based	China	Renmin Hospital of Wuhan University	Middle-income	31	29	RT-PCR	Neonatal mortality, NICU, preterm birth, maternal mortality, ICU, vertical transmission, fetal Stillbirth, 5-min Apgar score <7, birthweight <2500 g, and birthweight	Not applicable
Cohen 2020	Results of a French national survey	Population-based	France	internet platform	High-income	88	31	RT-PCR, Serology or lung CT-scanner	Cesarean delivery and Gestational diabetes.	Not applicable
Cojocaru 2020	Quality improvement	Facility-based	United States	University of Maryland Medical System	High-income	86	30.4	PCR	Maternal bonding, ICU admission, transmission	Not applicable
Cribiù 2020	Cohort	Facility-based	Spain	Fondazione IRCCS Ca’ Granda — Ospedale Maggiore Policlinico, Milan, and Department of Pathology, University of Basel	High-income	37 (21 positive)	31.3 (cases)/ 35 (controls)	PCR	Mode of delivery, indications for labor induction, and neonatal outcomes	Not applicable
Cruz-Lemini 2021	Prospective	Facility-based	Spain	by the Spanish Obstetric Emergency group in 42 hospitals	High-income	604 (174 positive asymptomatic)	32.6 (cases)/ 33.2 (controls)	PCR	Onset of labor, type of delivery, Preeclampsia, thrombotic risk, perinatal complications, neonatal data, and causes of NICU admission	Not applicable
Di Guardo 2021	Retrospective cohort	Registry-based	Italy	Department of Gynecology and Obstetrics of 2 tertiary referral hospitals	High-income	145	31.5	qRT- PCR	Maternal death, neonatal death, vertical transmission, and preterm birth.	Not applicable
Di Mascio 2020	Retrospective cohort	Registry-based	22 different countries in Europe, United States, South America, Asia and Australia	73 centers		388	32.2	RT-PCR	Maternal mortality and morbidity, including ICU admission, mechanical ventilation use, and death.	Not applicable
Dıaz-Corvillon 2020	Cross-sectional study	Population-based	Chile	Obstetrics & Gynecology Department of Clínica Dávila, Santiago	Middle-income	37	29.9	RT-PCR	Cesarean delivery, Instrumental delivery, neonatal mortality, preterm birth, NICU, fetal stillbirth, birthweight, birthweight <2500 g, 5-min Apgar score <7, and vertical transmission	546
Dumitriu 2020	Retrospective cohort	Registry-based	United States	NewYork–Presbyterian Morgan Stanley Chil- dren's Hospital or NewYork–Presbyterian Allen Hospital	High-income	100	28.8	Cobas or Xpert Xpress PCR (except for 1 –> symptomatic but negative)	Neonatal infectivity, maternal COVID-19 status, and neonatal characteristics and clinical courses	Not applicable
Facchetti 2020	Retrospective	Registry-based	Italy	Brescia Spedali Civili Hospital	High-income	15	35.1	RT-PCR	Induction of labor, neonatal mortality, NICU, preterm birth, fetal Stillbirth, gestational diabetes, 5-min Apgar score <7, birthweight, birthweight <2500 g and vertical transmission.	Not applicable
Farghaly 2020	Retrospective Cohort	Registry-based	United States	Brookdale Hospital Medical Center, New York	High-income	15	33.4	RT-PCR	Cesarean delivery, vaginal delivery, NICU, preterm birth, birthweight and vertical transmission.	64
Flaherman 2020	Prospective cohort	Registry-based	United States	Pregnancy Coronavirus Outcomes Registry (PRIORITY)	High-income	179	31.5	RT-PCR	Vaginal delivery, NICU, ICU, preterm birth, birthweight and vertical transmission.	84
Gale 2020	Prospective cohort	Registry-based	United Kingdom	British Paediatric Surveillance Unit	High-income	66 infected neonates	-	NA	Gestational age at delivery, mode of transmission, and disease's severity	Not applicable
Gaspar 2021	Retrospective	Registry-based	Portugal	Maternity of a Central Hospital in the Center Region	High-income	12	35.58	RT-PCR	Condition's severity, maternal mortality, spontaneous abortions, preterm births, cesarean sections, and vertical transmission	Not applicable
Ghema 2021	Descriptive	Facility-based	Morocco	neonatal ICU of Harouchi Mother and Child Hospital in Casablanca	Low-income	30 neonates	-	PCR	Maternal symptoms, ICU admission, median gestational age at delivery, and neonatal infectivity	Not applicable
Goyal 2020	Prospective observational	Facility-based	India	Department of Obstetrics and Gynecology at All India Institute of Medical Sciences, Jodhpur	Low-income	633 (COVID−19 period)/ 32 (Infected)/ 1116 (pre-COVID−19)	-	RT-PCR	Institutional deliveries, ICU admission, antenatal visits, and maternal and fetal outcomes in COVID positive.	Not applicable
Gulersen 2020	Retrospective Cohort	Registry-based	United States	Long Island Jewish Medical Center, Northwell Health, Queens, New Year	High-income	50	29.3	RT-PCR	Vaginal delivery, gestational diabetes, and birthweight	50
Handley 2020	Cohort	Registry-based	United States	(GeoBirth) From 2 Penn Medicine hospitals, Philadelphia	High-income	8867 (Total)/ 2992 (Pandemic period)/ 86 (Infected)	—	—	Stillbirth, overall preterm birth, spontaneous preterm birth, iatrogenic preterm birth	Not applicable
Hcinia 2021	Prospective cohort	Facility-based	France	Department of Obstetrics and Gynecology of the Centre Hospitalier de L'Ouest Guyanais (referral center of western French Guiana)	High-income	507 (137 positive)	25.7 (positive)/ 26.3 (negative)	PCR	Disease's severity, maternal death, ICU admission and oxygen support (noninvasive ventilation, endotracheal intubation), mode of delivery, preterm delivery, acute fetal distress, postpartum hemorrhage and transfusion, late miscarriages, stillbirth, neonatal death, NICU admission, respiratory distress, seizures, Apgar score 7 at 1 min, umbilical venous lactate ≥5 mmol/L at birth, and low birthweight	Not applicable
He 2020	Retrospective	Registry-based	China	Tongji Hospital affiliated to Huazhong University of Science & Technology, Wuhan	Middle-income	22 neonates	-	Based on the "New Coronavirus Pneumonia Prevention and Control Program 7th Edition."	Neonatal clinical characteristics, routine blood test, liver and kidney functions, and SARS-COV2 antibodies	Not applicable
HuiYang 2020	Retrospective	Registry-based	China	Patients in Wuhan, China	Middle-income	27	29.91	RT-PCR or clinically confirmed	Cesarean delivery, vaginal delivery, neonatal mortality, maternal mortality, gestational diabetes, preeclampsia, preterm birth, birthweight, birthweight <2500 g, neonatal asphyxia and vertical transmission.	Not applicable
Hui Yang 2020	Observation	Registry-based	China	Patients in Wuhan, China	Middle-income	13	30.2	RT-PCR	Cesarean delivery, vaginal delivery, NICU and vertical transmission.	42
Jenabi 2020	Case-control	Population-based	Iran	Hospitals of Hamadan Province	Middle-income	90	29.47 (Symptomatic)/ 28.78 (Asymptomatic)	rRT-PCR	C-section, low birthweight, preterm labor, preeclampsia, hospitalization, and neonatal death	Not applicable
Knight 2020	Prospective cohort	Population-based	United Kingdom	UK Obstetric Surveillance System	High-income	472		RT-PCR	Cesarean delivery, vaginal delivery, neonatal mortality, NICU, preterm birth, maternal mortality, ICU, vertical transmission, fetal Stillbirth and Iatrogenic preterm birth.	Not applicable
Leon-Abarca 2020	Retrospective analysis	Registry-based	Mexico	Patients across Mexico	Middle-income	3434		RT-PCR	ICU	Not applicable
Liu 2020	Retrospective case-control	Registry-based	China	Two centers in China	Middle-income	21	31	RT-PCR	ICU, 5-min Apgar score <7, preterm birth	Not applicable
Llorca 2021	Cohort	Facility-based	Spain	University Hospital Marqués de Valdecilla (HUMV), Santander	High-income	1167 (14 were positive)	34	RT-PCR and ELISA	Mode of delivery, COVID-19 incidence, and pathology in pregnancy	Not applicable
Lokken 2021	Retrospective cohort	Registry-based	United States	22 large hospitals, and 13 clinic systems providing prenatal care in Washington State	High-income	240	28.7	PCR	Disease severity, hospitalization because of COVID-19, ICU admission, maternal mortality, final pregnancy outcome, COVID-19 at final outcome, and recovery	Not applicable
Lopian 2020	Cohort	Facility-based	Israel	Mayanei Hayeshua Medical Center (MHMC) in Bnei Brak	High-income	21	30	RT-PCR	ICU admission, mortality, mode of delivery, Apgar score, and vertical transmission	Not applicable
Lu Zhang 2020	Retrospective observational study	Registry-based	China	Renmin Hospital of Wuhan University	Middle-income	18	29.11	RT-PCR or clinically confirmed	Cesarean delivery, vaginal delivery, gestational diabetes, preeclampsia, birthweight, preterm birth, and vertical transmission.	Not applicable
Luming Xu 2020	Retrospective observational study	Registry-based	China	Wuhan Union Hospital	Middle-income	5	28.8	RT-PCR	Cesarean delivery, vaginal delivery, neonatal mortality, preterm birth, birthweight, birthweight <2500 g, Neonatal Mortality, 5-min Apgar score <7 and vertical transmission.	Not applicable
Mahajan 2021	Retrospective	Registry-based	India	dedicated Covid-19 Hospital in Mumbai	Low-income	879	26.97	PCR	Twinning rate, term deliveries, spontaneous abortions, and hypertensive disorders of pregnancy	Not applicable
Martinez-Perez 2021	Prospective cohort	Facility-based	Spain	Spanish Obstetric Emergency group in 45 hospitals	High-income	246	32.6	RT-PCR	Cesarean delivery, vaginal delivery, Instrumental delivery, neonatal mortality, NICU, preterm birth, gestational diabetes, Postpartum hemorrhage, maternal mortality, ICU, vertical transmission, fetal Stillbirth and 5-min Apgar score <7.	763
Martinez-Portilla	Prospective cohort	Facility-based	Mexico	475 monitoring hospitals dedicated to COVID-19 and located in all 32 states of Mexico	Middle-income	5183 pregnant and 175,998 nonpregnant	28.5 (pregnant)	RT-PCR	Death, pneumonia, intubation, and ICU admission	Not applicable
Maru 2020	Retrospective cross-sectional study	Registry-based	United States	L&D Unit at Elmhurst 106 Hospital	High-income	46	30.2	Cepheid rapid PCR	Cesarean delivery, vaginal delivery, preterm birth, and gestational diabetes	78
Mattar 2020	Prospective cohort	Facility-based	Singapore	The National University Hospital, KK Women's and Children's Hospital (KKH), Singapore General Hospital	High-income	16	29.75	RT-PCR	Severe disease, pregnancy loss, and vertical and horizontal transmission	Not applicable
Mattern 2021	Prospective	Facility-based	France	The Antoine Be ´clère Hospital maternity ward (Paris area, France)	High-income	249 (20 Immunoglobulin G-positive)	32.83 (IgG-positive)	Serology test	Gestational age at delivery, birthweight, and infected neonate	Not applicable
Molina 2020	Retrospective	Registry-based	Spain	Mancha-Centro Hospital in Castile-La Mancha, Spain	High-income	20	34.9	Qualitative serologic positive antibody test and/or RT-PCR	Clinical characteristics, management, treatment, and obstetrical and neonatal outcomes	Not applicable
Moreno 2020	Retrospective	Registry-based	United States	Flushing Hospital Medical Centre or Jamaica Hospital Medical Centre (JHMC)	High-income	19	31.7	rRT-PCR	Vertical transmission of COVID-19	Not applicable
Nambair 2020	Retrospective Cohort	Registry-based	India	Tertiary Referral Center in South India	Low-income	350	NA	RT-PCR	Mode of delivery, postpartum hemorrhage, NICU, infected neonates, and breastfeeding	Not applicable
Nayak 2020	Retrospective	Registry-based	India	Department of Obstetrics and Gynaecology at Ter- tiary Care Hospital attached to a Medical College (Central Mumbai)	Low-income	977 (141 positive)	NA	PCR	Mode of delivery, Apgar score, and vertical transmission	Not applicable
Ochiai 2020	Retrospective	Registry-based	Japan	Tertiary center, Keio University Hospital (in central Tokyo)	High-income	3	32	RT-PCR or clinically confirmed	Cesarean delivery, vaginal delivery, NICU, preterm birth, birthweight, 5-min Apgar score <7, birthweight <2500 g, vertical transmission, and gestational diabetes	Not applicable
Oncel 2020	Cohort	Facility-based	Turkey	34 NICUs in Turkey	Middle-income	125		RT-PCR		Not applicable
Onwuzurike 2020	Retrospective	Registry-based	United States	Department of Obstetrics & Gynecology, Brigham and Women's Hospital, Boston, MA.	High-income	44	29.6	PCR	Disease's severity, hospitalization, indication for delivery, pregnancy and neonatal outcomes, and postpartum care	Not applicable
Ozsurmeli 2021	Retrospective cohort	Registry-based	Turkey	Istanbul Medeniyet University Göztepe Training and Research Hospital and University of Health Sciences Derince Training and Research Hospital	Middle-income	24	26.9	qRT-PCR	Clinical symptoms, mode of delivery, laboratory results, and disease's severity.	Not applicable
Pachtman 2020	Retrospective	Registry-based	United States	Seven hospitals within Northwell Health, New York state	High-income	20		PCR	Pregnancy complications, clinical symptoms, and cardiac enzymes	Not applicable
Patberg 2020	Retrospective cohort	Registry-based	United States	NYU Winthrop Hospital	High-income	133 (77 positive)	29.9 (positive)/ 32.3 (negative)	PCR	Fetal vascular malperfusion abnormalities, mode of delivery, pregnancy complications, and neonatal infection.	Not applicable
Pecks 2020	Retrospective	Registry-based	Germany	121 German hospitals and from Kepler University Hospital Linz, Austria	High-income	247	NA	NA	Outcomes in pregnant women regarding COVID-19, obstetrical pregnancy outcome, mode of delivery, gestational age, and neonatal outcomes	Not applicable
Peng 2020	Retrospective	Registry-based	China	Hubei Province	Middle-income	24	29.8	RT-PCR	Cesarean delivery, neonatal mortality, preterm birth, gestational diabetes, birthweight, and vertical transmission	21
Pereira 2020	Retrospective	Facility-based	Spain	Puerta de Hierro University Hospital Madrid, Spain	High-income	60	34	RT-PCR	Clinical symptoms, disease's severity, mode of delivery, treatment, and lab results	Not applicable
Pierce-Williams 2020	Cohort	Facility-based	United States	12 US institutions	High-income	64	33.2	Laboratory testing meeting criteria for diagnosis of severe or critical COVID-19 as defined by the "Chinese Center for Disease Control and Prevention."	Median duration from hospital admission to discharge, need for supplemental oxygen, intubation, cardiomyopathy, cardiac arrest, death, and timing of delivery.	Not applicable
Pineles 2020	Retrospective Cohort	Registry-based	United States	A community hospital in Houston, Texas	High-income	77		RT-PCR	Cesarean delivery, neonatal mortality, preterm birth, NICU, fetal stillbirth, birthweight, and vertical transmission.	858
Pirjani 2020	Prospective cohort	Facility-based	Iran	Arash Hospital in Tehran, Iran	Middle-income	199 (66 positive)	30.97 (positive)/ 28.79 (negative)	RT-PCR and CT		Not applicable
Prabhu et al,^16^ 2020	Prospective cohort	Facility-based	United States	NewYork Presbyterian-Weill Cornell Medical Center, New York Presbyterian-Lower Manhattan Hospital and New York Presbyterian-Queens, New York	High-income	70	31.24	RT-PCR	Cesarean delivery, vaginal delivery, preterm birth, live birth, ICU admission, gestational diabetes, preeclampsia, vertical transmission, and fetal stillbirth, NICU, birthweight, and severe neonatal asphyxia.	605
Pu Yang 2020	Retrospective	Registry-based	China	Zhongnan Hospital of Wuhan University	Middle-income	7	—	RT-PCR	Cesarean delivery, vaginal delivery, NICU, preterm birth, birthweight, neonatal asphyxia, and vertical transmission.	Not applicable
Qiancheng 2020	Retrospective	Registry-based	China	The Central Hospital of Wuhan	Middle-income	28	30	RT-PCR	Cesarean delivery, vaginal delivery, preterm birth, ICU admission, gestational diabetes, vertical transmission, fetal stillbirth, NICU, birthweight, birthweight <2500 g, severe neonatal asphyxia and Neonatal Mortality.	Not applicable
Qing-Lei Zeng 2020	Retrospective	Registry-based	China	12 centers in Henan and Shaanxi Provinces, China	Middle-income	2	—	RT-PCR	Cesarean delivery, vaginal delivery, neonatal mortality, preterm birth, maternal mortality, vertical transmission	Not applicable
Reale 2020	Prospective cohort	Facility-based	United States	Four large hospitals: 2 academic medi- cal centers and 2 community hospitals	High-income	93	29.6	RT-PCR	Cesarean delivery and gestational diabetes	2852
Ríos-Silva 2020	Retrospective Cohort	Registry-based	Mexico	The open national database of COVID-19 [12] from the Ministry of Health of Mexico.	Middle-income	29	448	RT-PCR	ICU admission and maternal mortality	Not applicable
Rizzo 2021	Prospective case-control	Population-based	Italy	The Division of Maternal Fetal Medicine, Università di Roma Tor Vergata, Italy	High-income	49	30.4	RT-PCR	Birthweight	98
Rong Yang 2020	Retrospective Cohort	Registry-based	China	The Maternal and Child Health Information Management System of Wuhan (MCHIMS)	Middle-income	65	_	RT-PCR	Cesarean delivery, vaginal delivery, gestational diabetes, preeclampsia, preterm birth, birthweight, neonatal asphyxia, and vertical transmission	Not applicable
Sahin 2020	Prospective cohort	Facility-based	Turkey	Turkish Ministry of Health Ankara City Hospital	Middle-income	29	26.38	RT-PCR	Cesarean delivery, vaginal delivery, preeclampsia, preterm delivery, ICU admission, NICU, vertical transmission, and birthweight	8
Sahin 2020 "update"	Prospective cohort	Facility-based	Turkey	Turkish Ministry of Health Ankara City Hospital	Middle-income	533	28.04	RT-PCR	Cesarean delivery, vaginal delivery, preterm delivery, ICU admission, gestational diabetes, preeclampsia, maternal mortality, NICU, vertical transmission, and birthweight	Not applicable
Sakowicz 2020	Retrospective cohort	Registry-based	United States	Northwestern Memorial Hospital or affiliated outpatient clinics	High-income	101	30	PCR	Gestational diabetes.	1317
Salvatore 2020	Observation cohort	Facility-based	United States	New York Presbyterian—Komansky Children's Hospital, Weill Cornell Medicine, New York Presbyterian—Lower Manhattan Hospital, and New York Presbyterian—Queens	High-income	78	_	RT-PCR	Cesarean delivery, vaginal delivery, NICU, preterm delivery, birthweight, birthweight <2500 g, and vertical transmission	Not applicable
Samadi 2021	Cross-sectional study	Population-based	Iran	Forghani Hospital in Qom, a tertiary referral hospital	Middle-income	258	29.5	RT-PCR or lung CT scan or both	Cesarean delivery, vaginal delivery, ICU, maternal mortality, gestational diabetes, and preeclampsia	Not applicable
San-juan 2020	Retrospective cohort	Registry-based	Spain	Department of Obstetrics of the University Hospital “12 de Octubre” (Madrid, Spain)	High-income	32	32	RT-PCR	Cesarean delivery, vaginal delivery, ICU, gestational diabetes, preterm delivery, birthweight, 5-min Apgar score <7, birthweight, and vertical transmission.	Not applicable
Santana 2021	Retrospective cohort	Registry-based	Spain	University Hospital La Paz, Madrid, Spain	High-income	29	31.9	RT-PCR	Cesarean delivery, vaginal delivery, preterm delivery, ICU admission, maternal mortality, gestational diabetes, vertical transmission, 5-min Apgar score <7, and birthweight	Not applicable
Santhosh 2021	Retrospective	Registry-based	Oman	A tertiary care center in Muscat, Oman	High-income	60	32	RT-PCR	Cesarean delivery, vaginal delivery, instrumental delivery, ICU, gestational diabetes, preeclampsia, preterm delivery, birthweight, birthweight <2500 g, fetal stillbirth, vertical transmission, and postpartum hemorrhage	Not applicable
Savasi 2020	Prospective cohort	Prospective cohort	Italy	12 maternity hospitals in Northern Italy including L. Sacco (Milan), Mangi- agalli (Milan), S. Gerardo MBBM Foundation (Mon- za), Papa Giovanni XXIII (Bergamo), and San Matteo (Pavia) as hub maternity hospitals, and Hospitals of Padua, Florence, Lecco, Trento, Modena, Seriate and Piacenza.	High-income	77	32	RT-PCR	Cesarean delivery, vaginal delivery, ICU, NICU, preterm delivery, birthweight, vertical transmission	Not applicable
Savirón-Cornudella 2020	Retrospective cohort	Registry-based	Spain	The Hospital Universitario General de Villalba, located in the North of Madrid	High-income	6	27.83	RT-PCR	Cesarean delivery, vaginal delivery, NICU, birthweight, and vertical transmission.	Not applicable
Savirón-Cornudella 2020	Retrospective cohort	Registry-based	Spain	The Villalba General University Hospital, Madrid and the Miguel Servet University Hospital, Zaragoza, Spain.	High-income	22	29.2	RT-PCR	Cesarean delivery, vaginal delivery, instrumental delivery, NICU, gestational diabetes, postpartum hemorrhage, preterm delivery, birthweight, 5-min Apgar score <7, and vertical transmission.	1189
Schwartz,^13^ 2020	Retrospective cohort	Registry-based	Iran	Ten hospitals in different cities throughout Iran	Middle-income	22 neonates			Cesarean delivery, preterm delivery, birthweight, birthweight <2500 g, and vertical transmission.	Not applicable
Sherer 2020	Retrospective cohort	Registry-based	United States	Johns Hopkins Hospital	High-income	22	27	RT-PCR	Cesarean delivery, vaginal delivery, NICU	11
Shmakov 2020	Prospective observational study	Registry-based	Russia	The National Medical Research Center for Obstetrics, Gynecology and Perinatology, Ministry of Healthcare of Russia Federation	Middle-income	66	30.3	RT-PCR	Cesarean delivery, vaginal delivery, instrumental delivery, ICU, preterm delivery, birthweight, vertical transmission, and maternal mortality	Not applicable
Singh 2021	Observational study	Registry-based	India	Tata Main Hospital, Jamshedpur, a tertiary care hospital in Eastern India	Low-income	132	27.5	RT-PCR	Cesarean delivery, vaginal delivery, instrumental delivery, postpartum hemorrhage, NICU, preterm delivery, birthweight, fetal stillbirth, neonatal mortality, and vertical transmission.	Not applicable
Smithgall 2020	Retrospective	Registry-based	United States	Academic hospital, Columbia University Irving Medical Center in New York City	High-income	51	32.3	RT-PCR	Cesarean delivery, vaginal delivery, neonatal mortality, preterm birth, 5-min Apgar score <7, and vertical transmission.	25
Soffer 2021	Retrospective cohort	Registry-based	United States	Large academic medical center serving patients from multiple communities	High-income	67	31	RT-PCR	ICU and gestational diabetes	Not applicable
Soto-Torres 2020	Retrospective case-control	Registry-based	United States	The Maternal-Fetal Medicine Division of the University of Texas McGovern Medical School Department of Obstetrics and Gynecology	High-income	106	28	RT-PCR	Cesarean delivery, vaginal delivery, preeclampsia, NICU, preterm delivery, fetal stillbirth, and birthweight	103
Suyuthi 2020	Descriptive study	Registry-based	Indonesia	Dr Wahidin Sudirohusodo Hospital	Middle-income	26	_	RT-PCR	Cesarean delivery, vaginal delivery, maternal mortality, gestational diabetes, preeclampsia, neonatal mortality, neonatal asphyxia, vertical transmission	Not applicable
Tug 2020	Retrospective study	Registry-based	Turkey	Four tertiary centers (Şehit Prof Dr İlhan Varank Training and Research Hospital, İstanbul; Kartal Dr Lütfi Kırdar Training and Research Hospital, İstanbul; Darıca Farabi Training and Research Hospital, Kocaeli; Medeniyet University Hospital, İstanbul)	Middle-income	188	31	RT-PCR (8 confirmed with imaging studies only)	Cesarean delivery, vaginal delivery, ICU, preterm delivery, gestational diabetes, preeclampsia, and vertical transmission.	Not applicable
Villalaın 2020	Retrospective cohort	Registry-based	Spain	Hospital Universitario 12 de Octubre, a large teaching hospital in the south of Madrid	High-income	673	32	RT-PCR	Cesarean delivery, vaginal delivery, NICU, fetal stillbirth, gestational diabetes, preterm delivery, birthweight, 5-min Apgar score <7, and vertical transmission	Not applicable
Vintzileos 2020	Retrospective cohort	Registry-based	United States	The NYU Winthrop Hospital of the NYU Langone Health System;	High-income	32	31	RT-PCR	Vertical transmission	Not applicable
Vivanti 2020	Retrospective	Registry-based	France	Four tertiary referral obstetrical units in the Paris metropolitan area included in the study were Antoine Béclère, Clamart; Bicêtre Hospital, Le Kremlin Bicêtre; Louis-Mourier, Colombes; and Centre Hospitalier SudFrancilien, Evry	High-income	100	33.1	RT-PCR (1 confirmed with imaging studies only)	Cesarean delivery, vaginal delivery, induction of labor, premature delivery, neonatal mortality, maternal mortality, preeclampsia, ICU, fetal death/stillbirth, NICU, and vertical transmission.	Not applicable
Vizheh 2021	Retrospective cohort	Registry-based	Iran	Three hospitals—Arash, Imam Khomeini, and Shariati	Middle-income	110	32.02	RT-PCR	Cesarean delivery, vaginal delivery, preterm delivery, neonatal mortality, maternal mortality, ICU, gestational diabetes, preeclampsia, NICU, birthweight, and vertical transmission.	Not applicable
Wang 2020	Retrospective	Registry-based	China	The Central Hospital of Wuhan, China	High-income	30	29.9	RT-PCR or imaging studies	Cesarean delivery and vaginal delivery.	Not applicable
Wang 2020	Retrospective cohort	Registry-based	United States	Boston Medical Center	High-income	53	29.8	RT-PCR	Cesarean delivery, vaginal delivery, preeclampsia, induction of labor, and preterm birth	760
Wei 2020	Retrospective		China	Tongji Hospital, Wuhan, China	Middle-income	17	33.3	RT-PCR	ICU and maternal mortality.	Not applicable
Wei Liu 2020	Retrospective	Registry-based	China	Tongji Hospital and HuangShi Maternal and Child Healthcare Hospital	Middle-income	15	32	RT-PCR	Cesarean delivery, vaginal delivery, gestational diabetes, postpartum hemorrhage, NICU, preterm birth, birthweight, and vertical transmission.	16
Wu 2020	Retrospective	Registry-based	China	Renmin Hospital, Wuhan Uni- versity, and Central Hospital of Wuhan, Tongji Medical College, Huazhong University of Science and Technology	Middle-income	29	29.59	RT-PCR or chest CT scan	Cesarean delivery, vaginal delivery, preterm birth, NICU, gestational diabetes, and postpartum hemorrhage	Not applicable
Xu 2020	Retrospective observational study	Registry-based	China	The west campus of Union hospital	Middle-income	34	30	RT-PCR	Cesarean delivery, vaginal delivery, gestational diabetes, preeclampsia, neonatal mortality, fetal stillbirth, NICU, severe neonatal asphyxia, and vertical transmission.	Not applicable
Yan 2020	Retrospective	Registry-based	China	25 hospitals in China	Middle-income	116	30.8	RT-PCR or clinically confirmed	Cesarean delivery, vaginal delivery, gestational diabetes, preeclampsia, neonatal mortality, fetal stillbirth, preterm birth, birthweight, neonatal mortality, maternal mortality, NICU, ICU, neonatal asphyxia, and vertical transmission.	Not applicable
Yao 2021	Retrospective cohort	Registry-based	United States	Department of Obstetrics and Gynecology, Loma Linda University	High-income	50	28.86	RT-PCR	Preterm birth	Not applicable
Yazihan 2020	Prospective case-control	Population-based	Turkey	Ankara City Hospital	Middle-income	95	29	RT-PCR	Preterm birth, gestational diabetes and preeclampsia.	92
Yin 2020	Retrospective cohort	Registry-based	China	Wuhan Union and Tongji hospitals of Huazhong University of Science and Technology.	Middle-income	31	31	RT-PCR	Cesarean delivery, vaginal delivery, neonatal mortality, preterm birth, vertical transmission, fetal stillbirth, 5-min Apgar score <7, birthweight <2500 g, and birthweight	Not applicable
Yingchun Zeng 2020	Retrospective cohort	Registry-based	China	Wuhan Union Hospital	Middle-income	14	31	RT-PCR	Cesarean delivery, vaginal delivery, preterm birth, maternal mortality, vertical transmission	Not applicable
Yu 2020	Retrospective descriptive	Registry-based	China	Tongji hospital	Middle-income	7	31.75	RT-PCR	Cesarean delivery, vaginal delivery, neonatal mortality, fetal stillbirth, preterm birth, birthweight, maternal mortality, vertical transmission	Not applicable
Zambrano 2020	Report	Population-based	United States	Women across the United States	High-income	409,462 (23,434 infected pregnant & 386,028 nonpregnant)	NA	Laboratory-confirmed	Signs and symptoms of COVID-19, ICU admission, and death	Not applicable
Zou 2020	Retrospective analysis	Registry-based	China	Tongji Hospital in Wuhan	Middle-income	6	31	RT-PCR	Cesarean delivery, 5-min Apgar score <7, neonatal mortality, maternal mortality, vertical transmission	Not applicable

*CT*, computerized tomography; *ICU*, intensive care unit; *IgG*, immunoglobulin G; *NICU*, neonatal intensive care unit; *NYU*, New York University; *OR*, odds ratio; *qRT-PCR*, real time quantitative reverse transcription-polymerase chain reaction; *RT-PCR*, reverse transcription-polymerase chain reaction.

Regarding the cohort and population-based studies, 43 studies were of poor quality, 41 were of fair quality, and the other 19 were of good quality, according to the National Institutes of Health (NIH) quality assessment tool for observational cohort studies. However, according to the NIH quality assessment tool for observational case-control studies, only 1 study was of poor quality, 3 were of fair quality, and the other 4 were of good quality. As for publication bias, most of our constructed funnel plots were asymmetrical, and the further Egger tests were significant (Appendix 2). However, the outcomes of spontaneous vaginal delivery and cesarean delivery were symmetrical with no small-study effects. Supplemental Table S1 shows the detailed risk of bias assessment for cohort studies, whereas Supplemental Table S2 shows detailed risk of bias for case-control studies.

### Maternal outcomes

**Figure 2:**
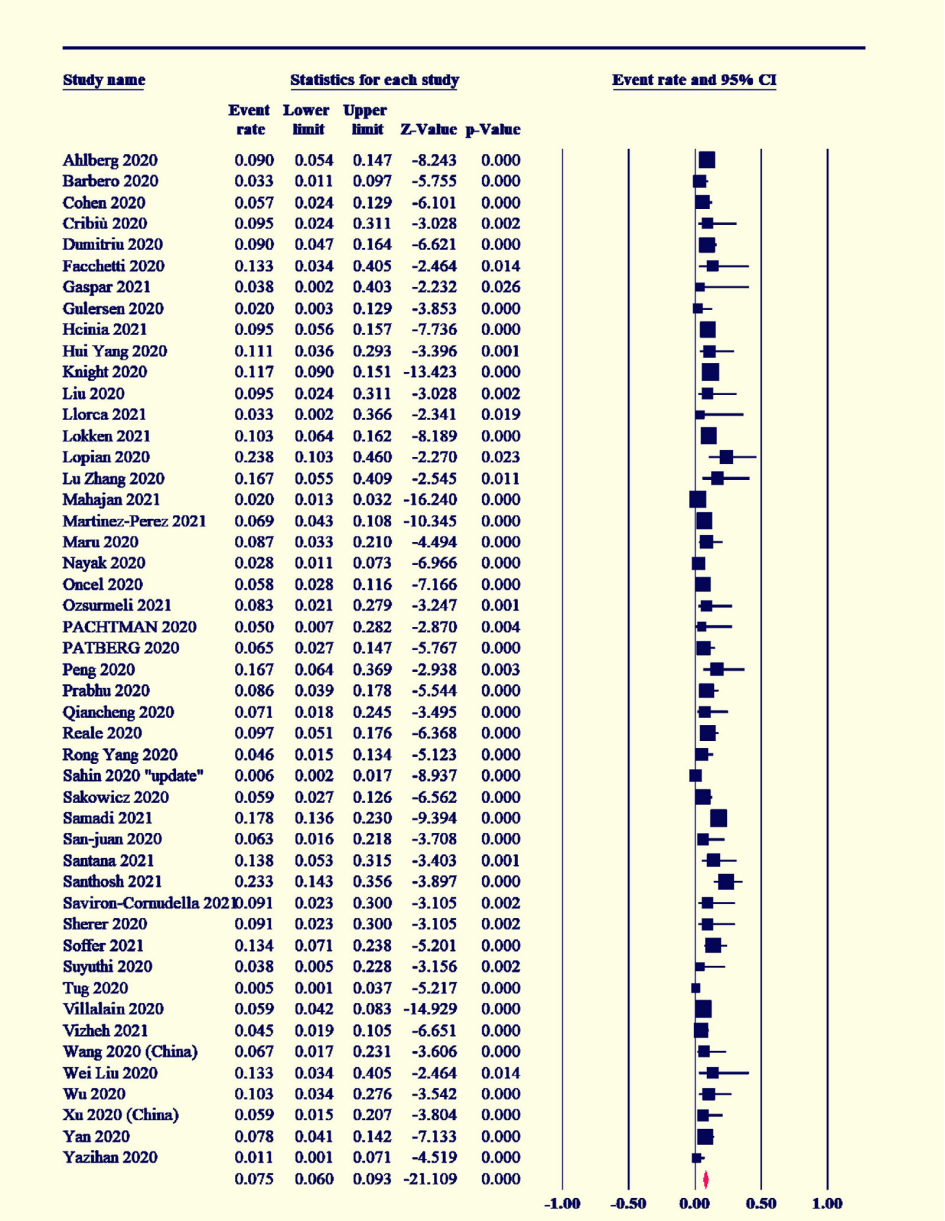
Forest plot of event rate with 95% CI for gestational diabetes mellitus *CI*, confidence interval.

**Figure 3:**
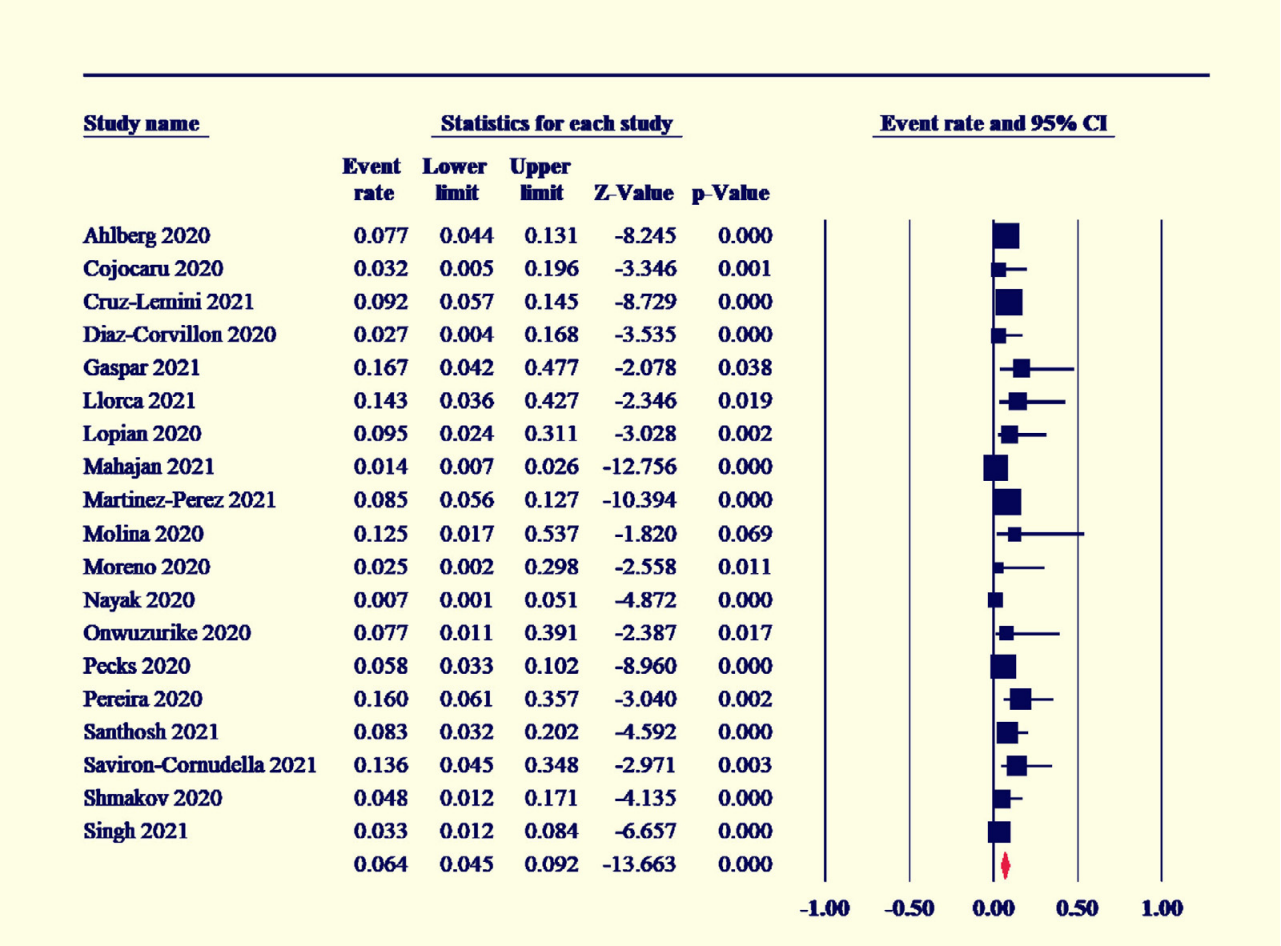
Forest plot of event rate with 95% CI for preeclampsia *CI*, confidence interval.

**Figure 4:**
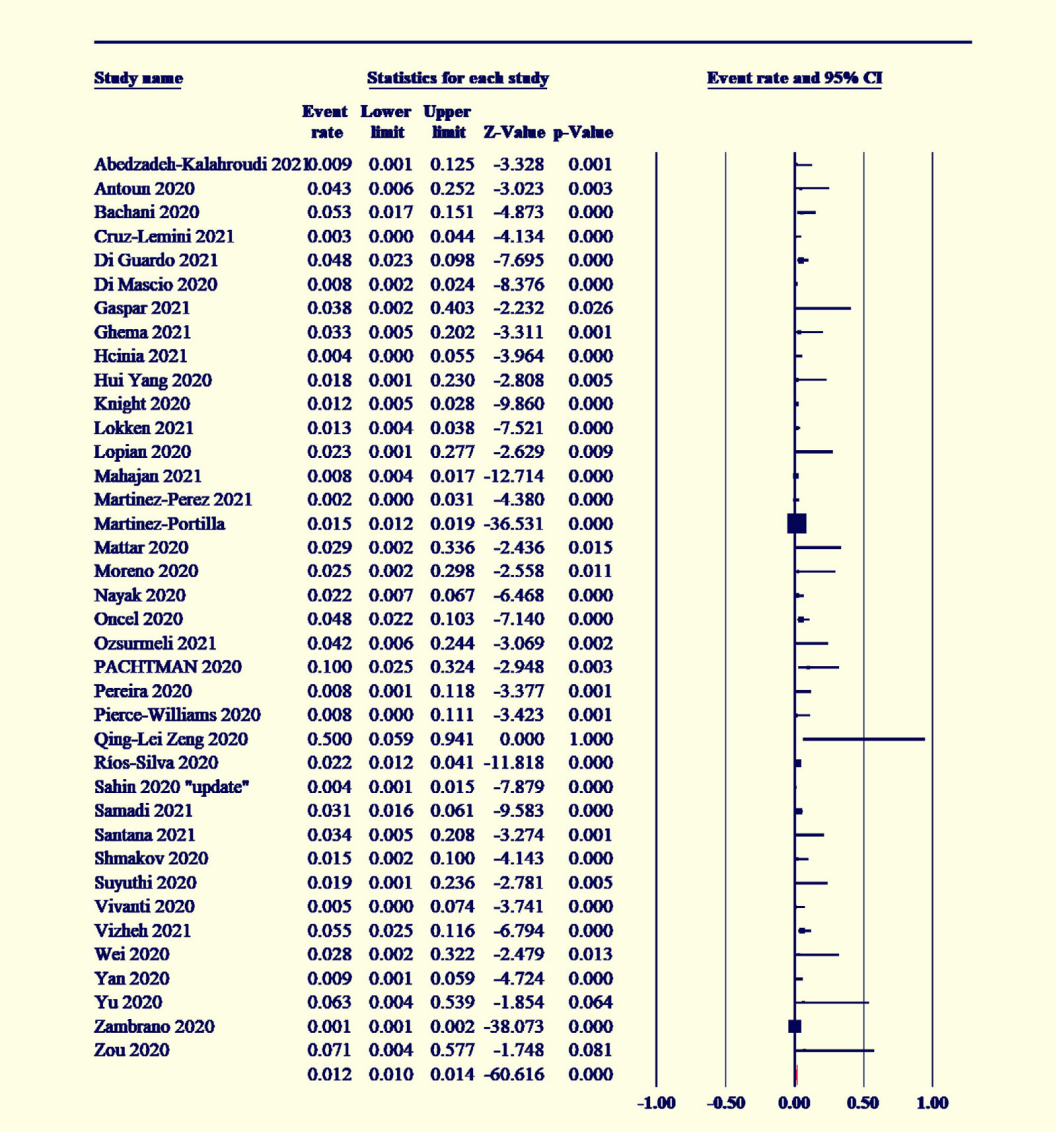
Forest plot of event rate with 95% CI for the maternal mortality rate *CI*, confidence interval.

**Figure 5:**
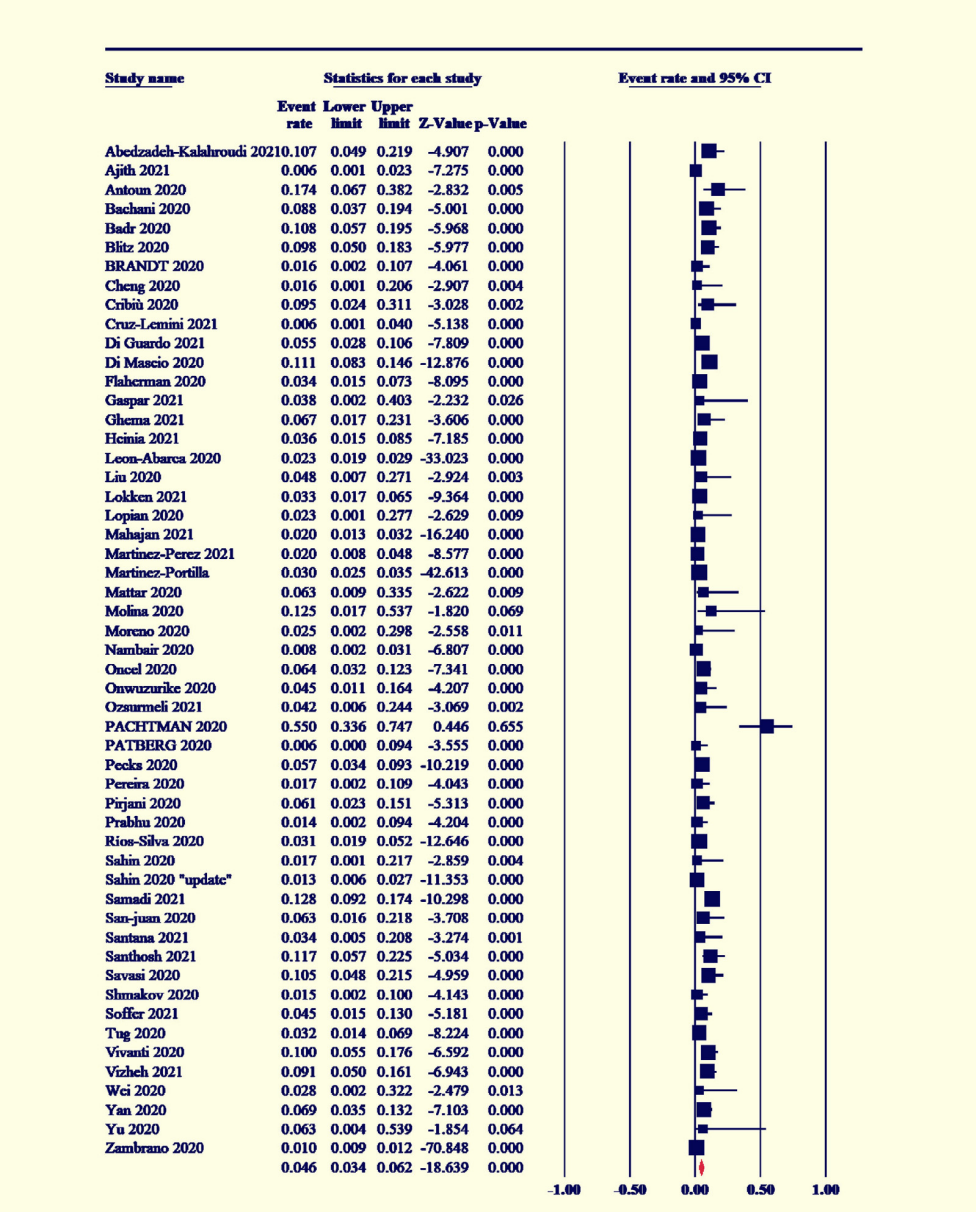
Forest plot of event rate with 95% CI for the maternal ICU admission rate *CI*, confidence interval; *ICU*, intensive care unit.

Among the COVID-19-positive women, 7.5% had gestational diabetes (95% CI [6–9.3]; *I*^2^˃50%) (Figure 2), and preeclampsia existed in 7% (95% CI [5.5–8.9]; *I*^2^˃50%) (Figure 3). The maternal mortality rate was 1.2% (95% CI [1–1.4]; *I*^2^<50%) (Figure 4), and 4.6% of COVID-19-positive women were admitted to the ICU (95% CI [3.4–6.2]; *I*^2^<50%) (Figure 5).

Marchand. COVID-19 outcomes in pregnancy. Am J Obstet Gynecol Glob Rep 2022.

Marchand. COVID-19 outcomes in pregnancy. Am J Obstet Gynecol Glob Rep 2022.

Marchand. COVID-19 outcomes in pregnancy. Am J Obstet Gynecol Glob Rep 2022.

Marchand. COVID-19 outcomes in pregnancy. Am J Obstet Gynecol Glob Rep 2022.

### Delivery outcomes

Among the COVID-19-positive pregnant women, 53.2% had an emergency, indicated, or elective cesarean delivery (95% CI [48–58.4]; *I*^2^<50%) (Supplement Figure S1); 41.5% had spontaneous vaginal delivery (95% CI [36.3–46.8]; *I*^2^<50%) (Supplement Figure S2); and 6.4% of them had an operative vaginal delivery (95% CI [4.5–9.2]; *I*^2^<50%) (Supplement Figure S3).

### Neonatal outcomes

The overall pooled proportion for low birthweight in the babies of COVID-19-positive women was 16.7% (95% CI [12.8–21.5]; *I*^2^˃50 %) (Supplement Figure S4). The premature delivery rate was estimated to be 20% (95% CI [17.1–23.3]; *I*^2^˃50%) (Supplement Figure S5). The pooled mean difference of the neonatal birthweight was 3069.7 g (95% CI [3009.7–3129.8 g]; *I*^2^<50%) (Supplement Figure S6). Almost 32.9% of the delivered neonates needed NICU admission (95% CI [17.6–31.6]; *I*^2^˃50%) (Supplement Figure S7). The neonatal death rate was 3.0% (95% CI [2–4]; *I*^2^˃50%) (Supplement Figure S8), and 1.9% experienced fetal death or stillbirth (95% CI [1.5–2.4]; *I*^2^˃50%) (Supplement Figure S9). The overall vertical transmission rate of SARS-CoV-2 infection was 3.5% (95% CI [2.7–4.7]; *I*^2^˃50%) (Supplement Figure S10).

### Comparative analysis with associated odds ratios

We implemented further comparative analysis between positive and negative COVID-19 patients to assess the possible associated risks. Pooled ORs were not statistically significant in most reported outcomes (Appendix 3). However, COVID-19 significantly increased the risk of premature delivery (OR, 1.48 [95% CI, 1.22–1.8]; *I*^2^=23.99%), preeclampsia (OR, 1.6; 95% CI, 1.2–2.1); *I*^2^=30.98), stillbirth (OR, 2.36 [95% CI, 1.24–4.462]; *I*^2^=5.54%), neonatal mortality (OR, 3.35; 95% CI, 1.07–10.5]; *I*^2^=0%), and maternal mortality (OR, 3.08 [95% CI, 1.5–6.3]; *I*^2^=0%). The pooled analyses were homogenous, with mild heterogeneity in the premature delivery and preeclampsia outcomes.

### Sensitivity and subgroups analyses

We performed further sensitivity and consequent subgroups analyses according to the quality of included studies to confirm the robustness of our analysis (Appendix 4). These analyses revealed no significant difference in most of our reported outcomes when considering all the quality variations. However, the premature delivery rate was relatively higher in poor quality articles (20% [95% CI, 14.9–26.8]), whereas the frequency of stillbirth events was notably higher in good quality articles (3.6% [95% CI, 1.1–11.8]). Furthermore, the reported vertical transmission rates were lower in good quality articles (26% [95% CI, 0.015–0.046]).

## Discussion

In this systematic review and meta-analysis, we pooled all available data from the literature to provide high-quality evidence regarding the clinical characteristics and the outcomes of pregnancy and delivery in COVID-19-positive pregnant women. Our analysis showed that 7.5% of pregnant women with COVID-19 had gestational diabetes, 7% had preeclampsia, and 4.6% needed ICU admission. A high rate of cesarean delivery (53.2%) was observed among pregnant women with COVID-19, with another 6.4% requiring an operative vaginal delivery. Li et al^11^ hypothesized that this rise in cesarean delivery deliveries is because of regulatory modifications to cope up with the pandemic. Furthermore, several reports showed that many pregnant cases with COVID-19 were indicated for emergency cesarean delivery because of maternal causes such as premature rupture of membrane and worsening respiratory status in patients with severe disease.^12^^,^^13^ Maternal mortality was low (1.2%), according to the pooled data from the included studies. This is in stark contrast to the mortality rates reported in previous coronavirus infections such as the Middle East respiratory syndrome and severe acute respiratory syndrome.^14^ Pooled data demonstrated that premature delivery accounted for approximately 20% of the total deliveries in pregnant women with COVID-19. Fetal death or stillbirth was relatively low (1.9%), and of the delivered neonates, 32.9% needed NICU admission. The authors recognize with great seriousness that these numbers represent significant increases of serious morbid complications (stillbirth, maternal death, neonatal death) over the general population but stand by the positive aspect that these numbers are relatively low in consideration of the subset of third trimester pregnancies with serious respiratory disease. In addition, the authors plan future analyses with subgroup calculation to differentiate studies by socioeconomic status of the country of origin. Our initial delving in this analysis has not shown any of these incidences to be significantly related to the originating country's socioeconomic factors.

Vertical transmission is a major concern in the setting of a global pandemic. Most of the included studies screened newborns for SARS-CoV-2 infection using nasopharyngeal swabs and reverse transcription-polymerase chain reaction (RT-PCR), and most studies performed testing at 24 hours of life. Vertical transmission was reported in 3.5%. Few studies currently include data as to whether these RT-PCR results correlate with clinical symptoms of disease in the newborn later, so this cannot not be explored as an outcome at this time. Therefore, the authors feel that the question of vertical transmission is still largely unanswered, though the low rate of SARS-CoV-2 detection by RT-PCR in newborns is reassuring.

Khoury et al^15^ studied the differences in the maternal and neonatal outcomes between initially symptomatic and asymptomatic pregnant women. Interestingly, the symptomatic patients had a higher risk of cesarean delivery and preterm birth than asymptomatic COVID-19-positive patients. This may give clinicians cause to alter some treatment plans, and in some circumstances, it may decrease the threshold for the administration of antenatal steroids secondary to the higher rates of preterm delivery in COVID-19 infected women. Prabhu et al^16^ showed that initially symptomatic women developed more postpartum fever than asymptomatic COVID-19-positive patients. Previous reports attributed this increased postpartum fever to a cytokine storm in response to the SARS-CoV-2 viral infection.^17^^,^^18^

Our results were similar to recently published systematic reviews and meta-analyses. Islam et al^19^ in 2020 reported that 66.38% of pregnant women had a cesarean delivery and 33.62% had a vaginal delivery. Di Toro et al^20^ in 2021 found that the rate of maternal ICU admission was 8%, that of preeclampsia was 7%, and that of preterm birth was 23%. However, the rate of cesarean delivery was slightly different, as they found that 85% of women underwent cesarean delivery.

Our study has several strengths. We executed a comprehensive systematic review and meta-analysis to investigate and describe the pregnancy outcomes among pregnant individuals infected with COVID-19. We reported as many outcomes as possible pertaining to the maternal clinical features and the fetal or neonatal outcomes among COVID-19-positive pregnant women. Methodologically, the MOOSE and PRISMA guidelines were followed throughout the steps of this study to ensure high-quality reporting. Nonetheless, few caveats warrant attention while interpreting the results of this meta-analysis. The observational nature of the included studies (most retrospective) is an important limitation. Most of the included studies were of moderate quality, and in most cases, the heterogeneity could not be resolved. Another concern is that most of the included pregnant women were in the third trimester, so the results of this meta-analysis cannot be generalized to pregnant women in the first and second trimesters. Lastly, there is an international hurry to publish COVID-19 studies, some of which may unfortunately affect the quality and reliability of the data. Unfortunately, the inclusion of such studies affects the quality and scientific evidence synthesized during the conduct of systematic review and meta-analysis reports. We hope that our comprehensive approach in this report provides a robust summary for practicing obstetricians making evidence-based clinical decisions when caring for pregnant women with COVID-19.

### Conclusion

Pregnant women with COVID-19 are at a significantly higher risk of cesarean delivery and premature delivery than uninfected pregnant women. Given the fact that these results are based on observational studies, further well-designed investigations are warranted to guide an evidence-based clinical practice. Being more vulnerable to unfavorable maternal and neonatal complications, clinicians may consider altering treatment plans to prepare for possible morbidities, most notably the consideration of steroids for the increased possibility of preterm delivery in COVID-19 infected women. Fortunately, despite these findings, there is still no evidence at this time of the sharply increased maternal mortality that was seen previously with both the 2003 SARS and 2012 MERS pandemics.

## Appendix Supplementary materials

Image, application 1

Image, application 2

Image, application 3

Image, application 4

Image, application 5

Image, image 6

Image, image 7

Image, image 8

Image, image 9

Image, image 10

Image, image 11

Image, image 12

Image, image 13

Image, image 14

## Footnotes

Supplementary material associated with this article can be found in the online version at doi:10.1016/j.xagr.2021.100049.
