# Supplementary material for: Systematic review and meta-analysis of COVID-19 maternal and neonatal clinical features and pregnancy outcomes up to June 3, 2021
Source: AJOG Glob Rep. 2022 Jan 3;2(1):100049. doi: 10.1016/j.xagr.2021.100049 (PMC8720679; doi:10.1016/j.xagr.2021.100049)
Supplement: Supplementary file 1 [file mmc1.docx]

Appendix 1: List of included studies.

1. Abedzadeh-Kalahroudi M, Sehat M, Vahedpour Z, Talebian P. Maternal and neonatal outcomes of pregnant patients with COVID-19: A prospective cohort study. Int J Gynecol Obstet. 2021;153(3):449–56.

2. Ahlberg M, Neovius M, Saltvedt S, Söderling J, Pettersson K, Brandkvist C, et al. Association of SARS-CoV-2 Test Status and Pregnancy Outcomes. JAMA - J Am Med Assoc. 2020;324(17):1782–5.

3. Ajith S, Reshmi VP, Nambiar S, Naser A, Athulya B. Prevalence and Risk Factors of Neonatal Covid-19 Infection: A Single-Centre Observational Study. J Obstet Gynecol India [Internet]. 2021;(0123456789):21–4. Available from: https://doi.org/10.1007/s13224-021-01436-7

4. Anand P, Yadav A, Debata P, Bachani S, Gupta N, Gera R. Clinical profile, viral load, management and outcome of neonates born to COVID 19 positive mothers: a tertiary care centre experience from India. Eur J Pediatr. 2021;180(2):547–59.

5. Antoun L, El Taweel N, Ahmed I, Patni S, Honest H. Maternal COVID-19 infection, clinical characteristics, pregnancy, and neonatal outcome: A prospective cohort study. European Journal of Obstetrics & Gynecology and Reproductive Biology. 2020 Sep 1;252:559-62.

6. Anuk AT, Tanacan A, Yetiskin FDY, Buyuk GN, Senel SA, Keskin HL, et al. Doppler assessment of the fetus in pregnant women recovered from COVID-19. J Obstet Gynaecol Res. 2021;47(5):1757–62.

7. Bachani S, Arora R, Dabral A, Marwah S, Anand P, Reddy KS, et al. Clinical Profile, Viral Load, Maternal-Fetal Outcomes of Pregnancy With COVID-19: 4-Week Retrospective, Tertiary Care Single-Centre Descriptive Study. J Obstet Gynaecol Canada [Internet]. 2021;43(4):474–82. Available from: https://doi.org/10.1016/j.jogc.2020.09.021

8. Badr DA, Mattern J, Carlin A, Cordier AG, Maillart E, El Hachem L, et al. Are clinical outcomes worse for pregnant women at ≥20 weeks’ gestation infected with coronavirus disease 2019? A multicenter case-control study with propensity score matching. Am J Obstet Gynecol. 2020;223(5):764–8.

9. Barbero P, Mugüerza L, Herraiz I, García Burguillo A, San Juan R, Forcén L, et al. SARS-CoV-2 in pregnancy: characteristics and outcomes of hospitalized and non-hospitalized women due to COVID-19. J Matern Neonatal Med [Internet]. 2020;0(0):1–7. Available from: https://doi.org/10.1080/14767058.2020.1793320

10. Blasco Santana L, Miraval Wong E, Álvarez-Troncoso J, Sánchez García L, Bartha JL, Regojo-Zapata RM. Maternal and perinatal outcomes and placental pathologic examination of 29 SARS-CoV-2 infected patients in the third trimester of gestation. J Obstet Gynaecol Res. 2021;1–9.

11. Brandt JS, Hill J, Reddy A, Schuster M, Patrick HS, Rosen T, et al. Epidemiology of coronavirus disease 2019 in pregnancy: risk factors and associations with adverse maternal and neonatal outcomes. Am J Obstet Gynecol. 2021;224(4):389.e1-389.e9.

12. Cheng B, Jiang T, Zhang L, Hu R, Tian J, Jiang Y, et al. Clinical characteristics of pregnant women with coronavirus disease 2019 in Wuhan, China. Open Forum Infect Dis. 2020;7(8):1–9.

13. Cohen J, Vignaux O, Jacquemard F. Covid-19 in pregnant women: General data from a French National Survey. Vol. 251, European Journal of Obstetrics and Gynecology and Reproductive Biology. 2020. p. 267–8.

14. Cojocaru L, Crimmins S, Sundararajan S, Goetzinger K, Elsamadicy E, Lankford A, et al. An initiative to evaluate the safety of maternal bonding in patients with SARS-CoV-2 infection. J Matern Neonatal Med [Internet]. 2020;0(0):1–7. Available from: https://doi.org/10.1080/14767058.2020.1828335

15. Cribiù FM, Erra R, Pugni L, Rubio-Perez C, Alonso L, Simonetti S, et al. Severe SARS-CoV-2 placenta infection can impact neonatal outcome in the absence of vertical transmission. J Clin Invest. 2021;131(6).

16. Cruz-Lemini M, Ferriols Perez E, de la Cruz Conty ML, Caño Aguilar A, Encinas Pardilla MB, Prats Rodríguez P, et al. Obstetric Outcomes of SARS-CoV-2 Infection in Asymptomatic Pregnant Women. Viruses. 2021;13(1):1–12.

17. Di Guardo F, Di Grazia FM, Di Gregorio LM, Zambrotta E, Carrara G, Gulino FA, et al. Poor maternal–neonatal outcomes in pregnant patients with confirmed SARS-Cov-2 infection: analysis of 145 cases. Arch Gynecol Obstet [Internet]. 2021;303(6):1483–8. Available from: https://doi.org/10.1007/s00404-020-05909-4

18. Díaz-Corvillón P, Mönckeberg M, Barros A, Illanes SE, Soldati A, Nien JK, et al. Routine screening for SARS CoV-2 in unselected pregnant women at delivery. Vol. 15, PLoS ONE. 2020.

19. Dumitriu D, Emeruwa UN, Hanft E, Liao G V., Ludwig E, Walzer L, et al. Outcomes of Neonates Born to Mothers with Severe Acute Respiratory Syndrome Coronavirus 2 Infection at a Large Medical Center in New York City. JAMA Pediatr. 2021;175(2):157–67.

20. Facchetti F, Bugatti M, Drera E, Tripodo C, Sartori E, Cancila V, et al. SARS-CoV2 vertical transmission with adverse effects on the newborn revealed through integrated immunohistochemical, electron microscopy and molecular analyses of Placenta. EBioMedicine. 2020;59.

21. Farghaly MAA, Kupferman F, Castillo F, Kim RM. Characteristics of Newborns Born to SARS-CoV-2-Positive Mothers: A Retrospective Cohort Study. Am J Perinatol. 2020;37(13):1310–6.

22. Flaherman VJ, Afshar Y, Boscardin WJ, Keller RL, H Mardy A, Prahl MK, et al. Infant Outcomes Following Maternal Infection With Severe Acute Respiratory Syndrome Coronavirus 2 (SARS-CoV-2): First Report From the Pregnancy Coronavirus Outcomes Registry (PRIORITY) Study. Clin Infect Dis. 2020;2(March):1–4.

23. Gale C, Quigley MA, Placzek A, Knight M, Ladhani S, Draper ES, et al. Characteristics and outcomes of neonatal SARS-CoV-2 infection in the UK: a prospective national cohort study using active surveillance. Lancet Child Adolesc Heal [Internet]. 2021;5(2):113–21. Available from: http://dx.doi.org/10.1016/S2352-4642(20)30342-4

24. Gaspar, de Vasconcelos Andreia, and Santos, Isabel Silva. "SARS-CoV2 in Pregnancy—The First Wave." Medicina 57.3 (2021): 241.

25. Gharagozloo, Mahshid, et al. "Mental health and marital satisfaction changes of pregnant and lactating women during the COVID-19 pandemic: A comparative cross-sectional study." world 17 (2021): 18.

26. Ghema K, Lehlimi M, Toumi H, Badre A, Chemsi M, Habzi A, et al. Outcomes of newborns to mothers with COVID-19. Infect Dis Now [Internet]. 2021;(xxxx). Available from: https://doi.org/10.1016/j.idnow.2021.03.003

27. Goyal M, Singh P, Singh K, Shekhar S, Agrawal N, Misra S. The effect of the COVID-19 pandemic on maternal health due to delay in seeking health care: Experience from a tertiary center. Int J Gynecol Obstet. 2021;152(2):231–5.

28. Gulersen M, Prasannan L, Tam Tam H, Metz CN, Rochelson B, Meirowitz N, et al. Histopathologic evaluation of placentas after diagnosis of maternal severe acute respiratory syndrome coronavirus 2 infection. Am J Obstet Gynecol MFM. 2020;2(4):100211.

29. Handley SC, Mullin AM, Elovitz MA, Gerson KD, Montoya-Williams D, Lorch SA, et al. Changes in Preterm Birth Phenotypes and Stillbirth at 2 Philadelphia Hospitals during the SARS-CoV-2 Pandemic, March-June 2020. JAMA - J Am Med Assoc. 2021;325(1):87–9.

30. Hcini N, Maamri F, Picone O, Carod JF, Lambert V, Mathieu M, et al. Maternal, fetal and neonatal outcomes of large series of SARS-CoV-2 positive pregnancies in peripartum period: A single-center prospective comparative study. Eur J Obstet Gynecol Reprod Biol. 2021;257:11–8.

31. He Z, Fang Y, Zuo Q, Huang X, Lei Y, Ren X, et al. Vertical transmission and kidney damage in newborns whose mothers had coronavirus disease 2019 during pregnancy. Int J Antimicrob Agents [Internet]. 2021;57(2):106260. Available from: https://doi.org/10.1016/j.ijantimicag.2020.106260

32. Hui Yang MD, Hu B, Zhan S, Yang LY, Xiong G. Effects of Severe Acute Respiratory Syndrome Coronavirus 2 Infection on Pregnant Women and Their Infants - A Retrospective Study in Wuhan, China. Archives of pathology & laboratory medicine. 2020 Oct 1;144(10):1217-22.

33. Jenabi E, Bashirian S, Khazaei S, Masoumi SZ, Ghelichkhani S, Goodarzi F, et al. Pregnancy outcomes among symptomatic and asymptomatic women infected with COVID-19 in the west of Iran: a case-control study. J Matern Neonatal Med [Internet]. 2020;0(0):1–3. Available from: https://doi.org/10.1080/14767058.2020.1861599

34. Knight M, Bunch K, Vousden N, Morris E, Simpson N, Gale C, et al. Characteristics and outcomes of pregnant women admitted to hospital with confirmed SARS-CoV-2 infection in UK: National population based cohort study. BMJ. 2020;369.

35. Leon-Abarca JA, Pena-Gallardo MT, Soliz J, Accinelli RA. Clinical evolution of COVID-19 during pregnancy at different altitudes: a population-based study (preprint). medRxiv. 2020;2020.09.14.20193177.

36. Liu F, Liu H, Hou L, Li J, Zheng H, Chi R, et al. Clinico-radiological features and outcomes in pregnant women with COVID-19 pneumonia compared with age-matched non-pregnant women. Infect Drug Resist. 2020;13:2845–54.

37. Liu W, Cheng H, Wang J, Ding L, Zhou Z, Liu S, et al. Clinical Analysis of Neonates Born to Mothers with or without COVID-19: A Retrospective Analysis of 48 Cases from Two Neonatal Intensive Care Units in Hubei Province. Am J Perinatol. 2020;37(13):1317–23.

38. Llorca J, Lechosa-Muñiz C, Gortazar P, Fernández-Ortiz M, Jubete Y, Cabero MJ. COVID-19 in a cohort of pregnant women and their descendants, the MOACC-19 study. BMJ Open. 2021;11(2):9–11.

39. Lokken EM, Huebner EM, Taylor GG, Hendrickson S, Vanderhoeven J, Kachikis A, et al. Disease severity, pregnancy outcomes, and maternal deaths among pregnant patients with severe acute respiratory syndrome coronavirus 2 infection in Washington State. Am J Obstet Gynecol [Internet]. 2021;(Cdc):1–14. Available from: https://doi.org/10.1016/j.ajog.2020.12.1221

40. Lopian M, Kashani-Ligumsky L, Czeiger S, Cohen R, Schindler Y, Lubin D, et al. Safety of vaginal delivery in women infected with COVID-19. Pediatr Neonatol [Internet]. 2021;62(1):90–6. Available from: https://doi.org/10.1016/j.pedneo.2020.10.010

41. Mahajan NN, Ansari M, Gaikwad C, Jadhav P, Tirkey D, Pophalkar MP, et al. Impact of SARS-CoV-2 on multiple gestation pregnancy. Int J Gynecol Obstet. 2021;152(2):220–5.

42. Martinez-Perez O, Prats Rodriguez P, Muner Hernandez M, Encinas Pardilla MB, Perez Perez N, Vila Hernandez MR, et al. The association between SARS-CoV-2 infection and preterm delivery: a prospective study with a multivariable analysis. BMC Pregnancy Childbirth. 2021;21(1):1–11.

43. Martinez-Portilla RJ, Sotiriadis A, Chatzakis C, Torres-Torres J, Espino y Sosa S, Sandoval-Mandujano K, et al. Pregnant women with SARS-CoV-2 infection are at higher risk of death and pneumonia: propensity score matched analysis of a nationwide prospective cohort (COV19Mx). Ultrasound Obstet Gynecol. 2021;57(2):224–31.

44. Maru S, Patil U, Carroll-Bennett R, Baum A, Bohn-Hemmerdinger T, Ditchik A, et al. Universal screening for SARS-CoV-2 infection among pregnant women at Elmhurst Hospital Center, Queens, New York. PLoS One. 2020;15(12 December).

45. Mattar CN, Kalimuddin S, Sadarangani SP, Tagore S, Thain S, Thoon KC, et al. Pregnancy Outcomes in COVID-19: A Prospective Cohort Study in Singapore. Ann Acad Med Singapore. 2020;49(11):857–69.

46. Mattern J, Vauloup-Fellous C, Zakaria H, Benachi A, Carrara J, Letourneau A, et al. Post lockdown COVID-19 seroprevalence and circulation at the time of delivery, France. PLoS One. 2020;15(10 October):1–8.

47. Molina EO, Pailos RH, Guillen MP, Pedreno AP, Rodriguez ER, Martinez AH. Covid-19 Infection in Symptomatic Pregnant Women at the Midpoint of the Pandemic in Spain: A Retrospective Analysis. Ginekol Pol. 2020;91(12):755–63.

48. Moreno SC, To J, Chun H, Ngai IM. Vertical Transmission of COVID-19 to the Neonate. Infect Dis Obstet Gynecol. 2020;2020.

49. Nambiar SS, Ajith S, Reshmi VP. Assessing disease outcome in COVID-19 pregnancies in a tertiary referral center in south India: A single-center retrospective cohort study. J SAFOG. 2020;12(5):335–9.

50. Nayak AH, Kapote DS, Fonseca M, Chavan N, Mayekar R, Sarmalkar M, et al. Impact of the Coronavirus Infection in Pregnancy: A Preliminary Study of 141 Patients. J Obstet Gynecol India [Internet]. 2020;70(4):256–61. Available from: https://doi.org/10.1007/s13224-020-01335-3

51. Ochiai D, Kasuga Y, Iida M, Ikenoue S, Tanaka M. Universal screening for SARS-CoV-2 in asymptomatic obstetric patients in Tokyo, Japan. Int J Gynecol Obstet. 2020;150(2):268–9.

52. Oncel MY, Akın IM, Kanburoglu MK, Tayman C, Coskun S, Narter F, Er I, Oncan TG, Memisoglu A, Cetinkaya M, Oguz D. A multicenter study on epidemiological and clinical characteristics of 125 newborns born to women infected with COVID-19 by Turkish Neonatal Society. European journal of pediatrics. 2021 Mar;180(3):733-42.

53. Onwuzurike C, Diouf K, Meadows AR, Nour NM. Racial and ethnic disparities in severity of COVID-19 disease in pregnancy in the United States. Int J Gynecol Obstet. 2020;151(2):293–5.

54. Pachtman Shetty SL, Meirowitz N, Blitz MJ, Gadomski T, Weinberg CR. Myocardial injury associated with coronavirus disease 2019 in pregnancy. Am J Obstet Gynecol [Internet]. 2021;224(2):229–32. Available from: https://doi.org/10.1016/j.ajog.2020.10.014

55. Patberg ET, Adams T, Rekawek P, Vahanian SA, Akerman M, Hernandez A, et al. Coronavirus disease 2019 infection and placental histopathology in women delivering at term. Am J Obstet Gynecol. 2021;224(4):382.e1-382.e18.

56. Pecks U, Kuschel B, Mense L, Oppelt P, Rüdiger M. Pregnancy and SARS-CoV-2 infection in Germany—the CRONOS registry. Dtsch Arztebl Int. 2020;117(49):841–2.

57. Peng S, Zhu H, Yang L, Cao L, Huang X, Dynes M, et al. A study of breastfeeding practices, SARS-CoV-2 and its antibodies in the breast milk of mothers confirmed with COVID-19. Lancet Reg Heal - West Pacific. 2020;4.

58. Pereira A, Cruz-Melguizo S, Adrien M, Fuentes L, Marin E, Perez-Medina T. Clinical course of coronavirus disease-2019 in pregnancy. Acta Obstet Gynecol Scand. 2020;99(7):839–47.

59. Pierce-Williams RAM, Burd J, Felder L, Khoury R, Bernstein PS, Avila K, et al. Clinical course of severe and critical coronavirus disease 2019 in hospitalized pregnancies: a United States cohort study. Am J Obstet Gynecol MFM. 2020;2(3):100134.

60. Pineles BL, Alamo IC, Farooq N, Green J, Blackwell SC, Sibai BM, et al. Racial-ethnic disparities and pregnancy outcomes in SARS-CoV-2 infection in a universally-tested cohort in Houston, Texas. Vol. 254, European Journal of Obstetrics and Gynecology and Reproductive Biology. 2020. p. 329–30.

61. Pirjani R, Hosseini R, Soori T, Rabiei M, Hosseini L, Abiri A, et al. Maternal and neonatal outcomes in COVID-19 infected pregnancies: A prospective cohort study. J Travel Med. 2020;27(7):1–7.

62. Prabhu M, Cagino K, Matthews KC, Friedlander RL, Glynn SM, Kubiak JM, et al. Pregnancy and postpartum outcomes in a universally tested population for SARS-CoV-2 in New York City: a prospective cohort study. BJOG An Int J Obstet Gynaecol. 2020;127(12):1548–56.

63. Qiancheng X, Jian S, Lingling P, Lei H, Xiaogan J, Weihua L, et al. Coronavirus disease 2019 in pregnancy. Int J Infect Dis [Internet]. 2020;95:376–83. Available from: https://doi.org/10.1016/j.ijid.2020.04.065

64. Quing-Lei Z, Song N, Cui GL, Genomic Epidemiology of SARS-CoV-2 From Mainland China With Newly Obtained Genomes From Henan Province. Frontiers in microbiology. 2021;12.

65. Reale SC, Lumbreras-Marquez MI, King CH, Burns SL, Fields KG, Diouf K, et al. Patient characteristics associated with SARS-CoV-2 infection in parturients admitted for labour and delivery in Massachusetts during the spring 2020 surge: A prospective cohort study. Paediatr Perinat Epidemiol. 2021;35(1):24–33.

66. Ríos-Silva M, Murillo-Zamora E, Mendoza-Cano O, Trujillo X, Huerta M. COVID-19 mortality among pregnant women in Mexico: A retrospective cohort study. J Glob Health. 2020;10(2).

67. Rizzo G, Mappa I, Maqina P, Bitsadze V, Khizroeva J, Makatsarya A, et al. Effect of SARS‐CoV‐2 infection during the second half of pregnancy on fetal growth and hemodynamics: A prospective study. Acta Obstet Gynecol Scand. 2021;100(6):1034–9.

68. Saccone G, Sen C, Di Mascio D, Galindo A, Grünebaum A, Yoshimatsu J, et al. Maternal and perinatal outcomes of pregnant women with SARS-CoV-2 infection. Ultrasound Obstet Gynecol. 2021;57(2):232–41.

69. Sahin D, Tanacan A, Erol SA, Anuk AT, Eyi EGY, Ozgu- Erdinc AS, et al. A pandemic center’s experience of managing pregnant women with COVID-19 infection in Turkey: A prospective cohort study. Int J Gynecol Obstet. 2020;151(1):74–82.

70. Sahin D, Tanacan A, Erol SA, Anuk AT, Yetiskin FDY, Keskin HL, et al. Updated experience of a tertiary pandemic center on 533 pregnant women with COVID-19 infection: A prospective cohort study from Turkey. Int J Gynecol Obstet. 2021;152(3):328–34.

71. Sakowicz A, Ayala AE, Ukeje CC, Witting CS, Grobman WA, Miller ES. Risk factors for severe acute respiratory syndrome coronavirus 2 infection in pregnant women. Am J Obstet Gynecol MFM. 2020;2(4):100198.

72. Salvatore CM, Han JY, Acker KP, Tiwari P, Jin J, Brandler M, et al. Neonatal management and outcomes during the COVID-19 pandemic: an observation cohort study. Lancet Child Adolesc Heal [Internet]. 2020;4(10):721–7. Available from: http://dx.doi.org/10.1016/S2352-4642(20)30235-2

73. Samadi P, Alipour Z, Ghaedrahmati M, Ahangari R. The severity of COVID-19 among pregnant women and the risk of adverse maternal outcomes. Int J Gynecol Obstet. 2021;(April):1–8.

74. San-Juan R, Barbero P, Fernández-Ruiz M, López-Medrano F, Lizasoáin M, Hernández-Jiménez P, et al. Incidence and clinical profiles of COVID-19 pneumonia in pregnant women: A single-centre cohort study from Spain. EClinicalMedicine. 2020;23.

75. Santhosh J, Al Salmani M, Khamis F, Ali Al Ubaidani S, Al-Zakwani I. Clinical characteristics of COVID-19 in pregnant women: A retrospective descriptive single-center study from a tertiary hospital in Muscat, Oman. Int J Gynecol Obstet. 2021;152(2):270–4.

76. Savasi VM, Parisi F, Patanè L, Ferrazzi E, Frigerio L, Pellegrino A, et al. Clinical findings and disease severity in hospitalized pregnant women with coronavirus disease 2019 (COVID-19). Obstet Gynecol. 2020;136(2):252–8.

77. Savirón-Cornudella R, Villalba A, Esteban LM, Tajada M, Rodríguez-Solanilla B, Andeyro-Garcia M, et al. Screening of severe acute respiratory syndrome coronavirus-2 infection during labor and delivery using polymerase chain reaction and immunoglobulin testing. Life Sci. 2021;271(December 2020).

78. Savirón-Cornudella R, Villalba A, Zapardiel J, Andeyro-Garcia M, Esteban LM, Pérez-López FR. Severe acute respiratory syndrome coronavirus 2 (SARS-CoV-2) universal screening in gravids during labor and delivery. Eur J Obstet Gynecol Reprod Biol. 2021;256:400–4.

79. Schwartz DA, Mohagheghi P, Beigi B, Zafaranloo N, Moshfegh F, Yazdani A. Spectrum of neonatal COVID-19 in Iran: 19 infants with SARS-CoV-2 perinatal infections with varying test results, clinical findings and outcomes. J Matern Neonatal Med [Internet]. 2020;0(0):1–10. Available from: https://doi.org/10.1080/14767058.2020.1797672

80. Sherer ML, Lei J, Creisher PS, Jang M, Reddy R, Voegtline K, et al. Pregnancy alters interleukin-1 beta expression and antiviral antibody responses during severe acute respiratory syndrome coronavirus 2 infection. Am J Obstet Gynecol. 2021;1–14.

81. Shmakov RG, Prikhodko A, Polushkina E, Shmakova E, Pyregov A, Bychenko V, et al. Clinical course of novel COVID-19 infection in pregnant women. J Matern Neonatal Med [Internet]. 2020;0(0):1–7. Available from: https://doi.org/10.1080/14767058.2020.1850683

82. Singh V, Choudhary A, Datta MR, Ray A. Maternal and Neonatal Outcomes of COVID-19 in Pregnancy: A Single-Centre Observational Study. Cureus. 2021;13(November 2020).

83. Smithgall MC, Liu-Jarin X, Hamele-Bena D, Cimic A, Mourad M, Debelenko L, et al. Third-trimester placentas of severe acute respiratory syndrome coronavirus 2 (SARS-CoV-2)-positive women: histomorphology, including viral immunohistochemistry and in-situ hybridization. Histopathology. 2020;77(6):994–9.

84. Soffer MD, Shook LL, James K, Sawyer MR, Ciaranello A, Mahrouk R, et al. Protocol-Driven Intensive Outpatient Management of Pregnant Patients with Symptomatic Coronavirus Disease 2019. Open Forum Infect Dis. 2020;7(11):1–7.

85. Soto‐Torres E, Andrade EH, Huntley E, Mendez‐Figueroa H, Blackwell SC. Ultrasound and Doppler findings in pregnant SARS‐CoV ‐2 positive women . Ultrasound in Obstetrics & Gynecology. 2021.

86. Sutton D, Fuchs K, D’Alton M, Goffman D. Universal Screening for SARS-CoV-2 in Women Admitted for Delivery. N Engl J Med. 2020;382(22):2163–4.

87. Suyuthi FP, Chalid MT, Padjalangi AN, Djaharuddin I, Massi MN. Maternal and perinatal outcome on pregnancy with COVID-19 infection at Dr. Wahidin Sudirohusodo Hospital Makassar during the period of April-July 2020. Indian J Forensic Med Toxicol. 2020;14(4):7519–22.

88. Tug N, Yassa M, Köle E, Sakin Ö, Çakır Köle M, Karateke A, et al. Pregnancy worsens the morbidity of COVID-19 and this effect becomes more prominent as pregnancy advances. J Turkish Soc Obstet Gynecol. 2020;17(3):149–54.

89. Villalaín C, Herraiz I, Luczkowiak J, Pérez-Rivilla A, Folgueira MD, Mejía I, et al. Seroprevalence analysis of SARS-CoV-2 in pregnant women along the first pandemic outbreak and perinatal outcome. PLoS One. 2020;15(11 November 2020):1–12.

90. Vivanti AJ, Mattern J, Vauloup-Fellous C, Jani J, Rigonnot L, Hachem L El, et al. Retrospective description of pregnant women infected with severe acute respiratory syndrome coronavirus 2, France. Emerg Infect Dis. 2020;26(9):2069–76.

91. Vizheh M, Muhidin S, Aghajani F, Maleki Z, Bagheri F, Hosamirudsari H, et al. Characteristics and outcomes of COVID-19 pneumonia in pregnancy compared with infected nonpregnant women. Int J Gynecol Obstet. 2021;153(3):462–8.

92. Wang MJ, Schapero M, Iverson R, Yarrington CD. Obstetric Hemorrhage Risk Associated with Novel COVID-19 Diagnosis from a Single-Institution Cohort in the United States. Am J Perinatol. 2020;37(14):1411–6.

93. Wang Z, Wang Z, Xiong G. Clinical characteristics and laboratory results of pregnant women with COVID-19 in Wuhan, China. Int J Gynecol Obstet. 2020;150(3):312–7.

94. Wei L, Gao X, Chen S, Zeng W, Wu J, Lin X, et al. Clinical characteristics and outcomes of childbearing-age women with COVID-19 in Wuhan: Retrospective, single-center study. J Med Internet Res. 2020;22(8):1–12.

95. Westgren M, Acharya G. Intensive care unit admissions for pregnant and nonpregnant women with coronavirus disease 2019. Am J Obstet Gynecol. 2020;223(5):779–80.

96. Wu YT, Liu J, Xu JJ, Chen YF, Yang W, Chen Y, et al. Neonatal outcome in 29 pregnant women with COVID-19: A retrospective study in Wuhan, China. PLoS Med [Internet]. 2020;17(7):1–19. Available from: http://dx.doi.org/10.1371/journal.pmed.1003195

97. Xu L, Yang Q, Shi H, Lei S, Liu X, Zhu Y, et al. Clinical presentations and outcomes of SARS-CoV-2 infected pneumonia in pregnant women and health status of their neonates. Sci Bull [Internet]. 2020;65(18):1537–42. Available from: https://doi.org/10.1016/j.scib.2020.04.040

98. Xu S, Shao F, Bao B, Ma X, Xu Z, You J, et al. Clinical manifestation and neonatal outcomes of pregnant patients with coronavirus disease 2019 pneumonia in Wuhan, China. Open Forum Infect Dis. 2020;7(7):1–7.

99. Yan J, Guo J, Fan C, Juan J, Yu X, Li J, et al. Coronavirus disease 2019 in pregnant women: a report based on 116 cases. Am J Obstet Gynecol [Internet]. 2020/04/23. 2020 Jul;223(1):111.e1-111.e14. Available from: https://pubmed.ncbi.nlm.nih.gov/32335053

100. Yang H, Sun G, Tang F, Peng M, Gao Y, Peng J, et al. Clinical features and outcomes of pregnant women suspected of coronavirus disease 2019. J Infect [Internet]. 2020;81(1):e40–4. Available from: https://doi.org/10.1016/j.jinf.2020.04.003

101. Yang P, Wang X, Liu P, Wei C, He B, Zheng J, et al. Clinical characteristics and risk assessment of newborns born to mothers with COVID-19. J Clin Virol [Internet]. 2020;127(April):104356. Available from: https://doi.org/10.1016/j.jcv.2020.104356

102. Yang R, Mei H, Zheng T, Fu Q, Zhang Y, Buka S, et al. Pregnant women with COVID-19 and risk of adverse birth outcomes and maternal-fetal vertical transmission: a population-based cohort study in Wuhan, China. BMC Med. 2020;18(1):1–7.

103. Yao R, Martin CB, Haase VS, Tse BC, Nishino M, Gheorghe C, et al. Initial clinical characteristics of gravid severe acute respiratory syndrome coronavirus 2–positive patients and the risk of progression to severe coronavirus disease 2019. Am J Obstet Gynecol MFM. 2021;3(4):100365.

104. Yazihan N, Tanacan A, Erol SA, Anuk AT, Sinaci S, Biriken D, et al. Comparison of VEGF-A values between pregnant women with COVID-19 and healthy pregnancies and its association with composite adverse outcomes. J Med Virol. 2021;93(4):2204–9.

105. Yin M-Z, Zhang L, Deng G-T, Han C-F, Shen M-X, Sun H-Y, et al. Severe Acute Respiratory Syndrome Coronavirus 2 (SARS-CoV-2) Infection During Pregnancy In China: A Retrospective Cohort Study. 2020;2.

106. Yu N, Li W, Kang Q, Xiong Z, Wang S, Lin X, et al. Clinical features and obstetric and neonatal outcomes of pregnant patients with COVID-19 in Wuhan, China: a retrospective, single-centre, descriptive study. Lancet Infect Dis [Internet]. 2020/03/24. 2020 May;20(5):559–64. Available from: https://pubmed.ncbi.nlm.nih.gov/32220284

107. Zambrano LD, Ellington S, Strid P, Galang RR, Oduyebo T, Tong VT, et al. Update: Characteristics of Symptomatic Women of Reproductive Age with Laboratory-Confirmed SARS-CoV-2 Infection by Pregnancy Status — United States, January 22–October 3, 2020. MMWR Morb Mortal Wkly Rep. 2020;69(44):1641–7.

108. Zeng QL, Li GM, Ji F, Ma SH, Zhang GF, Xu JH, et al. Clinical course and treatment efficacy of COVID-19 near Hubei Province, China: A multicentre, retrospective study. Transbound Emerg Dis. 2020;67(6):2971–82.

109. Zeng Y, Lin L, Yan Q, Wei W, Xiang Yang B, Huang R, et al. Update on clinical outcomes of women with COVID-19 during pregnancy. Int J Gynecol Obstet. 2020;150(2):264–6.

110. Zhang L, Dong L, Ming L, Wei M, Li J, Hu R, et al. Severe Acute Respiratory Syndrome Coronavirus 2(SARS-CoV-2) infection during late pregnancy: A Report of 18 patients from Wuhan, China. 2020;3:1–7.

111. Zou K, Chen H, Liu Y. Patients With COVID-19 Undergoing Cesarean Deliveries: Adapting the OR Suite and Perioperative Care to Prevent Transmission. AORN J. 2020;112(3):217–24.
