# Supplementary material for: Systematic review and meta-analysis of COVID-19 maternal and neonatal clinical features and pregnancy outcomes up to June 3, 2021
Source: AJOG Glob Rep. 2022 Jan 3;2(1):100049. doi: 10.1016/j.xagr.2021.100049 (PMC8720679; doi:10.1016/j.xagr.2021.100049)
Supplement: Supplementary file 2 [file mmc2.pdf]

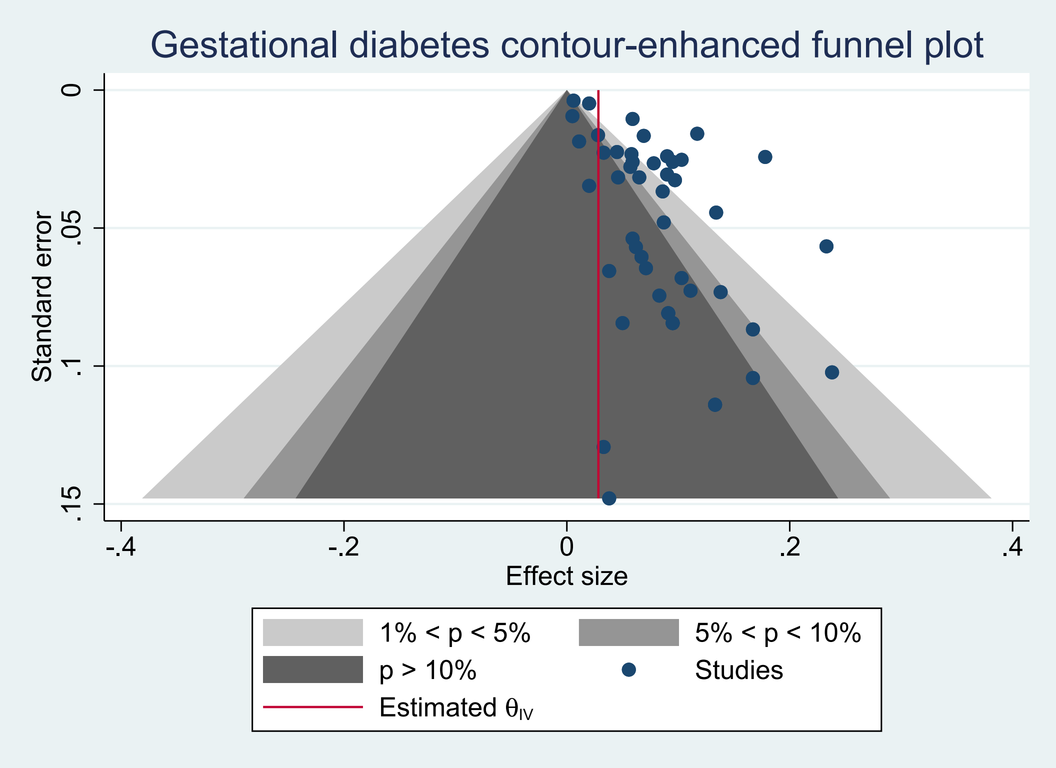

Gestational diabetes contour-enhanced funnel plot
Standard error
0
.05
.1
.15
-.4
-.2
0
.2
.4
Effect size
1% < p < 5%
5% < p < 10%
p > 10%
Studies
Estimated θIV

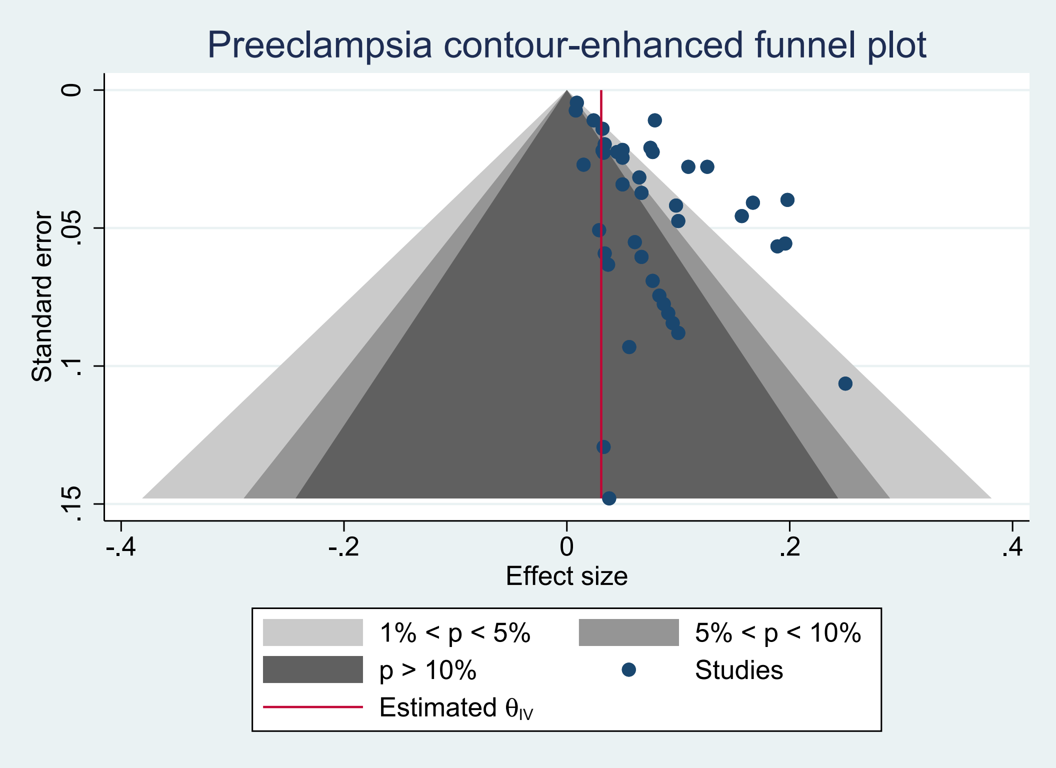

Preeclampsia contour-enhanced funnel plot
Standard error
0
.05
.1
.15
-.4
-.2
0
.2
.4
Effect size
1% < p < 5%
5% < p < 10%
p > 10%
Studies
Estimated θIV

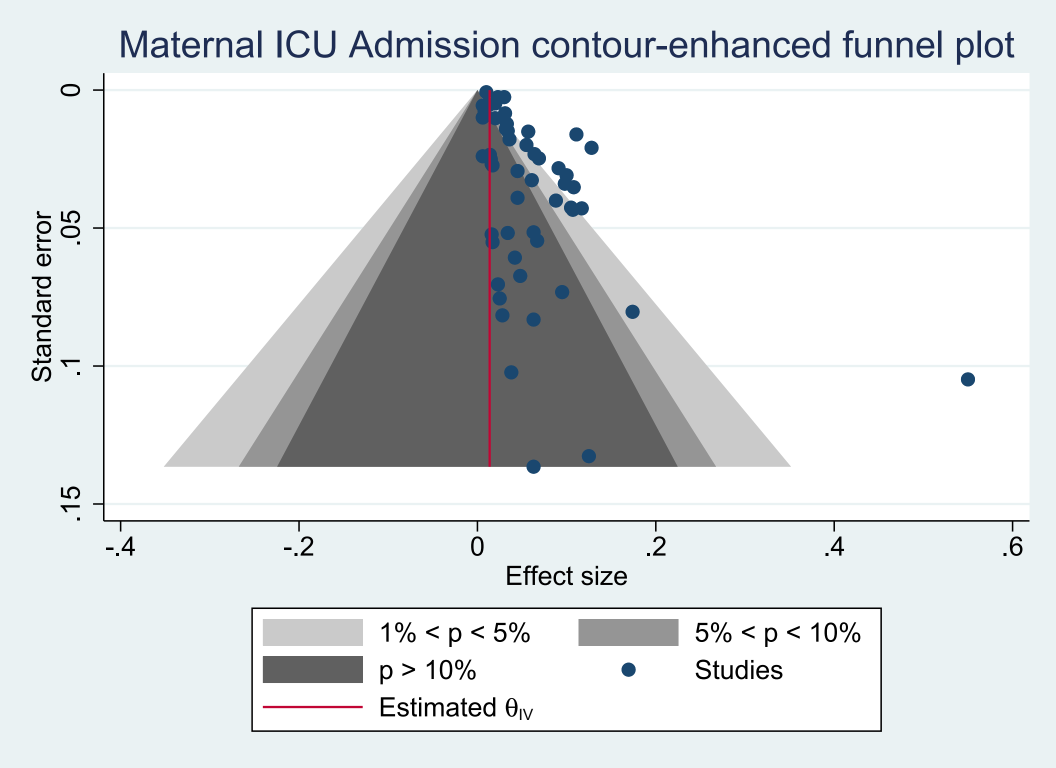

Maternal ICU Admission contour-enhanced funnel plot
Standard error
0
.05
.1
.15
-.4
-.2
0
.2
.4
.6
Effect size
1% < p < 5%
5% < p < 10%
p > 10%
Studies
Estimated θ_{IV}

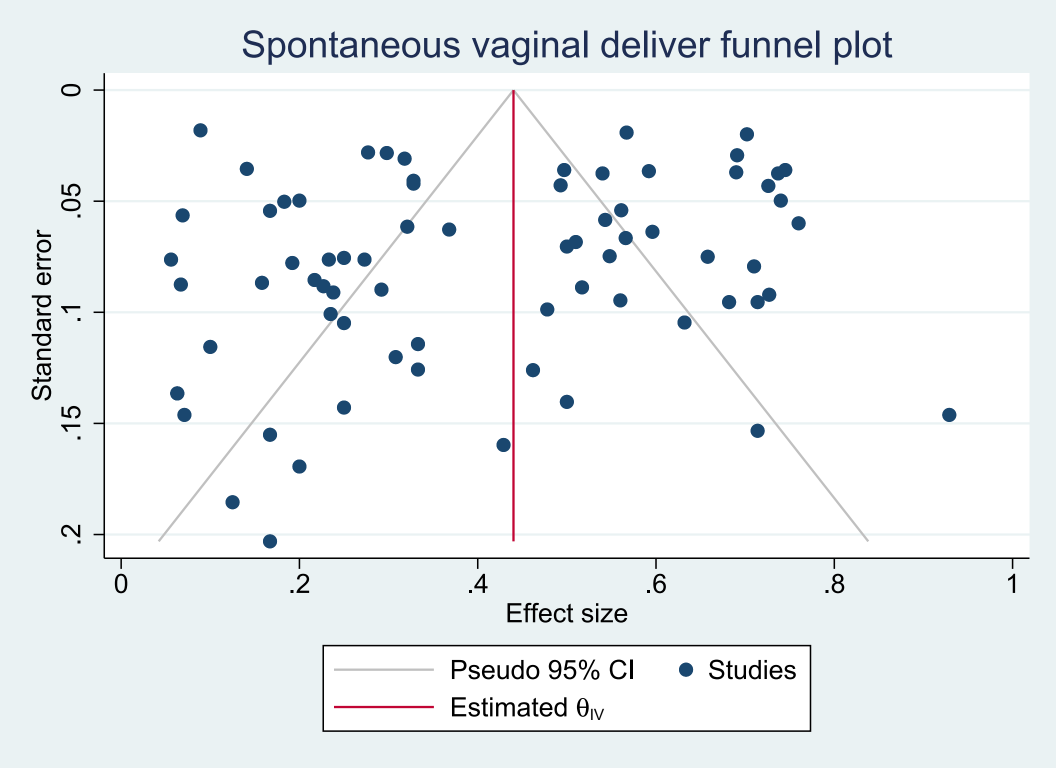

Spontaneous vaginal deliver funnel plot
Standard error
0
.05
.1
.15
.2
0
.2
.4
.6
.8
1
Effect size
Pseudo 95% CI
Studies
Estimated θ_{IV}

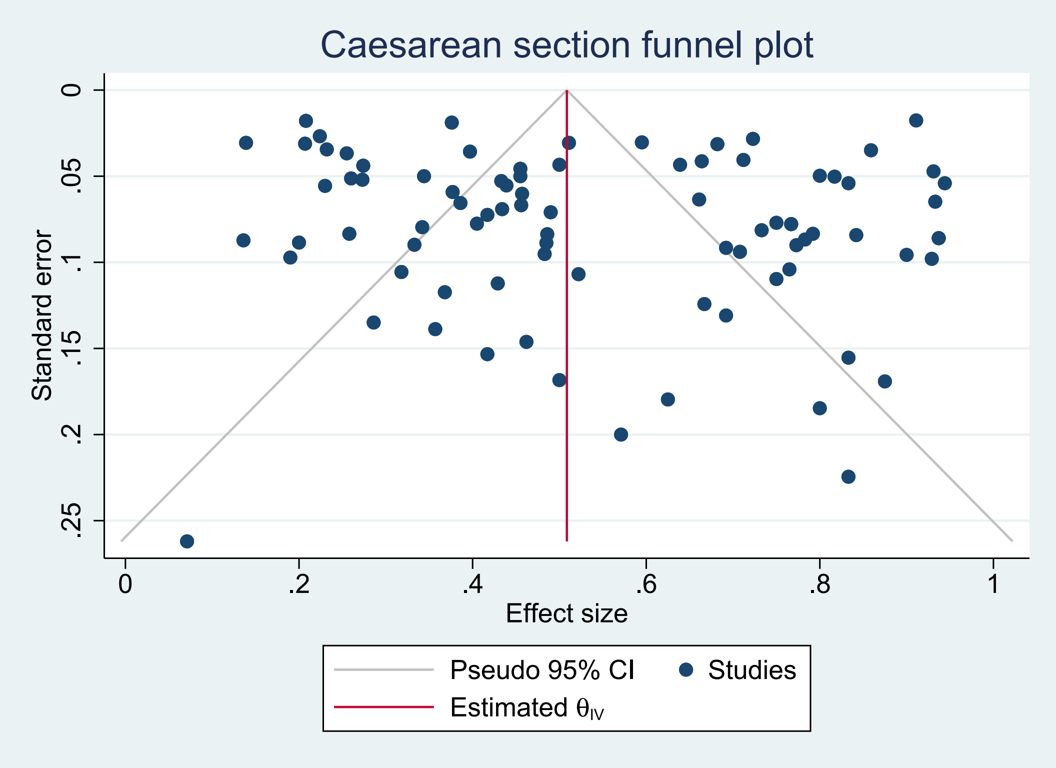

Caesarean section funnel plot
Standard error
0
.05
.1
.15
.2
.25
0
.2
.4
.6
.8
1
Effect size
Pseudo 95% CI
Studies
Estimated θ_{IV}

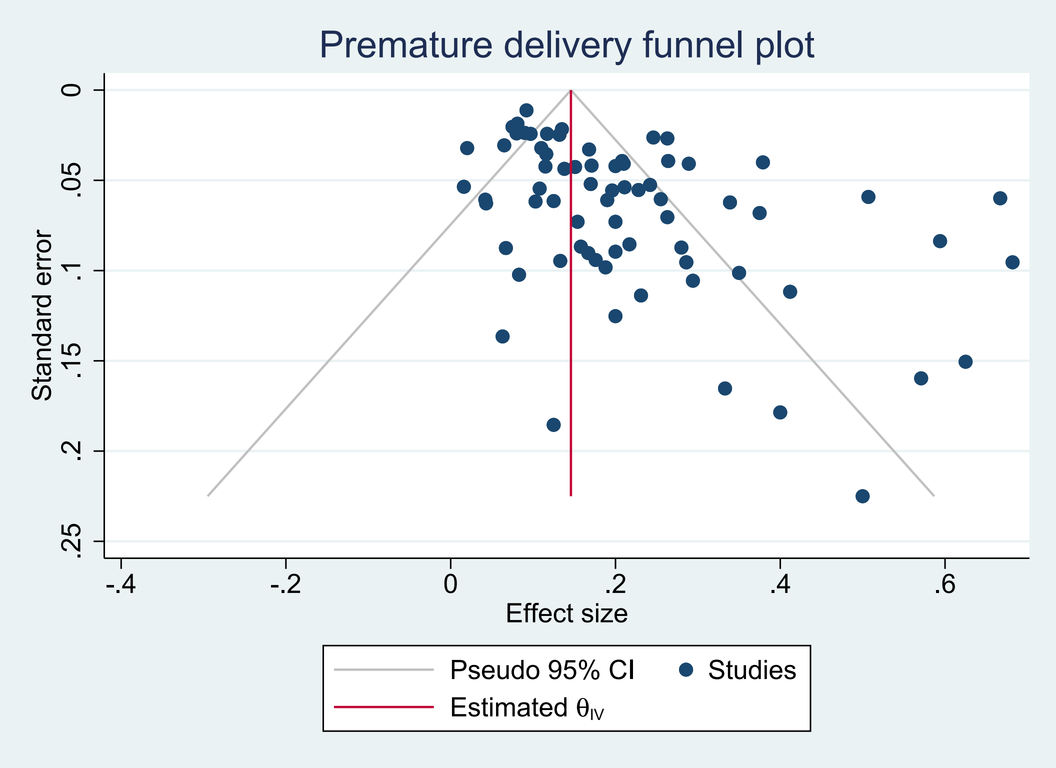
Premature delivery funnel plot
Standard error
0
.05
.1
.15
.2
.25
-.4
-.2
0
.2
.4
.6
Effect size
Pseudo 95% CI
Studies
Estimated θIV

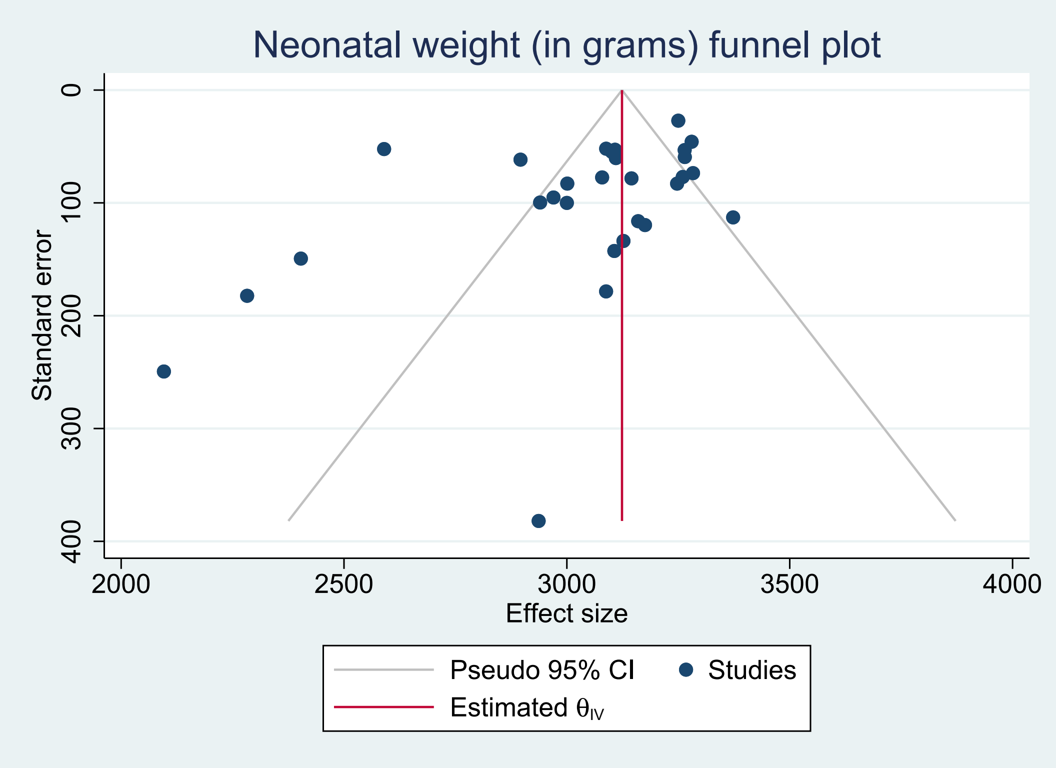

Neonatal weight (in grams) funnel plot
Standard error
0
100
200
300
400
2000
2500
3000
3500
4000
Effect size
Pseudo 95% CI
Studies
Estimated θ_{IV}

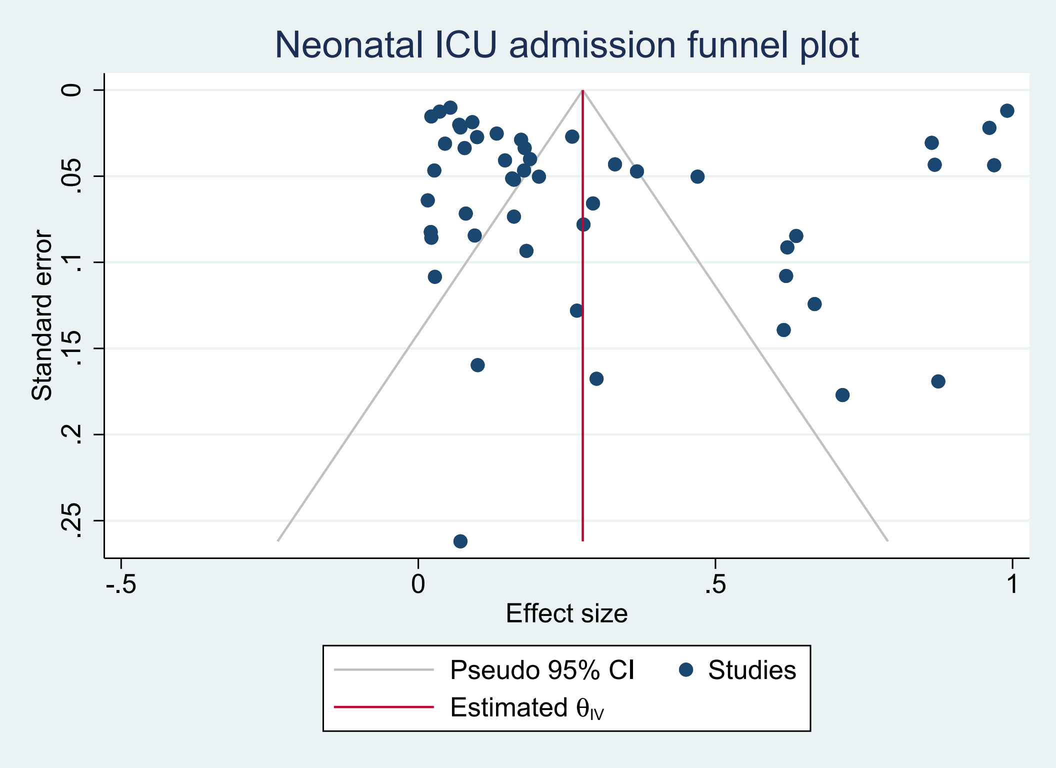
Neonatal ICU admission funnel plot
Standard error
0
.05
.1
.15
.2
.25
-.5
0
.5
1
Effect size
Pseudo 95% CI
Studies
Estimated θIV

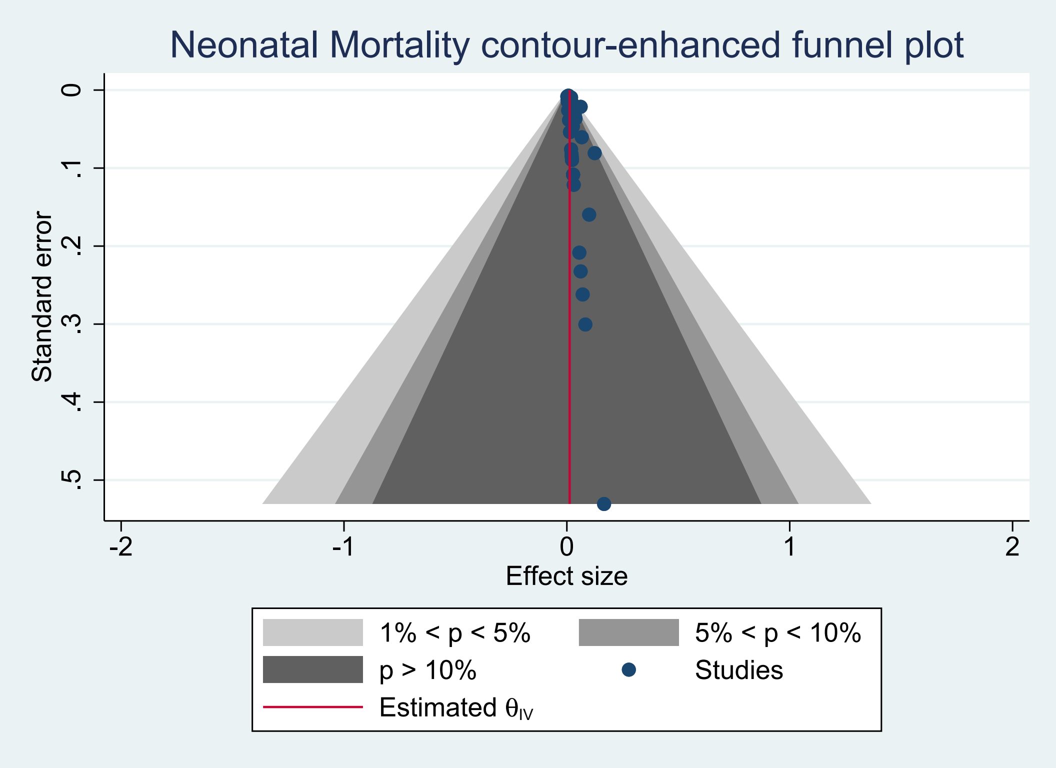
Neonatal Mortality contour-enhanced funnel plot
Standard error
0
.1
.2
.3
.4
.5
-2
-1
0
1
2
Effect size
1% < p < 5%
5% < p < 10%
p > 10%
Studies
Estimated θIV

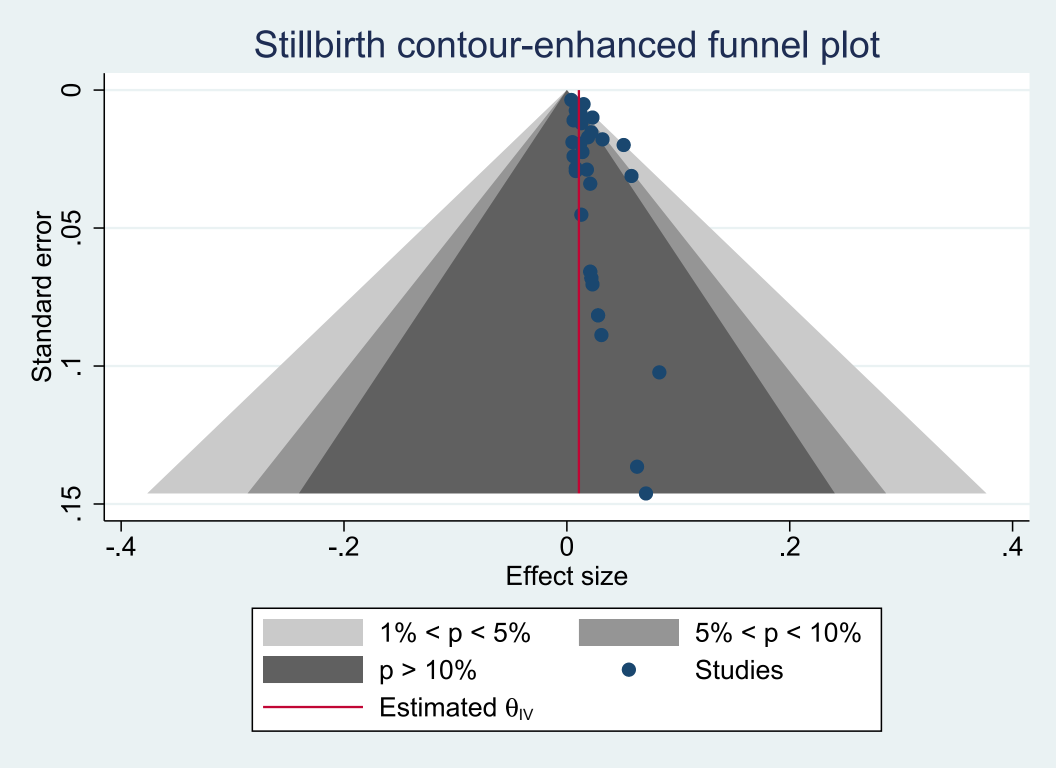
Stillbirth contour-enhanced funnel plot
Standard error
0
.05
.1
.15
-.4
-.2
0
.2
.4
Effect size
1% < p < 5%
5% < p < 10%
p > 10%
Studies
Estimated θIV

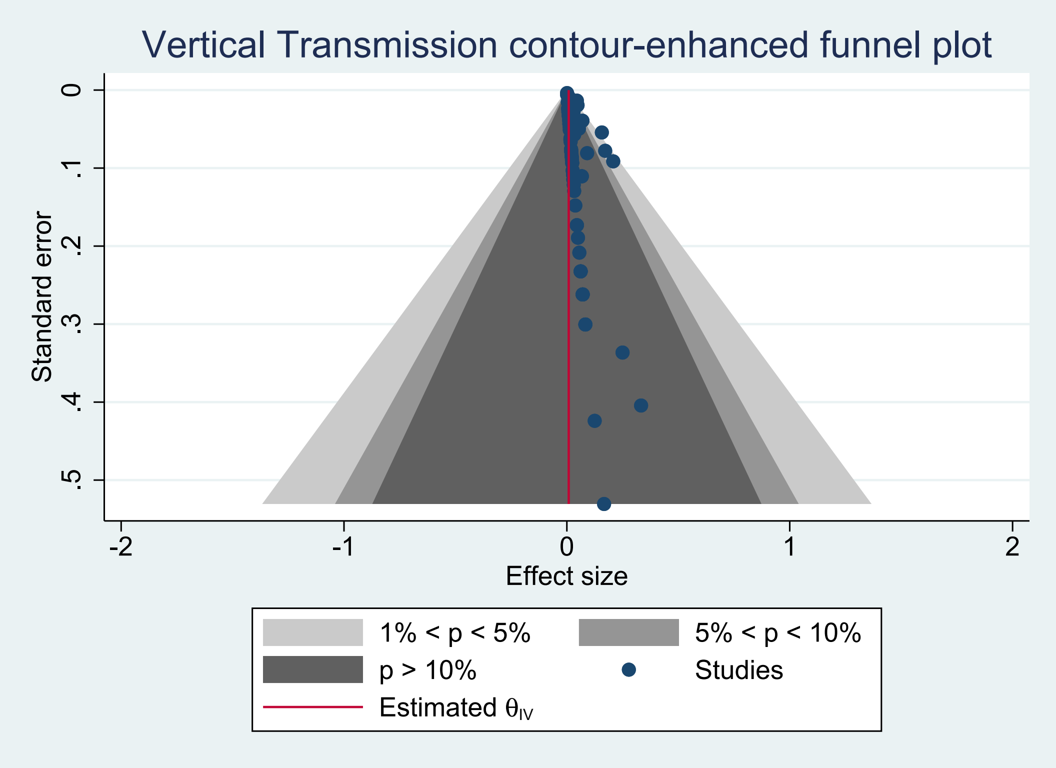

Vertical Transmission contour-enhanced funnel plot
Standard error
0
.1
.2
.3
.4
.5
-2
-1
0
1
2
Effect size
1% < p < 5%
5% < p < 10%
p > 10%
Studies
Estimated θIV
