# Supplementary material for: Systematic review and meta-analysis of COVID-19 maternal and neonatal clinical features and pregnancy outcomes up to June 3, 2021
Source: AJOG Glob Rep. 2022 Jan 3;2(1):100049. doi: 10.1016/j.xagr.2021.100049 (PMC8720679; doi:10.1016/j.xagr.2021.100049)

1. Mean Weight at Birth


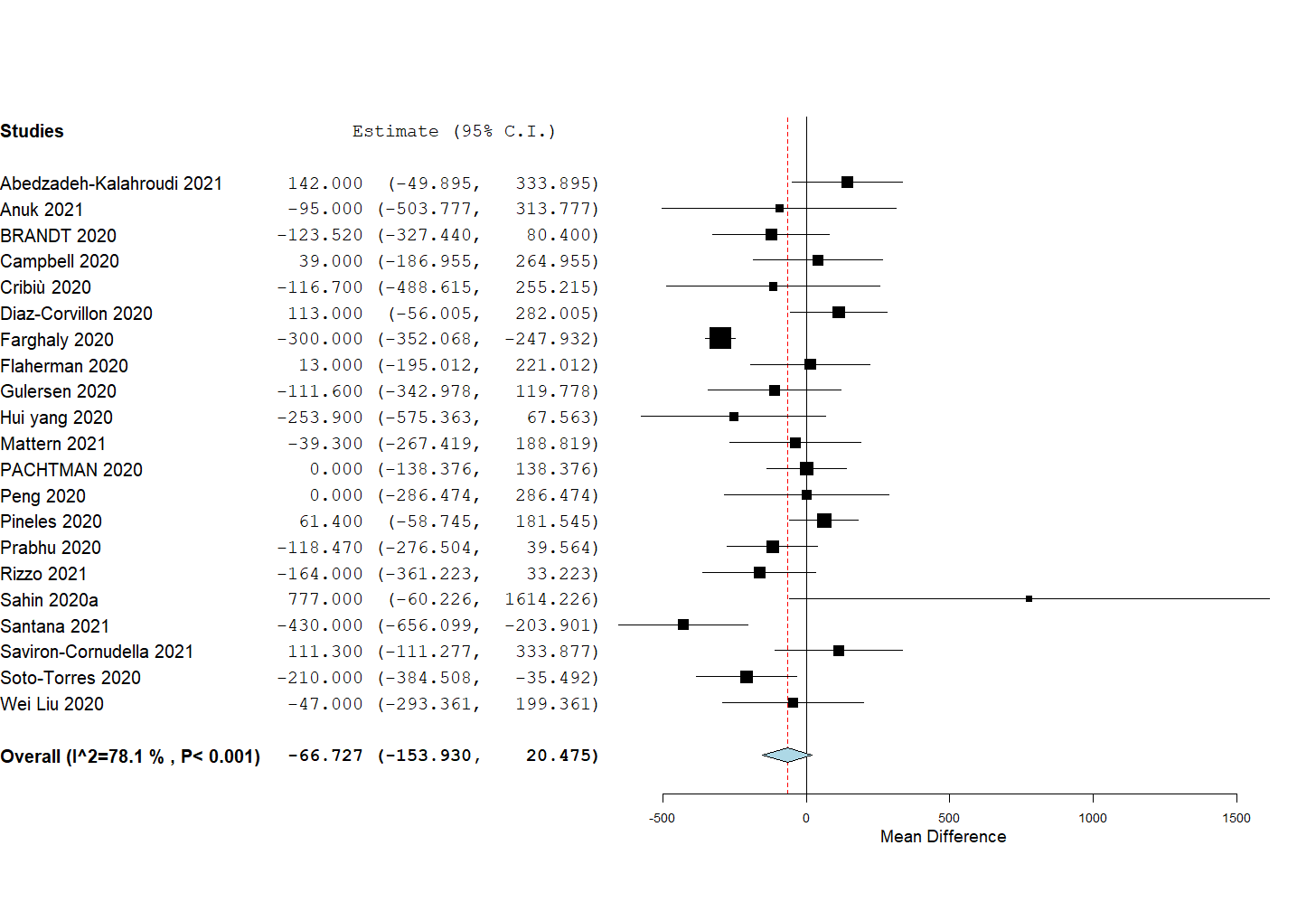


1. Low birthweight


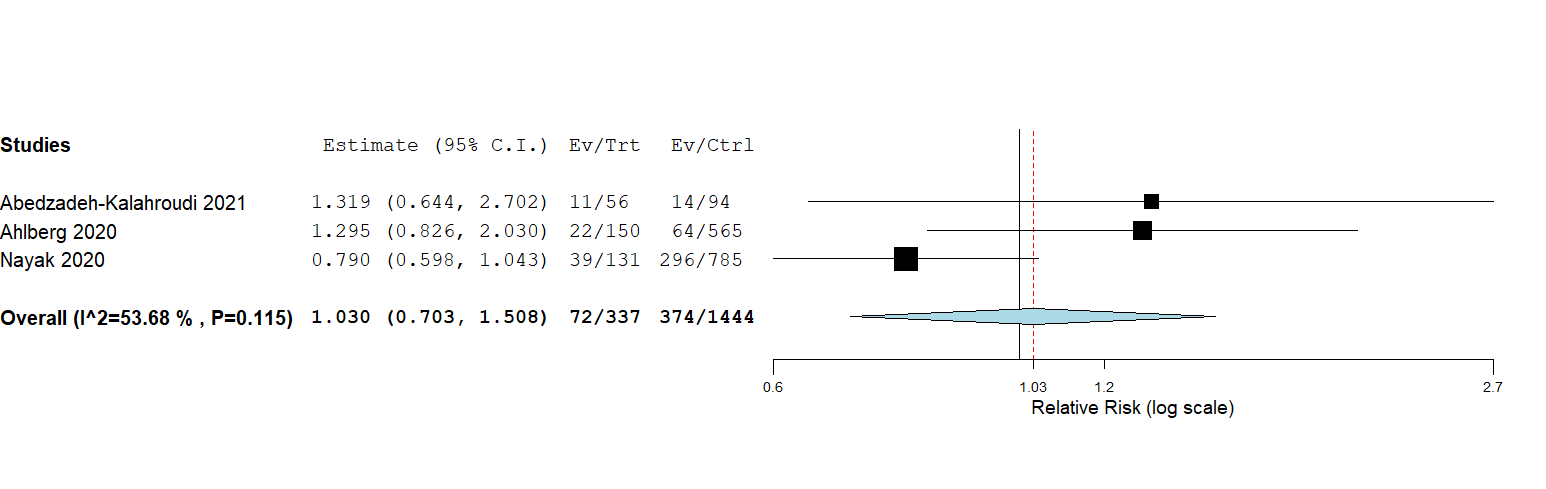


1. 5-min Apgar score


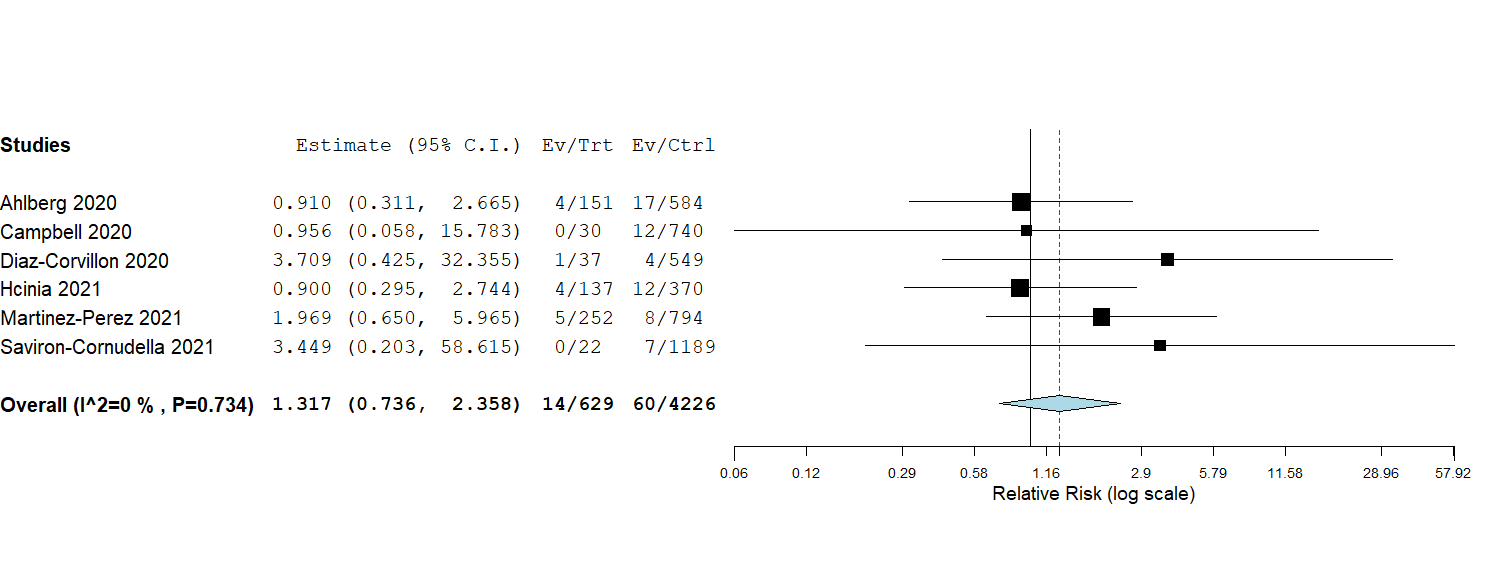


1. Preterm below 37 weeks


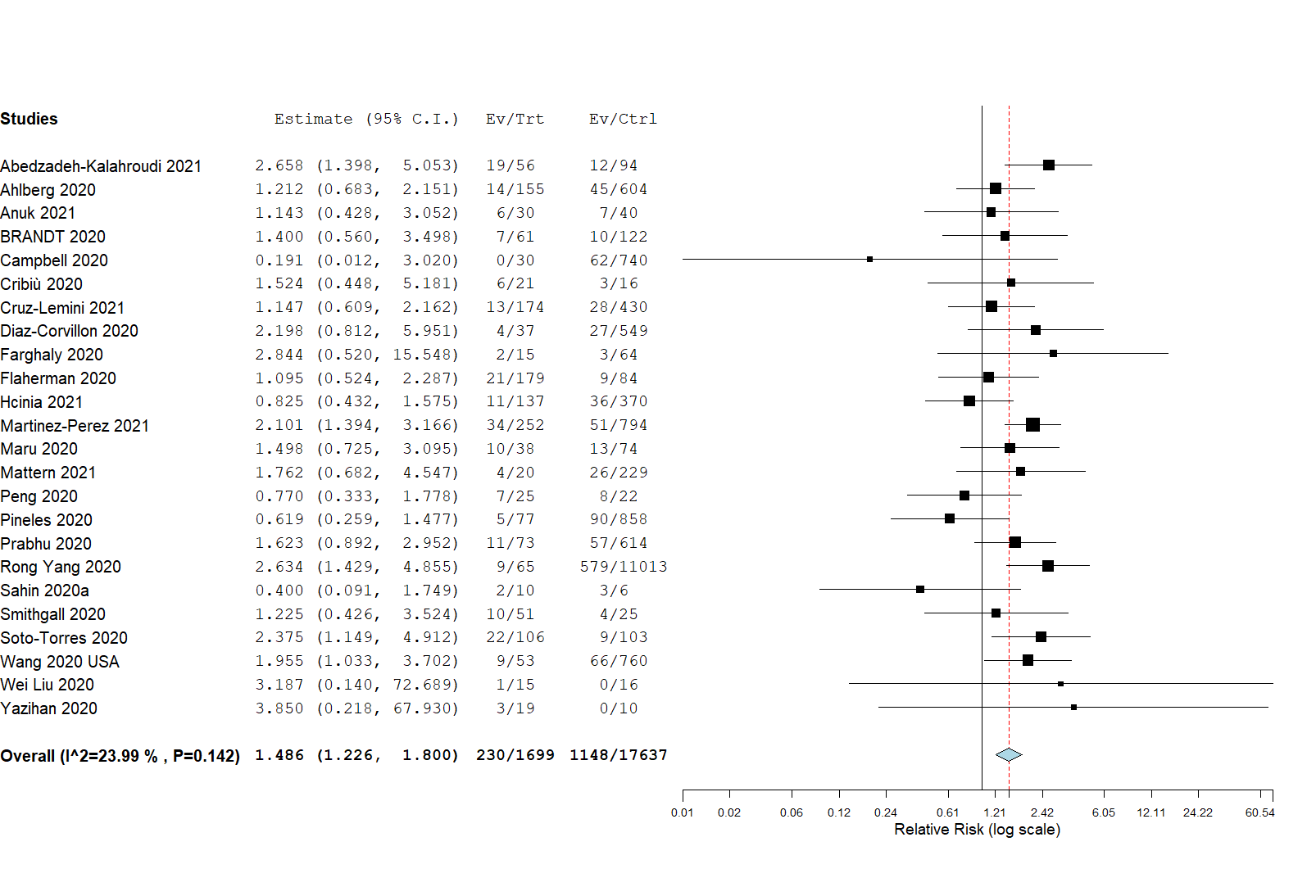


1. Cesarean section


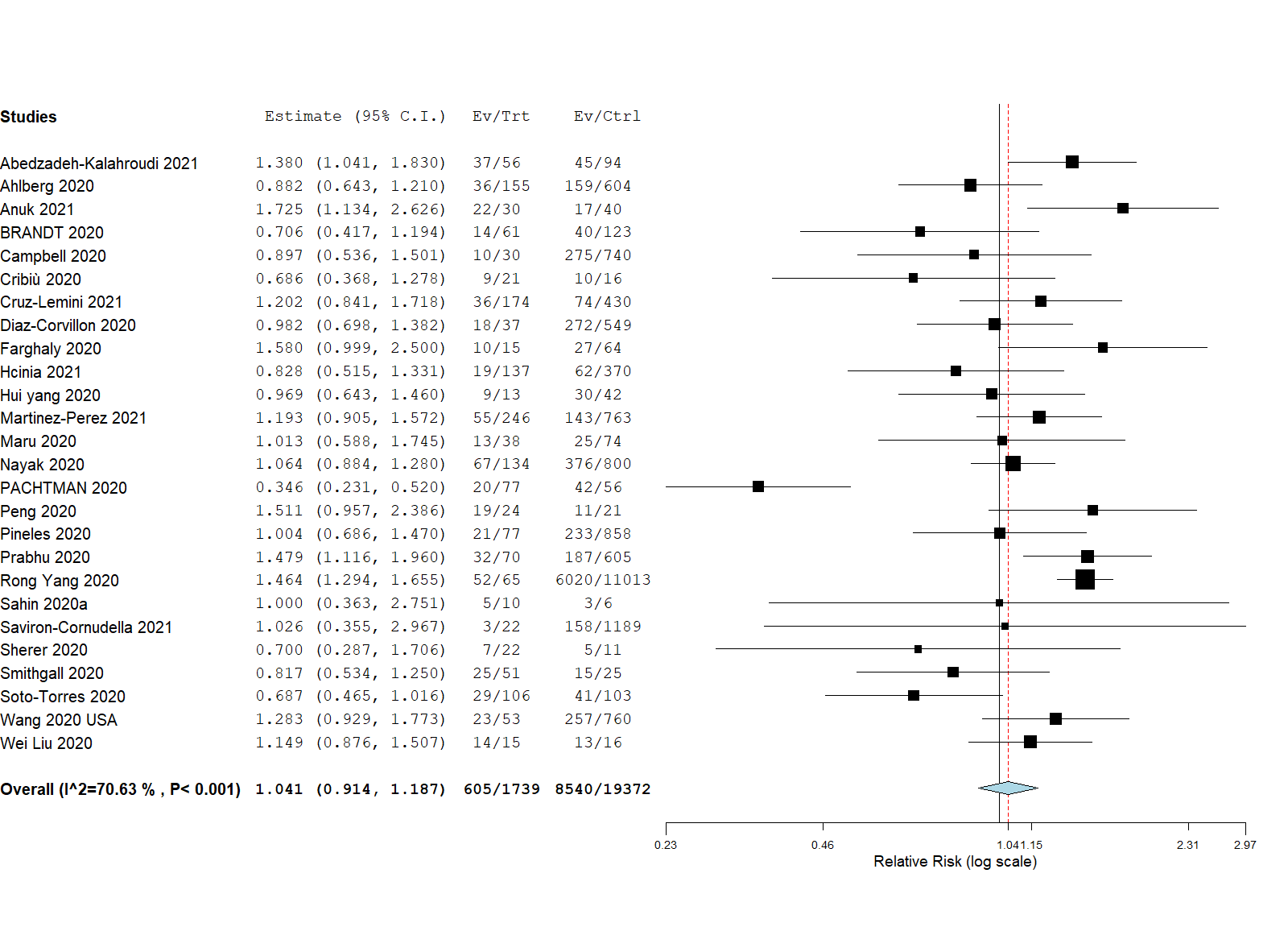


1. Spontaneous vaginal delivery


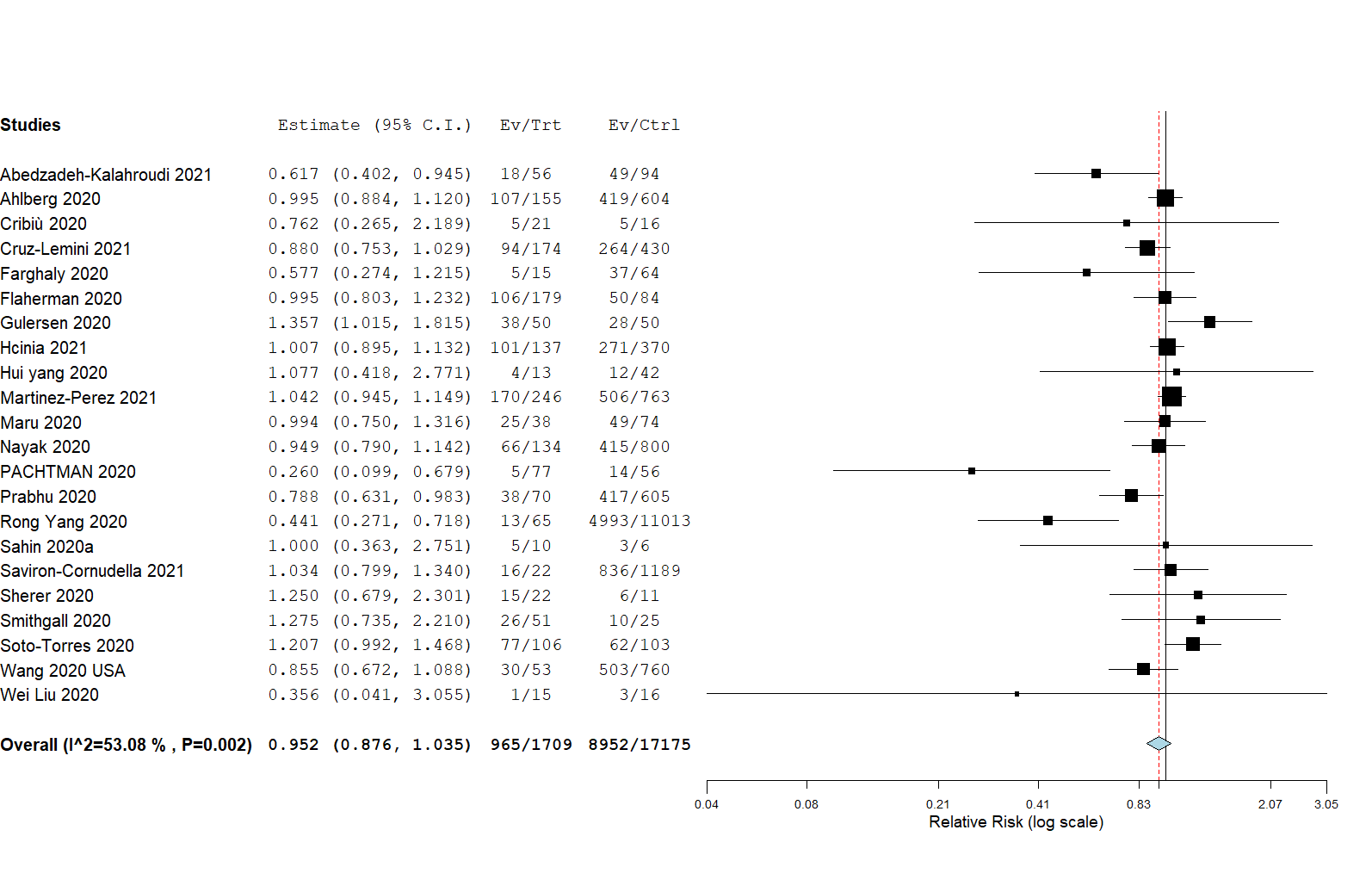


1. Preeclampsia


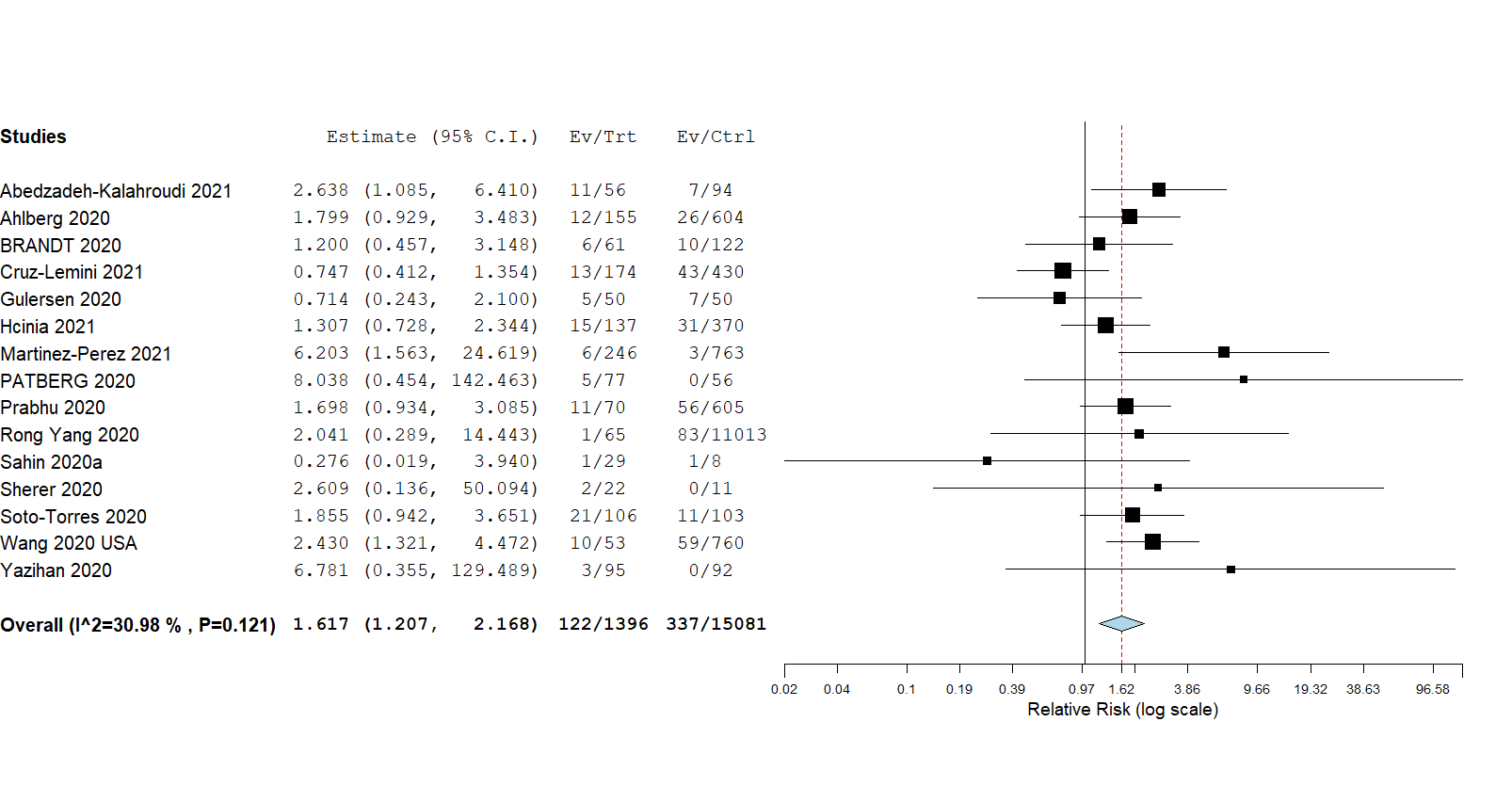


1. Gestational diabetes


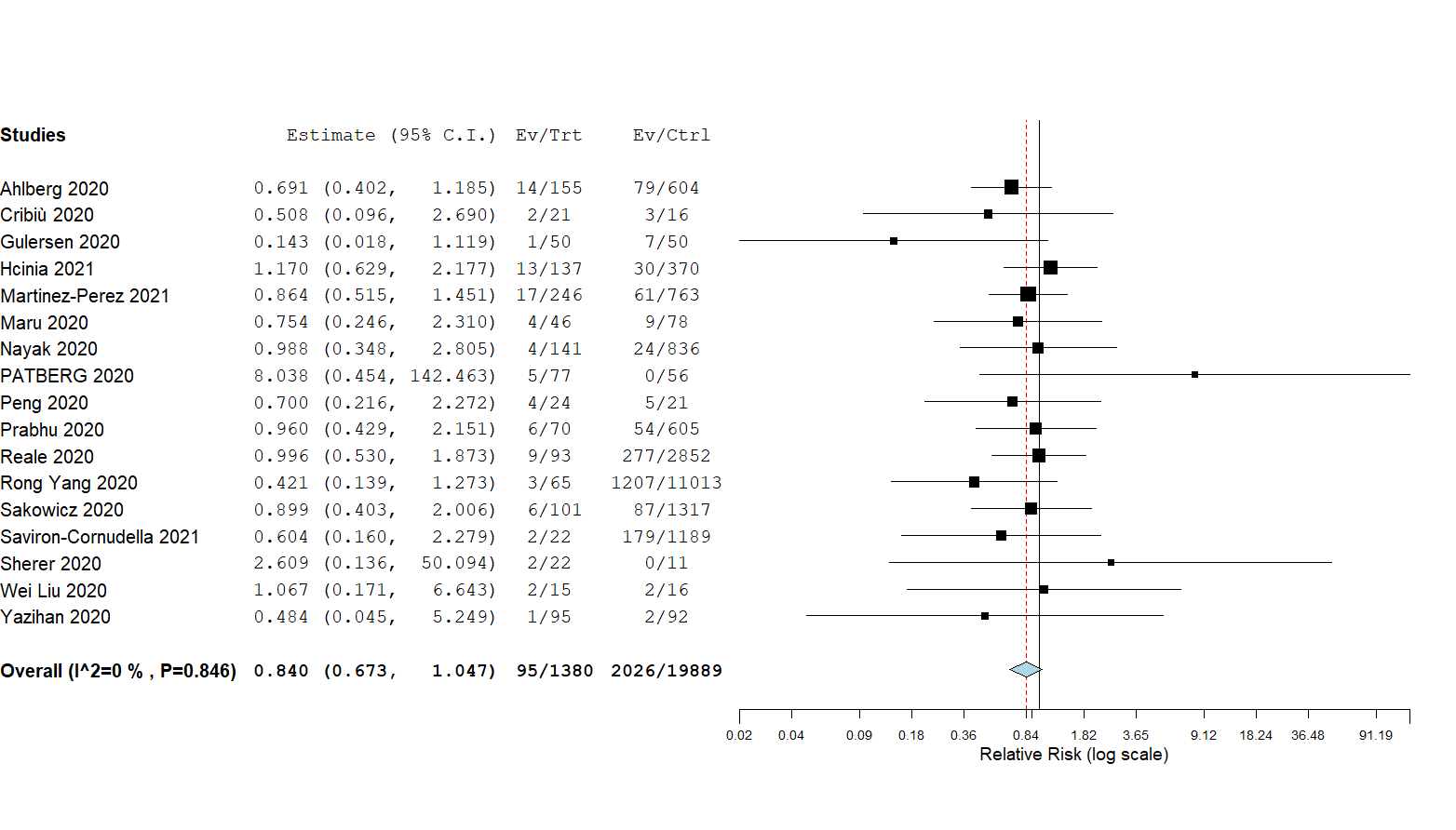


1. Maternal ICU admission


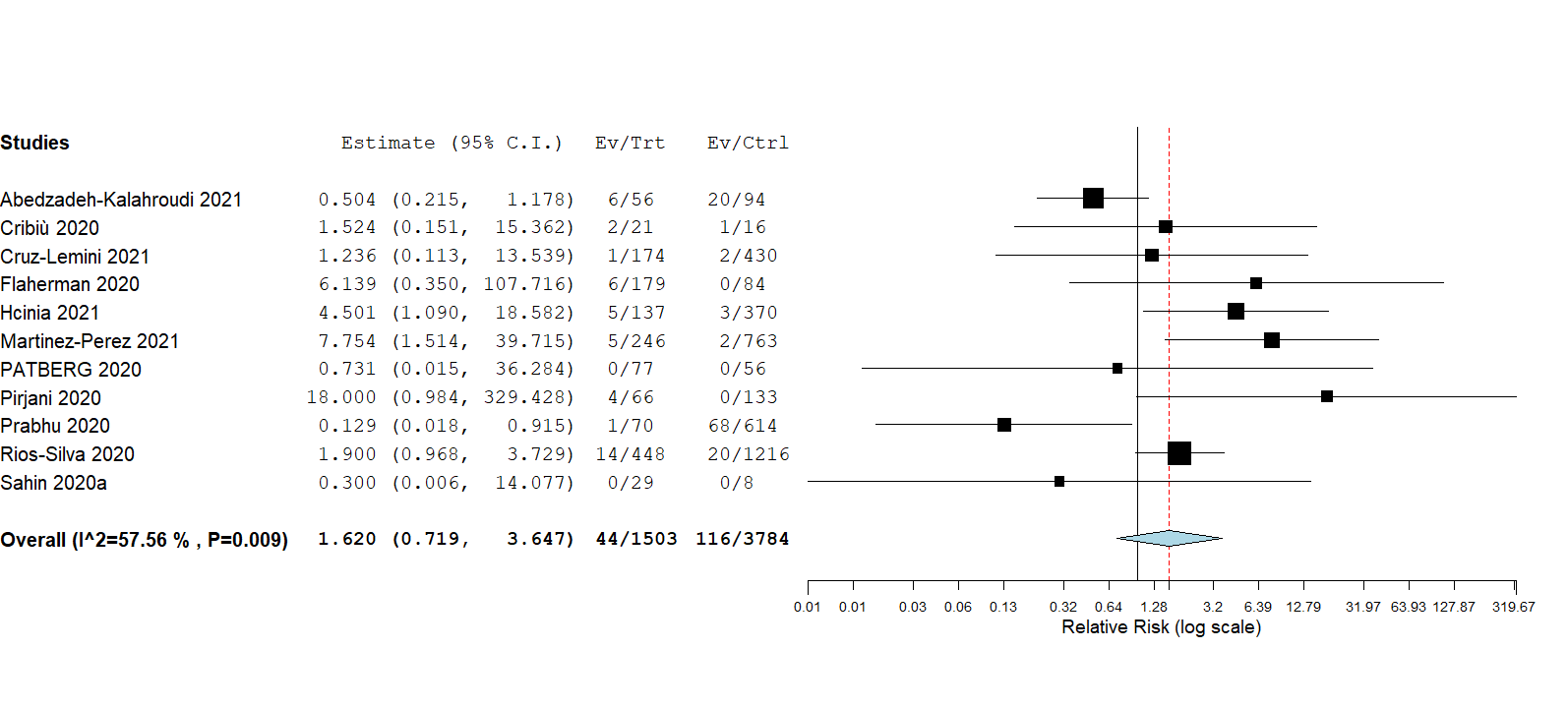


1. NICU admission.


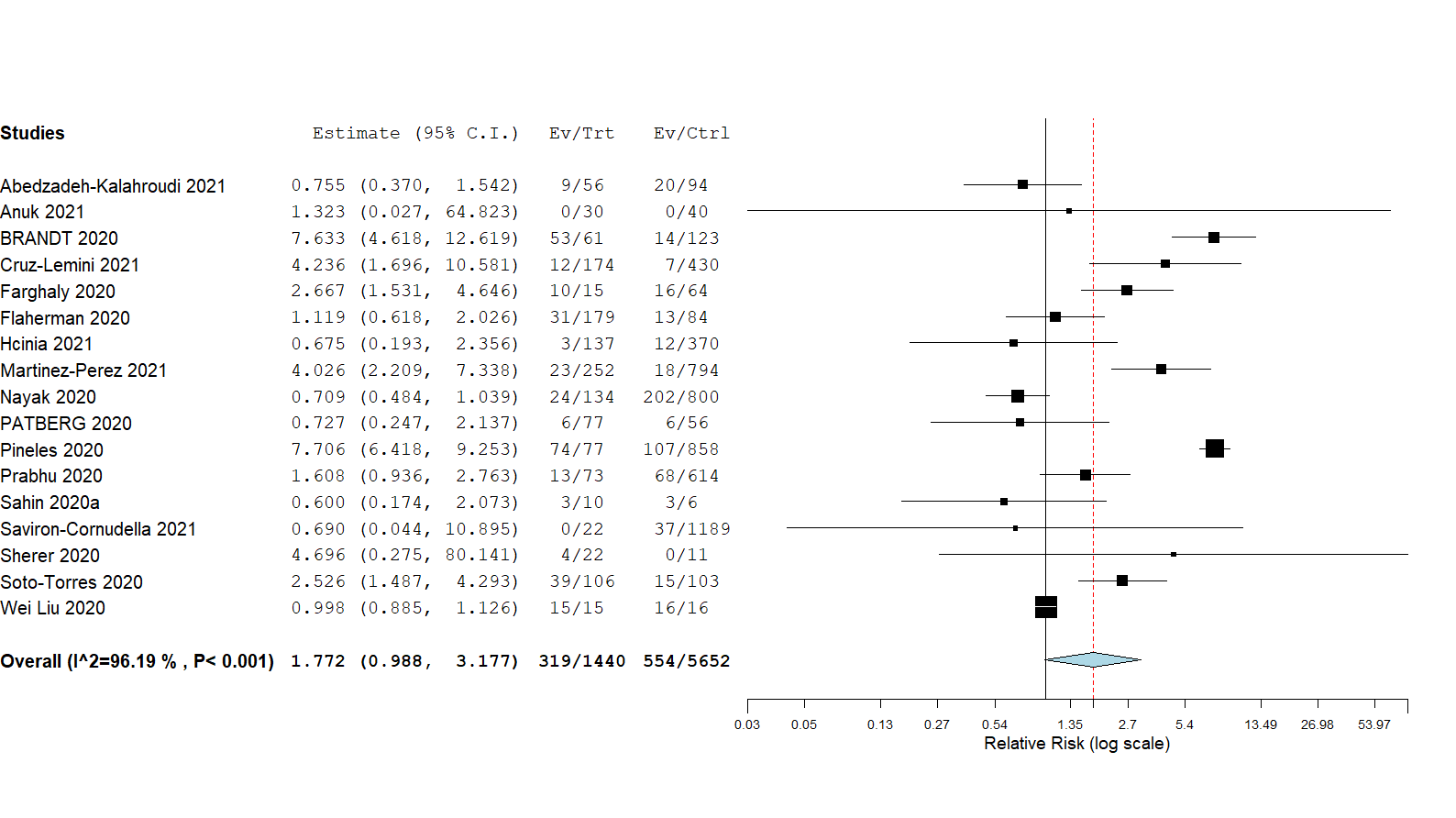


1. Stillbirth


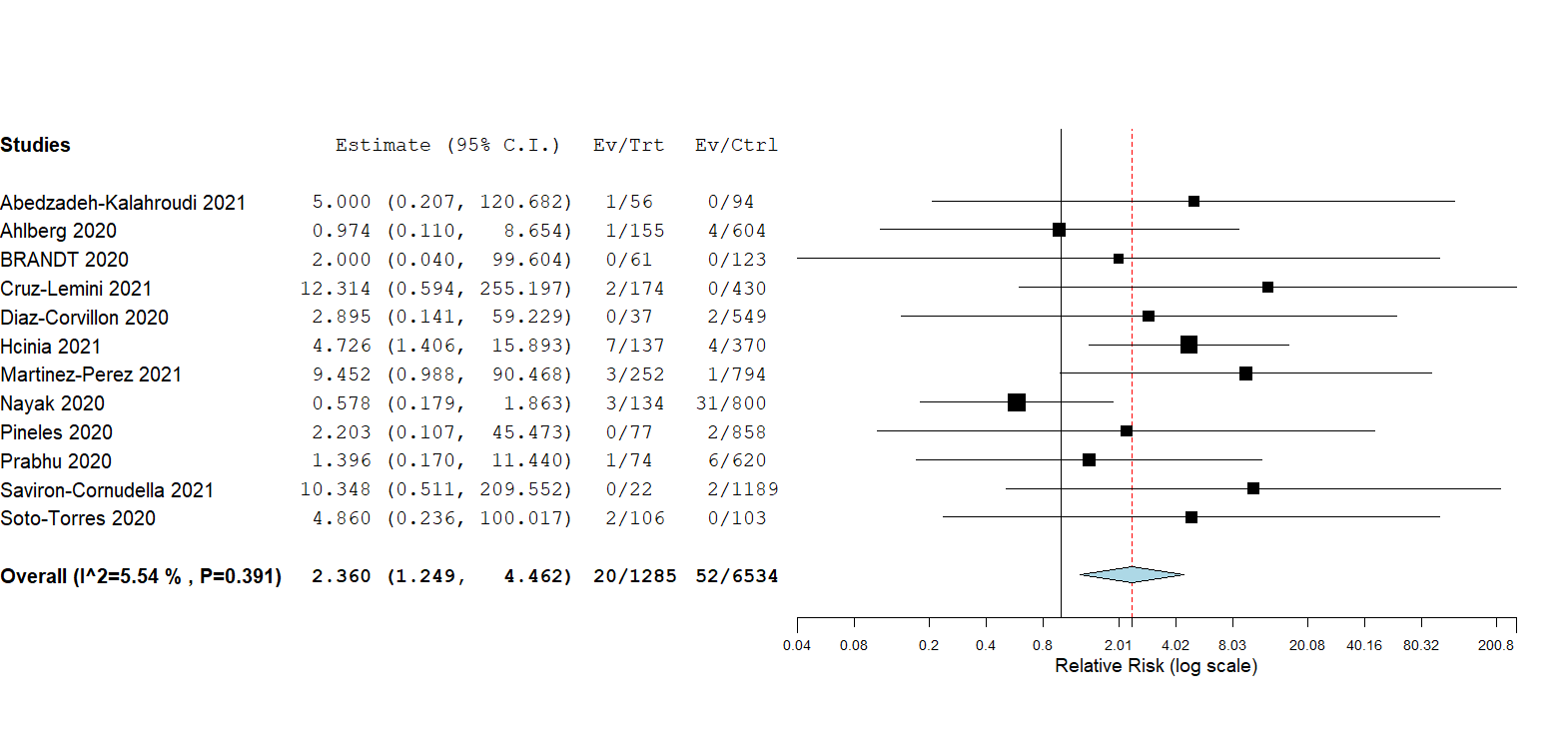


1. Neonatal mortality rate


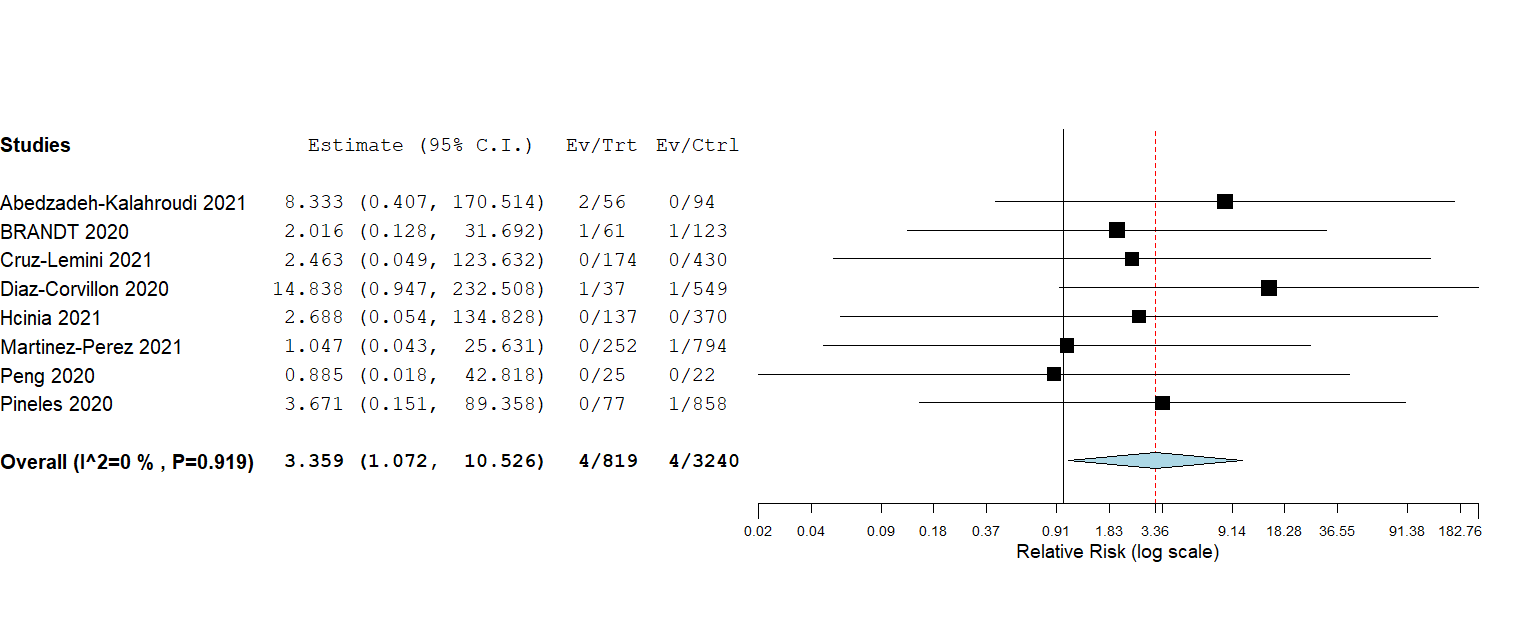


1. Vertical transmission


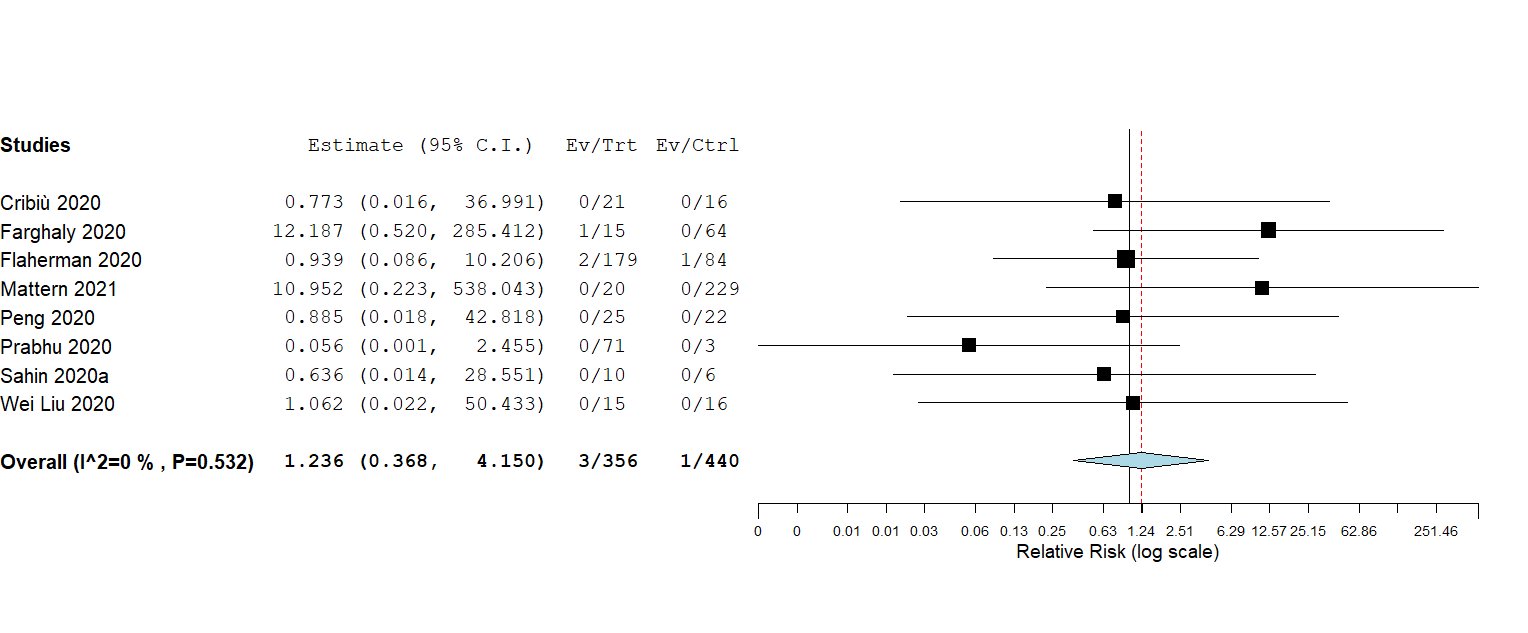


1. Maternal Mortality rate


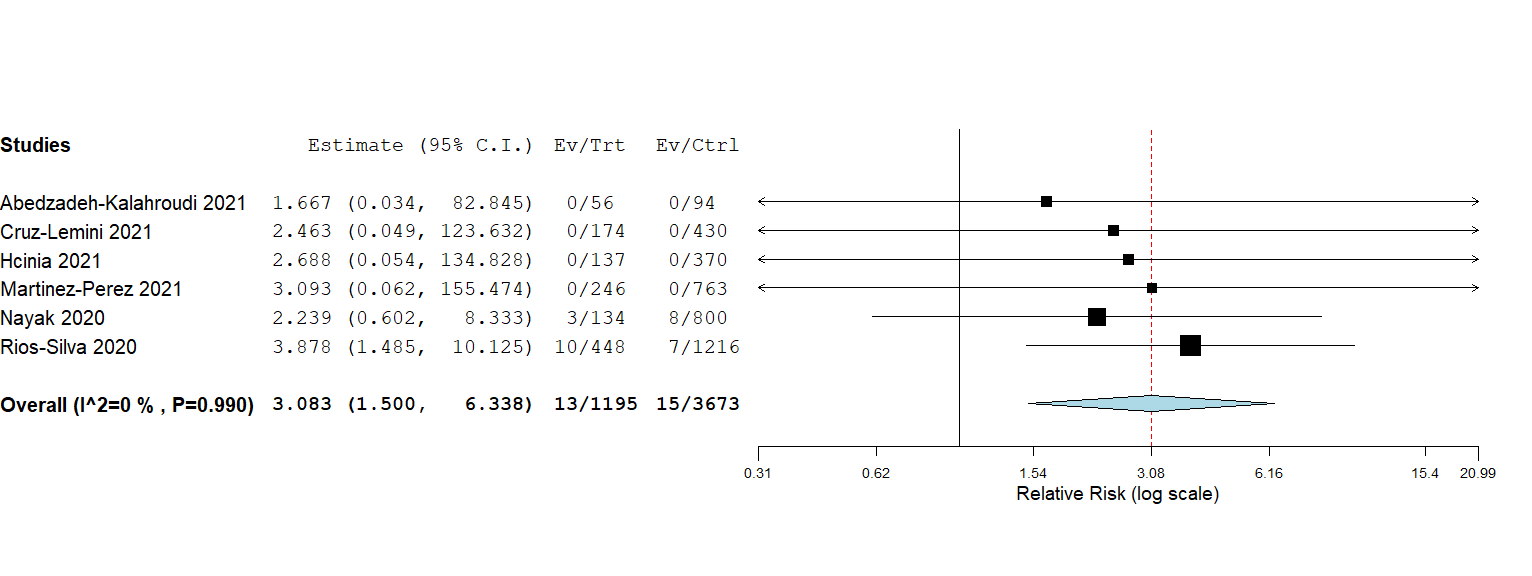

Supplement: Supplementary file 3 [file mmc3.docx]
