# Supplementary material for: Systematic review and meta-analysis of COVID-19 maternal and neonatal clinical features and pregnancy outcomes up to June 3, 2021
Source: AJOG Glob Rep. 2022 Jan 3;2(1):100049. doi: 10.1016/j.xagr.2021.100049 (PMC8720679; doi:10.1016/j.xagr.2021.100049)
Supplement: Supplementary file 4 [file mmc4.pdf]

Studies	Estimate (95% C.I.)
Ahlberg 2020	0.090 (0.053, 0.149)
Barbero 2020	0.033 (0.010, 0.100)
Gulersen 2020	0.020 (0.003, 0.139)
Knight 2020	0.117 (0.089, 0.152)
Lu Zhang 2020	0.167 (0.044, 0.468)
Mahajan 2021	0.020 (0.012, 0.032)
Maru 2020	0.087 (0.031, 0.222)
Ozsurmeli 2021	0.083 (0.018, 0.314)
Prabhu 2020	0.086 (0.038, 0.184)
Sahin 2020	0.006 (0.002, 0.017)
Samadi 2021	0.178 (0.134, 0.232)
Santhosh 2021	0.233 (0.136, 0.369)
Saviron−Cornudella 2021	0.091 (0.019, 0.341)
Soffer 2021	0.134 (0.068, 0.247)
Tug 2020	0.005 (0.001, 0.038)
Villalan 2020	0.059 (0.042, 0.083)
Vizheh 2021	0.045 (0.018, 0.107)
Wang 2020 (China)	0.067 (0.015, 0.254)
Wu 2020	0.103 (0.030, 0.302)
Peng 2020	0.167 (0.055, 0.408)
**Subgroup Fair quality (I^2=85.36 % , P=0.000)**	**0.070 (0.046, 0.104)**
Dumitriu 2020	0.090 (0.046, 0.168)
Hcinia 2021	0.095 (0.055, 0.159)
PACHTMAN 2020	0.050 (0.005, 0.338)
PATBERG 2020	0.065 (0.026, 0.152)
Reale 2020	0.097 (0.050, 0.180)
Rong Yang 2020	0.046 (0.014, 0.139)
Sakowicz 2020	0.059 (0.026, 0.129)
San−juan 2020	0.062 (0.014, 0.239)
Wei Liu 2020	0.133 (0.024, 0.484)
**Subgroup Good quality (I^2=0 % , P=0.920)**	**0.081 (0.061, 0.106)**
Cohen 2020	0.057 (0.023, 0.133)
Cribiù 2020	0.095 (0.020, 0.356)
Facchetti 2020	0.133 (0.024, 0.484)
Gaspar 2021	0.038 (0.001, 0.583)
Hui Yang 2020	0.111 (0.032, 0.323)
Liu 2020	0.095 (0.020, 0.356)
Llorca 2021	0.033 (0.001, 0.509)
Lokken 2021	0.103 (0.063, 0.164)
Lopian 2020	0.238 (0.085, 0.512)
Nayak 2020	0.028 (0.010, 0.075)
Oncel 2020	0.058 (0.027, 0.119)
Qiancheng 2020	0.071 (0.015, 0.272)
Santana 2021	0.138 (0.047, 0.343)
Sherer 2020	0.091 (0.019, 0.341)
Suyuthi 2020	0.038 (0.004, 0.263)
Xu 2020 (China)	0.059 (0.013, 0.226)
Yan 2020	0.078 (0.040, 0.145)
**Subgroup Poor quality (I^2=0 % , P=0.600)**	**0.081 (0.063, 0.104)**
**Overall (I^2=69.49 % , P=0.000)**	**0.075 (0.060, 0.094)**

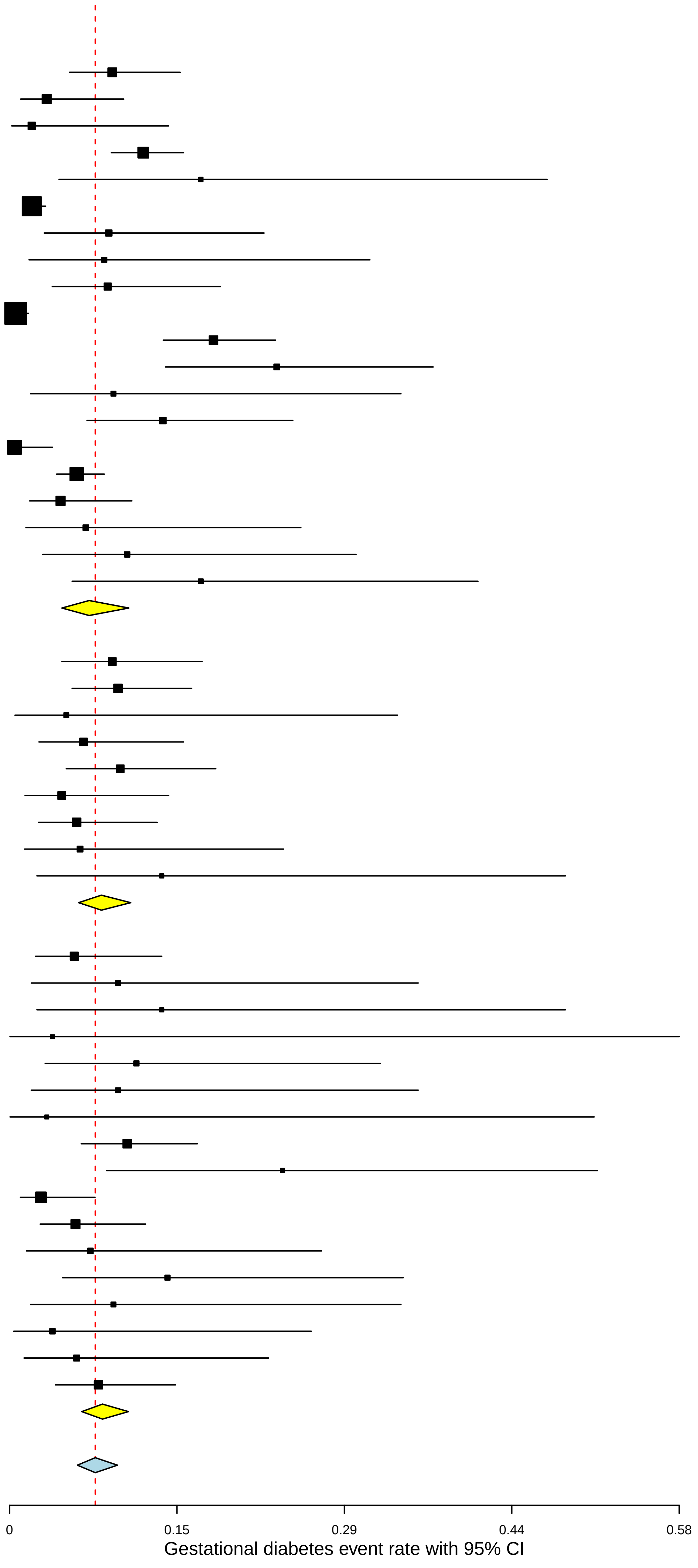

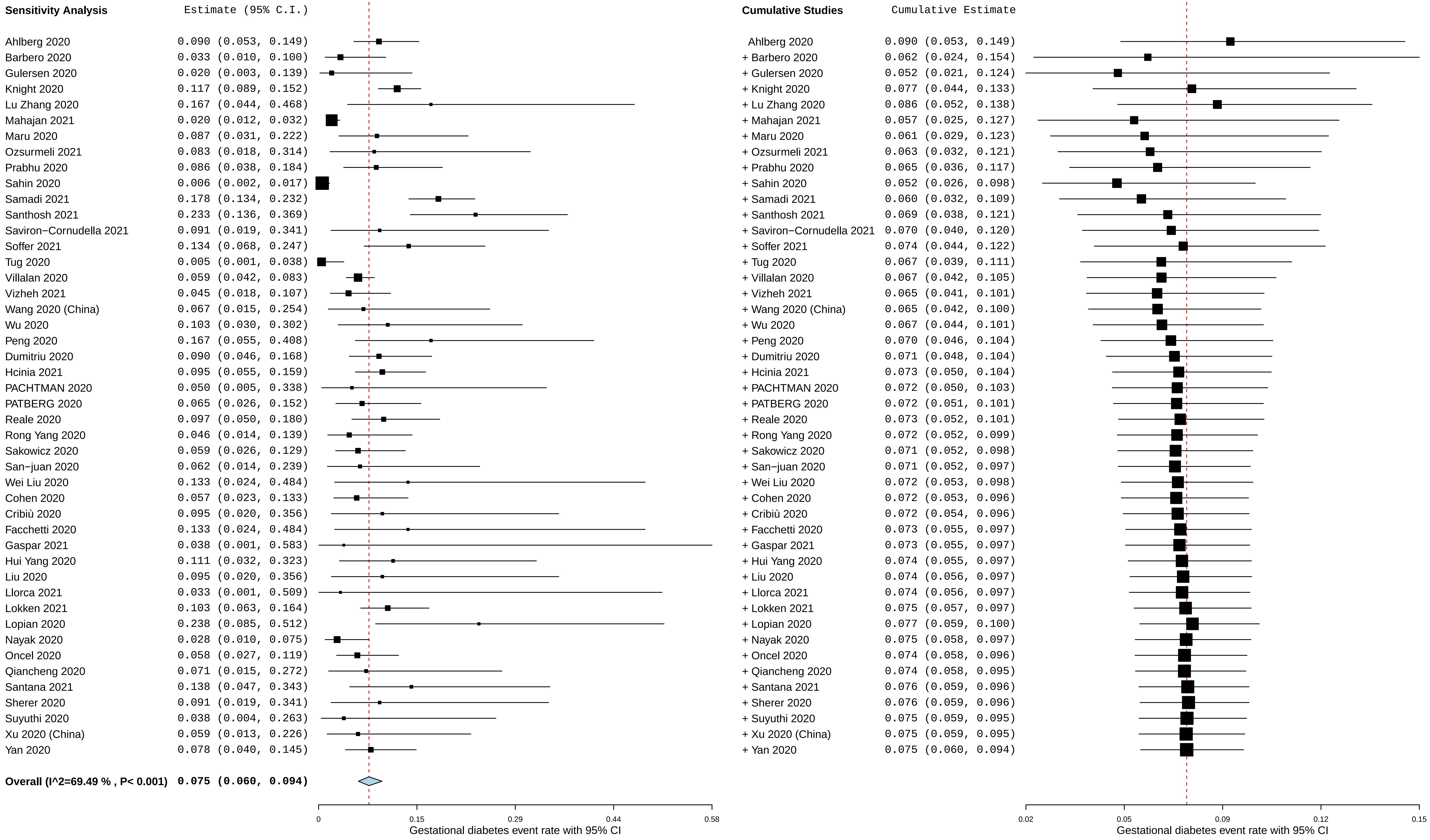
Sensitivity Analysis
Estimate (95% C.I.)
Ahlberg 2020 0.090 (0.053, 0.149)
Barbero 2020 0.033 (0.010, 0.100)
Gulersen 2020 0.020 (0.003, 0.139)
Knight 2020 0.117 (0.089, 0.152)
Lu Zhang 2020 0.167 (0.044, 0.468)
Mahajan 2021 0.020 (0.012, 0.032)
Maru 2020 0.087 (0.031, 0.222)
Ozsurmeli 2021 0.083 (0.018, 0.314)
Prabhu 2020 0.086 (0.038, 0.184)
Sahin 2020 0.006 (0.002, 0.017)
Samadi 2021 0.178 (0.134, 0.232)
Santhosh 2021 0.233 (0.136, 0.369)
Saviron−Cornudella 2021 0.091 (0.019, 0.341)
Soffer 2021 0.134 (0.068, 0.247)
Tug 2020 0.005 (0.001, 0.038)
Villalan 2020 0.059 (0.042, 0.083)
Vizheh 2021 0.045 (0.018, 0.107)
Wang 2020 (China) 0.067 (0.015, 0.254)
Wu 2020 0.103 (0.030, 0.302)
Peng 2020 0.167 (0.055, 0.408)
Dumitriu 2020 0.090 (0.046, 0.168)
Hcinia 2021 0.095 (0.055, 0.159)
PACHTMAN 2020 0.050 (0.005, 0.338)
PATBERG 2020 0.065 (0.026, 0.152)
Reale 2020 0.097 (0.050, 0.180)
Rong Yang 2020 0.046 (0.014, 0.139)
Sakowicz 2020 0.059 (0.026, 0.129)
San−juan 2020 0.062 (0.014, 0.239)
Wei Liu 2020 0.133 (0.024, 0.484)
Cohen 2020 0.057 (0.023, 0.133)
Cribiù 2020 0.095 (0.020, 0.356)
Facchetti 2020 0.133 (0.024, 0.484)
Gaspar 2021 0.038 (0.001, 0.583)
Hui Yang 2020 0.111 (0.032, 0.323)
Liu 2020 0.095 (0.020, 0.356)
Llorca 2021 0.033 (0.001, 0.509)
Lokken 2021 0.103 (0.063, 0.164)
Lopian 2020 0.238 (0.085, 0.512)
Nayak 2020 0.028 (0.010, 0.075)
Oncel 2020 0.058 (0.027, 0.119)
Qiancheng 2020 0.071 (0.015, 0.272)
Santana 2021 0.138 (0.047, 0.343)
Sherer 2020 0.091 (0.019, 0.341)
Suyuthi 2020 0.038 (0.004, 0.263)
Xu 2020 (China) 0.059 (0.013, 0.226)
Yan 2020 0.078 (0.040, 0.145)
Overall (I^2=69.49 % , P< 0.001) 0.075 (0.060, 0.094)
0
0.15
0.29
0.44
0.58
Gestational diabetes event rate with 95% CI
Cumulative Studies
Cumulative Estimate
Ahlberg 2020 0.090 (0.053, 0.149)
+ Barbero 2020 0.062 (0.024, 0.154)
+ Gulersen 2020 0.052 (0.021, 0.124)
+ Knight 2020 0.077 (0.044, 0.133)
+ Lu Zhang 2020 0.086 (0.052, 0.138)
+ Mahajan 2021 0.057 (0.025, 0.127)
+ Maru 2020 0.061 (0.029, 0.123)
+ Ozsurmeli 2021 0.063 (0.032, 0.121)
+ Prabhu 2020 0.065 (0.036, 0.117)
+ Sahin 2020 0.052 (0.026, 0.098)
+ Samadi 2021 0.060 (0.032, 0.109)
+ Santhosh 2021 0.069 (0.038, 0.121)
+ Saviron−Cornudella 2021 0.070 (0.040, 0.120)
+ Soffer 2021 0.074 (0.044, 0.122)
+ Tug 2020 0.067 (0.039, 0.111)
+ Villalan 2020 0.067 (0.042, 0.105)
+ Vizheh 2021 0.065 (0.041, 0.101)
+ Wang 2020 (China) 0.065 (0.042, 0.100)
+ Wu 2020 0.067 (0.044, 0.101)
+ Peng 2020 0.070 (0.046, 0.104)
+ Dumitriu 2020 0.071 (0.048, 0.104)
+ Hcinia 2021 0.073 (0.050, 0.104)
+ PACHTMAN 2020 0.072 (0.050, 0.103)
+ PATBERG 2020 0.072 (0.051, 0.101)
+ Reale 2020 0.073 (0.052, 0.101)
+ Rong Yang 2020 0.072 (0.052, 0.099)
+ Sakowicz 2020 0.071 (0.052, 0.098)
+ San−juan 2020 0.071 (0.052, 0.097)
+ Wei Liu 2020 0.072 (0.053, 0.098)
+ Cohen 2020 0.072 (0.053, 0.096)
+ Cribiù 2020 0.072 (0.054, 0.096)
+ Facchetti 2020 0.073 (0.055, 0.097)
+ Gaspar 2021 0.073 (0.055, 0.097)
+ Hui Yang 2020 0.074 (0.055, 0.097)
+ Liu 2020 0.074 (0.056, 0.097)
+ Llorca 2021 0.074 (0.056, 0.097)
+ Lokken 2021 0.075 (0.057, 0.097)
+ Lopian 2020 0.077 (0.059, 0.100)
+ Nayak 2020 0.075 (0.058, 0.097)
+ Oncel 2020 0.074 (0.058, 0.096)
+ Qiancheng 2020 0.074 (0.058, 0.095)
+ Santana 2021 0.076 (0.059, 0.096)
+ Sherer 2020 0.076 (0.059, 0.096)
+ Suyuthi 2020 0.075 (0.059, 0.095)
+ Xu 2020 (China) 0.075 (0.059, 0.095)
+ Yan 2020 0.075 (0.060, 0.094)
0.02
0.05
0.09
0.12
0.15
Gestational diabetes event rate with 95% CI

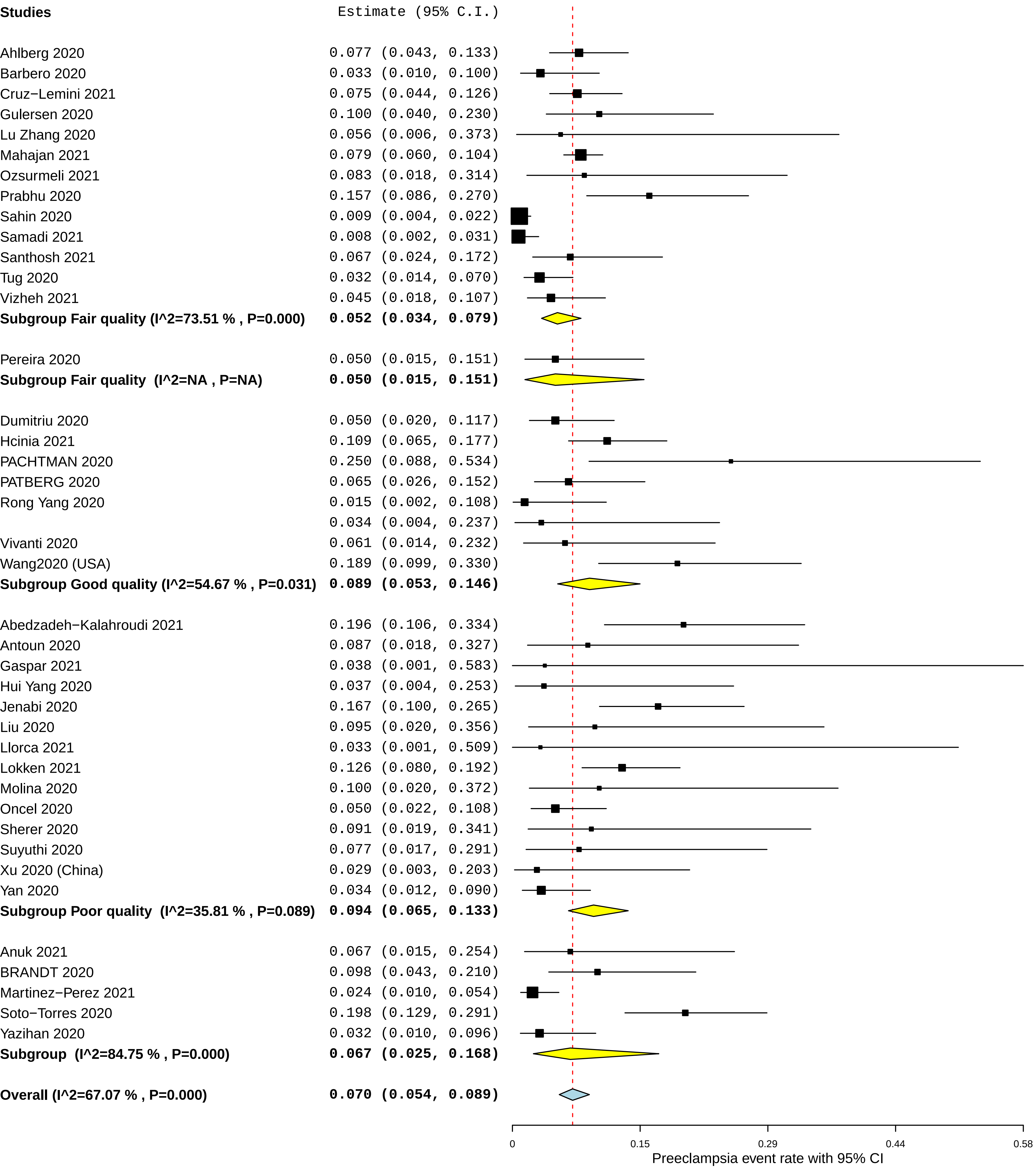
Studies
Estimate (95% C.I.)
Ahlberg 2020 0.077 (0.043, 0.133)
Barbero 2020 0.033 (0.010, 0.100)
Cruz−Lemini 2021 0.075 (0.044, 0.126)
Gulersen 2020 0.100 (0.040, 0.230)
Lu Zhang 2020 0.056 (0.006, 0.373)
Mahajan 2021 0.079 (0.060, 0.104)
Ozsurmeli 2021 0.083 (0.018, 0.314)
Prabhu 2020 0.157 (0.086, 0.270)
Sahin 2020 0.009 (0.004, 0.022)
Samadi 2021 0.008 (0.002, 0.031)
Santhosh 2021 0.067 (0.024, 0.172)
Tug 2020 0.032 (0.014, 0.070)
Vizheh 2021 0.045 (0.018, 0.107)
Subgroup Fair quality (I^2=73.51 % , P=0.000) 0.052 (0.034, 0.079)
Pereira 2020 0.050 (0.015, 0.151)
Subgroup Fair quality (I^2=NA , P=NA) 0.050 (0.015, 0.151)
Dumitriu 2020 0.050 (0.020, 0.117)
Hcinia 2021 0.109 (0.065, 0.177)
PACHTMAN 2020 0.250 (0.088, 0.534)
PATBERG 2020 0.065 (0.026, 0.152)
Rong Yang 2020 0.015 (0.002, 0.108)
0.034 (0.004, 0.237)
Vivanti 2020 0.061 (0.014, 0.232)
Wang2020 (USA) 0.189 (0.099, 0.330)
Subgroup Good quality (I^2=54.67 % , P=0.031) 0.089 (0.053, 0.146)
Abedzadeh−Kalahroudi 2021 0.196 (0.106, 0.334)
Antoun 2020 0.087 (0.018, 0.327)
Gaspar 2021 0.038 (0.001, 0.583)
Hui Yang 2020 0.037 (0.004, 0.253)
Jenabi 2020 0.167 (0.100, 0.265)
Liu 2020 0.095 (0.020, 0.356)
Llorca 2021 0.033 (0.001, 0.509)
Lokken 2021 0.126 (0.080, 0.192)
Molina 2020 0.100 (0.020, 0.372)
Oncel 2020 0.050 (0.022, 0.108)
Sherer 2020 0.091 (0.019, 0.341)
Suyuthi 2020 0.077 (0.017, 0.291)
Xu 2020 (China) 0.029 (0.003, 0.203)
Yan 2020 0.034 (0.012, 0.090)
Subgroup Poor quality (I^2=35.81 % , P=0.089) 0.094 (0.065, 0.133)
Anuk 2021 0.067 (0.015, 0.254)
BRANDT 2020 0.098 (0.043, 0.210)
Martinez−Perez 2021 0.024 (0.010, 0.054)
Soto−Torres 2020 0.198 (0.129, 0.291)
Yazihan 2020 0.032 (0.010, 0.096)
Subgroup (I^2=84.75 % , P=0.000) 0.067 (0.025, 0.168)
Overall (I^2=67.07 % , P=0.000) 0.070 (0.054, 0.089)
0
0.15
0.29
0.44
0.58
Preeclampsia event rate with 95% CI

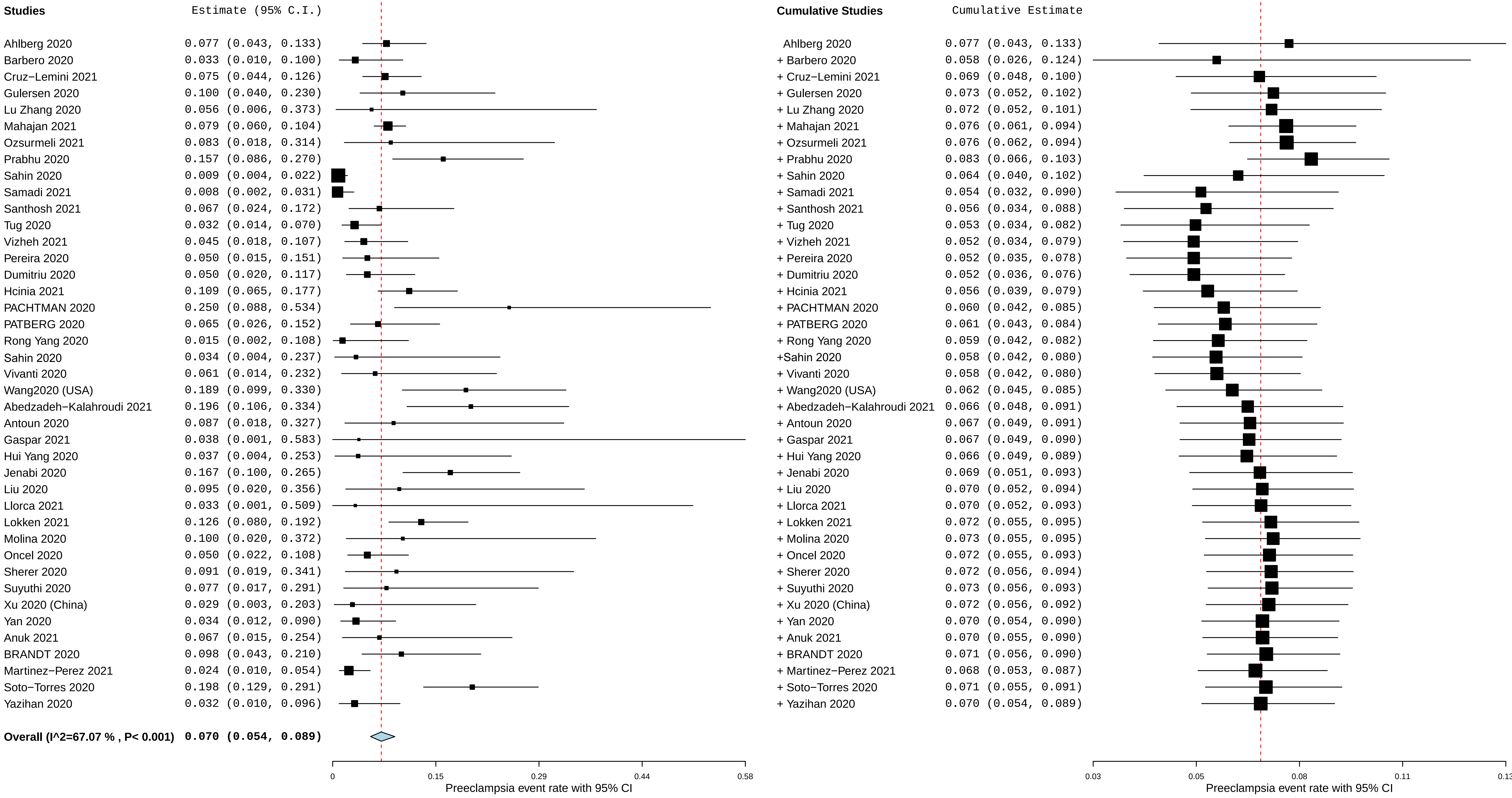

Studies
Estimate (95% C.I.)
Ahlberg 2020 0.077 (0.043, 0.133)
Barbero 2020 0.033 (0.010, 0.100)
Cruz−Lemini 2021 0.075 (0.044, 0.126)
Gulersen 2020 0.100 (0.040, 0.230)
Lu Zhang 2020 0.056 (0.006, 0.373)
Mahajan 2021 0.079 (0.060, 0.104)
Ozsurmeli 2021 0.083 (0.018, 0.314)
Prabhu 2020 0.157 (0.086, 0.270)
Sahin 2020 0.009 (0.004, 0.022)
Samadi 2021 0.008 (0.002, 0.031)
Santhosh 2021 0.067 (0.024, 0.172)
Tug 2020 0.032 (0.014, 0.070)
Vizheh 2021 0.045 (0.018, 0.107)
Pereira 2020 0.050 (0.015, 0.151)
Dumitriu 2020 0.050 (0.020, 0.117)
Hcinia 2021 0.109 (0.065, 0.177)
PACHTMAN 2020 0.250 (0.088, 0.534)
PATBERG 2020 0.065 (0.026, 0.152)
Rong Yang 2020 0.015 (0.002, 0.108)
Sahin 2020 0.034 (0.004, 0.237)
Vivanti 2020 0.061 (0.014, 0.232)
Wang2020 (USA) 0.189 (0.099, 0.330)
Abedzadeh−Kalahroudi 2021 0.196 (0.106, 0.334)
Antoun 2020 0.087 (0.018, 0.327)
Gaspar 2021 0.038 (0.001, 0.583)
Hui Yang 2020 0.037 (0.004, 0.253)
Jenabi 2020 0.167 (0.100, 0.265)
Liu 2020 0.095 (0.020, 0.356)
Llorca 2021 0.033 (0.001, 0.509)
Lokken 2021 0.126 (0.080, 0.192)
Molina 2020 0.100 (0.020, 0.372)
Oncel 2020 0.050 (0.022, 0.108)
Sherer 2020 0.091 (0.019, 0.341)
Suyuthi 2020 0.077 (0.017, 0.291)
Xu 2020 (China) 0.029 (0.003, 0.203)
Yan 2020 0.034 (0.012, 0.090)
Anuk 2021 0.067 (0.015, 0.254)
BRANDT 2020 0.098 (0.043, 0.210)
Martinez−Perez 2021 0.024 (0.010, 0.054)
Soto−Torres 2020 0.198 (0.129, 0.291)
Yazihan 2020 0.032 (0.010, 0.096)
Overall (I^2=67.07 % , P< 0.001) 0.070 (0.054, 0.089)
0
0.15
0.29
0.44
0.58
Preeclampsia event rate with 95% CI
Cumulative Studies
Cumulative Estimate
Ahlberg 2020 0.077 (0.043, 0.133)
+ Barbero 2020 0.058 (0.026, 0.124)
+ Cruz−Lemini 2021 0.069 (0.048, 0.100)
+ Gulersen 2020 0.073 (0.052, 0.102)
+ Lu Zhang 2020 0.072 (0.052, 0.101)
+ Mahajan 2021 0.076 (0.061, 0.094)
+ Ozsurmeli 2021 0.076 (0.062, 0.094)
+ Prabhu 2020 0.083 (0.066, 0.103)
+ Sahin 2020 0.064 (0.040, 0.102)
+ Samadi 2021 0.054 (0.032, 0.090)
+ Santhosh 2021 0.056 (0.034, 0.088)
+ Tug 2020 0.053 (0.034, 0.082)
+ Vizheh 2021 0.052 (0.034, 0.079)
+ Pereira 2020 0.052 (0.035, 0.078)
+ Dumitriu 2020 0.052 (0.036, 0.076)
+ Hcinia 2021 0.056 (0.039, 0.079)
+ PACHTMAN 2020 0.060 (0.042, 0.085)
+ PATBERG 2020 0.061 (0.043, 0.084)
+ Rong Yang 2020 0.059 (0.042, 0.082)
+Sahin 2020 0.058 (0.042, 0.080)
+ Vivanti 2020 0.058 (0.042, 0.080)
+ Wang2020 (USA) 0.062 (0.045, 0.085)
+ Abedzadeh−Kalahroudi 2021 0.066 (0.048, 0.091)
+ Antoun 2020 0.067 (0.049, 0.091)
+ Gaspar 2021 0.067 (0.049, 0.090)
+ Hui Yang 2020 0.066 (0.049, 0.089)
+ Jenabi 2020 0.069 (0.051, 0.093)
+ Liu 2020 0.070 (0.052, 0.094)
+ Llorca 2021 0.070 (0.052, 0.093)
+ Lokken 2021 0.072 (0.055, 0.095)
+ Molina 2020 0.073 (0.055, 0.095)
+ Oncel 2020 0.072 (0.055, 0.093)
+ Sherer 2020 0.072 (0.056, 0.094)
+ Suyuthi 2020 0.073 (0.056, 0.093)
+ Xu 2020 (China) 0.072 (0.056, 0.092)
+ Yan 2020 0.070 (0.054, 0.090)
+ Anuk 2021 0.070 (0.055, 0.090)
+ BRANDT 2020 0.071 (0.056, 0.090)
+ Martinez−Perez 2021 0.068 (0.053, 0.087)
+ Soto−Torres 2020 0.071 (0.055, 0.091)
+ Yazihan 2020 0.070 (0.054, 0.089)
0.03
0.05
0.08
0.11
0.13
Preeclampsia event rate with 95% CI

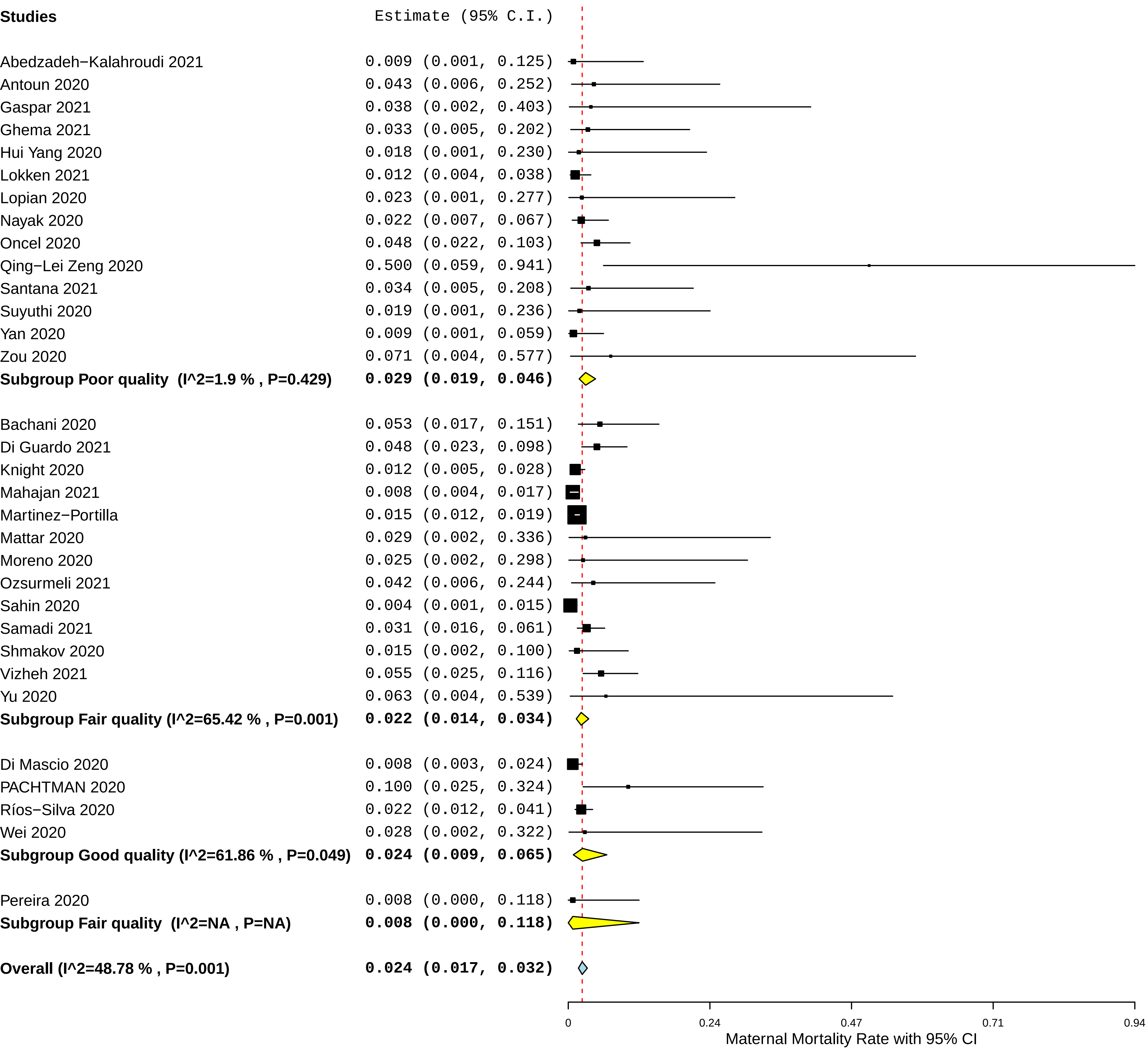
Studies
Estimate (95% C.I.)
Abedzadeh−Kalahroudi 2021 0.009 (0.001, 0.125)
Antoun 2020 0.043 (0.006, 0.252)
Gaspar 2021 0.038 (0.002, 0.403)
Ghema 2021 0.033 (0.005, 0.202)
Hui Yang 2020 0.018 (0.001, 0.230)
Lokken 2021 0.012 (0.004, 0.038)
Lopian 2020 0.023 (0.001, 0.277)
Nayak 2020 0.022 (0.007, 0.067)
Oncel 2020 0.048 (0.022, 0.103)
Qing−Lei Zeng 2020 0.500 (0.059, 0.941)
Santana 2021 0.034 (0.005, 0.208)
Suyuthi 2020 0.019 (0.001, 0.236)
Yan 2020 0.009 (0.001, 0.059)
Zou 2020 0.071 (0.004, 0.577)
Subgroup Poor quality (I^2=1.9 % , P=0.429) 0.029 (0.019, 0.046)
Bachani 2020 0.053 (0.017, 0.151)
Di Guardo 2021 0.048 (0.023, 0.098)
Knight 2020 0.012 (0.005, 0.028)
Mahajan 2021 0.008 (0.004, 0.017)
Martinez−Portilla 0.015 (0.012, 0.019)
Mattar 2020 0.029 (0.002, 0.336)
Moreno 2020 0.025 (0.002, 0.298)
Ozsurmeli 2021 0.042 (0.006, 0.244)
Sahin 2020 0.004 (0.001, 0.015)
Samadi 2021 0.031 (0.016, 0.061)
Shmakov 2020 0.015 (0.002, 0.100)
Vizheh 2021 0.055 (0.025, 0.116)
Yu 2020 0.063 (0.004, 0.539)
Subgroup Fair quality (I^2=65.42 % , P=0.001) 0.022 (0.014, 0.034)
Di Mascio 2020 0.008 (0.003, 0.024)
PACHTMAN 2020 0.100 (0.025, 0.324)
Ríos−Silva 2020 0.022 (0.012, 0.041)
Wei 2020 0.028 (0.002, 0.322)
Subgroup Good quality (I^2=61.86 % , P=0.049) 0.024 (0.009, 0.065)
Pereira 2020 0.008 (0.000, 0.118)
Subgroup Fair quality (I^2=NA , P=NA) 0.008 (0.000, 0.118)
Overall (I^2=48.78 % , P=0.001) 0.024 (0.017, 0.032)
0
0.24
0.47
0.71
0.94
Maternal Mortality Rate with 95% CI

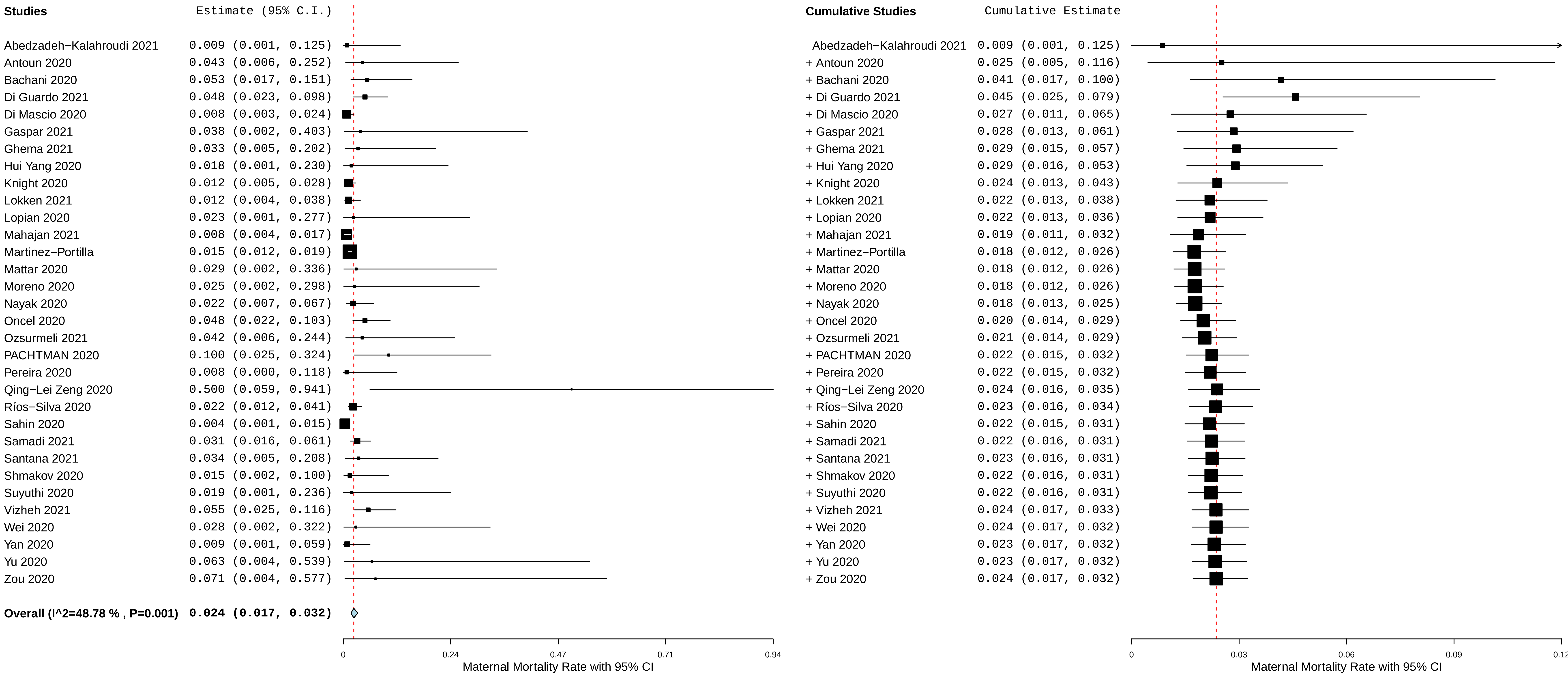
Studies
Estimate (95% C.I.)
Abedzadeh−Kalahroudi 2021 0.009 (0.001, 0.125)
Antoun 2020 0.043 (0.006, 0.252)
Bachani 2020 0.053 (0.017, 0.151)
Di Guardo 2021 0.048 (0.023, 0.098)
Di Mascio 2020 0.008 (0.003, 0.024)
Gaspar 2021 0.038 (0.002, 0.403)
Ghema 2021 0.033 (0.005, 0.202)
Hui Yang 2020 0.018 (0.001, 0.230)
Knight 2020 0.012 (0.005, 0.028)
Lokken 2021 0.012 (0.004, 0.038)
Lopian 2020 0.023 (0.001, 0.277)
Mahajan 2021 0.008 (0.004, 0.017)
Martinez−Portilla 0.015 (0.012, 0.019)
Mattar 2020 0.029 (0.002, 0.336)
Moreno 2020 0.025 (0.002, 0.298)
Nayak 2020 0.022 (0.007, 0.067)
Oncel 2020 0.048 (0.022, 0.103)
Ozsurmeli 2021 0.042 (0.006, 0.244)
PACHTMAN 2020 0.100 (0.025, 0.324)
Pereira 2020 0.008 (0.000, 0.118)
Qing−Lei Zeng 2020 0.500 (0.059, 0.941)
Ríos−Silva 2020 0.022 (0.012, 0.041)
Sahin 2020 0.004 (0.001, 0.015)
Samadi 2021 0.031 (0.016, 0.061)
Santana 2021 0.034 (0.005, 0.208)
Shmakov 2020 0.015 (0.002, 0.100)
Suyuthi 2020 0.019 (0.001, 0.236)
Vizheh 2021 0.055 (0.025, 0.116)
Wei 2020 0.028 (0.002, 0.322)
Yan 2020 0.009 (0.001, 0.059)
Yu 2020 0.063 (0.004, 0.539)
Zou 2020 0.071 (0.004, 0.577)
Overall (I^2=48.78 % , P=0.001) 0.024 (0.017, 0.032)
0
0.24
0.47
0.71
0.94
Maternal Mortality Rate with 95% CI
Cumulative Studies
Cumulative Estimate
Abedzadeh−Kalahroudi 2021 0.009 (0.001, 0.125)
+ Antoun 2020 0.025 (0.005, 0.116)
+ Bachani 2020 0.041 (0.017, 0.100)
+ Di Guardo 2021 0.045 (0.025, 0.079)
+ Di Mascio 2020 0.027 (0.011, 0.065)
+ Gaspar 2021 0.028 (0.013, 0.061)
+ Ghema 2021 0.029 (0.015, 0.057)
+ Hui Yang 2020 0.029 (0.016, 0.053)
+ Knight 2020 0.024 (0.013, 0.043)
+ Lokken 2021 0.022 (0.013, 0.038)
+ Lopian 2020 0.022 (0.013, 0.036)
+ Mahajan 2021 0.019 (0.011, 0.032)
+ Martinez−Portilla 0.018 (0.012, 0.026)
+ Mattar 2020 0.018 (0.012, 0.026)
+ Moreno 2020 0.018 (0.012, 0.026)
+ Nayak 2020 0.018 (0.013, 0.025)
+ Oncel 2020 0.020 (0.014, 0.029)
+ Ozsurmeli 2021 0.021 (0.014, 0.029)
+ PACHTMAN 2020 0.022 (0.015, 0.032)
+ Pereira 2020 0.022 (0.015, 0.032)
+ Qing−Lei Zeng 2020 0.024 (0.016, 0.035)
+ Ríos−Silva 2020 0.023 (0.016, 0.034)
+ Sahin 2020 0.022 (0.015, 0.031)
+ Samadi 2021 0.022 (0.016, 0.031)
+ Santana 2021 0.023 (0.016, 0.031)
+ Shmakov 2020 0.022 (0.016, 0.031)
+ Suyuthi 2020 0.022 (0.016, 0.031)
+ Vizheh 2021 0.024 (0.017, 0.033)
+ Wei 2020 0.024 (0.017, 0.032)
+ Yan 2020 0.023 (0.017, 0.032)
+ Yu 2020 0.023 (0.017, 0.032)
+ Zou 2020 0.024 (0.017, 0.032)
0
0.03
0.06
0.09
0.12
Maternal Mortality Rate with 95% CI

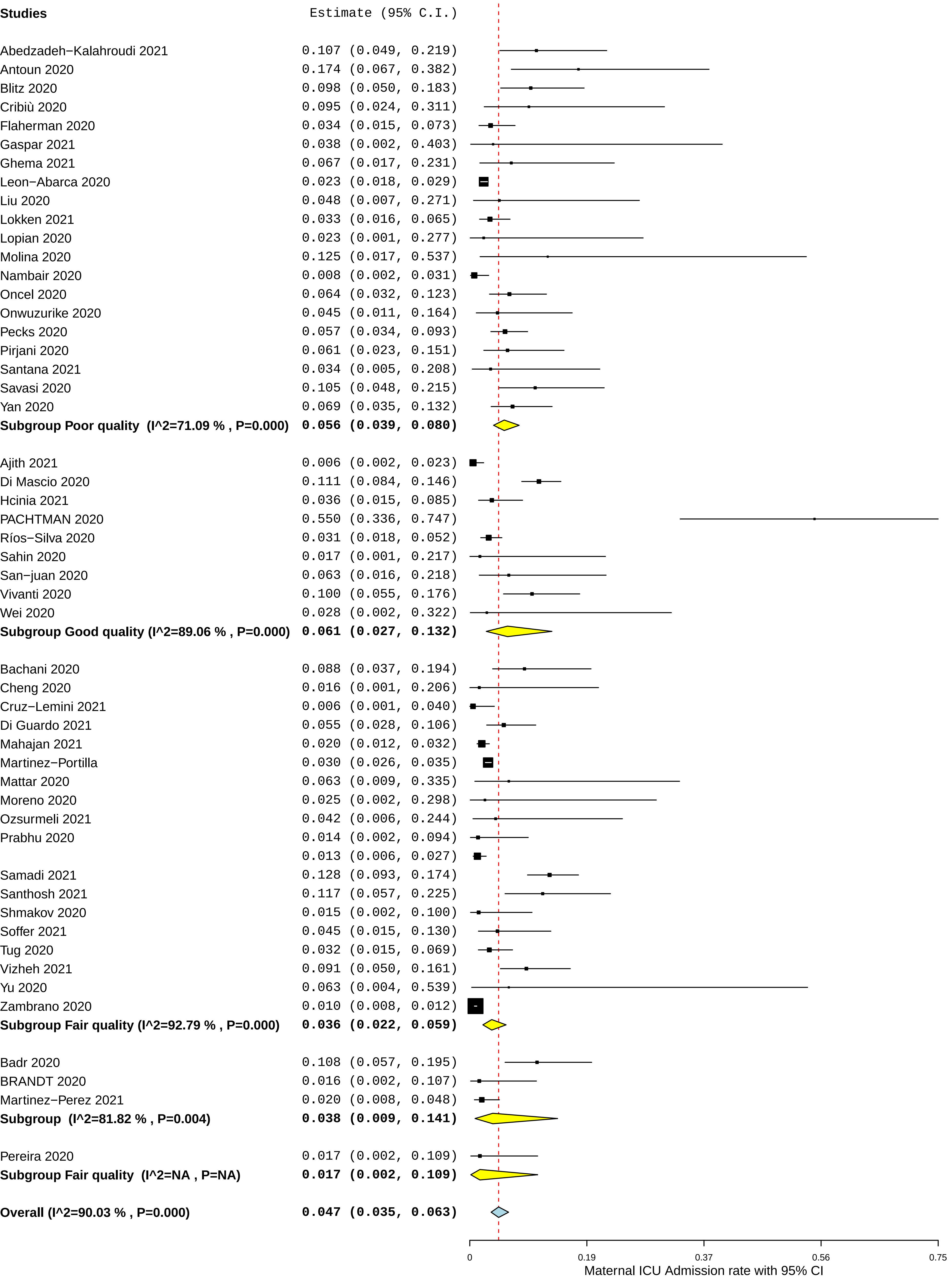

Studies
Estimate (95% C.I.)
Abedzadeh−Kalahroudi 2021 0.107 (0.049, 0.219)
Antoun 2020 0.174 (0.067, 0.382)
Blitz 2020 0.098 (0.050, 0.183)
Cribiù 2020 0.095 (0.024, 0.311)
Flaherman 2020 0.034 (0.015, 0.073)
Gaspar 2021 0.038 (0.002, 0.403)
Ghema 2021 0.067 (0.017, 0.231)
Leon−Abarca 2020 0.023 (0.018, 0.029)
Liu 2020 0.048 (0.007, 0.271)
Lokken 2021 0.033 (0.016, 0.065)
Lopian 2020 0.023 (0.001, 0.277)
Molina 2020 0.125 (0.017, 0.537)
Nambair 2020 0.008 (0.002, 0.031)
Oncel 2020 0.064 (0.032, 0.123)
Onwuzurike 2020 0.045 (0.011, 0.164)
Pecks 2020 0.057 (0.034, 0.093)
Pirjani 2020 0.061 (0.023, 0.151)
Santana 2021 0.034 (0.005, 0.208)
Savasi 2020 0.105 (0.048, 0.215)
Yan 2020 0.069 (0.035, 0.132)
Subgroup Poor quality (I^2=71.09 % , P=0.000) 0.056 (0.039, 0.080)
Ajith 2021 0.006 (0.002, 0.023)
Di Mascio 2020 0.111 (0.084, 0.146)
Hcinia 2021 0.036 (0.015, 0.085)
PACHTMAN 2020 0.550 (0.336, 0.747)
Ríos−Silva 2020 0.031 (0.018, 0.052)
Sahin 2020 0.017 (0.001, 0.217)
San−juan 2020 0.063 (0.016, 0.218)
Vivanti 2020 0.100 (0.055, 0.176)
Wei 2020 0.028 (0.002, 0.322)
Subgroup Good quality (I^2=89.06 % , P=0.000) 0.061 (0.027, 0.132)
Bachani 2020 0.088 (0.037, 0.194)
Cheng 2020 0.016 (0.001, 0.206)
Cruz−Lemini 2021 0.006 (0.001, 0.040)
Di Guardo 2021 0.055 (0.028, 0.106)
Mahajan 2021 0.020 (0.012, 0.032)
Martinez−Portilla 0.030 (0.026, 0.035)
Mattar 2020 0.063 (0.009, 0.335)
Moreno 2020 0.025 (0.002, 0.298)
Ozsurmeli 2021 0.042 (0.006, 0.244)
Prabhu 2020 0.014 (0.002, 0.094)
0.013 (0.006, 0.027)
Samadi 2021 0.128 (0.093, 0.174)
Santhosh 2021 0.117 (0.057, 0.225)
Shmakov 2020 0.015 (0.002, 0.100)
Soffer 2021 0.045 (0.015, 0.130)
Tug 2020 0.032 (0.015, 0.069)
Vizheh 2021 0.091 (0.050, 0.161)
Yu 2020 0.063 (0.004, 0.539)
Zambrano 2020 0.010 (0.008, 0.012)
Subgroup Fair quality (I^2=92.79 % , P=0.000) 0.036 (0.022, 0.059)
Badr 2020 0.108 (0.057, 0.195)
BRANDT 2020 0.016 (0.002, 0.107)
Martinez−Perez 2021 0.020 (0.008, 0.048)
Subgroup (I^2=81.82 % , P=0.004) 0.038 (0.009, 0.141)
Pereira 2020 0.017 (0.002, 0.109)
Subgroup Fair quality (I^2=NA , P=NA) 0.017 (0.002, 0.109)
Overall (I^2=90.03 % , P=0.000) 0.047 (0.035, 0.063)
0
0.19
0.37
0.56
0.75
Maternal ICU Admission rate with 95% CI

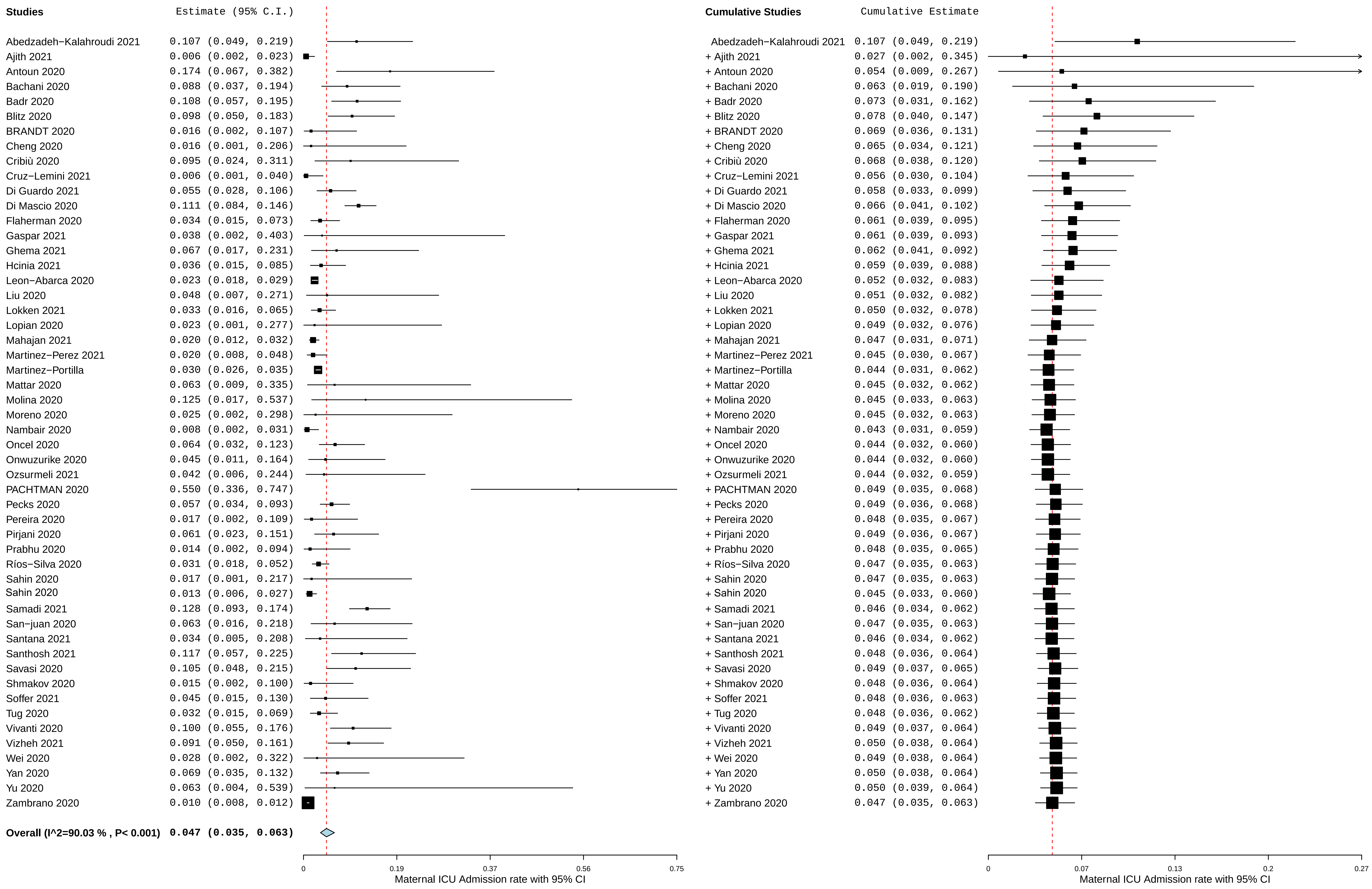
Studies
Estimate (95% C.I.)
Abedzadeh−Kalahroudi 2021 0.107 (0.049, 0.219)
Ajith 2021 0.006 (0.002, 0.023)
Antoun 2020 0.174 (0.067, 0.382)
Bachani 2020 0.088 (0.037, 0.194)
Badr 2020 0.108 (0.057, 0.195)
Blitz 2020 0.098 (0.050, 0.183)
BRANDT 2020 0.016 (0.002, 0.107)
Cheng 2020 0.016 (0.001, 0.206)
Cribiù 2020 0.095 (0.024, 0.311)
Cruz−Lemini 2021 0.006 (0.001, 0.040)
Di Guardo 2021 0.055 (0.028, 0.106)
Di Mascio 2020 0.111 (0.084, 0.146)
Flaherman 2020 0.034 (0.015, 0.073)
Gaspar 2021 0.038 (0.002, 0.403)
Ghema 2021 0.067 (0.017, 0.231)
Hcinia 2021 0.036 (0.015, 0.085)
Leon−Abarca 2020 0.023 (0.018, 0.029)
Liu 2020 0.048 (0.007, 0.271)
Lokken 2021 0.033 (0.016, 0.065)
Lopian 2020 0.023 (0.001, 0.277)
Mahajan 2021 0.020 (0.012, 0.032)
Martinez−Perez 2021 0.020 (0.008, 0.048)
Martinez−Portilla 0.030 (0.026, 0.035)
Mattar 2020 0.063 (0.009, 0.335)
Molina 2020 0.125 (0.017, 0.537)
Moreno 2020 0.025 (0.002, 0.298)
Nambair 2020 0.008 (0.002, 0.031)
Oncel 2020 0.064 (0.032, 0.123)
Onwuzurike 2020 0.045 (0.011, 0.164)
Ozsurmeli 2021 0.042 (0.006, 0.244)
PACHTMAN 2020 0.550 (0.336, 0.747)
Pecks 2020 0.057 (0.034, 0.093)
Pereira 2020 0.017 (0.002, 0.109)
Pirjani 2020 0.061 (0.023, 0.151)
Prabhu 2020 0.014 (0.002, 0.094)
Ríos−Silva 2020 0.031 (0.018, 0.052)
Sahin 2020 0.017 (0.001, 0.217)
Sahin 2020 0.013 (0.006, 0.027)
Samadi 2021 0.128 (0.093, 0.174)
San−juan 2020 0.063 (0.016, 0.218)
Santana 2021 0.034 (0.005, 0.208)
Santhosh 2021 0.117 (0.057, 0.225)
Savasi 2020 0.105 (0.048, 0.215)
Shmakov 2020 0.015 (0.002, 0.100)
Soffer 2021 0.045 (0.015, 0.130)
Tug 2020 0.032 (0.015, 0.069)
Vivanti 2020 0.100 (0.055, 0.176)
Vizheh 2021 0.091 (0.050, 0.161)
Wei 2020 0.028 (0.002, 0.322)
Yan 2020 0.069 (0.035, 0.132)
Yu 2020 0.063 (0.004, 0.539)
Zambrano 2020 0.010 (0.008, 0.012)
Overall (I^2=90.03 % , P< 0.001) 0.047 (0.035, 0.063)
0
0.19
0.37
0.56
0.75
Maternal ICU Admission rate with 95% CI
Cumulative Studies
Cumulative Estimate
Abedzadeh−Kalahroudi 2021 0.107 (0.049, 0.219)
+ Ajith 2021 0.027 (0.002, 0.345)
+ Antoun 2020 0.054 (0.009, 0.267)
+ Bachani 2020 0.063 (0.019, 0.190)
+ Badr 2020 0.073 (0.031, 0.162)
+ Blitz 2020 0.078 (0.040, 0.147)
+ BRANDT 2020 0.069 (0.036, 0.131)
+ Cheng 2020 0.065 (0.034, 0.121)
+ Cribiù 2020 0.068 (0.038, 0.120)
+ Cruz−Lemini 2021 0.056 (0.030, 0.104)
+ Di Guardo 2021 0.058 (0.033, 0.099)
+ Di Mascio 2020 0.066 (0.041, 0.102)
+ Flaherman 2020 0.061 (0.039, 0.095)
+ Gaspar 2021 0.061 (0.039, 0.093)
+ Ghema 2021 0.062 (0.041, 0.092)
+ Hcinia 2021 0.059 (0.039, 0.088)
+ Leon−Abarca 2020 0.052 (0.032, 0.083)
+ Liu 2020 0.051 (0.032, 0.082)
+ Lokken 2021 0.050 (0.032, 0.078)
+ Lopian 2020 0.049 (0.032, 0.076)
+ Mahajan 2021 0.047 (0.031, 0.071)
+ Martinez−Perez 2021 0.045 (0.030, 0.067)
+ Martinez−Portilla 0.044 (0.031, 0.062)
+ Mattar 2020 0.045 (0.032, 0.062)
+ Molina 2020 0.045 (0.033, 0.063)
+ Moreno 2020 0.045 (0.032, 0.063)
+ Nambair 2020 0.043 (0.031, 0.059)
+ Oncel 2020 0.044 (0.032, 0.060)
+ Onwuzurike 2020 0.044 (0.032, 0.060)
+ Ozsurmeli 2021 0.044 (0.032, 0.059)
+ PACHTMAN 2020 0.049 (0.035, 0.068)
+ Pecks 2020 0.049 (0.036, 0.068)
+ Pereira 2020 0.048 (0.035, 0.067)
+ Pirjani 2020 0.049 (0.036, 0.067)
+ Prabhu 2020 0.048 (0.035, 0.065)
+ Ríos−Silva 2020 0.047 (0.035, 0.063)
+ Sahin 2020 0.047 (0.035, 0.063)
+ Sahin 2020 0.045 (0.033, 0.060)
+ Samadi 2021 0.046 (0.034, 0.062)
+ San−juan 2020 0.047 (0.035, 0.063)
+ Santana 2021 0.046 (0.034, 0.062)
+ Santhosh 2021 0.048 (0.036, 0.064)
+ Savasi 2020 0.049 (0.037, 0.065)
+ Shmakov 2020 0.048 (0.036, 0.064)
+ Soffer 2021 0.048 (0.036, 0.063)
+ Tug 2020 0.048 (0.036, 0.062)
+ Vivanti 2020 0.049 (0.037, 0.064)
+ Vizheh 2021 0.050 (0.038, 0.064)
+ Wei 2020 0.049 (0.038, 0.064)
+ Yan 2020 0.050 (0.038, 0.064)
+ Yu 2020 0.050 (0.039, 0.064)
+ Zambrano 2020 0.047 (0.035, 0.063)
0
0.07
0.13
0.2
0.27
Maternal ICU Admission rate with 95% CI

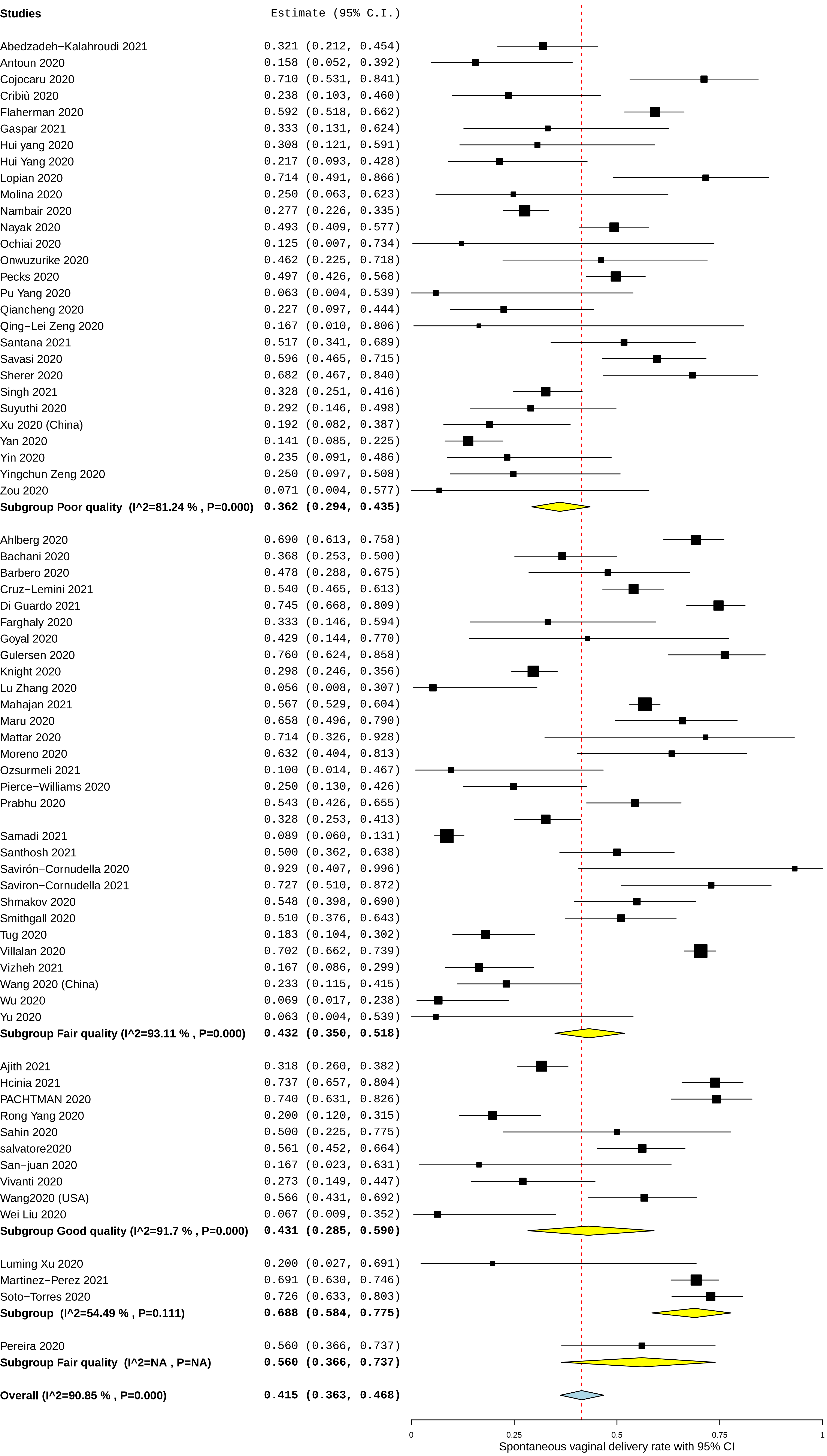
Studies
Estimate (95% C.I.)
Abedzadeh−Kalahroudi 2021 0.321 (0.212, 0.454)
Antoun 2020 0.158 (0.052, 0.392)
Cojocaru 2020 0.710 (0.531, 0.841)
Cribiù 2020 0.238 (0.103, 0.460)
Flaherman 2020 0.592 (0.518, 0.662)
Gaspar 2021 0.333 (0.131, 0.624)
Hui yang 2020 0.308 (0.121, 0.591)
Hui Yang 2020 0.217 (0.093, 0.428)
Lopian 2020 0.714 (0.491, 0.866)
Molina 2020 0.250 (0.063, 0.623)
Nambair 2020 0.277 (0.226, 0.335)
Nayak 2020 0.493 (0.409, 0.577)
Ochiai 2020 0.125 (0.007, 0.734)
Onwuzurike 2020 0.462 (0.225, 0.718)
Pecks 2020 0.497 (0.426, 0.568)
Pu Yang 2020 0.063 (0.004, 0.539)
Qiancheng 2020 0.227 (0.097, 0.444)
Qing−Lei Zeng 2020 0.167 (0.010, 0.806)
Santana 2021 0.517 (0.341, 0.689)
Savasi 2020 0.596 (0.465, 0.715)
Sherer 2020 0.682 (0.467, 0.840)
Singh 2021 0.328 (0.251, 0.416)
Suyuthi 2020 0.292 (0.146, 0.498)
Xu 2020 (China) 0.192 (0.082, 0.387)
Yan 2020 0.141 (0.085, 0.225)
Yin 2020 0.235 (0.091, 0.486)
Yingchun Zeng 2020 0.250 (0.097, 0.508)
Zou 2020 0.071 (0.004, 0.577)
Subgroup Poor quality (I^2=81.24 % , P=0.000) 0.362 (0.294, 0.435)
Ahlberg 2020 0.690 (0.613, 0.758)
Bachani 2020 0.368 (0.253, 0.500)
Barbero 2020 0.478 (0.288, 0.675)
Cruz−Lemini 2021 0.540 (0.465, 0.613)
Di Guardo 2021 0.745 (0.668, 0.809)
Farghaly 2020 0.333 (0.146, 0.594)
Goyal 2020 0.429 (0.144, 0.770)
Gulersen 2020 0.760 (0.624, 0.858)
Knight 2020 0.298 (0.246, 0.356)
Lu Zhang 2020 0.056 (0.008, 0.307)
Mahajan 2021 0.567 (0.529, 0.604)
Maru 2020 0.658 (0.496, 0.790)
Mattar 2020 0.714 (0.326, 0.928)
Moreno 2020 0.632 (0.404, 0.813)
Ozsurmeli 2021 0.100 (0.014, 0.467)
Pierce−Williams 2020 0.250 (0.130, 0.426)
Prabhu 2020 0.543 (0.426, 0.655)
0.328 (0.253, 0.413)
Samadi 2021 0.089 (0.060, 0.131)
Santhosh 2021 0.500 (0.362, 0.638)
Savirón−Cornudella 2020 0.929 (0.407, 0.996)
Saviron−Cornudella 2021 0.727 (0.510, 0.872)
Shmakov 2020 0.548 (0.398, 0.690)
Smithgall 2020 0.510 (0.376, 0.643)
Tug 2020 0.183 (0.104, 0.302)
Villalan 2020 0.702 (0.662, 0.739)
Vizheh 2021 0.167 (0.086, 0.299)
Wang 2020 (China) 0.233 (0.115, 0.415)
Wu 2020 0.069 (0.017, 0.238)
Yu 2020 0.063 (0.004, 0.539)
Subgroup Fair quality (I^2=93.11 % , P=0.000) 0.432 (0.350, 0.518)
Ajith 2021 0.318 (0.260, 0.382)
Hcinia 2021 0.737 (0.657, 0.804)
PACHTMAN 2020 0.740 (0.631, 0.826)
Rong Yang 2020 0.200 (0.120, 0.315)
Sahin 2020 0.500 (0.225, 0.775)
salvatore2020 0.561 (0.452, 0.664)
San-juan 2020 0.167 (0.023, 0.631)
Vivanti 2020 0.273 (0.149, 0.447)
Wang2020 (USA) 0.566 (0.431, 0.692)
Wei Liu 2020 0.067 (0.009, 0.352)
Subgroup Good quality (I^2=91.7 % , P=0.000) 0.431 (0.285, 0.590)
Luming Xu 2020 0.200 (0.027, 0.691)
Martinez−Perez 2021 0.691 (0.630, 0.746)
Soto−Torres 2020 0.726 (0.633, 0.803)
Subgroup (I^2=54.49 % , P=0.111) 0.688 (0.584, 0.775)
Pereira 2020 0.560 (0.366, 0.737)
Subgroup Fair quality (I^2=NA , P=NA) 0.560 (0.366, 0.737)
Overall (I^2=90.85 % , P=0.000) 0.415 (0.363, 0.468)
0
0.25
0.5
0.75
1
Spontaneous vaginal delivery rate with 95% CI

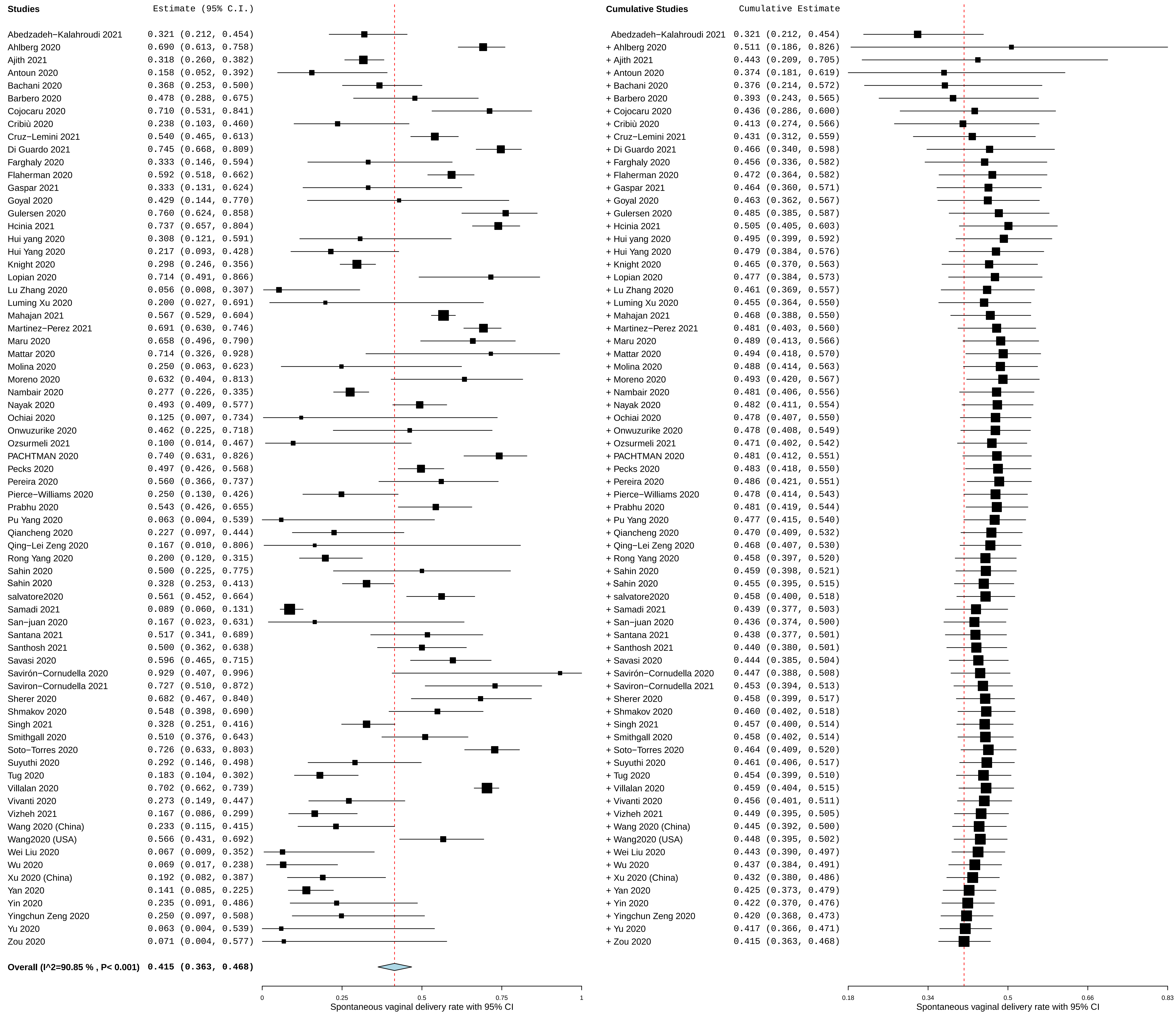
Studies
Estimate (95% C.I.)
Abedzadeh−Kalahroudi 2021 0.321 (0.212, 0.454)
Ahlberg 2020 0.690 (0.613, 0.758)
Ajith 2021 0.318 (0.260, 0.382)
Antoun 2020 0.158 (0.052, 0.392)
Bachani 2020 0.368 (0.253, 0.500)
Barbero 2020 0.478 (0.288, 0.675)
Cojocaru 2020 0.710 (0.531, 0.841)
Cribiù 2020 0.238 (0.103, 0.460)
Cruz−Lemini 2021 0.540 (0.465, 0.613)
Di Guardo 2021 0.745 (0.668, 0.809)
Farghaly 2020 0.333 (0.146, 0.594)
Flaherman 2020 0.592 (0.518, 0.662)
Gaspar 2021 0.333 (0.131, 0.624)
Goyal 2020 0.429 (0.144, 0.770)
Gulersen 2020 0.760 (0.624, 0.858)
Hcinia 2021 0.737 (0.657, 0.804)
Hui yang 2020 0.308 (0.121, 0.591)
Hui Yang 2020 0.217 (0.093, 0.428)
Knight 2020 0.298 (0.246, 0.356)
Lopian 2020 0.714 (0.491, 0.866)
Lu Zhang 2020 0.056 (0.008, 0.307)
Luming Xu 2020 0.200 (0.027, 0.691)
Mahajan 2021 0.567 (0.529, 0.604)
Martinez−Perez 2021 0.691 (0.630, 0.746)
Maru 2020 0.658 (0.496, 0.790)
Mattar 2020 0.714 (0.326, 0.928)
Molina 2020 0.250 (0.063, 0.623)
Moreno 2020 0.632 (0.404, 0.813)
Nambair 2020 0.277 (0.226, 0.335)
Nayak 2020 0.493 (0.409, 0.577)
Ochiai 2020 0.125 (0.007, 0.734)
Onwuzurike 2020 0.462 (0.225, 0.718)
Ozsurmeli 2021 0.100 (0.014, 0.467)
PACHTMAN 2020 0.740 (0.631, 0.826)
Pecks 2020 0.497 (0.426, 0.568)
Pereira 2020 0.560 (0.366, 0.737)
Pierce−Williams 2020 0.250 (0.130, 0.426)
Prabhu 2020 0.543 (0.426, 0.655)
Pu Yang 2020 0.063 (0.004, 0.539)
Qiancheng 2020 0.227 (0.097, 0.444)
Qing−Lei Zeng 2020 0.167 (0.010, 0.806)
Rong Yang 2020 0.200 (0.120, 0.315)
Sahin 2020 0.500 (0.225, 0.775)
Sahin 2020 0.328 (0.253, 0.413)
salvatore2020 0.561 (0.452, 0.664)
Samadi 2021 0.089 (0.060, 0.131)
San−juan 2020 0.167 (0.023, 0.631)
Santana 2021 0.517 (0.341, 0.689)
Santhosh 2021 0.500 (0.362, 0.638)
Savasi 2020 0.596 (0.465, 0.715)
Savirón−Cornudella 2020 0.929 (0.407, 0.996)
Saviron−Cornudella 2021 0.727 (0.510, 0.872)
Sherer 2020 0.682 (0.467, 0.840)
Shmakov 2020 0.548 (0.398, 0.690)
Singh 2021 0.328 (0.251, 0.416)
Smithgall 2020 0.510 (0.376, 0.643)
Soto−Torres 2020 0.726 (0.633, 0.803)
Suyuthi 2020 0.292 (0.146, 0.498)
Tug 2020 0.183 (0.104, 0.302)
Villalan 2020 0.702 (0.662, 0.739)
Vivanti 2020 0.273 (0.149, 0.447)
Vizheh 2021 0.167 (0.086, 0.299)
Wang 2020 (China) 0.233 (0.115, 0.415)
Wang2020 (USA) 0.566 (0.431, 0.692)
Wei Liu 2020 0.067 (0.009, 0.352)
Wu 2020 0.069 (0.017, 0.238)
Xu 2020 (China) 0.192 (0.082, 0.387)
Yan 2020 0.141 (0.085, 0.225)
Yin 2020 0.235 (0.091, 0.486)
Yingchun Zeng 2020 0.250 (0.097, 0.508)
Yu 2020 0.063 (0.004, 0.539)
Zou 2020 0.071 (0.004, 0.577)
Overall (I^2=90.85 % , P< 0.001) 0.415 (0.363, 0.468)
0
0.25
0.5
0.75
1
Spontaneous vaginal delivery rate with 95% CI
Cumulative Studies
Cumulative Estimate
Abedzadeh−Kalahroudi 2021 0.321 (0.212, 0.454)
+ Ahlberg 2020 0.511 (0.186, 0.826)
+ Ajith 2021 0.443 (0.209, 0.705)
+ Antoun 2020 0.374 (0.181, 0.619)
+ Bachani 2020 0.376 (0.214, 0.572)
+ Barbero 2020 0.393 (0.243, 0.565)
+ Cojocaru 2020 0.436 (0.286, 0.600)
+ Cribiù 2020 0.413 (0.274, 0.566)
+ Cruz−Lemini 2021 0.431 (0.312, 0.559)
+ Di Guardo 2021 0.466 (0.340, 0.598)
+ Farghaly 2020 0.456 (0.336, 0.582)
+ Flaherman 2020 0.472 (0.364, 0.582)
+ Gaspar 2021 0.464 (0.360, 0.571)
+ Goyal 2020 0.463 (0.362, 0.567)
+ Gulersen 2020 0.485 (0.385, 0.587)
+ Hcinia 2021 0.505 (0.405, 0.603)
+ Hui yang 2020 0.495 (0.399, 0.592)
+ Hui Yang 2020 0.479 (0.384, 0.576)
+ Knight 2020 0.465 (0.370, 0.563)
+ Lopian 2020 0.477 (0.384, 0.573)
+ Lu Zhang 2020 0.461 (0.369, 0.557)
+ Luming Xu 2020 0.455 (0.364, 0.550)
+ Mahajan 2021 0.468 (0.388, 0.550)
+ Martinez−Perez 2021 0.481 (0.403, 0.560)
+ Maru 2020 0.489 (0.413, 0.566)
+ Mattar 2020 0.494 (0.418, 0.570)
+ Molina 2020 0.488 (0.414, 0.563)
+ Moreno 2020 0.493 (0.420, 0.567)
+ Nambair 2020 0.481 (0.406, 0.556)
+ Nayak 2020 0.482 (0.411, 0.554)
+ Ochiai 2020 0.478 (0.407, 0.550)
+ Onwuzurike 2020 0.478 (0.408, 0.549)
+ Ozsurmeli 2021 0.471 (0.402, 0.542)
+ PACHTMAN 2020 0.481 (0.412, 0.551)
+ Pecks 2020 0.483 (0.418, 0.550)
+ Pereira 2020 0.486 (0.421, 0.551)
+ Pierce−Williams 2020 0.478 (0.414, 0.543)
+ Prabhu 2020 0.481 (0.419, 0.544)
+ Pu Yang 2020 0.477 (0.415, 0.540)
+ Qiancheng 2020 0.470 (0.409, 0.532)
+ Qing−Lei Zeng 2020 0.468 (0.407, 0.530)
+ Rong Yang 2020 0.458 (0.397, 0.520)
+ Sahin 2020 0.459 (0.398, 0.521)
+ Sahin 2020 0.455 (0.395, 0.515)
+ salvatore2020 0.458 (0.400, 0.518)
+ Samadi 2021 0.439 (0.377, 0.503)
+ San−juan 2020 0.436 (0.374, 0.500)
+ Santana 2021 0.438 (0.377, 0.501)
+ Santhosh 2021 0.440 (0.380, 0.501)
+ Savasi 2020 0.444 (0.385, 0.504)
+ Savirón−Cornudella 2020 0.447 (0.388, 0.508)
+ Saviron−Cornudella 2021 0.453 (0.394, 0.513)
+ Sherer 2020 0.458 (0.399, 0.517)
+ Shmakov 2020 0.460 (0.402, 0.518)
+ Singh 2021 0.457 (0.400, 0.514)
+ Smithgall 2020 0.458 (0.402, 0.514)
+ Soto−Torres 2020 0.464 (0.409, 0.520)
+ Suyuthi 2020 0.461 (0.406, 0.517)
+ Tug 2020 0.454 (0.399, 0.510)
+ Villalan 2020 0.459 (0.404, 0.515)
+ Vivanti 2020 0.456 (0.401, 0.511)
+ Vizheh 2021 0.449 (0.395, 0.505)
+ Wang 2020 (China) 0.445 (0.392, 0.500)
+ Wang2020 (USA) 0.448 (0.395, 0.502)
+ Wei Liu 2020 0.443 (0.390, 0.497)
+ Wu 2020 0.437 (0.384, 0.491)
+ Xu 2020 (China) 0.432 (0.380, 0.486)
+ Yan 2020 0.425 (0.373, 0.479)
+ Yin 2020 0.422 (0.370, 0.476)
+ Yingchun Zeng 2020 0.420 (0.368, 0.473)
+ Yu 2020 0.417 (0.366, 0.471)
+ Zou 2020 0.415 (0.363, 0.468)
0.18
0.34
0.5
0.66
0.83
Spontaneous vaginal delivery rate with 95% CI

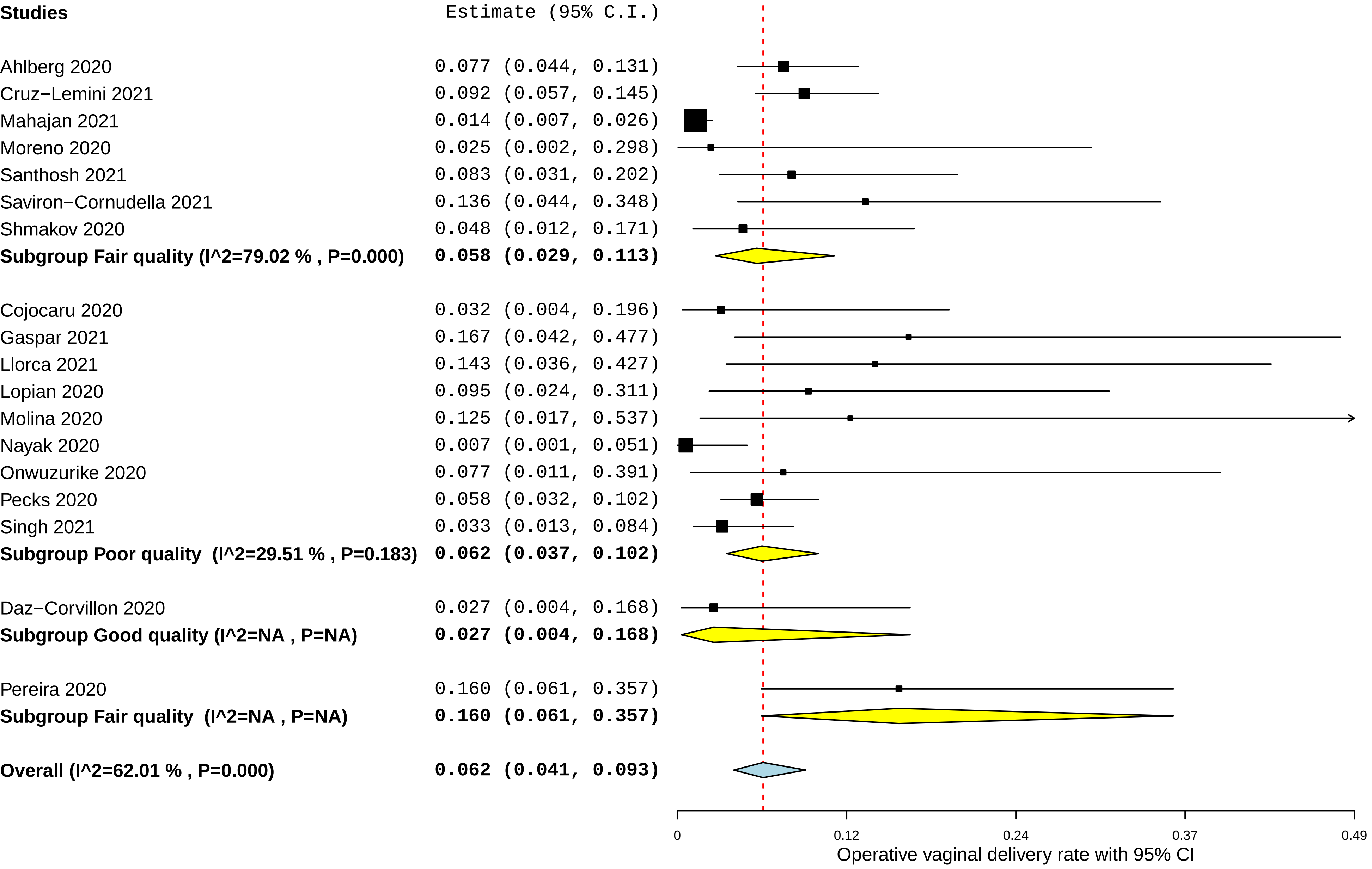
Studies
Estimate (95% C.I.)
Ahlberg 2020 0.077 (0.044, 0.131)
Cruz−Lemini 2021 0.092 (0.057, 0.145)
Mahajan 2021 0.014 (0.007, 0.026)
Moreno 2020 0.025 (0.002, 0.298)
Santhosh 2021 0.083 (0.031, 0.202)
Saviron−Cornudella 2021 0.136 (0.044, 0.348)
Shmakov 2020 0.048 (0.012, 0.171)
Subgroup Fair quality (I^2=79.02 % , P=0.000) 0.058 (0.029, 0.113)
Cojocaru 2020 0.032 (0.004, 0.196)
Gaspar 2021 0.167 (0.042, 0.477)
Llorca 2021 0.143 (0.036, 0.427)
Lopian 2020 0.095 (0.024, 0.311)
Molina 2020 0.125 (0.017, 0.537)
Nayak 2020 0.007 (0.001, 0.051)
Onwuzurike 2020 0.077 (0.011, 0.391)
Pecks 2020 0.058 (0.032, 0.102)
Singh 2021 0.033 (0.013, 0.084)
Subgroup Poor quality (I^2=29.51 % , P=0.183) 0.062 (0.037, 0.102)
Daz−Corvillon 2020 0.027 (0.004, 0.168)
Subgroup Good quality (I^2=NA , P=NA) 0.027 (0.004, 0.168)
Pereira 2020 0.160 (0.061, 0.357)
Subgroup Fair quality (I^2=NA , P=NA) 0.160 (0.061, 0.357)
Overall (I^2=62.01 % , P=0.000) 0.062 (0.041, 0.093)
0
0.12
0.24
0.37
0.49
Operative vaginal delivery rate with 95% CI

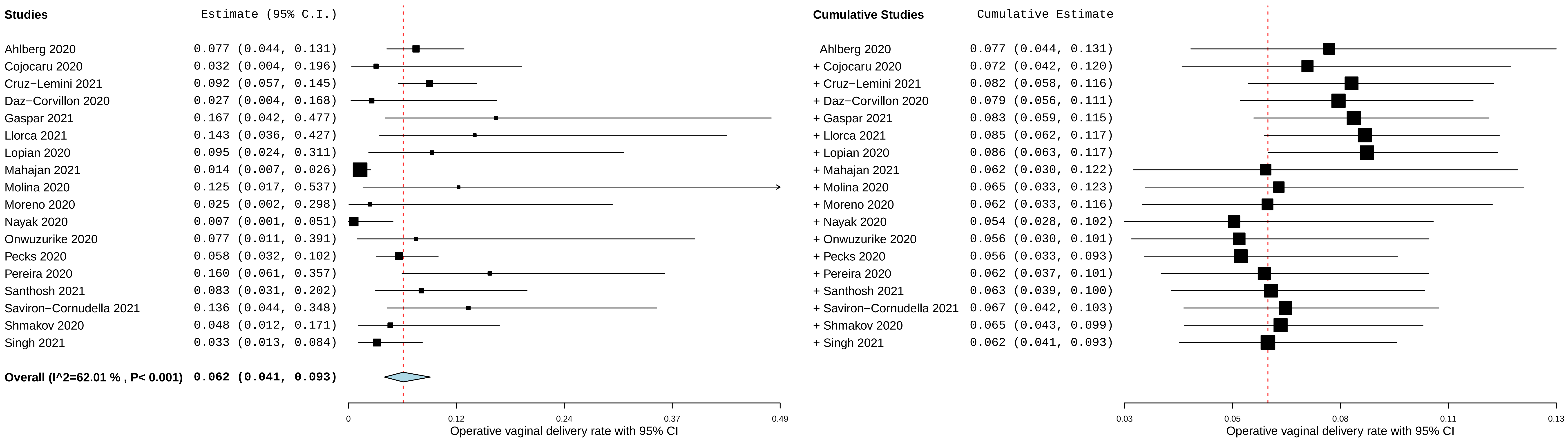
Studies
Estimate (95% C.I.)
Ahlberg 2020 0.077 (0.044, 0.131)
Cojocaru 2020 0.032 (0.004, 0.196)
Cruz−Lemini 2021 0.092 (0.057, 0.145)
Daz−Corvillon 2020 0.027 (0.004, 0.168)
Gaspar 2021 0.167 (0.042, 0.477)
Llorca 2021 0.143 (0.036, 0.427)
Lopian 2020 0.095 (0.024, 0.311)
Mahajan 2021 0.014 (0.007, 0.026)
Molina 2020 0.125 (0.017, 0.537)
Moreno 2020 0.025 (0.002, 0.298)
Nayak 2020 0.007 (0.001, 0.051)
Onwuzurike 2020 0.077 (0.011, 0.391)
Pecks 2020 0.058 (0.032, 0.102)
Pereira 2020 0.160 (0.061, 0.357)
Santhosh 2021 0.083 (0.031, 0.202)
Saviron−Cornudella 2021 0.136 (0.044, 0.348)
Shmakov 2020 0.048 (0.012, 0.171)
Singh 2021 0.033 (0.013, 0.084)
Overall (I^2=62.01 % , P< 0.001) 0.062 (0.041, 0.093)
0
0.12
0.24
0.37
0.49
Operative vaginal delivery rate with 95% CI
Cumulative Studies
Cumulative Estimate
Ahlberg 2020 0.077 (0.044, 0.131)
+ Cojocaru 2020 0.072 (0.042, 0.120)
+ Cruz−Lemini 2021 0.082 (0.058, 0.116)
+ Daz−Corvillon 2020 0.079 (0.056, 0.111)
+ Gaspar 2021 0.083 (0.059, 0.115)
+ Llorca 2021 0.085 (0.062, 0.117)
+ Lopian 2020 0.086 (0.063, 0.117)
+ Mahajan 2021 0.062 (0.030, 0.122)
+ Molina 2020 0.065 (0.033, 0.123)
+ Moreno 2020 0.062 (0.033, 0.116)
+ Nayak 2020 0.054 (0.028, 0.102)
+ Onwuzurike 2020 0.056 (0.030, 0.101)
+ Pecks 2020 0.056 (0.033, 0.093)
+ Pereira 2020 0.062 (0.037, 0.101)
+ Santhosh 2021 0.063 (0.039, 0.100)
+ Saviron−Cornudella 2021 0.067 (0.042, 0.103)
+ Shmakov 2020 0.065 (0.043, 0.099)
+ Singh 2021 0.062 (0.041, 0.093)
0.03
0.05
0.08
0.11
0.13
Operative vaginal delivery rate with 95% CI

Studies	Estimate (95% C.I.)
Abedzadeh−Kalahroudi 2021	0.661 (0.548, 0.797)
Antoun 2020	0.842 (0.693, 1.023)
Campbell 2020	0.333 (0.201, 0.553)
Cohen 2020	0.357 (0.177, 0.721)
Cojocaru 2020	0.258 (0.142, 0.469)
Cribiù 2020	0.429 (0.262, 0.702)
Gaspar 2021	0.417 (0.214, 0.814)
Ghema 2021	0.767 (0.630, 0.934)
Hui Yang 2020	0.783 (0.631, 0.971)
Hui yang 2020	0.692 (0.481, 0.995)
Jenabi 2020	0.433 (0.342, 0.549)
Llorca 2021	0.286 (0.125, 0.654)
Lokken 2021	0.455 (0.374, 0.553)
Lopian 2020	0.190 (0.078, 0.460)
Molina 2020	0.625 (0.365, 1.069)
Nambair 2020	0.723 (0.669, 0.781)
Nayak 2020	0.500 (0.422, 0.592)
Ochiai 2020	0.875 (0.604, 1.267)
Oncel 2020	0.712 (0.637, 0.796)
Onwuzurike 2020	0.462 (0.257, 0.830)
Pecks 2020	0.397 (0.333, 0.473)
Pineles 2020	0.273 (0.190, 0.393)
Pu Yang 2020	0.937 (0.783, 1.121)
Qiancheng 2020	0.773 (0.617, 0.969)
Qing−Lei Zeng 2020	0.833 (0.502, 1.382)
Santana 2021	0.483 (0.331, 0.704)
Savasi 2020	0.386 (0.278, 0.535)
Sherer 2020	0.318 (0.172, 0.587)
Singh 2021	0.639 (0.559, 0.730)
Suyuthi 2020	0.708 (0.547, 0.916)
Xu 2020 (China)	0.692 (0.535, 0.895)
Yan 2020	0.859 (0.793, 0.930)
Yin 2020	0.765 (0.588, 0.995)
Yingchun Zeng 2020	0.750 (0.565, 0.995)
Zou 2020	0.929 (0.757, 1.140)
**Subgroup Poor quality (I^2=85.54 % , P=0.000)**	**0.593 (0.535, 0.657)**
Ahlberg 2020	0.232 (0.174, 0.309)
Anand 2020	0.377 (0.279, 0.510)
Bachani 2020	0.456 (0.343, 0.606)
Barbero 2020	0.522 (0.353, 0.772)
Cruz−Lemini 2021	0.207 (0.155, 0.277)
Di Guardo 2021	0.255 (0.193, 0.337)
Farghaly 2020	0.667 (0.467, 0.953)
Goyal 2020	0.571 (0.300, 1.085)
Knight 2020	0.595 (0.538, 0.658)
Lu Zhang 2020	0.944 (0.844, 1.056)
Mahajan 2021	0.376 (0.341, 0.415)
Maru 2020	0.342 (0.220, 0.532)
Moreno 2020	0.368 (0.204, 0.664)
Ozsurmeli 2021	0.900 (0.732, 1.107)
Pierce−Williams 2020	0.750 (0.614, 0.916)
Prabhu 2020	0.457 (0.354, 0.590)
Sahin 2020−	0.664 (0.588, 0.750)
Samadi 2021	0.911 (0.877, 0.946)
Santhosh 2021	0.417 (0.299, 0.582)
Savirón−Cornudella 2020	0.071 (0.005, 1.032)
Saviron−Cornudella 2021	0.136 (0.047, 0.390)
Shmakov 2020	0.405 (0.281, 0.584)
Smithgall 2020	0.490 (0.370, 0.649)
Tug 2020	0.817 (0.725, 0.921)
Villalan 2020	0.208 (0.176, 0.246)
Vizheh 2021	0.833 (0.733, 0.946)
Wang 2020 (China)	0.767 (0.630, 0.934)
Wu 2020	0.931 (0.843, 1.028)
Yu 2020	0.937 (0.783, 1.121)
**Subgroup Fair quality (I^2=96.86 % , P=0.000)**	**0.508 (0.429, 0.602)**
Ajith 2021	0.682 (0.623, 0.746)
Di Mascio 2020	0.511 (0.454, 0.575)
Daz−Corvillon 2020	0.486 (0.349, 0.677)
Dumitriu 2020	0.455 (0.367, 0.564)
Hcinia 2021	0.139 (0.092, 0.211)
PACHTMAN 2020	0.260 (0.178, 0.379)
Reale 2020	0.344 (0.260, 0.456)
Rong Yang 2020	0.800 (0.709, 0.903)
Sahin 2020	0.500 (0.269, 0.929)
salvatore2020	0.439 (0.344, 0.561)
San−juan 2020	0.833 (0.582, 1.192)
Vivanti 2020	0.485 (0.341, 0.689)
Wang2020 (USA)	0.434 (0.319, 0.590)
Wei Liu 2020	0.933 (0.814, 1.069)
**Subgroup Good quality (I^2=92.8 % , P=0.000)**	**0.486 (0.397, 0.594)**
Anuk 2021	0.733 (0.590, 0.910)
BRANDT 2020	0.230 (0.146, 0.363)
Luming Xu 2020	0.800 (0.516, 1.240)
Martinez−Perez 2021	0.224 (0.178, 0.282)
Soto−Torres 2020	0.274 (0.201, 0.373)
**Subgroup (I^2=94.7 % , P=0.000)**	**0.383 (0.213, 0.688)**
Peng 2020	0.792 (0.645, 0.972)
Pereira 2020	0.200 (0.091, 0.438)
**Subgroup Fair quality (I^2=90.98 % , P=0.001)**	**0.420 (0.110, 1.612)**
**Overall (I^2=94.44 % , P=0.000)**	**0.523 (0.479, 0.570)**

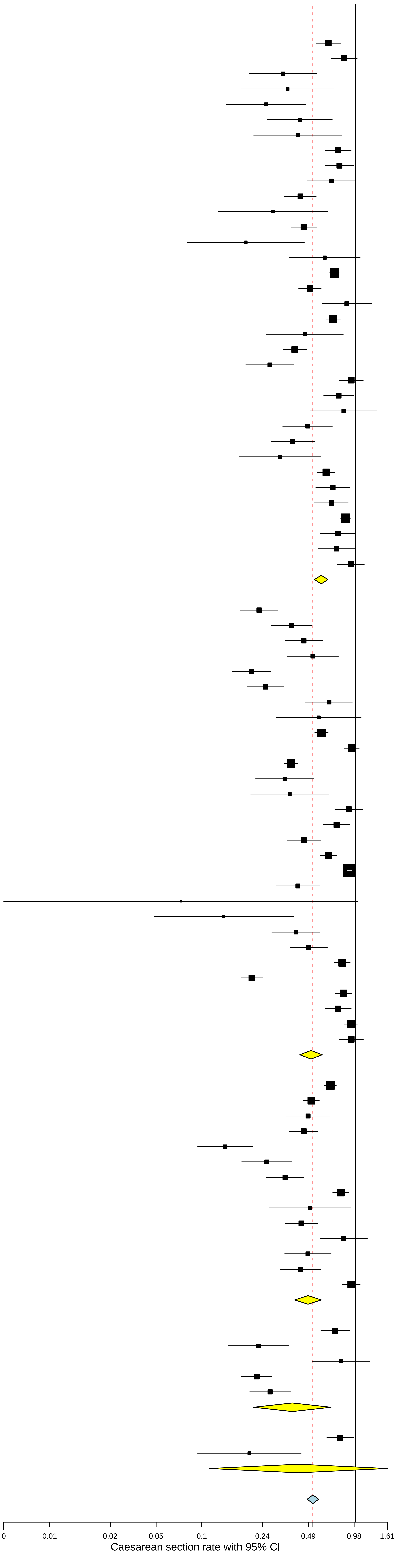

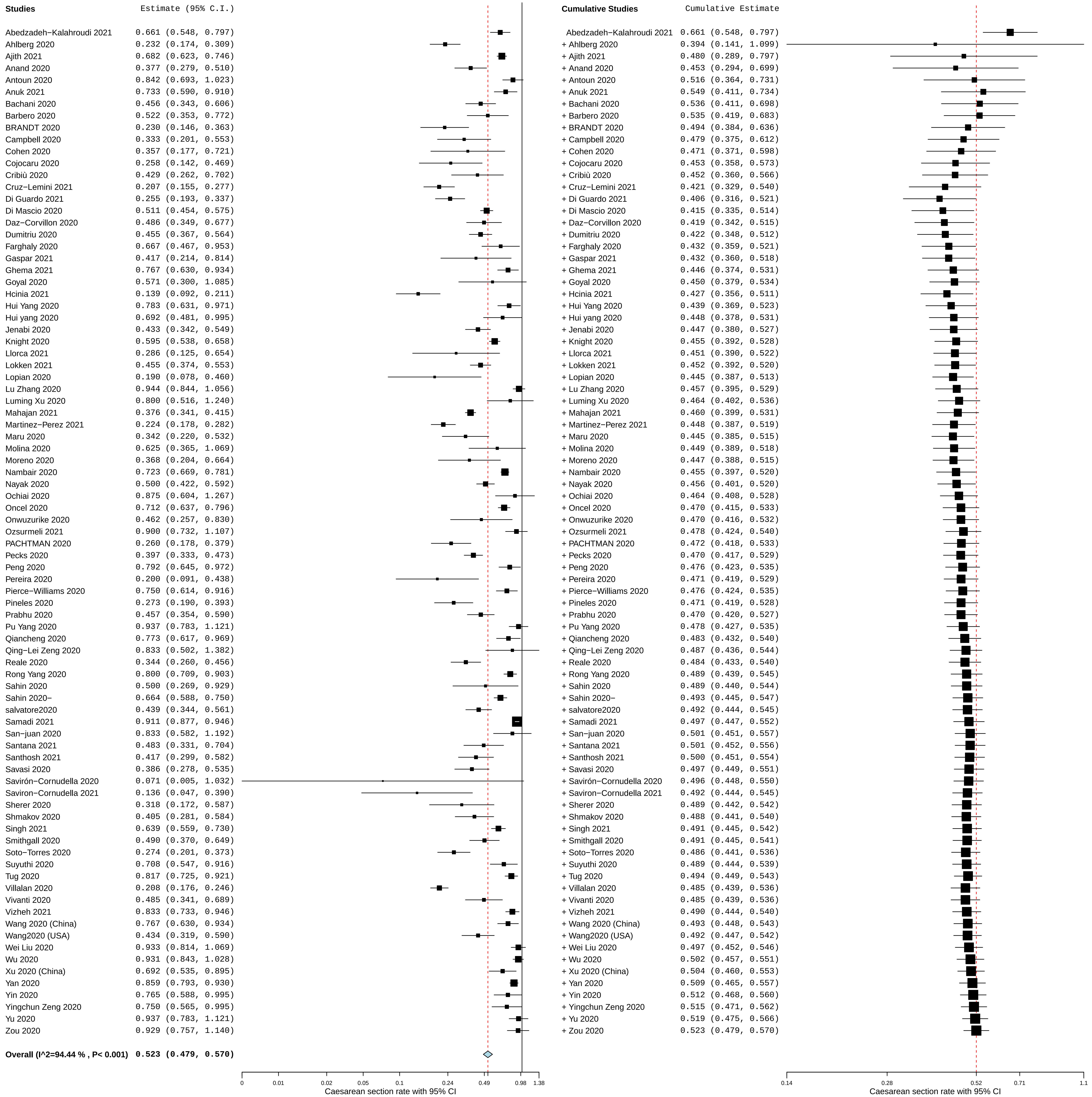

Studies
Estimate (95% C.I.)
Abedzadeh−Kalahroudi 2021 0.661 (0.548, 0.797)
Ahlberg 2020 0.232 (0.174, 0.309)
Ajith 2021 0.682 (0.623, 0.746)
Anand 2020 0.377 (0.279, 0.510)
Antoun 2020 0.842 (0.693, 1.023)
Anuk 2021 0.733 (0.590, 0.910)
Bachani 2020 0.456 (0.343, 0.606)
Barbero 2020 0.522 (0.353, 0.772)
BRANDT 2020 0.230 (0.146, 0.363)
Campbell 2020 0.333 (0.201, 0.553)
Cohen 2020 0.357 (0.177, 0.721)
Cojocaru 2020 0.258 (0.142, 0.469)
Cribiù 2020 0.429 (0.262, 0.702)
Cruz−Lemini 2021 0.207 (0.155, 0.277)
Di Guardo 2021 0.255 (0.193, 0.337)
Di Mascio 2020 0.511 (0.454, 0.575)
Daz−Corvillon 2020 0.486 (0.349, 0.677)
Dumitriu 2020 0.455 (0.367, 0.564)
Farghaly 2020 0.667 (0.467, 0.953)
Gaspar 2021 0.417 (0.214, 0.814)
Ghema 2021 0.767 (0.630, 0.934)
Goyal 2020 0.571 (0.300, 1.085)
Hcinia 2021 0.139 (0.092, 0.211)
Hui Yang 2020 0.783 (0.631, 0.971)
Hui yang 2020 0.692 (0.481, 0.995)
Jenabi 2020 0.433 (0.342, 0.549)
Knight 2020 0.595 (0.538, 0.658)
Llorca 2021 0.286 (0.125, 0.654)
Lokken 2021 0.455 (0.374, 0.553)
Lopian 2020 0.190 (0.078, 0.460)
Lu Zhang 2020 0.944 (0.844, 1.056)
Luming Xu 2020 0.800 (0.516, 1.240)
Mahajan 2021 0.376 (0.341, 0.415)
Martinez−Perez 2021 0.224 (0.178, 0.282)
Maru 2020 0.342 (0.220, 0.532)
Molina 2020 0.625 (0.365, 1.069)
Moreno 2020 0.368 (0.204, 0.664)
Nambair 2020 0.723 (0.669, 0.781)
Nayak 2020 0.500 (0.422, 0.592)
Ochiai 2020 0.875 (0.604, 1.267)
Oncel 2020 0.712 (0.637, 0.796)
Onwuzurike 2020 0.462 (0.257, 0.830)
Ozsurmeli 2021 0.900 (0.732, 1.107)
PACHTMAN 2020 0.260 (0.178, 0.379)
Pecks 2020 0.397 (0.333, 0.473)
Peng 2020 0.792 (0.645, 0.972)
Pereira 2020 0.200 (0.091, 0.438)
Pierce−Williams 2020 0.750 (0.614, 0.916)
Pineles 2020 0.273 (0.190, 0.393)
Prabhu 2020 0.457 (0.354, 0.590)
Pu Yang 2020 0.937 (0.783, 1.121)
Qiancheng 2020 0.773 (0.617, 0.969)
Qing−Lei Zeng 2020 0.833 (0.502, 1.382)
Reale 2020 0.344 (0.260, 0.456)
Rong Yang 2020 0.800 (0.709, 0.903)
Sahin 2020 0.500 (0.269, 0.929)
Sahin 2020− 0.664 (0.588, 0.750)
salvatore2020 0.439 (0.344, 0.561)
Samadi 2021 0.911 (0.877, 0.946)
San−juan 2020 0.833 (0.582, 1.192)
Santana 2021 0.483 (0.331, 0.704)
Santhosh 2021 0.417 (0.299, 0.582)
Savasi 2020 0.386 (0.278, 0.535)
Savirón−Cornudella 2020 0.071 (0.005, 1.032)
Saviron−Cornudella 2021 0.136 (0.047, 0.390)
Sherer 2020 0.318 (0.172, 0.587)
Shmakov 2020 0.405 (0.281, 0.584)
Singh 2021 0.639 (0.559, 0.730)
Smithgall 2020 0.490 (0.370, 0.649)
Soto−Torres 2020 0.274 (0.201, 0.373)
Suyuthi 2020 0.708 (0.547, 0.916)
Tug 2020 0.817 (0.725, 0.921)
Villalan 2020 0.208 (0.176, 0.246)
Vivanti 2020 0.485 (0.341, 0.689)
Vizheh 2021 0.833 (0.733, 0.946)
Wang 2020 (China) 0.767 (0.630, 0.934)
Wang2020 (USA) 0.434 (0.319, 0.590)
Wei Liu 2020 0.933 (0.814, 1.069)
Wu 2020 0.931 (0.843, 1.028)
Xu 2020 (China) 0.692 (0.535, 0.895)
Yan 2020 0.859 (0.793, 0.930)
Yin 2020 0.765 (0.588, 0.995)
Yingchun Zeng 2020 0.750 (0.565, 0.995)
Yu 2020 0.937 (0.783, 1.121)
Zou 2020 0.929 (0.757, 1.140)
Overall (I^2=94.44 % , P< 0.001) 0.523 (0.479, 0.570)
0
0.01
0.02
0.05
0.1
0.24
0.49
0.98
1.38
Caesarean section rate with 95% CI
Cumulative Studies
Cumulative Estimate
Abedzadeh−Kalahroudi 2021 0.661 (0.548, 0.797)
+ Ahlberg 2020 0.394 (0.141, 1.099)
+ Ajith 2021 0.480 (0.289, 0.797)
+ Anand 2020 0.453 (0.294, 0.699)
+ Antoun 2020 0.516 (0.364, 0.731)
+ Anuk 2021 0.549 (0.411, 0.734)
+ Bachani 2020 0.536 (0.411, 0.698)
+ Barbero 2020 0.535 (0.419, 0.683)
+ BRANDT 2020 0.494 (0.384, 0.636)
+ Campbell 2020 0.479 (0.375, 0.612)
+ Cohen 2020 0.471 (0.371, 0.598)
+ Cojocaru 2020 0.453 (0.358, 0.573)
+ Cribiù 2020 0.452 (0.360, 0.566)
+ Cruz−Lemini 2021 0.421 (0.329, 0.540)
+ Di Guardo 2021 0.406 (0.316, 0.521)
+ Di Mascio 2020 0.415 (0.335, 0.514)
+ Daz−Corvillon 2020 0.419 (0.342, 0.515)
+ Dumitriu 2020 0.422 (0.348, 0.512)
+ Farghaly 2020 0.432 (0.359, 0.521)
+ Gaspar 2021 0.432 (0.360, 0.518)
+ Ghema 2021 0.446 (0.374, 0.531)
+ Goyal 2020 0.450 (0.379, 0.534)
+ Hcinia 2021 0.427 (0.356, 0.511)
+ Hui Yang 2020 0.439 (0.369, 0.523)
+ Hui yang 2020 0.448 (0.378, 0.531)
+ Jenabi 2020 0.447 (0.380, 0.527)
+ Knight 2020 0.455 (0.392, 0.528)
+ Llorca 2021 0.451 (0.390, 0.522)
+ Lokken 2021 0.452 (0.392, 0.520)
+ Lopian 2020 0.445 (0.387, 0.513)
+ Lu Zhang 2020 0.457 (0.395, 0.529)
+ Luming Xu 2020 0.464 (0.402, 0.536)
+ Mahajan 2021 0.460 (0.399, 0.531)
+ Martinez−Perez 2021 0.448 (0.387, 0.519)
+ Maru 2020 0.445 (0.385, 0.515)
+ Molina 2020 0.449 (0.389, 0.518)
+ Moreno 2020 0.447 (0.388, 0.515)
+ Nambair 2020 0.455 (0.397, 0.520)
+ Nayak 2020 0.456 (0.401, 0.520)
+ Ochiai 2020 0.464 (0.408, 0.528)
+ Oncel 2020 0.470 (0.415, 0.533)
+ Onwuzurike 2020 0.470 (0.416, 0.532)
+ Ozsurmeli 2021 0.478 (0.424, 0.540)
+ PACHTMAN 2020 0.472 (0.418, 0.533)
+ Pecks 2020 0.470 (0.417, 0.529)
+ Peng 2020 0.476 (0.423, 0.535)
+ Pereira 2020 0.471 (0.419, 0.529)
+ Pierce−Williams 2020 0.476 (0.424, 0.535)
+ Pineles 2020 0.471 (0.419, 0.528)
+ Prabhu 2020 0.470 (0.420, 0.527)
+ Pu Yang 2020 0.478 (0.427, 0.535)
+ Qiancheng 2020 0.483 (0.432, 0.540)
+ Qing−Lei Zeng 2020 0.487 (0.436, 0.544)
+ Reale 2020 0.484 (0.433, 0.540)
+ Rong Yang 2020 0.489 (0.439, 0.545)
+ Sahin 2020 0.489 (0.440, 0.544)
+ Sahin 2020− 0.493 (0.445, 0.547)
+ salvatore2020 0.492 (0.444, 0.545)
+ Samadi 2021 0.497 (0.447, 0.552)
+ San−juan 2020 0.501 (0.451, 0.557)
+ Santana 2021 0.501 (0.452, 0.556)
+ Santhosh 2021 0.500 (0.451, 0.554)
+ Savasi 2020 0.497 (0.449, 0.551)
+ Savirón−Cornudella 2020 0.496 (0.448, 0.550)
+ Saviron−Cornudella 2021 0.492 (0.444, 0.545)
+ Sherer 2020 0.489 (0.442, 0.542)
+ Shmakov 2020 0.488 (0.441, 0.540)
+ Singh 2021 0.491 (0.445, 0.542)
+ Smithgall 2020 0.491 (0.445, 0.541)
+ Soto−Torres 2020 0.486 (0.441, 0.536)
+ Suyuthi 2020 0.489 (0.444, 0.539)
+ Tug 2020 0.494 (0.449, 0.543)
+ Villalan 2020 0.485 (0.439, 0.536)
+ Vivanti 2020 0.485 (0.439, 0.536)
+ Vizheh 2021 0.490 (0.444, 0.540)
+ Wang 2020 (China) 0.493 (0.448, 0.543)
+ Wang2020 (USA) 0.492 (0.447, 0.542)
+ Wei Liu 2020 0.497 (0.452, 0.546)
+ Wu 2020 0.502 (0.457, 0.551)
+ Xu 2020 (China) 0.504 (0.460, 0.553)
+ Yan 2020 0.509 (0.465, 0.557)
+ Yin 2020 0.512 (0.468, 0.560)
+ Yingchun Zeng 2020 0.515 (0.471, 0.562)
+ Yu 2020 0.519 (0.475, 0.566)
+ Zou 2020 0.523 (0.479, 0.570)
0.14
0.28
0.52
0.71
1.1
Caesarean section rate with 95% CI

Studies	Estimate (95% C.I.)
Abedzadeh−Kalahroudi 2021	0.339 (0.243, 0.472)
Antoun 2020	0.625 (0.446, 0.875)
Barbero 2020	0.500 (0.266, 0.941)
BRANDT 2020	0.400 (0.200, 0.800)
Di Guardo 2021	0.286 (0.161, 0.508)
Daz−Corvillon 2020	0.280 (0.163, 0.482)
Dumitriu 2020	0.264 (0.200, 0.348)
Farghaly 2020	0.263 (0.163, 0.424)
Flaherman 2020	0.255 (0.166, 0.391)
Gaspar 2021	0.242 (0.163, 0.360)
Ghema 2021	0.231 (0.102, 0.522)
Hui yang 2020	0.200 (0.136, 0.295)
Knight 2020	0.200 (0.074, 0.541)
Leon−Abarca 2020	0.196 (0.117, 0.327)
Liu 2020	0.190 (0.107, 0.337)
Llorca 2021	0.188 (0.079, 0.447)
Lu Zhang 2020	0.170 (0.098, 0.295)
Mahajan 2021	0.167 (0.068, 0.409)
Martinez−Perez 2021	0.158 (0.064, 0.392)
Martinez−Portilla	0.154 (0.069, 0.345)
Nambair 2020	0.125 (0.021, 0.734)
Ochiai 2020	0.117 (0.079, 0.173)
Oncel 2020	0.116 (0.066, 0.203)
Ozsurmeli 2021	0.110 (0.064, 0.188)
Pecks 2020	0.097 (0.061, 0.154)
Prabhu 2020	0.067 (0.013, 0.352)
Pu Yang 2020	0.065 (0.029, 0.147)
Reale 2020	0.042 (0.007, 0.244)
Ríos−Silva 2020	0.020 (0.003, 0.129)
**Subgroup Poor quality (I^2=75.57 % , P=0.000)**	**0.197 (0.158, 0.244)**
Ahlberg 2020	0.090 (0.055, 0.147)
Anand 2020	0.667 (0.575, 0.774)
Bachani 2020	0.571 (0.381, 0.856)
Blitz 2020	0.412 (0.262, 0.648)
Campbell 2020	0.379 (0.312, 0.461)
Cheng 2020	0.375 (0.271, 0.518)
Cruz−Lemini 2021	0.289 (0.222, 0.376)
Di Mascio 2020	0.286 (0.161, 0.508)
Gale 2020	0.246 (0.201, 0.301)
Goyal 2020	0.228 (0.147, 0.354)
Handley 2020	0.211 (0.133, 0.335)
He 2020	0.208 (0.147, 0.295)
Hui Yang 2020	0.200 (0.106, 0.379)
Jenabi 2020	0.200 (0.093, 0.428)
Lopian 2020	0.171 (0.109, 0.268)
Luming Xu 2020	0.168 (0.117, 0.242)
Mattern 2021	0.135 (0.100, 0.183)
Nayak 2020	0.125 (0.054, 0.289)
Onwuzurike 2020	0.115 (0.060, 0.222)
PACHTMAN 2020	0.108 (0.046, 0.255)
Peng 2020	0.092 (0.072, 0.117)
Pereira 2020	0.083 (0.017, 0.413)
Pirjani 2020	0.075 (0.045, 0.124)
Rizzo 2021	0.016 (0.001, 0.211)
**Subgroup Fair quality (I^2=93.47 % , P=0.000)**	**0.200 (0.149, 0.268)**
Ajith 2021	0.682 (0.554, 0.840)
Cohen 2020	0.350 (0.213, 0.574)
Cojocaru 2020	0.333 (0.151, 0.732)
Cribiù 2020	0.294 (0.159, 0.542)
Facchetti 2020	0.263 (0.217, 0.319)
Maru 2020	0.151 (0.090, 0.252)
Mattar 2020	0.138 (0.078, 0.245)
Molina 2020	0.133 (0.044, 0.405)
Moreno 2020	0.132 (0.093, 0.188)
Pierce−Williams 2020	0.081 (0.053, 0.124)
Pineles 2020	0.080 (0.046, 0.139)
Qiancheng 2020	0.063 (0.007, 0.539)
**Subgroup Good quality (I^2=92.95 % , P=0.000)**	**0.189 (0.118, 0.303)**
Anuk 2021	0.594 (0.472, 0.747)
Badr 2020	0.507 (0.413, 0.623)
Gulersen 2020	0.217 (0.110, 0.428)
Hcinia 2021	0.210 (0.147, 0.301)
PATBERG 2020	0.103 (0.038, 0.276)
Qing−Lei Zeng 2020	0.043 (0.007, 0.252)
**Subgroup (I^2=88.57 % , P=0.000)**	**0.270 (0.163, 0.446)**
Lokken 2021	0.176 (0.073, 0.427)
**Subgroup Fair quality (I^2=NA , P=NA)**	**0.176 (0.073, 0.427)**
**Overall (I^2=90.23 % , P=0.000)**	**0.200 (0.169, 0.235)**

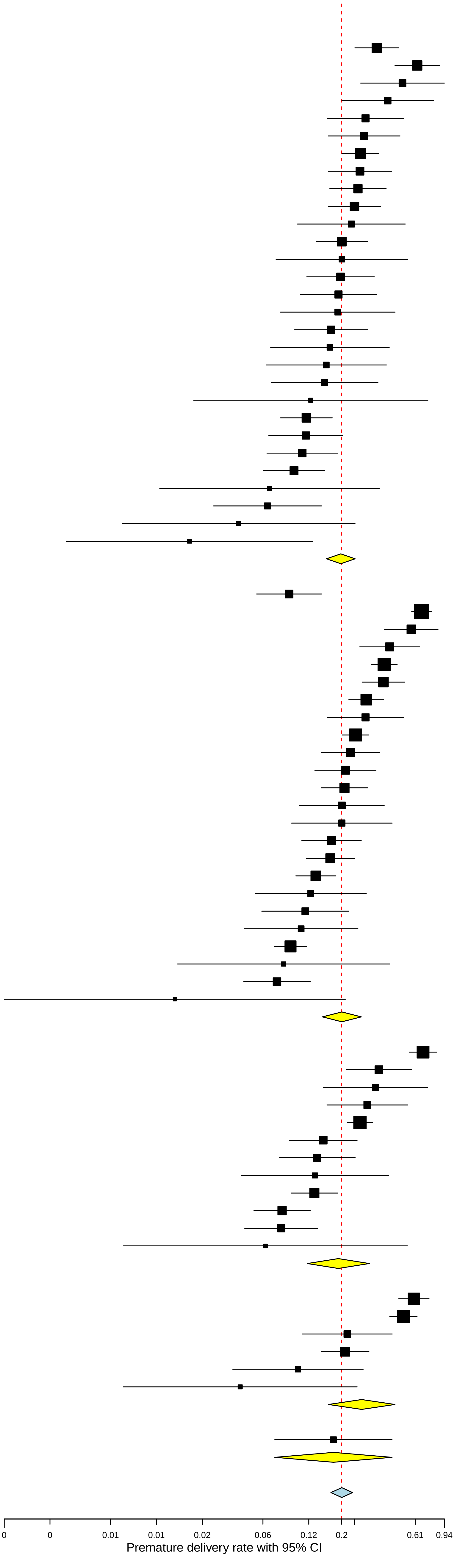

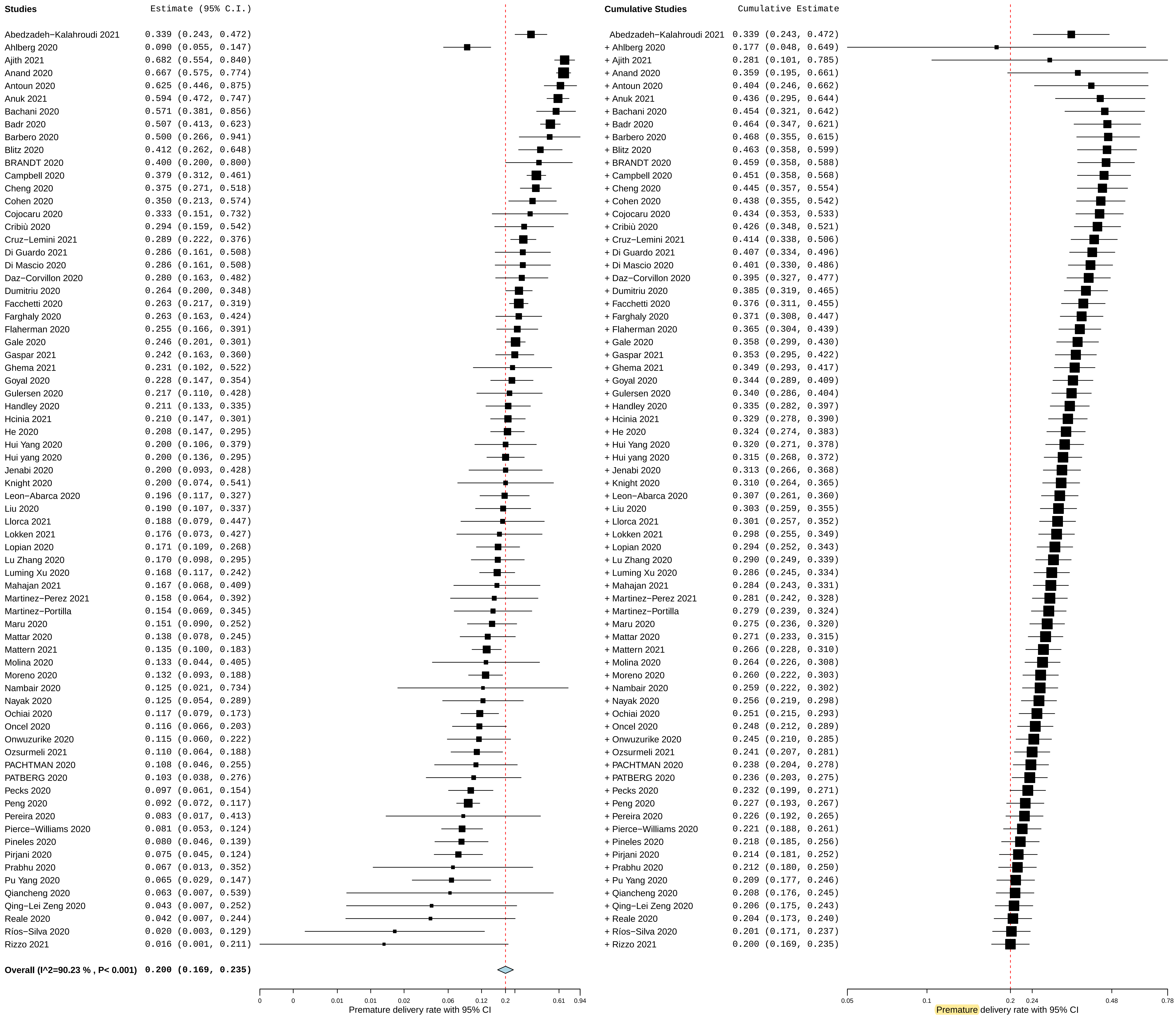
Studies
Estimate (95% C.I.)
Abedzadeh−Kalahroudi 2021 0.339 (0.243, 0.472)
Ahlberg 2020 0.090 (0.055, 0.147)
Ajith 2021 0.682 (0.554, 0.840)
Anand 2020 0.667 (0.575, 0.774)
Antoun 2020 0.625 (0.446, 0.875)
Anuk 2021 0.594 (0.472, 0.747)
Bachani 2020 0.571 (0.381, 0.856)
Badr 2020 0.507 (0.413, 0.623)
Barbero 2020 0.500 (0.266, 0.941)
Blitz 2020 0.412 (0.262, 0.648)
BRANDT 2020 0.400 (0.200, 0.800)
Campbell 2020 0.379 (0.312, 0.461)
Cheng 2020 0.375 (0.271, 0.518)
Cohen 2020 0.350 (0.213, 0.574)
Cojocaru 2020 0.333 (0.151, 0.732)
Cribiù 2020 0.294 (0.159, 0.542)
Cruz−Lemini 2021 0.289 (0.222, 0.376)
Di Guardo 2021 0.286 (0.161, 0.508)
Di Mascio 2020 0.286 (0.161, 0.508)
Daz−Corvillon 2020 0.280 (0.163, 0.482)
Dumitriu 2020 0.264 (0.200, 0.348)
Facchetti 2020 0.263 (0.217, 0.319)
Farghaly 2020 0.263 (0.163, 0.424)
Flaherman 2020 0.255 (0.166, 0.391)
Gale 2020 0.246 (0.201, 0.301)
Gaspar 2021 0.242 (0.163, 0.360)
Ghema 2021 0.231 (0.102, 0.522)
Goyal 2020 0.228 (0.147, 0.354)
Gulersen 2020 0.217 (0.110, 0.428)
Handley 2020 0.211 (0.133, 0.335)
Hcinia 2021 0.210 (0.147, 0.301)
He 2020 0.208 (0.147, 0.295)
Hui Yang 2020 0.200 (0.106, 0.379)
Hui yang 2020 0.200 (0.136, 0.295)
Jenabi 2020 0.200 (0.093, 0.428)
Knight 2020 0.200 (0.074, 0.541)
Leon−Abarca 2020 0.196 (0.117, 0.327)
Liu 2020 0.190 (0.107, 0.337)
Llorca 2021 0.188 (0.079, 0.447)
Lokken 2021 0.176 (0.073, 0.427)
Lopian 2020 0.171 (0.109, 0.268)
Lu Zhang 2020 0.170 (0.098, 0.295)
Luming Xu 2020 0.168 (0.117, 0.242)
Mahajan 2021 0.167 (0.068, 0.409)
Martinez−Perez 2021 0.158 (0.064, 0.392)
Martinez−Portilla 0.154 (0.069, 0.345)
Maru 2020 0.151 (0.090, 0.252)
Mattar 2020 0.138 (0.078, 0.245)
Mattern 2021 0.135 (0.100, 0.183)
Molina 2020 0.133 (0.044, 0.405)
Moreno 2020 0.132 (0.093, 0.188)
Nambair 2020 0.125 (0.021, 0.734)
Nayak 2020 0.125 (0.054, 0.289)
Ochiai 2020 0.117 (0.079, 0.173)
Oncel 2020 0.116 (0.066, 0.203)
Onwuzurike 2020 0.115 (0.060, 0.222)
Ozsurmeli 2021 0.110 (0.064, 0.188)
PACHTMAN 2020 0.108 (0.046, 0.255)
PATBERG 2020 0.103 (0.038, 0.276)
Pecks 2020 0.097 (0.061, 0.154)
Peng 2020 0.092 (0.072, 0.117)
Pereira 2020 0.083 (0.017, 0.413)
Pierce−Williams 2020 0.081 (0.053, 0.124)
Pineles 2020 0.080 (0.046, 0.139)
Pirjani 2020 0.075 (0.045, 0.124)
Prabhu 2020 0.067 (0.013, 0.352)
Pu Yang 2020 0.065 (0.029, 0.147)
Qiancheng 2020 0.063 (0.007, 0.539)
Qing−Lei Zeng 2020 0.043 (0.007, 0.252)
Reale 2020 0.042 (0.007, 0.244)
Ríos−Silva 2020 0.020 (0.003, 0.129)
Rizzo 2021 0.016 (0.001, 0.211)
Overall (I^2=90.23 % , P< 0.001) 0.200 (0.169, 0.235)
0 0 0.01 0.01 0.02 0.06 0.12 0.2 0.61 0.94
Premature delivery rate with 95% CI
Cumulative Studies
Cumulative Estimate
Abedzadeh−Kalahroudi 2021 0.339 (0.243, 0.472)
+ Ahlberg 2020 0.177 (0.048, 0.649)
+ Ajith 2021 0.281 (0.101, 0.785)
+ Anand 2020 0.359 (0.195, 0.661)
+ Antoun 2020 0.404 (0.246, 0.662)
+ Anuk 2021 0.436 (0.295, 0.644)
+ Bachani 2020 0.454 (0.321, 0.642)
+ Badr 2020 0.464 (0.347, 0.621)
+ Barbero 2020 0.468 (0.355, 0.615)
+ Blitz 2020 0.463 (0.358, 0.599)
+ BRANDT 2020 0.459 (0.358, 0.588)
+ Campbell 2020 0.451 (0.358, 0.568)
+ Cheng 2020 0.445 (0.357, 0.554)
+ Cohen 2020 0.438 (0.355, 0.542)
+ Cojocaru 2020 0.434 (0.353, 0.533)
+ Cribiù 2020 0.426 (0.348, 0.521)
+ Cruz−Lemini 2021 0.414 (0.338, 0.506)
+ Di Guardo 2021 0.407 (0.334, 0.496)
+ Di Mascio 2020 0.401 (0.330, 0.486)
+ Daz−Corvillon 2020 0.395 (0.327, 0.477)
+ Dumitriu 2020 0.385 (0.319, 0.465)
+ Facchetti 2020 0.376 (0.311, 0.455)
+ Farghaly 2020 0.371 (0.308, 0.447)
+ Flaherman 2020 0.365 (0.304, 0.439)
+ Gale 2020 0.358 (0.299, 0.430)
+ Gaspar 2021 0.353 (0.295, 0.422)
+ Ghema 2021 0.349 (0.293, 0.417)
+ Goyal 2020 0.344 (0.289, 0.409)
+ Gulersen 2020 0.340 (0.286, 0.404)
+ Handley 2020 0.335 (0.282, 0.397)
+ Hcinia 2021 0.329 (0.278, 0.390)
+ He 2020 0.324 (0.274, 0.383)
+ Hui Yang 2020 0.320 (0.271, 0.378)
+ Hui yang 2020 0.315 (0.268, 0.372)
+ Jenabi 2020 0.313 (0.266, 0.368)
+ Knight 2020 0.310 (0.264, 0.365)
+ Leon−Abarca 2020 0.307 (0.261, 0.360)
+ Liu 2020 0.303 (0.259, 0.355)
+ Llorca 2021 0.301 (0.257, 0.352)
+ Lokken 2021 0.298 (0.255, 0.349)
+ Lopian 2020 0.294 (0.252, 0.343)
+ Lu Zhang 2020 0.290 (0.249, 0.339)
+ Luming Xu 2020 0.286 (0.245, 0.334)
+ Mahajan 2021 0.284 (0.243, 0.331)
+ Martinez−Perez 2021 0.281 (0.242, 0.328)
+ Martinez−Portilla 0.279 (0.239, 0.324)
+ Maru 2020 0.275 (0.236, 0.320)
+ Mattar 2020 0.271 (0.233, 0.315)
+ Mattern 2021 0.266 (0.228, 0.310)
+ Molina 2020 0.264 (0.226, 0.308)
+ Moreno 2020 0.260 (0.222, 0.303)
+ Nambair 2020 0.259 (0.222, 0.302)
+ Nayak 2020 0.256 (0.219, 0.298)
+ Ochiai 2020 0.251 (0.215, 0.293)
+ Oncel 2020 0.248 (0.212, 0.289)
+ Onwuzurike 2020 0.245 (0.210, 0.285)
+ Ozsurmeli 2021 0.241 (0.207, 0.281)
+ PACHTMAN 2020 0.238 (0.204, 0.278)
+ PATBERG 2020 0.236 (0.203, 0.275)
+ Pecks 2020 0.232 (0.199, 0.271)
+ Peng 2020 0.227 (0.193, 0.267)
+ Pereira 2020 0.226 (0.192, 0.265)
+ Pierce−Williams 2020 0.221 (0.188, 0.261)
+ Pineles 2020 0.218 (0.185, 0.256)
+ Pirjani 2020 0.214 (0.181, 0.252)
+ Prabhu 2020 0.212 (0.180, 0.250)
+ Pu Yang 2020 0.209 (0.177, 0.246)
+ Qiancheng 2020 0.208 (0.176, 0.245)
+ Qing−Lei Zeng 2020 0.206 (0.175, 0.243)
+ Reale 2020 0.204 (0.173, 0.240)
+ Ríos−Silva 2020 0.201 (0.171, 0.237)
+ Rizzo 2021 0.200 (0.169, 0.235)
0.05 0.1 0.2 0.24 0.48 0.78
Premature delivery rate with 95% CI

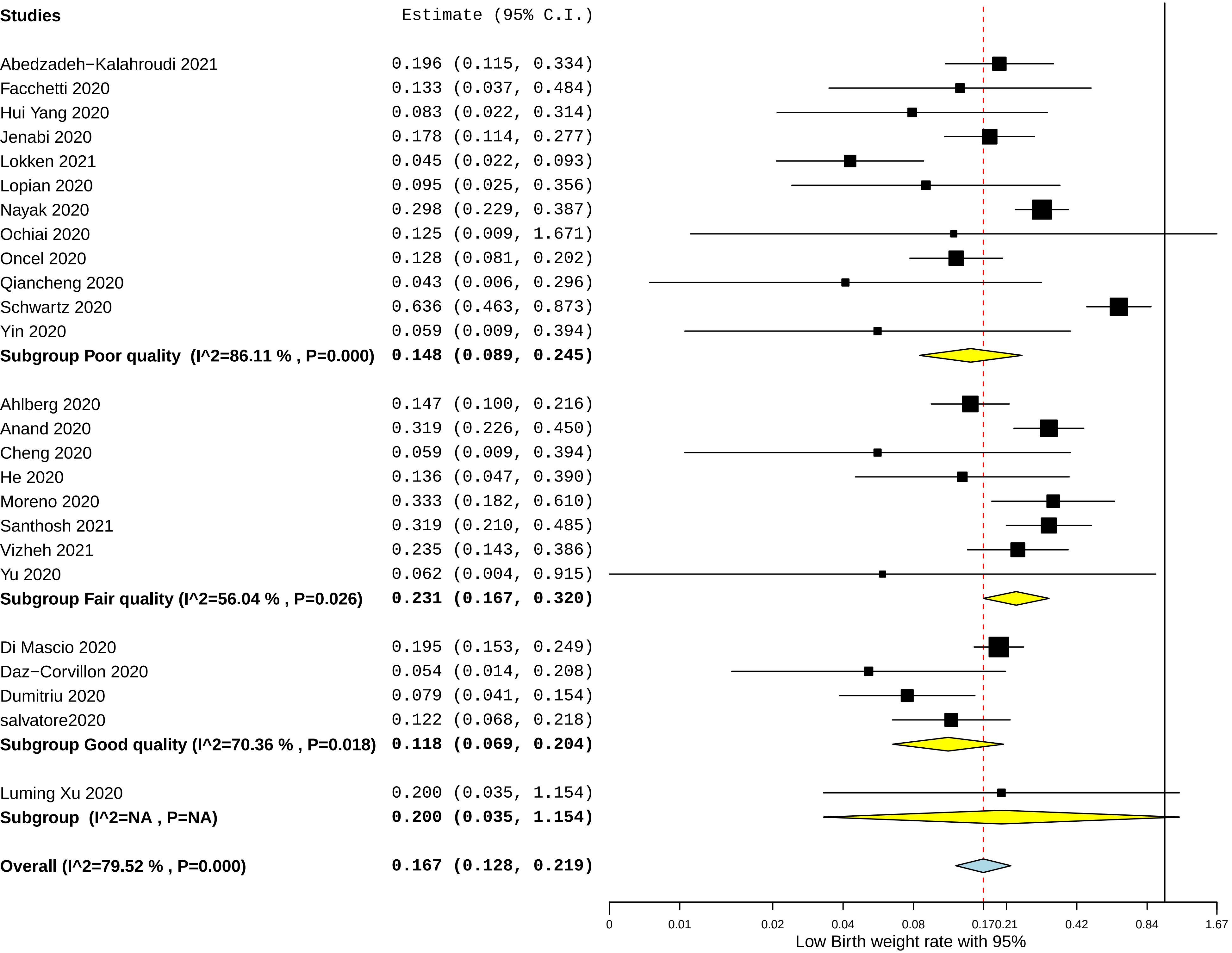
Studies
Estimate (95% C.I.)
Abedzadeh−Kalahroudi 2021 0.196 (0.115, 0.334)
Facchetti 2020 0.133 (0.037, 0.484)
Hui Yang 2020 0.083 (0.022, 0.314)
Jenabi 2020 0.178 (0.114, 0.277)
Lokken 2021 0.045 (0.022, 0.093)
Lopian 2020 0.095 (0.025, 0.356)
Nayak 2020 0.298 (0.229, 0.387)
Ochiai 2020 0.125 (0.009, 1.671)
Oncel 2020 0.128 (0.081, 0.202)
Qiancheng 2020 0.043 (0.006, 0.296)
Schwartz 2020 0.636 (0.463, 0.873)
Yin 2020 0.059 (0.009, 0.394)
Subgroup Poor quality (I^2=86.11 % , P=0.000) 0.148 (0.089, 0.245)
Ahlberg 2020 0.147 (0.100, 0.216)
Anand 2020 0.319 (0.226, 0.450)
Cheng 2020 0.059 (0.009, 0.394)
He 2020 0.136 (0.047, 0.390)
Moreno 2020 0.333 (0.182, 0.610)
Santhosh 2021 0.319 (0.210, 0.485)
Vizheh 2021 0.235 (0.143, 0.386)
Yu 2020 0.062 (0.004, 0.915)
Subgroup Fair quality (I^2=56.04 % , P=0.026) 0.231 (0.167, 0.320)
Di Mascio 2020 0.195 (0.153, 0.249)
Daz−Corvillon 2020 0.054 (0.014, 0.208)
Dumitriu 2020 0.079 (0.041, 0.154)
salvatore2020 0.122 (0.068, 0.218)
Subgroup Good quality (I^2=70.36 % , P=0.018) 0.118 (0.069, 0.204)
Luming Xu 2020 0.200 (0.035, 1.154)
Subgroup (I^2=NA , P=NA) 0.200 (0.035, 1.154)
Overall (I^2=79.52 % , P=0.000) 0.167 (0.128, 0.219)
0
0.01
0.02
0.04
0.08
0.17
0.21
0.42
0.84
1.67
Low Birth weight rate with 95%

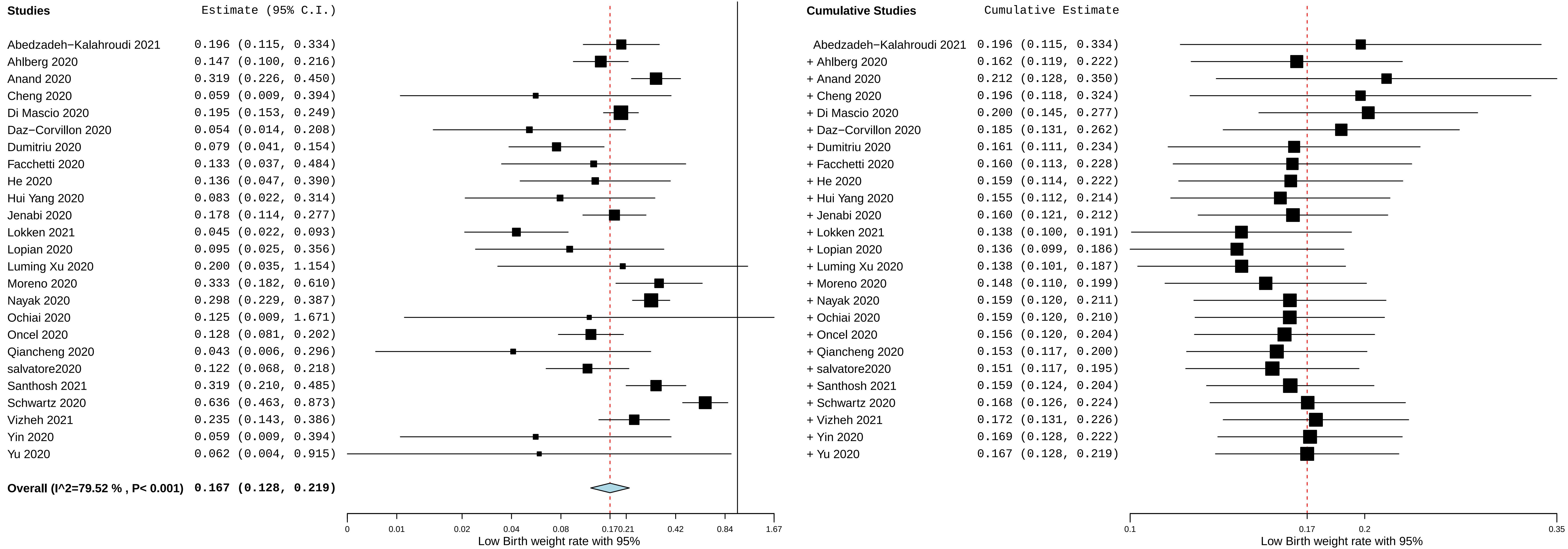
Studies
Estimate (95% C.I.)
Abedzadeh−Kalahroudi 2021 0.196 (0.115, 0.334)
Ahlberg 2020 0.147 (0.100, 0.216)
Anand 2020 0.319 (0.226, 0.450)
Cheng 2020 0.059 (0.009, 0.394)
Di Mascio 2020 0.195 (0.153, 0.249)
Daz−Corvillon 2020 0.054 (0.014, 0.208)
Dumitriu 2020 0.079 (0.041, 0.154)
Facchetti 2020 0.133 (0.037, 0.484)
He 2020 0.136 (0.047, 0.390)
Hui Yang 2020 0.083 (0.022, 0.314)
Jenabi 2020 0.178 (0.114, 0.277)
Lokken 2021 0.045 (0.022, 0.093)
Lopian 2020 0.095 (0.025, 0.356)
Luming Xu 2020 0.200 (0.035, 1.154)
Moreno 2020 0.333 (0.182, 0.610)
Nayak 2020 0.298 (0.229, 0.387)
Ochiai 2020 0.125 (0.009, 1.671)
Oncel 2020 0.128 (0.081, 0.202)
Qiancheng 2020 0.043 (0.006, 0.296)
salvatore2020 0.122 (0.068, 0.218)
Santhosh 2021 0.319 (0.210, 0.485)
Schwartz 2020 0.636 (0.463, 0.873)
Vizheh 2021 0.235 (0.143, 0.386)
Yin 2020 0.059 (0.009, 0.394)
Yu 2020 0.062 (0.004, 0.915)
Overall (I^2=79.52 % , P< 0.001) 0.167 (0.128, 0.219)
0 0.01 0.02 0.04 0.08 0.170.21 0.42 0.84 1.67
Low Birth weight rate with 95%
Cumulative Studies
Cumulative Estimate
Abedzadeh−Kalahroudi 2021 0.196 (0.115, 0.334)
+ Ahlberg 2020 0.162 (0.119, 0.222)
+ Anand 2020 0.212 (0.128, 0.350)
+ Cheng 2020 0.196 (0.118, 0.324)
+ Di Mascio 2020 0.200 (0.145, 0.277)
+ Daz−Corvillon 2020 0.185 (0.131, 0.262)
+ Dumitriu 2020 0.161 (0.111, 0.234)
+ Facchetti 2020 0.160 (0.113, 0.228)
+ He 2020 0.159 (0.114, 0.222)
+ Hui Yang 2020 0.155 (0.112, 0.214)
+ Jenabi 2020 0.160 (0.121, 0.212)
+ Lokken 2021 0.138 (0.100, 0.191)
+ Lopian 2020 0.136 (0.099, 0.186)
+ Luming Xu 2020 0.138 (0.101, 0.187)
+ Moreno 2020 0.148 (0.110, 0.199)
+ Nayak 2020 0.159 (0.120, 0.211)
+ Ochiai 2020 0.159 (0.120, 0.210)
+ Oncel 2020 0.156 (0.120, 0.204)
+ Qiancheng 2020 0.153 (0.117, 0.200)
+ salvatore2020 0.151 (0.117, 0.195)
+ Santhosh 2021 0.159 (0.124, 0.204)
+ Schwartz 2020 0.168 (0.126, 0.224)
+ Vizheh 2021 0.172 (0.131, 0.226)
+ Yin 2020 0.169 (0.128, 0.222)
+ Yu 2020 0.167 (0.128, 0.219)
0.1 0.17 0.2 0.35
Low Birth weight rate with 95%

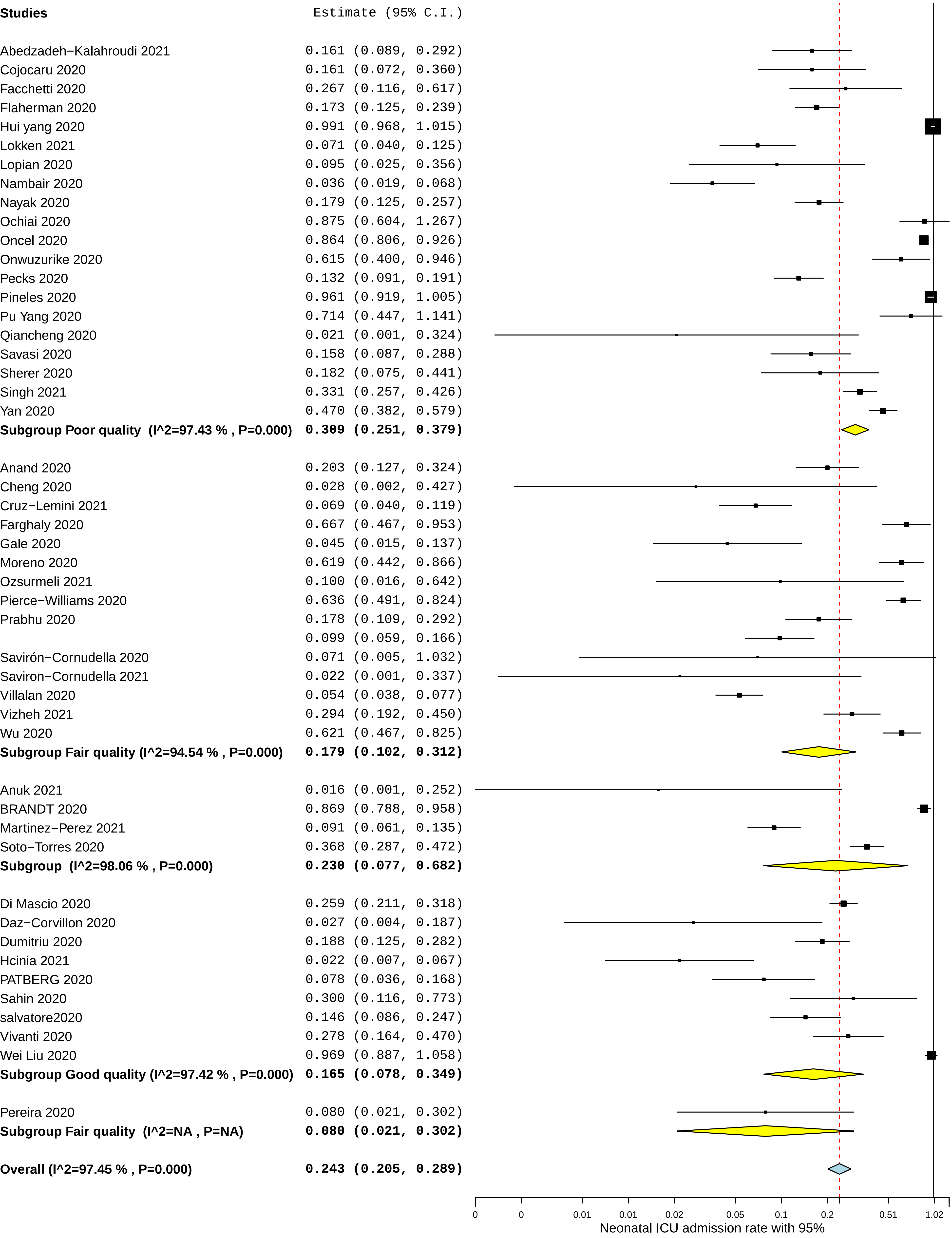
Studies
Estimate (95% C.I.)
Abedzadeh−Kalahroudi 2021 0.161 (0.089, 0.292)
Cojocaru 2020 0.161 (0.072, 0.360)
Facchetti 2020 0.267 (0.116, 0.617)
Flaherman 2020 0.173 (0.125, 0.239)
Hui yang 2020 0.991 (0.968, 1.015)
Lokken 2021 0.071 (0.040, 0.125)
Lopian 2020 0.095 (0.025, 0.356)
Nambair 2020 0.036 (0.019, 0.068)
Nayak 2020 0.179 (0.125, 0.257)
Ochiai 2020 0.875 (0.604, 1.267)
Oncel 2020 0.864 (0.806, 0.926)
Onwuzurike 2020 0.615 (0.400, 0.946)
Pecks 2020 0.132 (0.091, 0.191)
Pineles 2020 0.961 (0.919, 1.005)
Pu Yang 2020 0.714 (0.447, 1.141)
Qiancheng 2020 0.021 (0.001, 0.324)
Savasi 2020 0.158 (0.087, 0.288)
Sherer 2020 0.182 (0.075, 0.441)
Singh 2021 0.331 (0.257, 0.426)
Yan 2020 0.470 (0.382, 0.579)
Subgroup Poor quality (I^2=97.43 % , P=0.000) 0.309 (0.251, 0.379)
Anand 2020 0.203 (0.127, 0.324)
Cheng 2020 0.028 (0.002, 0.427)
Cruz−Lemini 2021 0.069 (0.040, 0.119)
Farghaly 2020 0.667 (0.467, 0.953)
Gale 2020 0.045 (0.015, 0.137)
Moreno 2020 0.619 (0.442, 0.866)
Ozsurmeli 2021 0.100 (0.016, 0.642)
Pierce−Williams 2020 0.636 (0.491, 0.824)
Prabhu 2020 0.178 (0.109, 0.292)
0.099 (0.059, 0.166)
Savirón−Cornudella 2020 0.071 (0.005, 1.032)
Saviron−Cornudella 2021 0.022 (0.001, 0.337)
Villalan 2020 0.054 (0.038, 0.077)
Vizheh 2021 0.294 (0.192, 0.450)
Wu 2020 0.621 (0.467, 0.825)
Subgroup Fair quality (I^2=94.54 % , P=0.000) 0.179 (0.102, 0.312)
Anuk 2021 0.016 (0.001, 0.252)
BRANDT 2020 0.869 (0.788, 0.958)
Martinez−Perez 2021 0.091 (0.061, 0.135)
Soto−Torres 2020 0.368 (0.287, 0.472)
Subgroup (I^2=98.06 % , P=0.000) 0.230 (0.077, 0.682)
Di Mascio 2020 0.259 (0.211, 0.318)
Daz−Corvillon 2020 0.027 (0.004, 0.187)
Dumitriu 2020 0.188 (0.125, 0.282)
Hcinia 2021 0.022 (0.007, 0.067)
PATBERG 2020 0.078 (0.036, 0.168)
Sahin 2020 0.300 (0.116, 0.773)
salvatore2020 0.146 (0.086, 0.247)
Vivanti 2020 0.278 (0.164, 0.470)
Wei Liu 2020 0.969 (0.887, 1.058)
Subgroup Good quality (I^2=97.42 % , P=0.000) 0.165 (0.078, 0.349)
Pereira 2020 0.080 (0.021, 0.302)
Subgroup Fair quality (I^2=NA , P=NA) 0.080 (0.021, 0.302)
Overall (I^2=97.45 % , P=0.000) 0.243 (0.205, 0.289)
0
0
0.01
0.01
0.02
0.05
0.1
0.2
0.51
1.02
Neonatal ICU admission rate with 95%

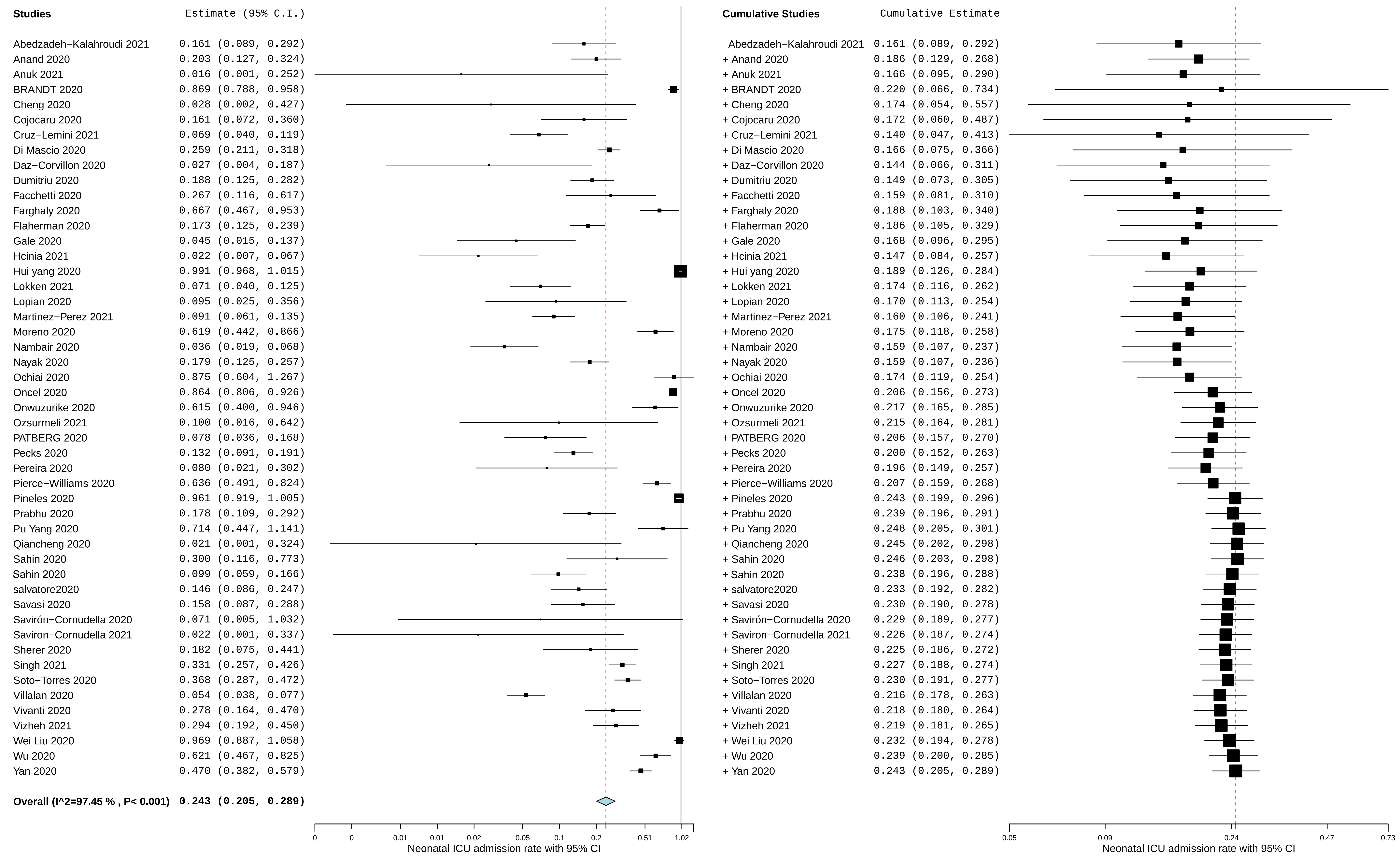
Studies
Estimate (95% C.I.)
Abedzadeh−Kalahroudi 2021 0.161 (0.089, 0.292)
Anand 2020 0.203 (0.127, 0.324)
Anuk 2021 0.016 (0.001, 0.252)
BRANDT 2020 0.869 (0.788, 0.958)
Cheng 2020 0.028 (0.002, 0.427)
Cojocaru 2020 0.161 (0.072, 0.360)
Cruz−Lemini 2021 0.069 (0.040, 0.119)
Di Mascio 2020 0.259 (0.211, 0.318)
Daz−Corvillon 2020 0.027 (0.004, 0.187)
Dumitriu 2020 0.188 (0.125, 0.282)
Facchetti 2020 0.267 (0.116, 0.617)
Farghaly 2020 0.667 (0.467, 0.953)
Flaherman 2020 0.173 (0.125, 0.239)
Gale 2020 0.045 (0.015, 0.137)
Hcinia 2021 0.022 (0.007, 0.067)
Hui yang 2020 0.991 (0.968, 1.015)
Lokken 2021 0.071 (0.040, 0.125)
Lopian 2020 0.095 (0.025, 0.356)
Martinez−Perez 2021 0.091 (0.061, 0.135)
Moreno 2020 0.619 (0.442, 0.866)
Nambair 2020 0.036 (0.019, 0.068)
Nayak 2020 0.179 (0.125, 0.257)
Ochiai 2020 0.875 (0.604, 1.267)
Oncel 2020 0.864 (0.806, 0.926)
Onwuzurike 2020 0.615 (0.400, 0.946)
Ozsurmeli 2021 0.100 (0.016, 0.642)
PATBERG 2020 0.078 (0.036, 0.168)
Pecks 2020 0.132 (0.091, 0.191)
Pereira 2020 0.080 (0.021, 0.302)
Pierce−Williams 2020 0.636 (0.491, 0.824)
Pineles 2020 0.961 (0.919, 1.005)
Prabhu 2020 0.178 (0.109, 0.292)
Pu Yang 2020 0.714 (0.447, 1.141)
Qiancheng 2020 0.021 (0.001, 0.324)
Sahin 2020 0.300 (0.116, 0.773)
Sahin 2020 0.099 (0.059, 0.166)
salvatore2020 0.146 (0.086, 0.247)
Savasi 2020 0.158 (0.087, 0.288)
Savirón−Cornudella 2020 0.071 (0.005, 1.032)
Saviron−Cornudella 2021 0.022 (0.001, 0.337)
Sherer 2020 0.182 (0.075, 0.441)
Singh 2021 0.331 (0.257, 0.426)
Soto−Torres 2020 0.368 (0.287, 0.472)
Villalan 2020 0.054 (0.038, 0.077)
Vivanti 2020 0.278 (0.164, 0.470)
Vizheh 2021 0.294 (0.192, 0.450)
Wei Liu 2020 0.969 (0.887, 1.058)
Wu 2020 0.621 (0.467, 0.825)
Yan 2020 0.470 (0.382, 0.579)
Overall (I^2=97.45 % , P< 0.001) 0.243 (0.205, 0.289)
0 0 0.01 0.01 0.02 0.05 0.1 0.2 0.51 1.02
Neonatal ICU admission rate with 95% CI
Cumulative Studies
Cumulative Estimate
Abedzadeh−Kalahroudi 2021 0.161 (0.089, 0.292)
+ Anand 2020 0.186 (0.129, 0.268)
+ Anuk 2021 0.166 (0.095, 0.290)
+ BRANDT 2020 0.220 (0.066, 0.734)
+ Cheng 2020 0.174 (0.054, 0.557)
+ Cojocaru 2020 0.172 (0.060, 0.487)
+ Cruz−Lemini 2021 0.140 (0.047, 0.413)
+ Di Mascio 2020 0.166 (0.075, 0.366)
+ Daz−Corvillon 2020 0.144 (0.066, 0.311)
+ Dumitriu 2020 0.149 (0.073, 0.305)
+ Facchetti 2020 0.159 (0.081, 0.310)
+ Farghaly 2020 0.188 (0.103, 0.340)
+ Flaherman 2020 0.186 (0.105, 0.329)
+ Gale 2020 0.168 (0.096, 0.295)
+ Hcinia 2021 0.147 (0.084, 0.257)
+ Hui yang 2020 0.189 (0.126, 0.284)
+ Lokken 2021 0.174 (0.116, 0.262)
+ Lopian 2020 0.170 (0.113, 0.254)
+ Martinez−Perez 2021 0.160 (0.106, 0.241)
+ Moreno 2020 0.175 (0.118, 0.258)
+ Nambair 2020 0.159 (0.107, 0.237)
+ Nayak 2020 0.159 (0.107, 0.236)
+ Ochiai 2020 0.174 (0.119, 0.254)
+ Oncel 2020 0.206 (0.156, 0.273)
+ Onwuzurike 2020 0.217 (0.165, 0.285)
+ Ozsurmeli 2021 0.215 (0.164, 0.281)
+ PATBERG 2020 0.206 (0.157, 0.270)
+ Pecks 2020 0.200 (0.152, 0.263)
+ Pereira 2020 0.196 (0.149, 0.257)
+ Pierce−Williams 2020 0.207 (0.159, 0.268)
+ Pineles 2020 0.243 (0.199, 0.296)
+ Prabhu 2020 0.239 (0.196, 0.291)
+ Pu Yang 2020 0.248 (0.205, 0.301)
+ Qiancheng 2020 0.245 (0.202, 0.298)
+ Sahin 2020 0.246 (0.203, 0.298)
+ Sahin 2020 0.238 (0.196, 0.288)
+ salvatore2020 0.233 (0.192, 0.282)
+ Savasi 2020 0.230 (0.190, 0.278)
+ Savirón−Cornudella 2020 0.229 (0.189, 0.277)
+ Saviron−Cornudella 2021 0.226 (0.187, 0.274)
+ Sherer 2020 0.225 (0.186, 0.272)
+ Singh 2021 0.227 (0.188, 0.274)
+ Soto−Torres 2020 0.230 (0.191, 0.277)
+ Villalan 2020 0.216 (0.178, 0.263)
+ Vivanti 2020 0.218 (0.180, 0.264)
+ Vizheh 2021 0.219 (0.181, 0.265)
+ Wei Liu 2020 0.232 (0.194, 0.278)
+ Wu 2020 0.239 (0.200, 0.285)
+ Yan 2020 0.243 (0.205, 0.289)
0.05 0.09 0.24 0.47 0.73
Neonatal ICU admission rate with 95% CI

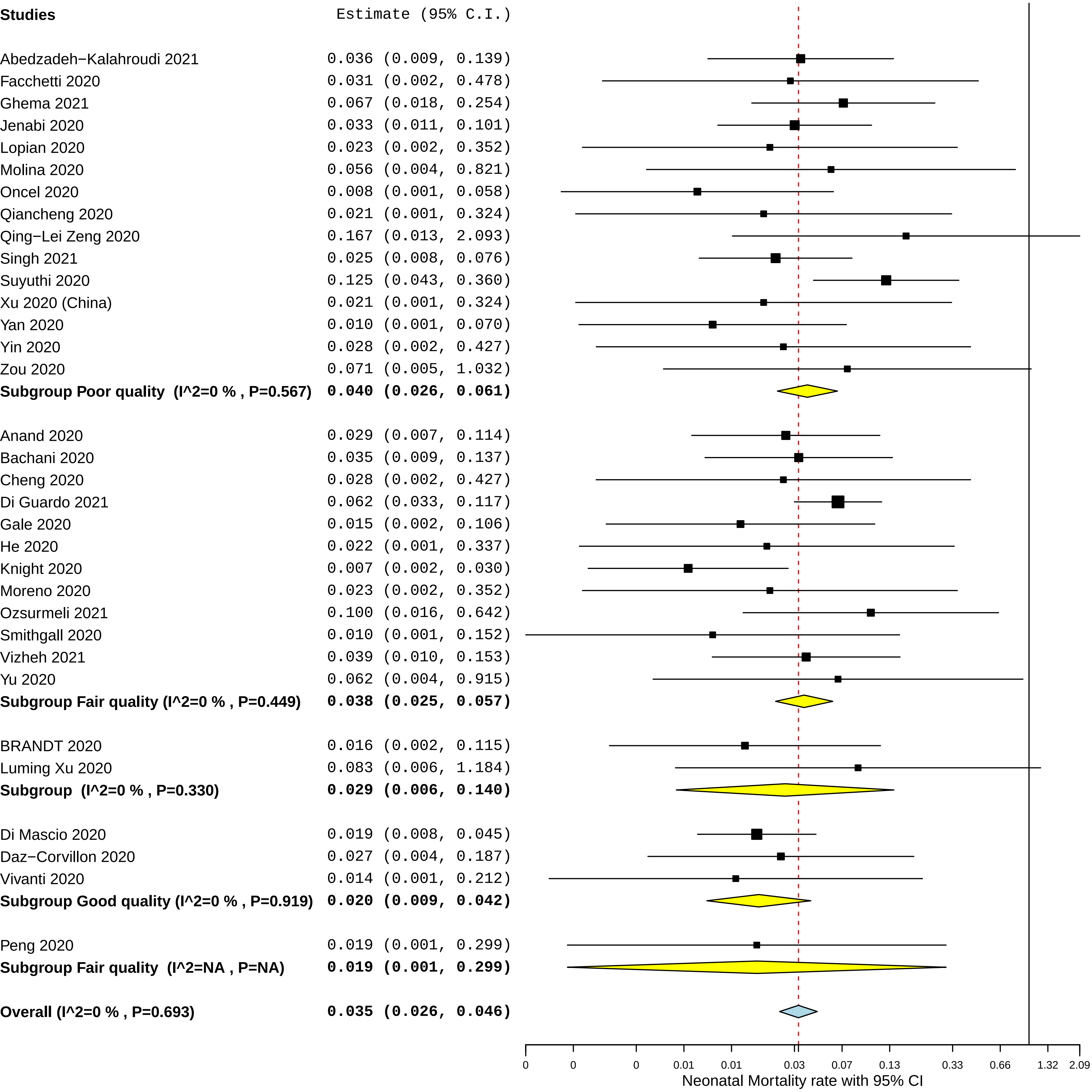

Studies
Estimate (95% C.I.)
Abedzadeh−Kalahroudi 2021 0.036 (0.009, 0.139)
Facchetti 2020 0.031 (0.002, 0.478)
Ghema 2021 0.067 (0.018, 0.254)
Jenabi 2020 0.033 (0.011, 0.101)
Lopian 2020 0.023 (0.002, 0.352)
Molina 2020 0.056 (0.004, 0.821)
Oncel 2020 0.008 (0.001, 0.058)
Qiancheng 2020 0.021 (0.001, 0.324)
Qing−Lei Zeng 2020 0.167 (0.013, 2.093)
Singh 2021 0.025 (0.008, 0.076)
Suyuthi 2020 0.125 (0.043, 0.360)
Xu 2020 (China) 0.021 (0.001, 0.324)
Yan 2020 0.010 (0.001, 0.070)
Yin 2020 0.028 (0.002, 0.427)
Zou 2020 0.071 (0.005, 1.032)
Subgroup Poor quality (I^2=0 % , P=0.567) 0.040 (0.026, 0.061)
Anand 2020 0.029 (0.007, 0.114)
Bachani 2020 0.035 (0.009, 0.137)
Cheng 2020 0.028 (0.002, 0.427)
Di Guardo 2021 0.062 (0.033, 0.117)
Gale 2020 0.015 (0.002, 0.106)
He 2020 0.022 (0.001, 0.337)
Knight 2020 0.007 (0.002, 0.030)
Moreno 2020 0.023 (0.002, 0.352)
Ozsurmeli 2021 0.100 (0.016, 0.642)
Smithgall 2020 0.010 (0.001, 0.152)
Vizheh 2021 0.039 (0.010, 0.153)
Yu 2020 0.062 (0.004, 0.915)
Subgroup Fair quality (I^2=0 % , P=0.449) 0.038 (0.025, 0.057)
BRANDT 2020 0.016 (0.002, 0.115)
Luming Xu 2020 0.083 (0.006, 1.184)
Subgroup (I^2=0 % , P=0.330) 0.029 (0.006, 0.140)
Di Mascio 2020 0.019 (0.008, 0.045)
Daz−Corvillon 2020 0.027 (0.004, 0.187)
Vivanti 2020 0.014 (0.001, 0.212)
Subgroup Good quality (I^2=0 % , P=0.919) 0.020 (0.009, 0.042)
Peng 2020 0.019 (0.001, 0.299)
Subgroup Fair quality (I^2=NA , P=NA) 0.019 (0.001, 0.299)
Overall (I^2=0 % , P=0.693) 0.035 (0.026, 0.046)
0 0 0 0.01 0.01 0.03 0.07 0.13 0.33 0.66 1.32 2.09
Neonatal Mortality rate with 95% CI

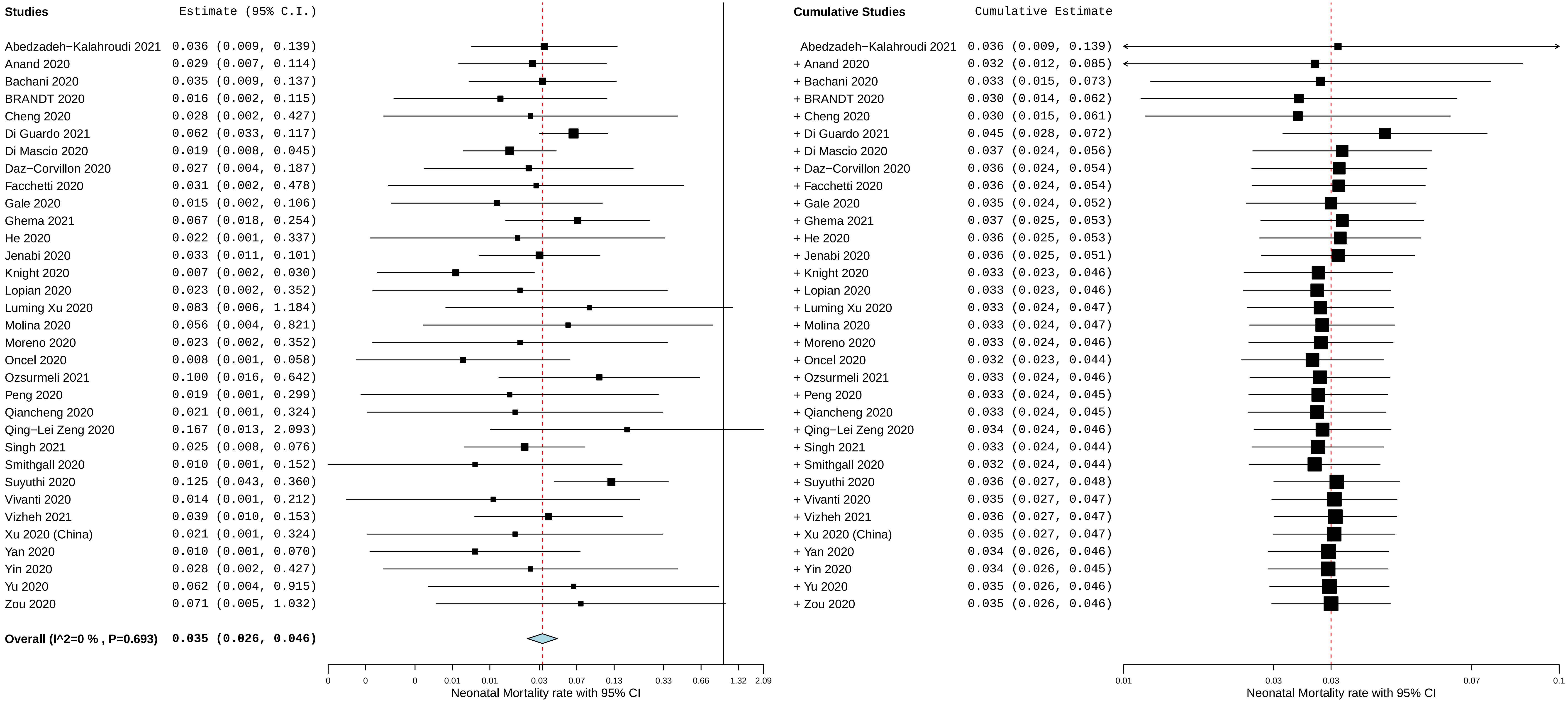
Studies
Estimate (95% C.I.)
Abedzadeh−Kalahroudi 2021 0.036 (0.009, 0.139)
Anand 2020 0.029 (0.007, 0.114)
Bachani 2020 0.035 (0.009, 0.137)
BRANDT 2020 0.016 (0.002, 0.115)
Cheng 2020 0.028 (0.002, 0.427)
Di Guardo 2021 0.062 (0.033, 0.117)
Di Mascio 2020 0.019 (0.008, 0.045)
Daz−Corvillon 2020 0.027 (0.004, 0.187)
Facchetti 2020 0.031 (0.002, 0.478)
Gale 2020 0.015 (0.002, 0.106)
Ghema 2021 0.067 (0.018, 0.254)
He 2020 0.022 (0.001, 0.337)
Jenabi 2020 0.033 (0.011, 0.101)
Knight 2020 0.007 (0.002, 0.030)
Lopian 2020 0.023 (0.002, 0.352)
Luming Xu 2020 0.083 (0.006, 1.184)
Molina 2020 0.056 (0.004, 0.821)
Moreno 2020 0.023 (0.002, 0.352)
Oncel 2020 0.008 (0.001, 0.058)
Ozsurmeli 2021 0.100 (0.016, 0.642)
Peng 2020 0.019 (0.001, 0.299)
Qiancheng 2020 0.021 (0.001, 0.324)
Qing−Lei Zeng 2020 0.167 (0.013, 2.093)
Singh 2021 0.025 (0.008, 0.076)
Smithgall 2020 0.010 (0.001, 0.152)
Suyuthi 2020 0.125 (0.043, 0.360)
Vivanti 2020 0.014 (0.001, 0.212)
Vizheh 2021 0.039 (0.010, 0.153)
Xu 2020 (China) 0.021 (0.001, 0.324)
Yan 2020 0.010 (0.001, 0.070)
Yin 2020 0.028 (0.002, 0.427)
Yu 2020 0.062 (0.004, 0.915)
Zou 2020 0.071 (0.005, 1.032)
Overall (I^2=0 % , P=0.693) 0.035 (0.026, 0.046)
0 0 0 0.01 0.01 0.03 0.07 0.13 0.33 0.66 1.32 2.09
Neonatal Mortality rate with 95% CI
Cumulative Studies
Cumulative Estimate
Abedzadeh−Kalahroudi 2021 0.036 (0.009, 0.139)
+ Anand 2020 0.032 (0.012, 0.085)
+ Bachani 2020 0.033 (0.015, 0.073)
+ BRANDT 2020 0.030 (0.014, 0.062)
+ Cheng 2020 0.030 (0.015, 0.061)
+ Di Guardo 2021 0.045 (0.028, 0.072)
+ Di Mascio 2020 0.037 (0.024, 0.056)
+ Daz−Corvillon 2020 0.036 (0.024, 0.054)
+ Facchetti 2020 0.036 (0.024, 0.054)
+ Gale 2020 0.035 (0.024, 0.052)
+ Ghema 2021 0.037 (0.025, 0.053)
+ He 2020 0.036 (0.025, 0.053)
+ Jenabi 2020 0.036 (0.025, 0.051)
+ Knight 2020 0.033 (0.023, 0.046)
+ Lopian 2020 0.033 (0.023, 0.046)
+ Luming Xu 2020 0.033 (0.024, 0.047)
+ Molina 2020 0.033 (0.024, 0.047)
+ Moreno 2020 0.033 (0.024, 0.046)
+ Oncel 2020 0.032 (0.023, 0.044)
+ Ozsurmeli 2021 0.033 (0.024, 0.046)
+ Peng 2020 0.033 (0.024, 0.045)
+ Qiancheng 2020 0.033 (0.024, 0.045)
+ Qing−Lei Zeng 2020 0.034 (0.024, 0.046)
+ Singh 2021 0.033 (0.024, 0.044)
+ Smithgall 2020 0.032 (0.024, 0.044)
+ Suyuthi 2020 0.036 (0.027, 0.048)
+ Vivanti 2020 0.035 (0.027, 0.047)
+ Vizheh 2021 0.036 (0.027, 0.047)
+ Xu 2020 (China) 0.035 (0.027, 0.047)
+ Yan 2020 0.034 (0.026, 0.046)
+ Yin 2020 0.034 (0.026, 0.045)
+ Yu 2020 0.035 (0.026, 0.046)
+ Zou 2020 0.035 (0.026, 0.046)
0.01 0.03 0.03 0.07 0.1
Neonatal Mortality rate with 95% CI

Studies	Estimate (95% C.I.)
Abedzadeh−Kalahroudi 2021	0.018 (0.003, 0.116)
Blitz 2020	0.028 (0.002, 0.322)
BRANDT 2020	0.023 (0.011, 0.049)
Campbell 2020	0.023 (0.002, 0.277)
Cribiù 2020	0.021 (0.002, 0.259)
Cruz−Lemini 2021	0.021 (0.003, 0.136)
Daz−Corvillon 2020	0.015 (0.008, 0.028)
Dumitriu 2020	0.014 (0.002, 0.090)
Facchetti 2020	0.013 (0.001, 0.178)
Flaherman 2020	0.012 (0.002, 0.078)
Gaspar 2021	0.011 (0.003, 0.045)
Gulersen 2020	0.009 (0.002, 0.035)
Hui Yang 2020	0.006 (0.001, 0.044)
Knight 2020	0.004 (0.001, 0.015)
**Subgroup Poor quality (I^2=0 % , P=0.892)**	**0.014 (0.010, 0.020)**
Ahlberg 2020	0.083 (0.017, 0.413)
Anand 2020	0.063 (0.007, 0.539)
Anuk 2021	0.051 (0.025, 0.103)
Bachani 2020	0.032 (0.012, 0.082)
Cohen 2020	0.022 (0.007, 0.067)
Cojocaru 2020	0.022 (0.002, 0.268)
Di Guardo 2021	0.021 (0.002, 0.259)
Farghaly 2020	0.013 (0.003, 0.050)
Gale 2020	0.012 (0.004, 0.036)
Ghema 2021	0.011 (0.004, 0.034)
Goyal 2020	0.011 (0.003, 0.041)
Hcinia 2021	0.008 (0.002, 0.031)
**Subgroup Fair quality (I^2=30.9 % , P=0.144)**	**0.022 (0.014, 0.034)**
Ajith 2021	0.071 (0.009, 0.577)
Badr 2020	0.031 (0.003, 0.350)
Barbero 2020	0.028 (0.002, 0.322)
Cheng 2020	0.022 (0.002, 0.268)
**Subgroup Good quality (I^2=0 % , P=0.895)**	**0.036 (0.011, 0.118)**
Antoun 2020	0.058 (0.023, 0.144)
Di Mascio 2020	0.019 (0.005, 0.072)
Handley 2020	0.008 (0.001, 0.116)
**Subgroup (I^2=36.62 % , P=0.206)**	**0.030 (0.011, 0.085)**
**Overall (I^2=8.04 % , P=0.336)**	**0.020 (0.016, 0.026)**

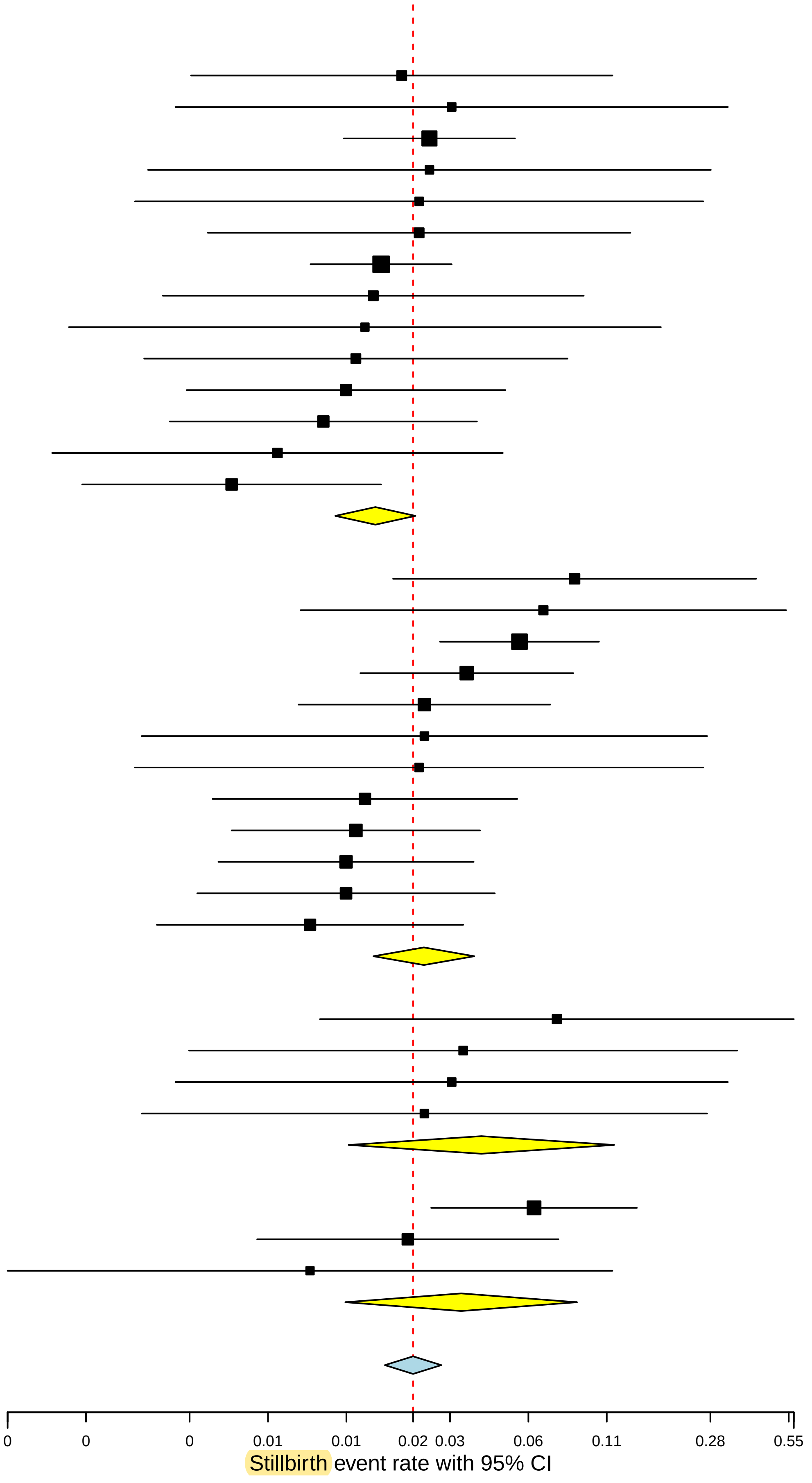

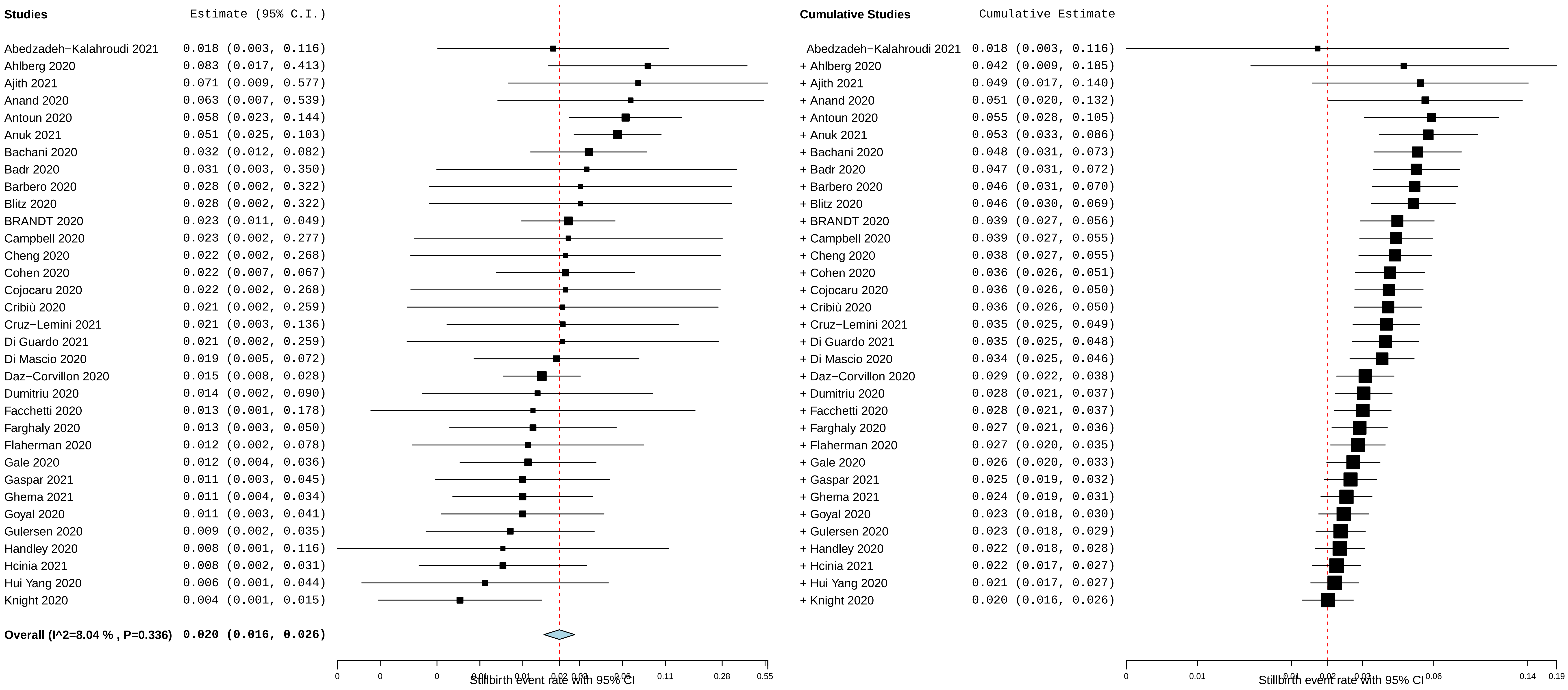
Studies
Estimate (95% C.I.)
Abedzadeh−Kalahroudi 2021 0.018 (0.003, 0.116)
Ahlberg 2020 0.083 (0.017, 0.413)
Ajith 2021 0.071 (0.009, 0.577)
Anand 2020 0.063 (0.007, 0.539)
Antoun 2020 0.058 (0.023, 0.144)
Anuk 2021 0.051 (0.025, 0.103)
Bachani 2020 0.032 (0.012, 0.082)
Badr 2020 0.031 (0.003, 0.350)
Barbero 2020 0.028 (0.002, 0.322)
Blitz 2020 0.028 (0.002, 0.322)
BRANDT 2020 0.023 (0.011, 0.049)
Campbell 2020 0.023 (0.002, 0.277)
Cheng 2020 0.022 (0.002, 0.268)
Cohen 2020 0.022 (0.007, 0.067)
Cojocaru 2020 0.022 (0.002, 0.268)
Cribiù 2020 0.021 (0.002, 0.259)
Cruz−Lemini 2021 0.021 (0.003, 0.136)
Di Guardo 2021 0.021 (0.002, 0.259)
Di Mascio 2020 0.019 (0.005, 0.072)
Daz−Corvillon 2020 0.015 (0.008, 0.028)
Dumitriu 2020 0.014 (0.002, 0.090)
Facchetti 2020 0.013 (0.001, 0.178)
Farghaly 2020 0.013 (0.003, 0.050)
Flaherman 2020 0.012 (0.002, 0.078)
Gale 2020 0.012 (0.004, 0.036)
Gaspar 2021 0.011 (0.003, 0.045)
Ghema 2021 0.011 (0.004, 0.034)
Goyal 2020 0.011 (0.003, 0.041)
Gulersen 2020 0.009 (0.002, 0.035)
Handley 2020 0.008 (0.001, 0.116)
Hcinia 2021 0.008 (0.002, 0.031)
Hui Yang 2020 0.006 (0.001, 0.044)
Knight 2020 0.004 (0.001, 0.015)
Overall (I^2=8.04 % , P=0.336) 0.020 (0.016, 0.026)
0 0 0 0.01 0.01 0.02 0.03 0.06 0.11 0.28 0.55
Stillbirth event rate with 95% CI
Cumulative Studies
Cumulative Estimate
Abedzadeh−Kalahroudi 2021 0.018 (0.003, 0.116)
+ Ahlberg 2020 0.042 (0.009, 0.185)
+ Ajith 2021 0.049 (0.017, 0.140)
+ Anand 2020 0.051 (0.020, 0.132)
+ Antoun 2020 0.055 (0.028, 0.105)
+ Anuk 2021 0.053 (0.033, 0.086)
+ Bachani 2020 0.048 (0.031, 0.073)
+ Badr 2020 0.047 (0.031, 0.072)
+ Barbero 2020 0.046 (0.031, 0.070)
+ Blitz 2020 0.046 (0.030, 0.069)
+ BRANDT 2020 0.039 (0.027, 0.056)
+ Campbell 2020 0.039 (0.027, 0.055)
+ Cheng 2020 0.038 (0.027, 0.055)
+ Cohen 2020 0.036 (0.026, 0.051)
+ Cojocaru 2020 0.036 (0.026, 0.050)
+ Cribiù 2020 0.036 (0.026, 0.050)
+ Cruz−Lemini 2021 0.035 (0.025, 0.049)
+ Di Guardo 2021 0.035 (0.025, 0.048)
+ Di Mascio 2020 0.034 (0.025, 0.046)
+ Daz−Corvillon 2020 0.029 (0.022, 0.038)
+ Dumitriu 2020 0.028 (0.021, 0.037)
+ Facchetti 2020 0.028 (0.021, 0.037)
+ Farghaly 2020 0.027 (0.021, 0.036)
+ Flaherman 2020 0.027 (0.020, 0.035)
+ Gale 2020 0.026 (0.020, 0.033)
+ Gaspar 2021 0.025 (0.019, 0.032)
+ Ghema 2021 0.024 (0.019, 0.031)
+ Goyal 2020 0.023 (0.018, 0.030)
+ Gulersen 2020 0.023 (0.018, 0.029)
+ Handley 2020 0.022 (0.018, 0.028)
+ Hcinia 2021 0.022 (0.017, 0.027)
+ Hui Yang 2020 0.021 (0.017, 0.027)
+ Knight 2020 0.020 (0.016, 0.026)
0 0.01 0.01 0.02 0.03 0.06 0.14 0.19
Stillbirth event rate with 95% CI

Studies	Estimate (95% C.I.)
Antoun 2020	0.062 (0.004, 0.915)
Campbell 2020	0.016 (0.001, 0.252)
Cojocaru 2020	0.016 (0.001, 0.244)
Cribiù 2020	0.023 (0.002, 0.352)
Facchetti 2020	0.067 (0.010, 0.443)
Flaherman 2020	0.011 (0.003, 0.044)
Gaspar 2021	0.038 (0.002, 0.583)
Ghema 2021	0.033 (0.005, 0.229)
Hui Yang 2020	0.020 (0.001, 0.311)
Hui yang 2020	0.024 (0.002, 0.368)
Llorca 2021	0.033 (0.002, 0.509)
Lokken 2021	0.011 (0.001, 0.171)
Molina 2020	0.056 (0.004, 0.821)
Ochiai 2020	0.125 (0.009, 1.671)
Onwuzurike 2020	0.050 (0.003, 0.745)
Pineles 2020	0.014 (0.002, 0.099)
Pu Yang 2020	0.062 (0.004, 0.915)
Qiancheng 2020	0.021 (0.001, 0.324)
Qing−Lei Zeng 2020	0.167 (0.013, 2.093)
Santana 2021	0.031 (0.004, 0.215)
Savasi 2020	0.070 (0.027, 0.181)
Schwartz 2020	0.091 (0.024, 0.341)
Singh 2021	0.017 (0.004, 0.065)
Suyuthi 2020	0.208 (0.095, 0.454)
Xu 2020 (China)	0.021 (0.001, 0.324)
Yin 2020	0.028 (0.002, 0.427)
Yingchun Zeng 2020	0.029 (0.002, 0.451)
Zou 2020	0.071 (0.005, 1.032)
**Subgroup Poor quality (I^2=8.31 % , P=0.340)**	**0.047 (0.032, 0.070)**
Barbero 2020	0.020 (0.001, 0.311)
Cheng 2020	0.028 (0.002, 0.427)
Di Guardo 2021	0.048 (0.023, 0.099)
Farghaly 2020	0.067 (0.010, 0.443)
Gale 2020	0.030 (0.008, 0.119)
Goyal 2020	0.062 (0.004, 0.915)
He 2020	0.022 (0.001, 0.337)
Knight 2020	0.045 (0.026, 0.079)
Lu Zhang 2020	0.026 (0.002, 0.406)
Mattar 2020	0.083 (0.006, 1.184)
Mattern 2021	0.024 (0.002, 0.368)
Moreno 2020	0.023 (0.002, 0.352)
Ozsurmeli 2021	0.045 (0.003, 0.682)
Santhosh 2021	0.250 (0.046, 1.365)
Savirón−Cornudella 2020	0.071 (0.005, 1.032)
Saviron−Cornudella 2021	0.022 (0.001, 0.337)
Shmakov 2020	0.012 (0.001, 0.183)
Smithgall 2020	0.010 (0.001, 0.152)
Tug 2020	0.008 (0.000, 0.130)
Vintzileos 2020	0.017 (0.001, 0.260)
Vizheh 2021	0.157 (0.083, 0.296)
Wu 2020	0.172 (0.077, 0.383)
Yu 2020	0.333 (0.067, 1.652)
**Subgroup Fair quality (I^2=33.62 % , P=0.060)**	**0.061 (0.040, 0.093)**
Di Mascio 2020	0.004 (0.001, 0.027)
Daz−Corvillon 2020	0.054 (0.014, 0.208)
Dumitriu 2020	0.020 (0.005, 0.078)
Hcinia 2021	0.029 (0.011, 0.077)
Rong Yang 2020	0.013 (0.001, 0.201)
Sahin 2020	0.045 (0.003, 0.682)
San−juan 2020	0.071 (0.005, 1.032)
Vivanti 2020	0.028 (0.004, 0.192)
Wei Liu 2020	0.031 (0.002, 0.478)
**Subgroup Good quality (I^2=0 % , P=0.650)**	**0.026 (0.015, 0.046)**
Luming Xu 2020	0.083 (0.006, 1.184)
Martinez−Perez 2021	0.016 (0.005, 0.049)
**Subgroup (I^2=20.12 % , P=0.263)**	**0.023 (0.006, 0.088)**
Peng 2020	0.019 (0.001, 0.299)
Pereira 2020	0.019 (0.001, 0.299)
**Subgroup Fair quality (I^2=0 % , P=1.000)**	**0.019 (0.003, 0.133)**
**Overall (I^2=24.75 % , P=0.042)**	**0.045 (0.034, 0.058)**

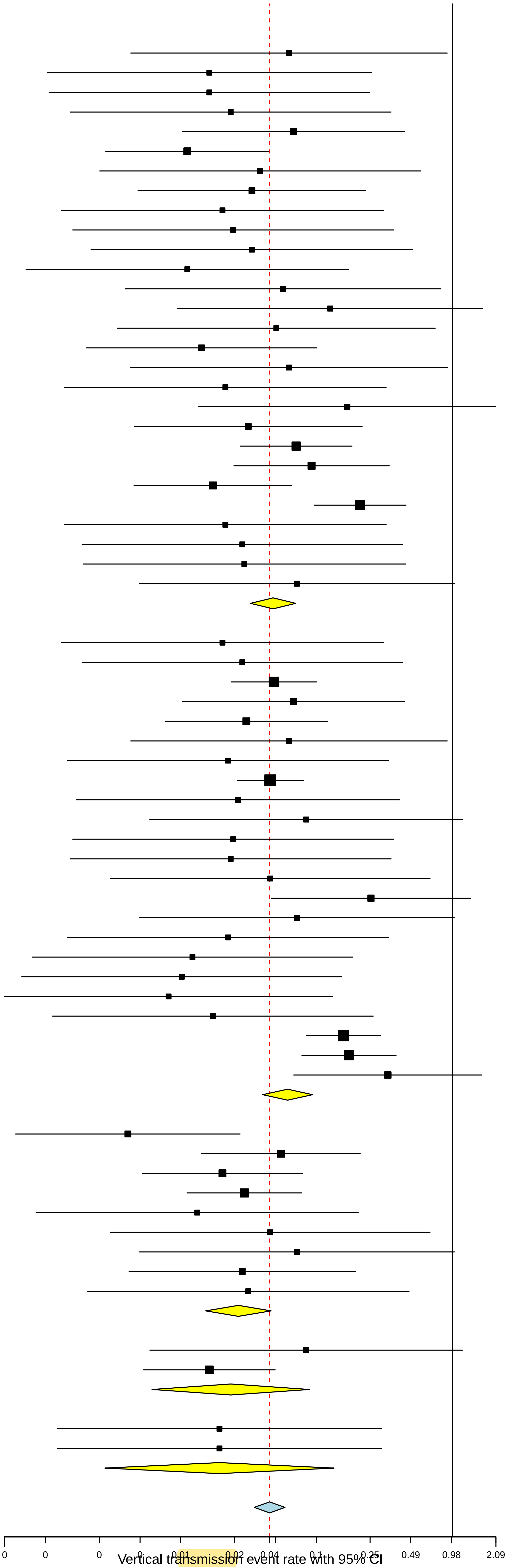

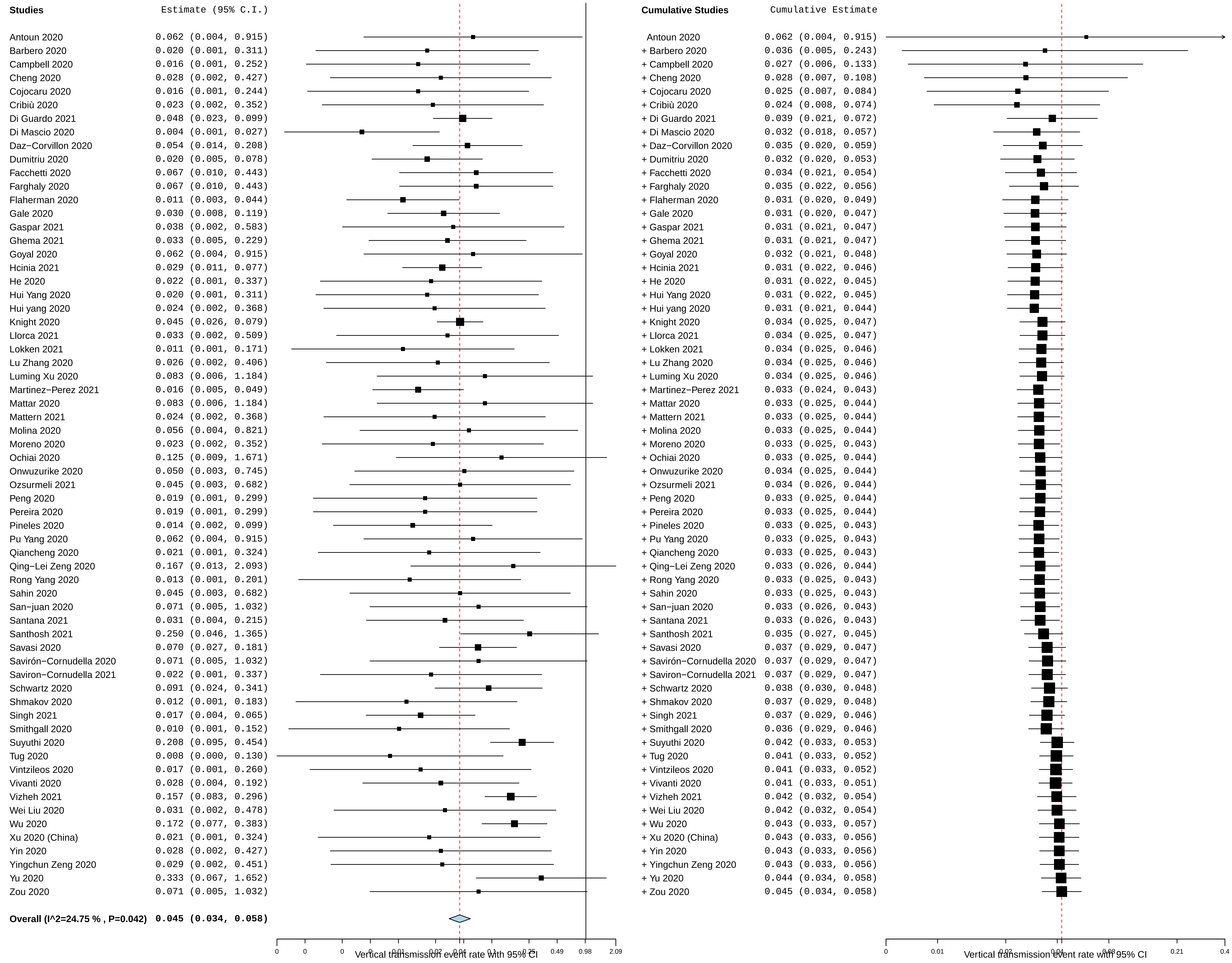
Studies
Estimate (95% C.I.)
Antoun 2020 0.062 (0.004, 0.915)
Barbero 2020 0.020 (0.001, 0.311)
Campbell 2020 0.016 (0.001, 0.252)
Cheng 2020 0.028 (0.002, 0.427)
Cojocaru 2020 0.016 (0.001, 0.244)
Cribiù 2020 0.023 (0.002, 0.352)
Di Guardo 2021 0.048 (0.023, 0.099)
Di Mascio 2020 0.004 (0.001, 0.027)
Daz−Corvillon 2020 0.054 (0.014, 0.208)
Dumitriu 2020 0.020 (0.005, 0.078)
Facchetti 2020 0.067 (0.010, 0.443)
Farghaly 2020 0.067 (0.010, 0.443)
Flaherman 2020 0.011 (0.003, 0.044)
Gale 2020 0.030 (0.008, 0.119)
Gaspar 2021 0.038 (0.002, 0.583)
Ghema 2021 0.033 (0.005, 0.229)
Goyal 2020 0.062 (0.004, 0.915)
Hcinia 2021 0.029 (0.011, 0.077)
He 2020 0.022 (0.001, 0.337)
Hui Yang 2020 0.020 (0.001, 0.311)
Hui yang 2020 0.024 (0.002, 0.368)
Knight 2020 0.045 (0.026, 0.079)
Llorca 2021 0.033 (0.002, 0.509)
Lokken 2021 0.011 (0.001, 0.171)
Lu Zhang 2020 0.026 (0.002, 0.406)
Luming Xu 2020 0.083 (0.006, 1.184)
Martinez−Perez 2021 0.016 (0.005, 0.049)
Mattar 2020 0.083 (0.006, 1.184)
Mattern 2021 0.024 (0.002, 0.368)
Molina 2020 0.056 (0.004, 0.821)
Moreno 2020 0.023 (0.002, 0.352)
Ochiai 2020 0.125 (0.009, 1.671)
Onwuzurike 2020 0.050 (0.003, 0.745)
Ozsurmeli 2021 0.045 (0.003, 0.682)
Peng 2020 0.019 (0.001, 0.299)
Pereira 2020 0.019 (0.001, 0.299)
Pineles 2020 0.014 (0.002, 0.099)
Pu Yang 2020 0.062 (0.004, 0.915)
Qiancheng 2020 0.021 (0.001, 0.324)
Qing−Lei Zeng 2020 0.167 (0.013, 2.093)
Rong Yang 2020 0.013 (0.001, 0.201)
Sahin 2020 0.045 (0.003, 0.682)
San−juan 2020 0.071 (0.005, 1.032)
Santana 2021 0.031 (0.004, 0.215)
Santhosh 2021 0.250 (0.046, 1.365)
Savasi 2020 0.070 (0.027, 0.181)
Savirón−Cornudella 2020 0.071 (0.005, 1.032)
Saviron−Cornudella 2021 0.022 (0.001, 0.337)
Schwartz 2020 0.091 (0.024, 0.341)
Shmakov 2020 0.012 (0.001, 0.183)
Singh 2021 0.017 (0.004, 0.065)
Smithgall 2020 0.010 (0.001, 0.152)
Suyuthi 2020 0.208 (0.095, 0.454)
Tug 2020 0.008 (0.000, 0.130)
Vintzileos 2020 0.017 (0.001, 0.260)
Vivanti 2020 0.028 (0.004, 0.192)
Vizheh 2021 0.157 (0.083, 0.296)
Wei Liu 2020 0.031 (0.002, 0.478)
Wu 2020 0.172 (0.077, 0.383)
Xu 2020 (China) 0.021 (0.001, 0.324)
Yin 2020 0.028 (0.002, 0.427)
Yingchun Zeng 2020 0.029 (0.002, 0.451)
Yu 2020 0.333 (0.067, 1.652)
Zou 2020 0.071 (0.005, 1.032)
Overall (I^2=24.75 % , P=0.042) 0.045 (0.034, 0.058)
Vertical transmission event rate with 95% CI
Cumulative Studies
Cumulative Estimate
Antoun 2020 0.062 (0.004, 0.915)
+ Barbero 2020 0.036 (0.005, 0.243)
+ Campbell 2020 0.027 (0.006, 0.133)
+ Cheng 2020 0.028 (0.007, 0.108)
+ Cojocaru 2020 0.025 (0.007, 0.084)
+ Cribiù 2020 0.024 (0.008, 0.074)
+ Di Guardo 2021 0.039 (0.021, 0.072)
+ Di Mascio 2020 0.032 (0.018, 0.057)
+ Daz−Corvillon 2020 0.035 (0.020, 0.059)
+ Dumitriu 2020 0.032 (0.020, 0.053)
+ Facchetti 2020 0.034 (0.021, 0.054)
+ Farghaly 2020 0.035 (0.022, 0.056)
+ Flaherman 2020 0.031 (0.020, 0.049)
+ Gale 2020 0.031 (0.020, 0.047)
+ Gaspar 2021 0.031 (0.021, 0.047)
+ Ghema 2021 0.031 (0.021, 0.047)
+ Goyal 2020 0.032 (0.021, 0.048)
+ Hcinia 2021 0.031 (0.022, 0.046)
+ He 2020 0.031 (0.022, 0.045)
+ Hui Yang 2020 0.031 (0.022, 0.045)
+ Hui yang 2020 0.031 (0.021, 0.044)
+ Knight 2020 0.034 (0.025, 0.047)
+ Llorca 2021 0.034 (0.025, 0.047)
+ Lokken 2021 0.034 (0.025, 0.046)
+ Lu Zhang 2020 0.034 (0.025, 0.046)
+ Luming Xu 2020 0.034 (0.025, 0.046)
+ Martinez−Perez 2021 0.033 (0.024, 0.043)
+ Mattar 2020 0.033 (0.025, 0.044)
+ Mattern 2021 0.033 (0.025, 0.044)
+ Molina 2020 0.033 (0.025, 0.044)
+ Moreno 2020 0.033 (0.025, 0.043)
+ Ochiai 2020 0.033 (0.025, 0.044)
+ Onwuzurike 2020 0.034 (0.025, 0.044)
+ Ozsurmeli 2021 0.034 (0.026, 0.044)
+ Peng 2020 0.033 (0.025, 0.044)
+ Pereira 2020 0.033 (0.025, 0.044)
+ Pineles 2020 0.033 (0.025, 0.043)
+ Pu Yang 2020 0.033 (0.025, 0.043)
+ Qiancheng 2020 0.033 (0.025, 0.043)
+ Qing−Lei Zeng 2020 0.033 (0.026, 0.044)
+ Rong Yang 2020 0.033 (0.025, 0.043)
+ Sahin 2020 0.033 (0.025, 0.043)
+ San−juan 2020 0.033 (0.026, 0.043)
+ Santana 2021 0.033 (0.026, 0.043)
+ Santhosh 2021 0.035 (0.027, 0.045)
+ Savasi 2020 0.037 (0.029, 0.047)
+ Savirón−Cornudella 2020 0.037 (0.029, 0.047)
+ Saviron−Cornudella 2021 0.037 (0.029, 0.047)
+ Schwartz 2020 0.038 (0.030, 0.048)
+ Shmakov 2020 0.037 (0.029, 0.048)
+ Singh 2021 0.037 (0.029, 0.046)
+ Smithgall 2020 0.036 (0.029, 0.046)
+ Suyuthi 2020 0.042 (0.033, 0.053)
+ Tug 2020 0.041 (0.033, 0.052)
+ Vintzileos 2020 0.041 (0.033, 0.052)
+ Vivanti 2020 0.041 (0.033, 0.051)
+ Vizheh 2021 0.042 (0.032, 0.054)
+ Wei Liu 2020 0.042 (0.032, 0.054)
+ Wu 2020 0.043 (0.033, 0.057)
+ Xu 2020 (China) 0.043 (0.033, 0.056)
+ Yin 2020 0.043 (0.033, 0.056)
+ Yingchun Zeng 2020 0.043 (0.033, 0.056)
+ Yu 2020 0.044 (0.034, 0.058)
+ Zou 2020 0.045 (0.034, 0.058)
Vertical transmission event rate with 95% CI

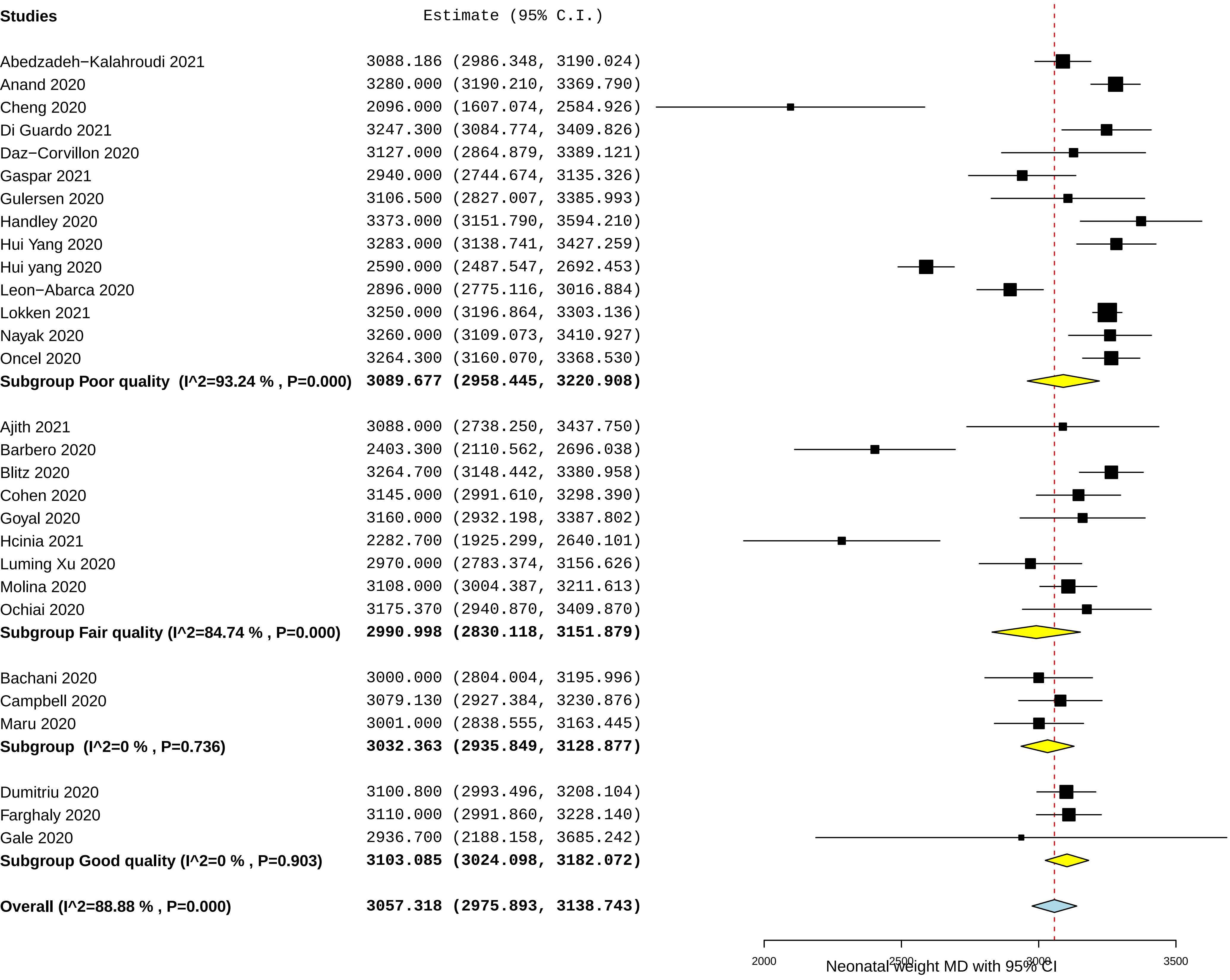
Studies
Estimate (95% C.I.)
Abedzadeh−Kalahroudi 2021 3088.186 (2986.348, 3190.024)
Anand 2020 3280.000 (3190.210, 3369.790)
Cheng 2020 2096.000 (1607.074, 2584.926)
Di Guardo 2021 3247.300 (3084.774, 3409.826)
Daz−Corvillon 2020 3127.000 (2864.879, 3389.121)
Gaspar 2021 2940.000 (2744.674, 3135.326)
Gulersen 2020 3106.500 (2827.007, 3385.993)
Handley 2020 3373.000 (3151.790, 3594.210)
Hui Yang 2020 3283.000 (3138.741, 3427.259)
Hui yang 2020 2590.000 (2487.547, 2692.453)
Leon−Abarca 2020 2896.000 (2775.116, 3016.884)
Lokken 2021 3250.000 (3196.864, 3303.136)
Nayak 2020 3260.000 (3109.073, 3410.927)
Oncel 2020 3264.300 (3160.070, 3368.530)
Subgroup Poor quality (I^2=93.24 % , P=0.000) 3089.677 (2958.445, 3220.908)
Ajith 2021 3088.000 (2738.250, 3437.750)
Barbero 2020 2403.300 (2110.562, 2696.038)
Blitz 2020 3264.700 (3148.442, 3380.958)
Cohen 2020 3145.000 (2991.610, 3298.390)
Goyal 2020 3160.000 (2932.198, 3387.802)
Hcinia 2021 2282.700 (1925.299, 2640.101)
Luming Xu 2020 2970.000 (2783.374, 3156.626)
Molina 2020 3108.000 (3004.387, 3211.613)
Ochiai 2020 3175.370 (2940.870, 3409.870)
Subgroup Fair quality (I^2=84.74 % , P=0.000) 2990.998 (2830.118, 3151.879)
Bachani 2020 3000.000 (2804.004, 3195.996)
Campbell 2020 3079.130 (2927.384, 3230.876)
Maru 2020 3001.000 (2838.555, 3163.445)
Subgroup (I^2=0 % , P=0.736) 3032.363 (2935.849, 3128.877)
Dumitriu 2020 3100.800 (2993.496, 3208.104)
Farghaly 2020 3110.000 (2991.860, 3228.140)
Gale 2020 2936.700 (2188.158, 3685.242)
Subgroup Good quality (I^2=0 % , P=0.903) 3103.085 (3024.098, 3182.072)
Overall (I^2=88.88 % , P=0.000) 3057.318 (2975.893, 3138.743)
2000
2500
3000
3500
Neonatal weight MD with 95% CI

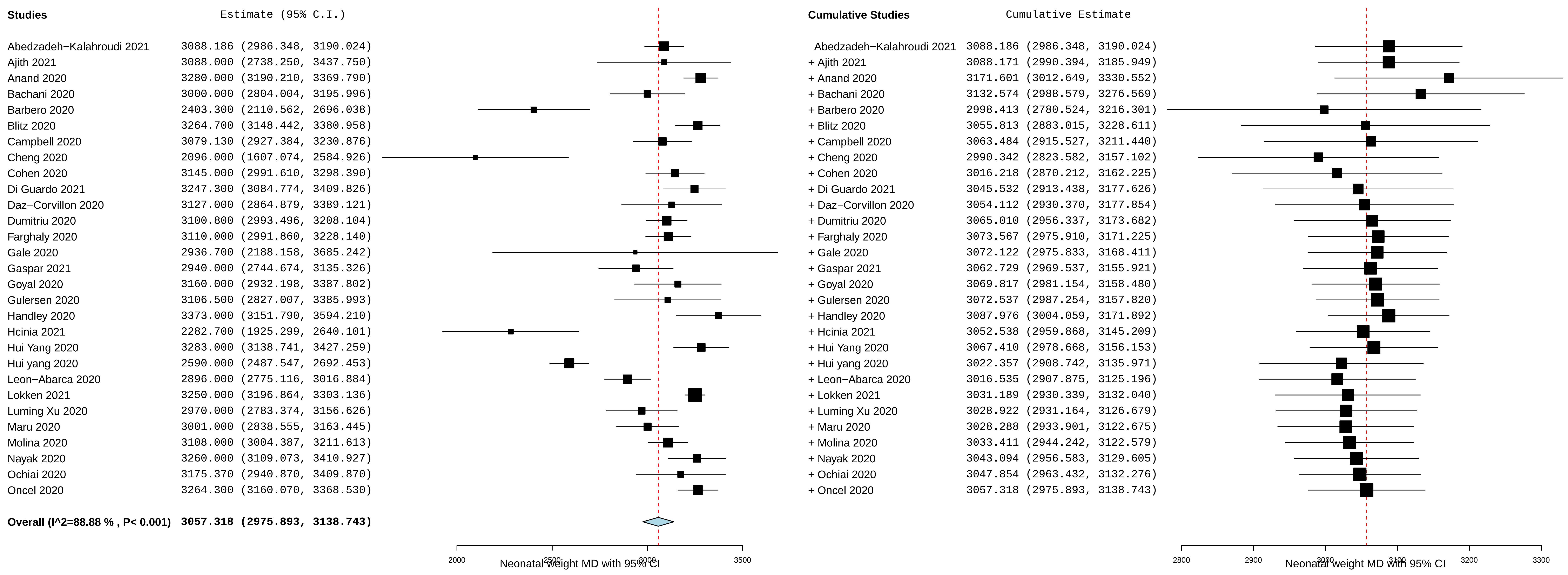
Studies
Estimate (95% C.I.)
Abedzadeh−Kalahroudi 2021 3088.186 (2986.348, 3190.024)
Ajith 2021 3088.000 (2738.250, 3437.750)
Anand 2020 3280.000 (3190.210, 3369.790)
Bachani 2020 3000.000 (2804.004, 3195.996)
Barbero 2020 2403.300 (2110.562, 2696.038)
Blitz 2020 3264.700 (3148.442, 3380.958)
Campbell 2020 3079.130 (2927.384, 3230.876)
Cheng 2020 2096.000 (1607.074, 2584.926)
Cohen 2020 3145.000 (2991.610, 3298.390)
Di Guardo 2021 3247.300 (3084.774, 3409.826)
Daz−Corvillon 2020 3127.000 (2864.879, 3389.121)
Dumitriu 2020 3100.800 (2993.496, 3208.104)
Farghaly 2020 3110.000 (2991.860, 3228.140)
Gale 2020 2936.700 (2188.158, 3685.242)
Gaspar 2021 2940.000 (2744.674, 3135.326)
Goyal 2020 3160.000 (2932.198, 3387.802)
Gulersen 2020 3106.500 (2827.007, 3385.993)
Handley 2020 3373.000 (3151.790, 3594.210)
Hcinia 2021 2282.700 (1925.299, 2640.101)
Hui Yang 2020 3283.000 (3138.741, 3427.259)
Hui yang 2020 2590.000 (2487.547, 2692.453)
Leon−Abarca 2020 2896.000 (2775.116, 3016.884)
Lokken 2021 3250.000 (3196.864, 3303.136)
Luming Xu 2020 2970.000 (2783.374, 3156.626)
Maru 2020 3001.000 (2838.555, 3163.445)
Molina 2020 3108.000 (3004.387, 3211.613)
Nayak 2020 3260.000 (3109.073, 3410.927)
Ochiai 2020 3175.370 (2940.870, 3409.870)
Oncel 2020 3264.300 (3160.070, 3368.530)
Overall (I^2=88.88 % , P< 0.001) 3057.318 (2975.893, 3138.743)
2000
2500
3000
3500
Neonatal weight MD with 95% CI
Cumulative Studies
Cumulative Estimate
Abedzadeh−Kalahroudi 2021 3088.186 (2986.348, 3190.024)
+ Ajith 2021 3088.171 (2990.394, 3185.949)
+ Anand 2020 3171.601 (3012.649, 3330.552)
+ Bachani 2020 3132.574 (2988.579, 3276.569)
+ Barbero 2020 2998.413 (2780.524, 3216.301)
+ Blitz 2020 3055.813 (2883.015, 3228.611)
+ Campbell 2020 3063.484 (2915.527, 3211.440)
+ Cheng 2020 2990.342 (2823.582, 3157.102)
+ Cohen 2020 3016.218 (2870.212, 3162.225)
+ Di Guardo 2021 3045.532 (2913.438, 3177.626)
+ Daz−Corvillon 2020 3054.112 (2930.370, 3177.854)
+ Dumitriu 2020 3065.010 (2956.337, 3173.682)
+ Farghaly 2020 3073.567 (2975.910, 3171.225)
+ Gale 2020 3072.122 (2975.833, 3168.411)
+ Gaspar 2021 3062.729 (2969.537, 3155.921)
+ Goyal 2020 3069.817 (2981.154, 3158.480)
+ Gulersen 2020 3072.537 (2987.254, 3157.820)
+ Handley 2020 3087.976 (3004.059, 3171.892)
+ Hcinia 2021 3052.538 (2959.868, 3145.209)
+ Hui Yang 2020 3067.410 (2978.668, 3156.153)
+ Hui yang 2020 3022.357 (2908.742, 3135.971)
+ Leon−Abarca 2020 3016.535 (2907.875, 3125.196)
+ Lokken 2021 3031.189 (2930.339, 3132.040)
+ Luming Xu 2020 3028.922 (2931.164, 3126.679)
+ Maru 2020 3028.288 (2933.901, 3122.675)
+ Molina 2020 3033.411 (2944.242, 3122.579)
+ Nayak 2020 3043.094 (2956.583, 3129.605)
+ Ochiai 2020 3047.854 (2963.432, 3132.276)
+ Oncel 2020 3057.318 (2975.893, 3138.743)
2800
2900
3000
3100
3200
3300
Neonatal weight MD with 95% CI
