# Supplementary material for: Systematic review and meta-analysis of COVID-19 maternal and neonatal clinical features and pregnancy outcomes up to June 3, 2021
Source: AJOG Glob Rep. 2022 Jan 3;2(1):100049. doi: 10.1016/j.xagr.2021.100049 (PMC8720679; doi:10.1016/j.xagr.2021.100049)
Supplement: Supplementary file 5 [file mmc5.docx]

**Table S1.** Risk of bias assessment of the cohort studies using the NIH tool.

| **Studies** | **1. Was the research question or objective in this paper clearly stated?** | **2. Was the study population clearly specified and defined?** | **3. Was the participation rate of eligible persons at least 50%?** | **4. Were all the subjects selected or recruited from the same or similar populations? Were inclusion and exclusion criteria for being in the study pre-specified and applied uniformly to all participants?** | **5. Was a sample size justification, power description, or variance and effect estimates provided?** | **6. For the analyses in this paper, were the exposure(s) of interest measured prior to the outcome(s) being measured?** | **7. Was the time frame sufficient so that one could reasonably expect to see an association between exposure and outcome if it existed?** | **8. For exposures that can vary in amount or level, did the study examine different levels of the exposure as related to the outcome (e.g., categories of exposure, or exposure measured as continuous variable)?** | **9. Were the exposure measures (independent variables) clearly defined, valid, reliable, and implemented consistently across all study participants?** | **10. Was the exposure(s) assessed more than once over time?** | **11. Were the outcome measures (dependent variables) clearly defined, valid, reliable, and implemented consistently across all study participants?** | **12. Were the outcome assessors blinded to the exposure status of participants?** | **13. Was loss to follow-up after baseline 20% or less?** | **14. Were key potential confounding variables measured and adjusted statistically for their impact on the relationship between exposure(s) an outcome(s)?** | **Total scores** (Yes = 1, No = 0.5, NR & NA & CD = 0) | **Quality rating:** good (9-14 point) or fair (7-8 point) or poor (0-7 points) |
| --- | --- | --- | --- | --- | --- | --- | --- | --- | --- | --- | --- | --- | --- | --- | --- | --- |
| Ajith 2021 | Yes | Yes | Yes | Yes | No | Yes | Yes | NA | NA | NA | No | No | Yes | No | 9 | Good quality |
| Kalahroudi 2021 | Yes | Yes | NR | Yes | NR | Yes | Yes | NA | NA | NA | No | No | Yes | No | 7.5 | Poor quality |
| Ahlberg 2020 | Yes | No | Yes | Yes | NR | Yes | Yes | NA | NA | NA | NR | No | Yes | Yes | 8 | Fair quality |
| Anand 2020 | Yes | Yes | Yes | Yes | NR | Yes | Yes | NA | NA | NA | No | No | Yes | No | 8.5 | Fair quality |
| Antoun 202 | Yes | Yes | NR | Yes | No | Yes | Yes | NA | NA | NA | No | No | Yes | NR | 7.5 | Poor quality |
| Bachani 2020 | Yes | Yes | Yes | Yes | NR | Yes | Yes | NA | NA | NA | No | No | Yes | No | 8.5 | Fair quality |
| Barbero 220 | Yes | Yes | Yes | Yes | NR | Yes | Yes | NA | NA | NA | NR | No | Yes | No | 8 | Fair quality |
| Blitz 2020 | Yes | No | NR | Yes | NR | Yes | Yes | NA | NA | NA | NR | No | Yes | NR | 6 | Poor quality |
| Cribiu 2020 | Yes | Yes | NR | Yes | NR | Yes | Yes | NA | NA | NA | No | No | Yes | NR | 7 | Poor quality |
| Campbell 2020 | Yes | No | NR | Yes | NR | Yes | Yes | NA | NA | NA | No | No | Yes | NR | 6.5 | Poor quality |
| Cohen 2020 | Yes | No | NR | Yes | NR | Yes | Yes | NA | NA | NA | NR | No | Yes | NR | 6 | Poor quality |
| Cheng 2020 | Yes | Yes | Yes | Yes | No | Yes | Yes | NA | NA | NA | NR | No | Yes | No | 8.5 | Fair quality |
| Cojocaru 2020 | Yes | Yes | NR | Yes | NR | Yes | Yes | NA | NA | NA | No | No | Yes | No | 7.5 | Poor quality |
| Lemini 2021 | Yes | Yes | Yes | Yes | NR | Yes | Yes | NA | NA | NA | NR | No | Yes | Yes | 8.5 | Fair quality |
| Mascio 2020 | Yes | Yes | Yes | Yes | Yes | Yes | Yes | NA | NA | NA | No | No | Yes | Yes | 10 | Good quality |
| Corvillo´ 2020 | Yes | Yes | Yes | Yes | Yes | Yes | Yes | NA | NA | NA | No | No | Yes | Yes | 10 | Good quality |
| Guardo 2021 | Yes | Yes | NR | Yes | Yes | Yes | Yes | NA | NA | NA | NR | No | Yes | No | 8 | Fair quality |
| Dumitriu 2020 | Yes | Yes | Yes | Yes | Yes | Yes | Yes | NA | NA | NA | No | No | Yes | No | 9.5 | Good quality |
| Facchettia 2020 | Yes | Yes | NR | Yes | NR | Yes | Yes | NA | NA | NA | NR | No | Yes | No | 7 | Poor quality |
| Farghaly 2020 | Yes | Yes | NR | Yes | No | Yes | Yes | NA | NA | NA | NR | No | Yes | Yes | 8 | Fair quality |
| Flaherman 2020 | Yes | Yes | NR | Yes | NR | Yes | Yes | NA | NA | NA | No | No | Yes | No | 7.5 | Poor quality |
| Gale 2020 | Yes | Yes | Yes | Yes | NR | Yes | Yes | NA | NA | NA | No | No | Yes | No | 8 | Fair quality |
| Gaspar 2021 | Yes | Yes | NR | Yes | No | Yes | Yes | NA | NA | NA | NR | No | Yes | No | 7.5 | Poor quality |
| Ghema 2021 | Yes | No | Yes | Yes | NR | Yes | Yes | NA | NA | NA | No | No | Yes | NR | 7.5 | Poor quality |
| Goyal 2020 | Yes | Yes | Yes | Yes | No | Yes | Yes | NA | NA | NA | NR | No | Yes | No | 8.5 | Fair quality |
| Gulersen 2020 | Yes | Yes | NR | Yes | Yes | Yes | Yes | NA | NA | NA | No | No | Yes | No | 8.5 | Fair quality |
| Handlay 2020 | Yes | No | Yes | Yes | NR | Yes | Yes | NA | NA | NA | NR | No | Yes | NR | 7 | Poor quality |
| Hcini 2021 | Yes | Yes | Yes | Yes | No | Yes | Yes | NA | NA | NA | NR | No | Yes | Yes | 9 | Good quality |
| He 2020 | Yes | Yes | Yes | Yes | No | Yes | Yes | NA | NA | NA | NR | No | Yes | No | 8.5 | Fair quality |
| Knight 2020 | Yes | Yes | NR | Yes | No | Yes | Yes | NA | NA | NA | No | No | Yes | No | 8 | Fair quality |
| Abarca 2020 | Yes | Yes | NR | Yes | NR | Yes | Yes | NA | NA | NA | NR | No | Yes | No | 7 | Poor quality |
| Llorca 2021 | Yes | Yes | NR | Yes | No | Yes | Yes | NA | NA | NA | No | No | Yes | No | 7.5 | Poor quality |
| Lokken 2021 | Yes | Yes | NR | Yes | NR | Yes | Yes | NA | NA | NA | No | No | Yes | No | 7.5 | Poor quality |
| Lopian 2020 | Yes | Yes | NR | Yes | NR | Yes | Yes | NA | NA | NA | NR | No | Yes | NR | 6.5 | Poor quality |
| Mahajan 2021 | Yes | Yes | NR | Yes | No | Yes | Yes | NA | NA | NA | No | No | Yes | No | 8 | Fair quality |
| Perez 2021 | Yes | Yes | NR | Yes | Yes | Yes | Yes | NA | NA | NA | NR | No | Yes | Yes | 8.5 | Fair quality |
| Portilla 2020 | Yes | Yes | NR | Yes | Yes | Yes | Yes | NA | NA | NA | NR | No | Yes | Yes | 8.5 | Fair quality |
| Maru 2020 | Yes | Yes | Yes | Yes | NR | Yes | Yes | NA | NA | NA | No | No | Yes | No | 8.5 | Fair quality |
| Mattar 2020 | Yes | Yes | Yes | Yes | NR | Yes | Yes | NA | NA | NA | No | No | Yes | No | 8.5 | Fair quality |
| Mattern 2021 | Yes | Yes | Yes | Yes | NR | Yes | Yes | NA | NA | NA | No | No | Yes | No | 8.5 | Fair quality |
| Zambrano 2020 | Yes | Yes | Yes | Yes | NR | Yes | Yes | NA | NA | NA | No | No | Yes | NR | 8 | Fair quality |
| Molina 2020 | Yes | Yes | NR | Yes | NR | Yes | Yes | NA | NA | NA | NR | No | Yes | No | 7 | Poor quality |
| Moreno 2020 | Yes | Yes | NR | Yes | No | Yes | Yes | NA | NA | NA | No | No | Yes | No | 8 | Fair quality |
| Nambiar 2020 | Yes | No | NR | Yes | NR | Yes | Yes | NA | NA | NA | NR | No | Yes | NR | 6 | Poor quality |
| Nayak 2020 | Yes | Yes | NR | Yes | NR | Yes | Yes | NA | NA | NA | NR | No | Yes | No | 7 | Poor quality |
| Ochiai 2020 | Yes | No | NR | Yes | NR | Yes | Yes | NA | NA | NA | No | No | Yes | NR | 6.5 | Poor quality |
| Oncel 2020 | Yes | Yes | NR | Yes | No | Yes | Yes | NA | NA | NA | NR | No | Yes | No | 7.5 | Poor quality |
| Onwuzurike 2020 | Yes | No | NR | Yes | NR | Yes | Yes | NA | NA | NA | NR | No | Yes | NR | 6 | Poor quality |
| Ozsurmeli 2021 | Yes | Yes | Yes | Yes | NR | Yes | Yes | NA | NA | NA | No | No | Yes | No | 8.5 | Fair quality |
| Shetty 2020 | Yes | Yes | Yes | Yes | No | Yes | Yes | NA | NA | NA | No | No | Yes | No | 9 | Good quality |
| Patberg 2020 | Yes | Yes | Yes | Yes | No | Yes | Yes | NA | NA | NA | NR | No | Yes | Yes | 9 | Good quality |
| Pecks 2020 | Yes | No | NR | Yes | NR | Yes | Yes | NA | NA | NA | No | No | Yes | NR | 6.5 | Poor quality |
| Peng 2020 | Yes | Yes | NR | Yes | Yes | Yes | Yes | NA | NA | NA | NR | No | Yes | No | 8 | Fair quality |
| Pereira 2020 | Yes | Yes | Yes | Yes | No | Yes | Yes | NA | NA | NA | NR | No | Yes | No | 8.5 | Fair quality |
| Rebecca 2020 | Yes | Yes | Yes | Yes | No | Yes | Yes | NA | NA | NA | No | No | Yes | No | 9 | Good quality |
| Pineles 2020 | Yes | No | NR | Yes | NR | Yes | Yes | NA | NA | NA | No | No | Yes | NR | 6.5 | Poor quality |
| Pirjani 2020 | Yes | Yes | NR | Yes | No | Yes | Yes | NA | NA | NA | NR | No | Yes | No | 7.5 | Poor quality |
| Prabhu 2020 | Yes | Yes | NR | Yes | No | Yes | Yes | NA | NA | NA | No | No | Yes | No | 8 | Fair quality |
| Qiancheng 2020 | Yes | Yes | NR | Yes | No | Yes | Yes | NA | NA | NA | NR | No | Yes | No | 7.5 | Poor quality |
| Reale 2020 | Yes | Yes | Yes | Yes | NR | Yes | Yes | NA | NA | NA | No | No | Yes | Yes | 9 | Good quality |
| Silva 2020 | Yes | Yes | Yes | Yes | NR | Yes | Yes | NA | NA | NA | No | No | Yes | Yes | 9 | Good quality |
| Sahin (a) 2020 | Yes | Yes | Yes | Yes | No | Yes | Yes | NA | NA | NA | No | No | Yes | No | 9 | Good quality |
| Sahin (b) 2020 | Yes | Yes | Yes | Yes | NR | Yes | Yes | NA | NA | NA | NR | No | Yes | No | 8 | Fair quality |
| Sakowicz 2020 | Yes | Yes | Yes | Yes | Yes | Yes | Yes | NA | NA | NA | NR | No | Yes | Yes | 9.5 | Good quality |
| Salvatore 2020 | Yes | Yes | Yes | Yes | No | Yes | Yes | NA | NA | NA | No | No | Yes | No | 9 | Good quality |
| Samadi 2021 | Yes | Yes | NR | Yes | Yes | Yes | Yes | NA | NA | NA | NR | No | Yes | Yes | 8.5 | Fair quality |
| Juan 2020 | Yes | Yes | NR | Yes | No | Yes | Yes | NA | NA | NA | NR | No | Yes | No | 7.5 | Poor quality |
| Santana 2021 | Yes | Yes | NR | Yes | NR | Yes | Yes | NA | NA | NA | No | No | Yes | No | 7.5 | Poor quality |
| Santhosh 2021 | Yes | Yes | Yes | Yes | NR | Yes | Yes | NA | NA | NA | No | No | Yes | No | 8.5 | Fair quality |
| Savasi 2020 | Yes | Yes | NR | Yes | NR | Yes | Yes | NA | NA | NA | No | No | Yes | No | 7.5 | Poor quality |
| Cornudella 2020 | Yes | Yes | Yes | Yes | No | Yes | Yes | NA | NA | NA | NR | No | Yes | No | 8.5 | Fair quality |
| Cornudella 2021 | Yes | Yes | Yes | Yes | NR | Yes | Yes | NA | NA | NA | NR | No | Yes | No | 8 | Fair quality |
| Schwartz 2020 | Yes | Yes | NR | Yes | NR | Yes | Yes | NA | NA | NA | No | No | Yes | NR | 7 | Poor quality |
| Sherer 2020 | Yes | Yes | NR | Yes | NR | Yes | Yes | NA | NA | NA | NR | No | Yes | No | 7 | Poor quality |
| Shmakov 2020 | Yes | Yes | NR | Yes | No | Yes | Yes | NA | NA | NA | No | No | Yes | No | 8 | Fair quality |
| Singh 2020 | Yes | Yes | NR | Yes | NR | Yes | Yes | NA | NA | NA | No | No | Yes | No | 7.5 | Poor quality |
| Smithgall 2020 | Yes | Yes | NR | Yes | No | Yes | Yes | NA | NA | NA | No | No | Yes | No | 8 | Fair quality |
| Soffer 2021 | Yes | Yes | Yes | Yes | No | Yes | Yes | NA | NA | NA | NR | No | Yes | No | 8.5 | Fair quality |
| Suyuthi 2020 | Yes | Yes | NR | Yes | NR | Yes | Yes | NA | NA | NA | NR | No | Yes | NR | 6.5 | Poor quality |
| Tug 2020 | Yes | Yes | Yes | Yes | NR | Yes | Yes | NA | NA | NA | No | No | Yes | No | 8.5 | Fair quality |
| Villalaı ´n 2020 | Yes | Yes | Yes | Yes | No | Yes | Yes | NA | NA | NA | NR | No | Yes | No | 8.5 | Fair quality |
| Vintzileos | Yes | Yes | Yes | Yes | NR | Yes | Yes | NA | NA | NA | No | No | Yes | NR | 8.5 | Fair quality |
| Vivanti 2020 | Yes | Yes | Yes | Yes | No | Yes | Yes | NA | NA | NA | No | No | Yes | No | 9 | Good quality |
| Vizheh 2021 | Yes | Yes | NR | Yes | No | Yes | Yes | NA | NA | NA | NR | No | Yes | Yes | 8 | Fair quality |
| Wang (a) 2020 | Yes | Yes | NR | Yes | No | Yes | Yes | NA | NA | NA | No | No | Yes | No | 8 | Fair quality |
| Wang (b) 2020 | Yes | Yes | Yes | Yes | No | Yes | Yes | NA | NA | NA | NR | No | Yes | Yes | 9 | Good quality |
| Wei 2020 | Yes | Yes | Yes | Yes | No | Yes | Yes | NA | NA | NA | NR | No | Yes | Yes | 9 | Good quality |
| Liu 2020 | Yes | Yes | NR | Yes | NR | Yes | Yes | NA | NA | NA | No | No | Yes | No | 7.5 | Poor quality |
| Wu 2020 | Yes | Yes | Yes | Yes | No | Yes | Yes | NA | NA | NA | No | No | Yes | No | 9 | Good quality |
| Xu (a) 2020 | Yes | Yes | Yes | Yes | No | Yes | Yes | NA | NA | NA | NR | No | Yes | No | 8.5 | Fair quality |
| Xu (b) 2020 | Yes | Yes | NR | Yes | NR | Yes | Yes | NA | NA | NA | NR | No | Yes | NR | 6.5 | Poor quality |
| Yan 2020 | Yes | Yes | NR | Yes | NR | Yes | Yes | NA | NA | NA | No | No | Yes | No | 7.5 | Poor quality |
| Rong Yang 2020 | Yes | Yes | Yes | Yes | Yes | Yes | Yes | NA | NA | NA | No | No | Yes | Yes | 10 | Good quality |
| Hui Yang (a) 2020 | Yes | Yes | NR | Yes | NR | Yes | Yes | NA | NA | NA | No | No | Yes | No | 7.5 | Poor quality |
| Hui Yang (b) 2020 | Yes | Yes | NR | Yes | No | Yes | Yes | NA | NA | NA | NR | No | Yes | No | 7 | Poor quality |
| Pu Yang 2020 | Yes | Yes | NR | Yes | NR | Yes | Yes | NA | NA | NA | NR | No | Yes | NR | 6.5 | Poor quality |
| Yao 2021 | Yes | Yes | Yes | Yes | No | Yes | Yes | NA | NA | NA | No | No | Yes | Yes | 9.5 | Good quality |
| Yin 2020 | Yes | Yes | NR | Yes | NR | Yes | Yes | NA | NA | NA | NR | No | Yes | No | 7 | Poor quality |
| Yu 2020 | Yes | Yes | Yes | Yes | NR | Yes | Yes | NA | NA | NA | No | No | Yes | No | 8.5 | Fair quality |
| Lei Zeng 2020 | Yes | Yes | Yes | Yes | NR | Yes | Yes | NA | NA | NA | No | No | Yes | No | 8.5 | Fair quality |
| Yingchun Zeng 2020 | Yes | Yes | NR | Yes | NR | Yes | Yes | NA | NA | NA | NR | No | Yes | No | 7 | Poor quality |
| Zhang 2020 | Yes | Yes | NR | Yes | NR | Yes | Yes | NA | NA | NA | No | No | Yes | No | 7.5 | Poor quality |
| Zou 2020 | Yes | Yes | NR | Yes | No | Yes | Yes | NA | NA | NA | No | No | Yes | NR | 7.5 | Poor quality |

**NA: not applicable, NR: not reported.**

**Table S2**. Risk of bias assessment of the case-control studies using the NIH tool.

| **Studies** | **1. Was the research question or objective in this paper clearly stated and appropriate?** | **2. Was the study population clearly specified and defined?** | **3. Did the authors include a sample size justification?** | **4. Were controls selected or recruited from the same or similar population that gave rise to the cases (including the same timeframe)?** | **5. Were the definitions, inclusion and exclusion criteria, algorithms or processes used to identify or select cases and controls valid, reliable, and implemented consistently across all study participants?** | **6. Were the cases clearly defined and differentiated from controls?** | **7. If less than 100 percent of eligible cases and/or controls were selected for the study, were the cases and/or controls randomly selected from those eligible?** | **8. Was there use of concurrent controls?** | **9. Were the investigators able to confirm that the exposure/risk occurred prior to the development of the condition or event that defined a participant as a case?** | **10. Were the measures of exposure/risk clearly defined, valid, reliable, and implemented consistently (including the same time period) across all study participants?** | **11. Were the assessors of exposure/risk blinded to the case or control status of participants?** | **12. Were key potential confounding variables measured and adjusted statistically in the analyses? If matching was used, did the investigators account for matching during study analysis?** | **Total scores** (Yes = 1, No = 0.5, NR & NA & CD = 0) | **Quality rating:** good (9-12 point) or fair (7-8 point) or poor (0-7 points) |
| --- | --- | --- | --- | --- | --- | --- | --- | --- | --- | --- | --- | --- | --- | --- |
| Anuk 2021 | Yes | Yes | NR | Yes | Yes | Yes | NR | NA | Yes | Yes | Yes | NR | 8 | Fair quality |
| Badr 2020 | Yes | Yes | NR | Yes | Yes | Yes | Yes | NA | Yes | Yes | No | Yes | 9.5 | Good quality |
| Brandt 2020 | Yes | Yes | No | Yes | Yes | Yes | NR | NA | Yes | Yes | No | Yes | 9 | Good quality |
| Jenabi 2020 | Yes | Yes | No | Yes | Yes | Yes | NR | NA | Yes | Yes | No | No | 8.5 | Fair quality |
| Liu 2020 | Yes | Yes | No | Yes | Yes | Yes | NR | NA | Yes | Yes | Yes | Yes | 9.5 | Good quality |
| Rizzo 2021 | Yes | Yes | Yes | Yes | Yes | Yes | NR | NA | Yes | Yes | No | Yes | 9.5 | Good quality |
| Soto 2020 | Yes | Yes | NR | Yes | No | Yes | NR | NA | Yes | Yes | No | Yes | 8 | Fair quality |
| Yazihan 2020 | Yes | Yes | NR | Yes | No | Yes | NR | NA | Yes | Yes | No | NR | 7 | Poor quality |

**NA: not applicable, NR: not reported.**
