# Supplementary figures and images for: Systematic review and meta-analysis of COVID-19 maternal and neonatal clinical features and pregnancy outcomes up to June 3, 2021
Source: AJOG Glob Rep. 2022 Jan 3;2(1):100049. doi: 10.1016/j.xagr.2021.100049 (PMC8720679; doi:10.1016/j.xagr.2021.100049)

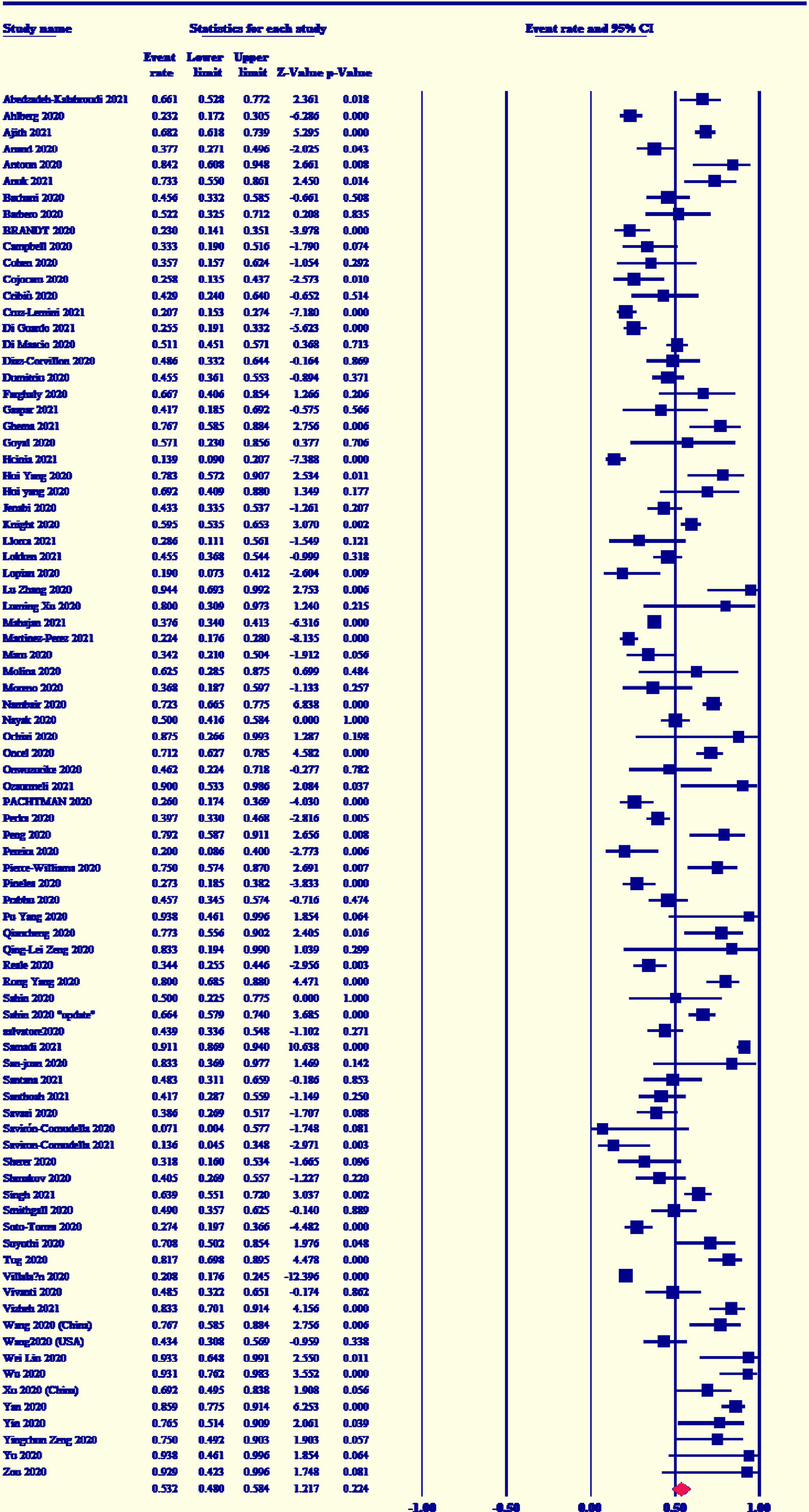

Supplement: Supplementary file 6 [file mmc6.jpg]

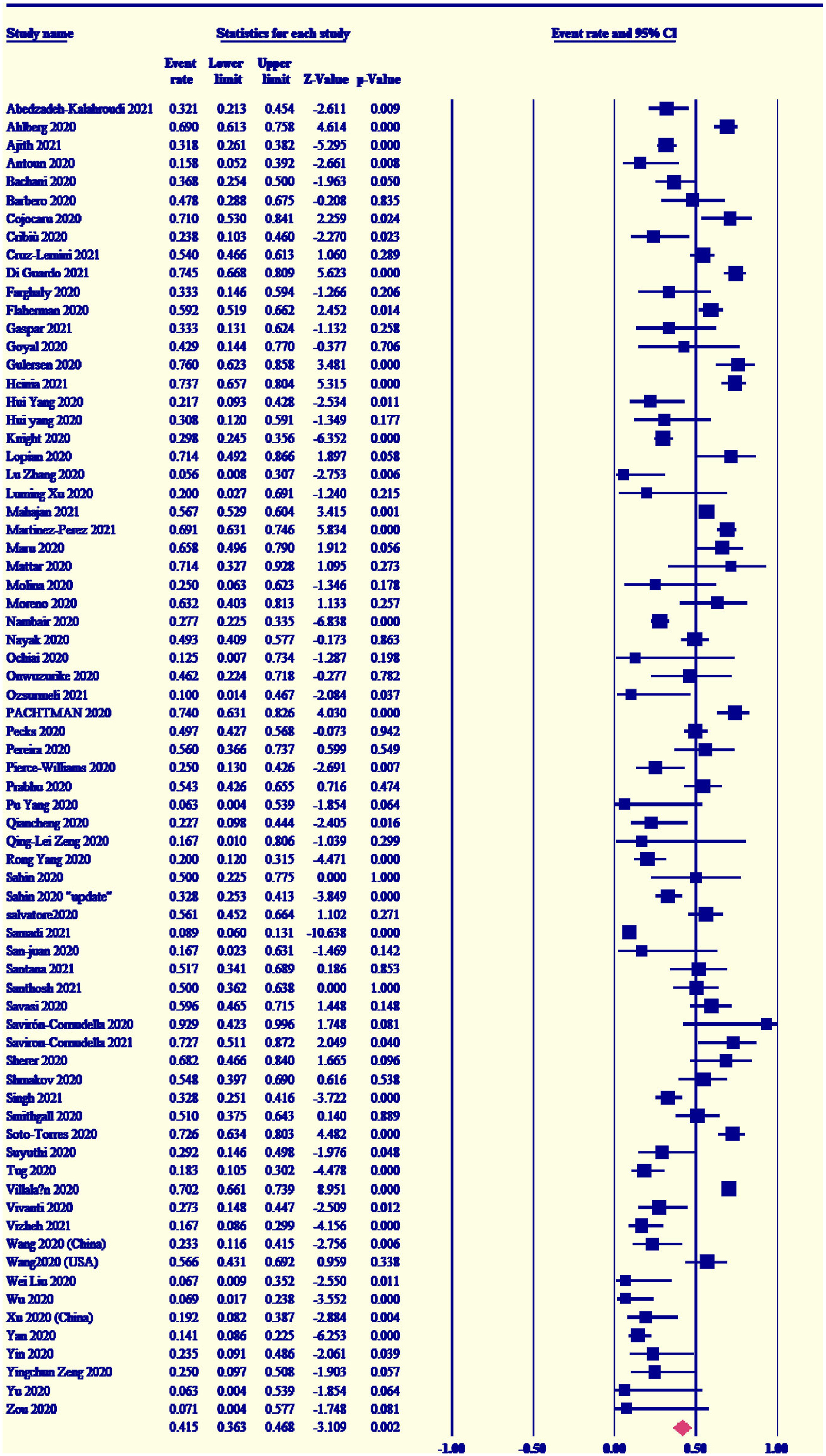

Supplement: Supplementary file 7 [file mmc7.jpg]

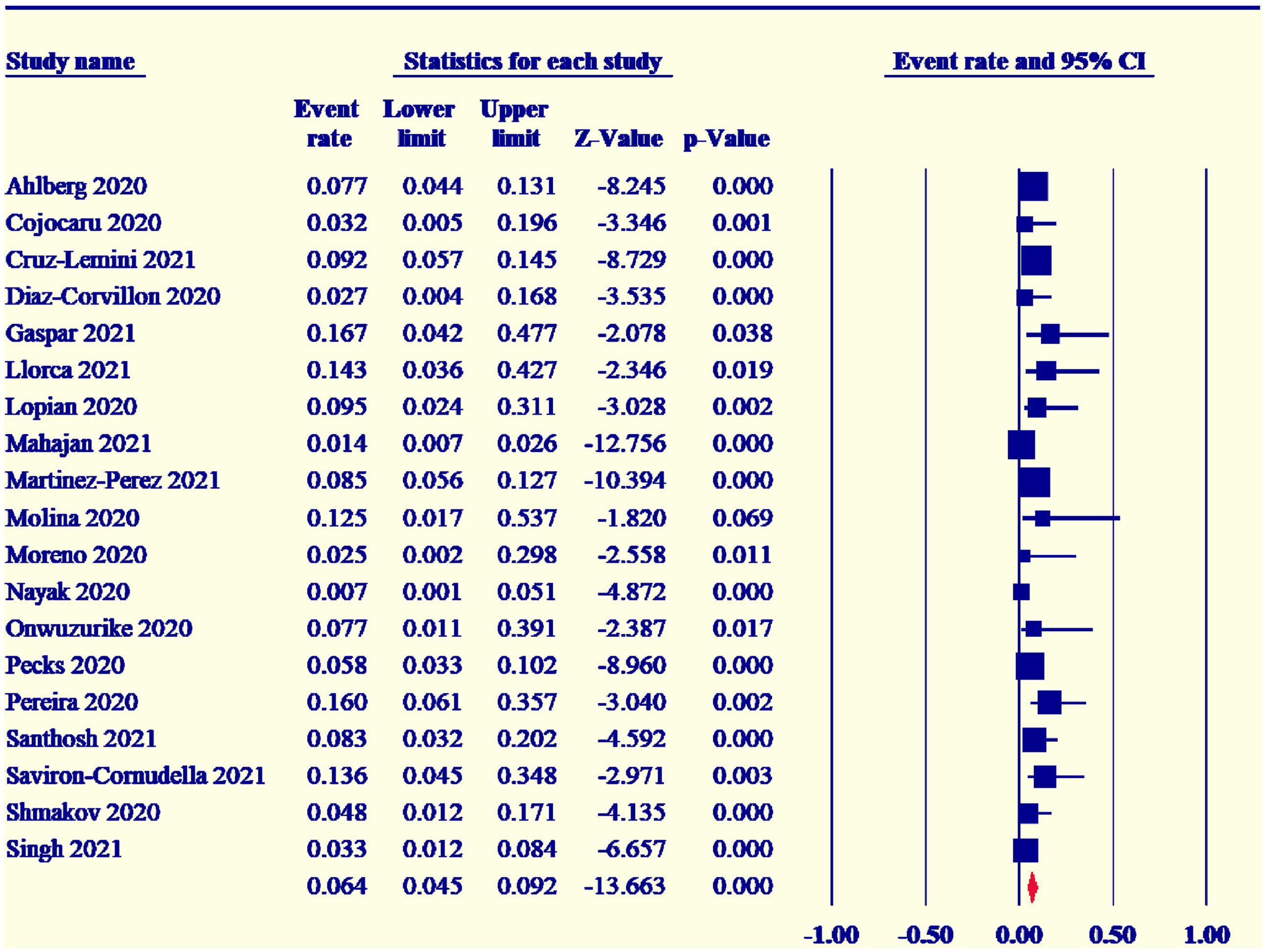

Supplement: Supplementary file 8 [file mmc8.jpg]

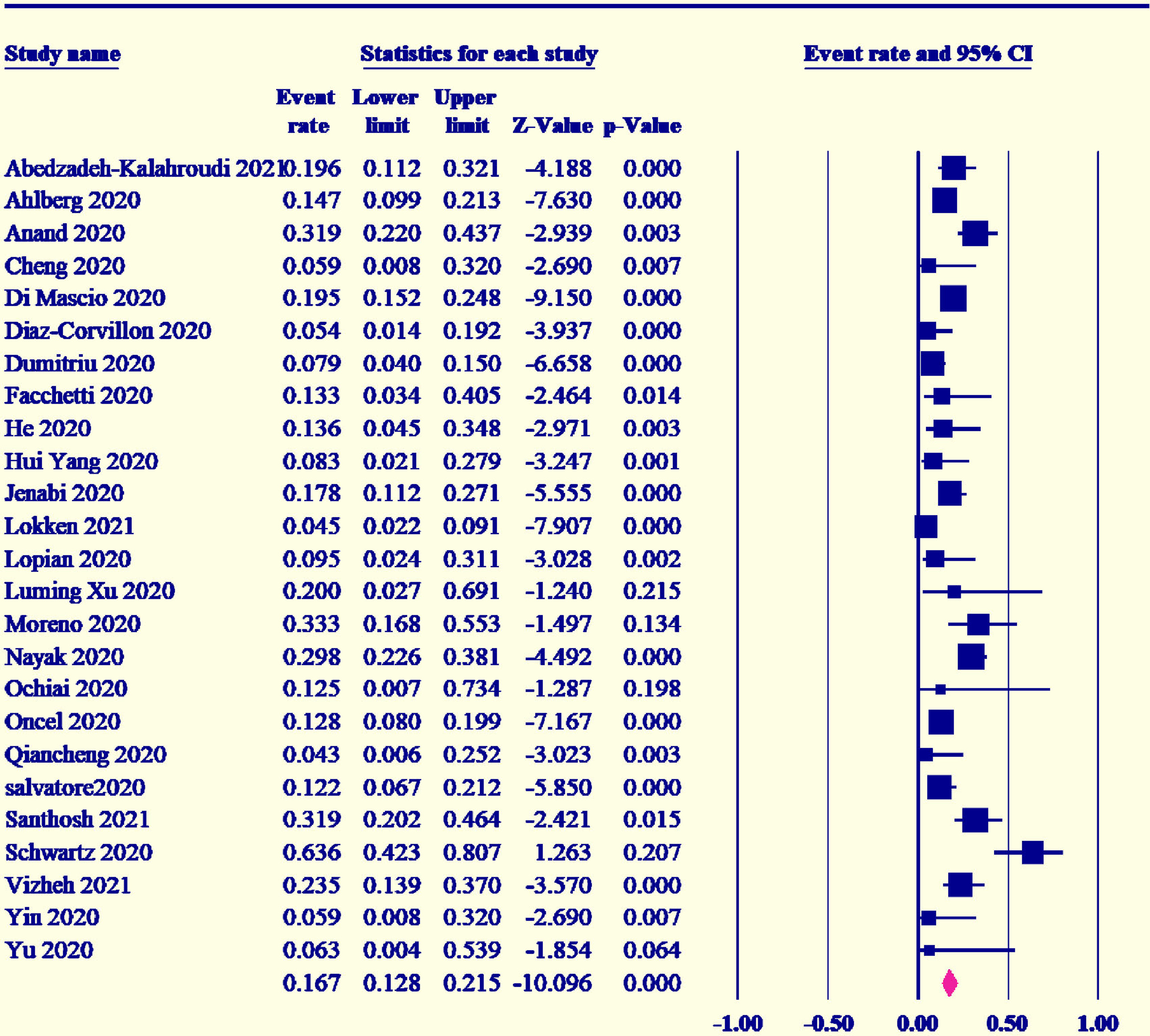

Supplement: Supplementary file 9 [file mmc9.jpg]

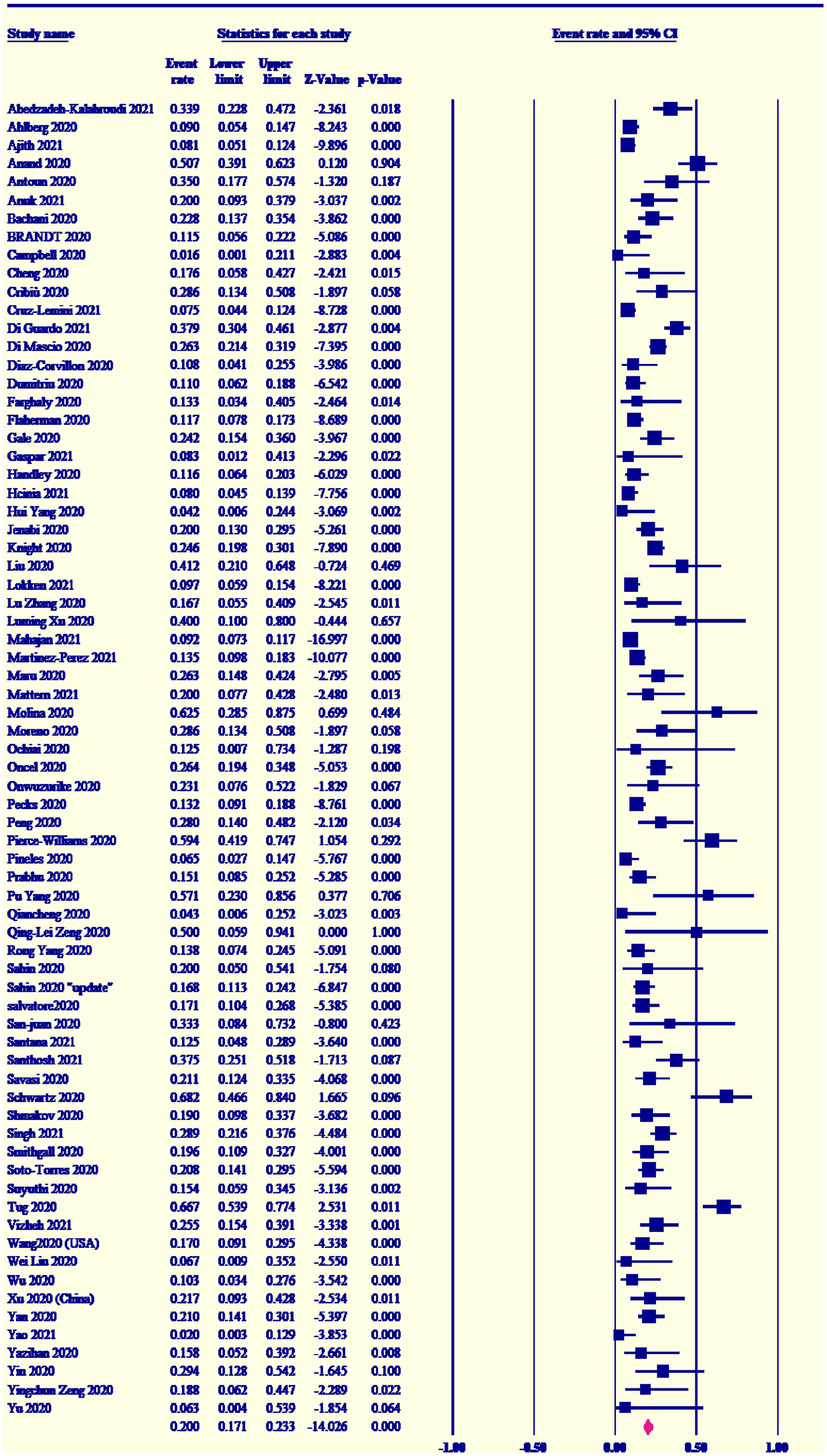

Supplement: Supplementary file 10 [file mmc10.jpg]

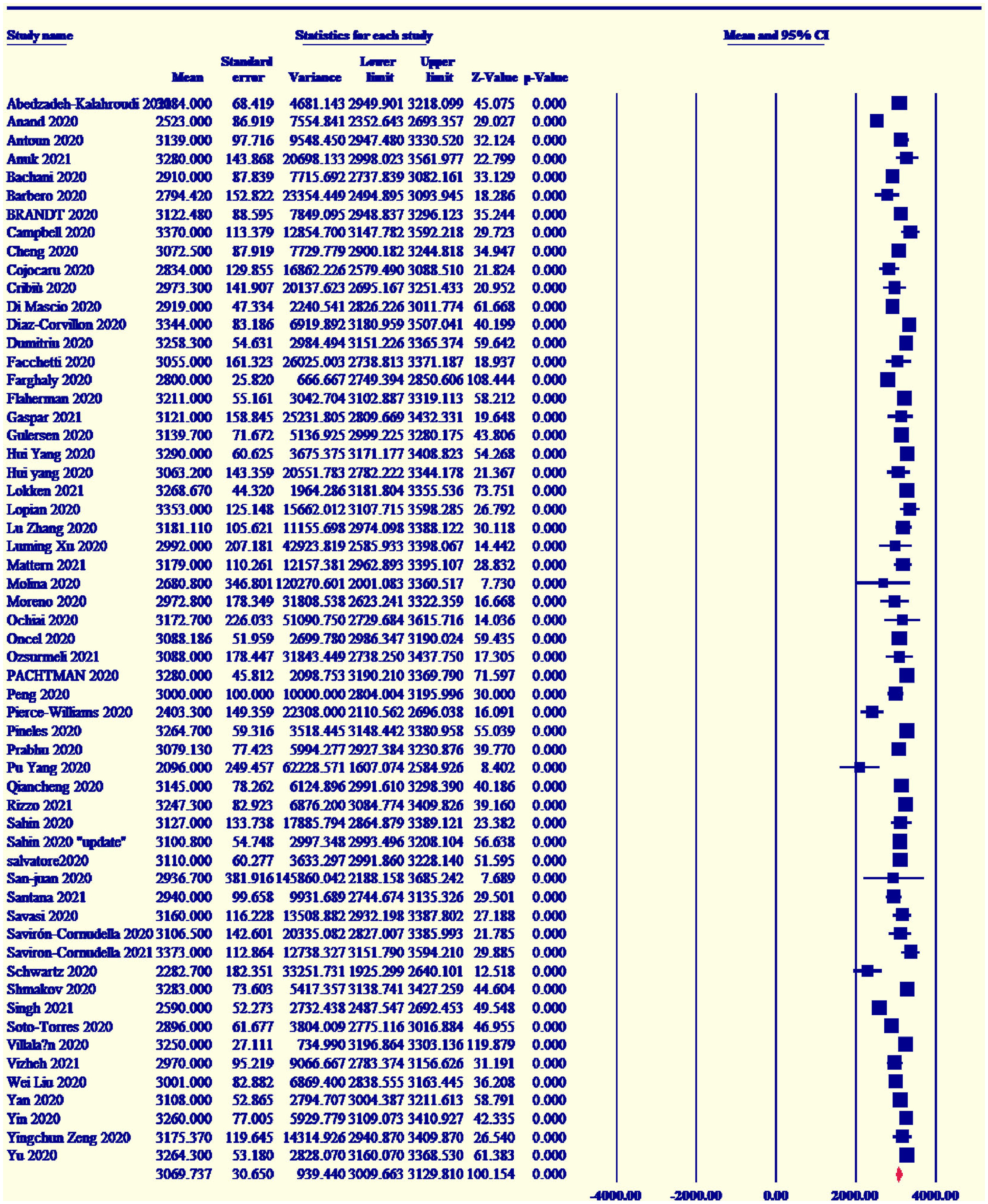

Supplement: Supplementary file 11 [file mmc11.jpg]

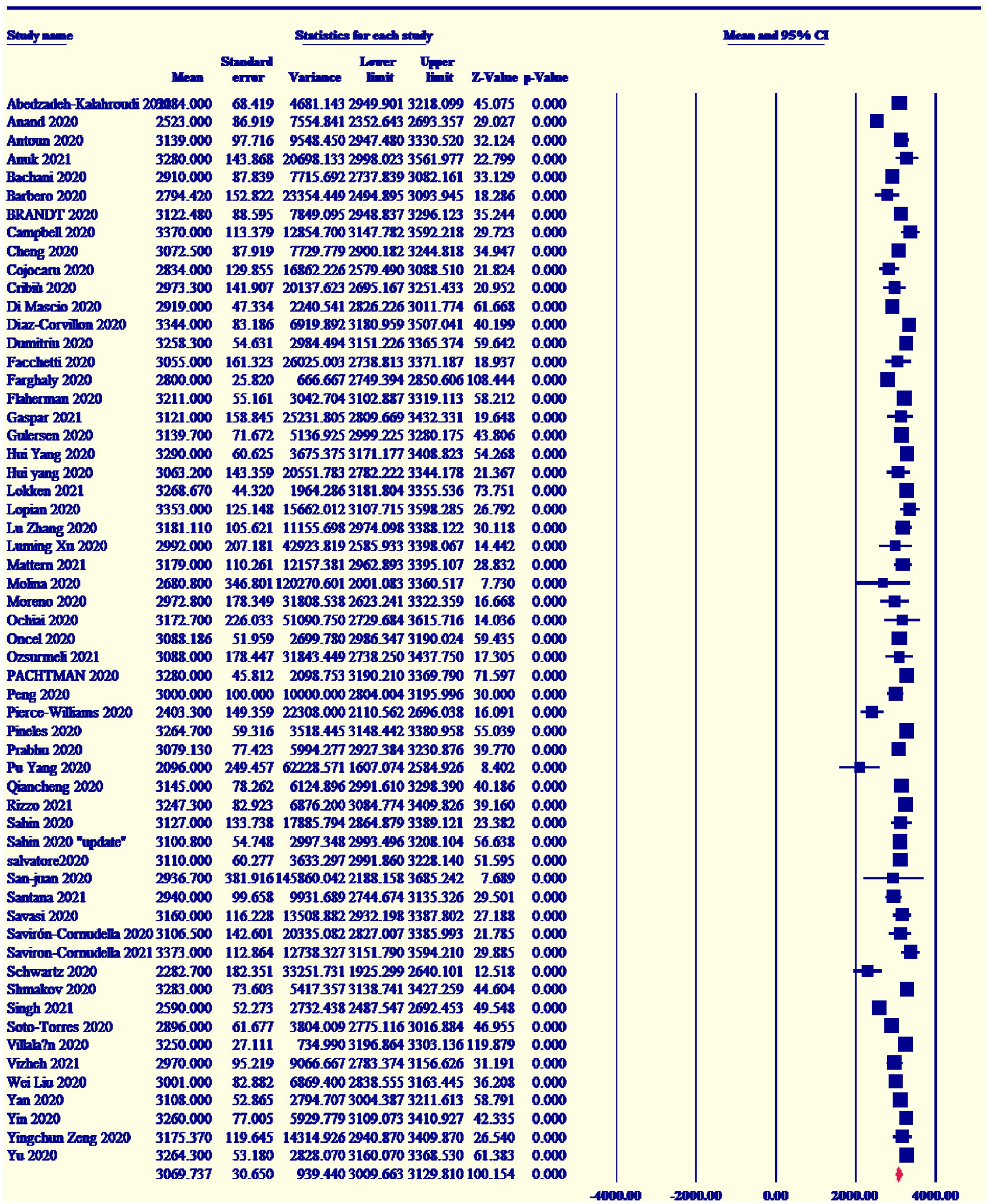

Supplement: Supplementary file 12 [file mmc12.jpg]

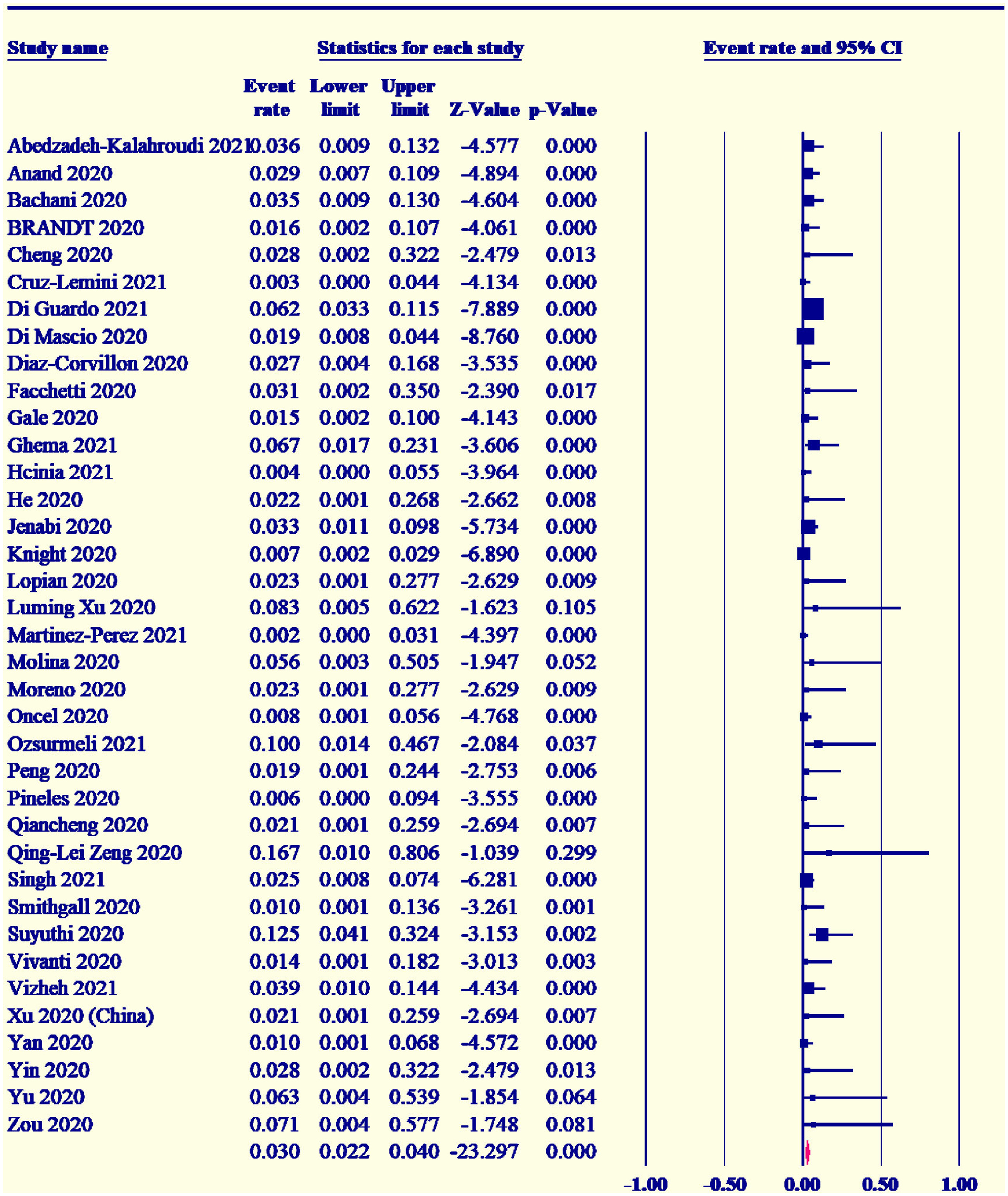

Supplement: Supplementary file 13 [file mmc13.jpg]

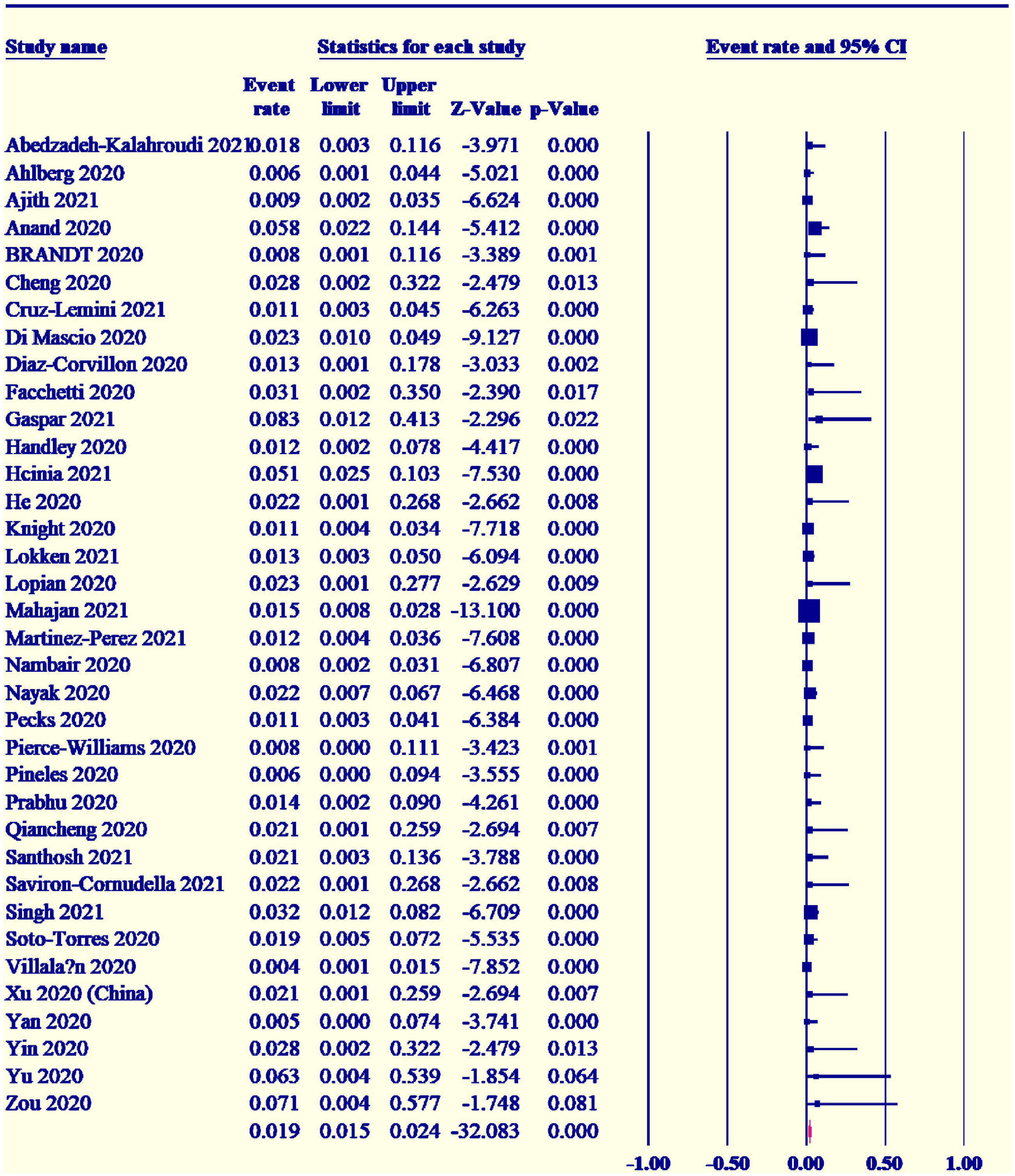

Supplement: Supplementary file 14 [file mmc14.jpg]
